# Intake of ultra-processed foods in Sweden and its association with diet quality and weight status: cross-sectional analyses from three national dietary surveys

**DOI:** 10.1186/s12937-026-01408-5

**Published:** 2026-10-13

**Authors:** Torsten Sällström, Anna Turesson Wadell, Julia Wanselius, Ola Wallengren, Agneta Åkesson, Rikard Landberg, Anna Karin Lindroos, Emma Patterson, Lotta Moraeus, Therese Karlsson, Linnea Bärebring

**Affiliations:** 1 Department of Internal Medicine and Clinical Nutrition, Institute of Medicine, Sahlgrenska Academy, University of Gothenburg, PO Box 459, Gothenburg, SE-405 30 Sweden; 2 Department of Food and Nutrition, and Sport Science, University of Gothenburg, Gothenburg, Sweden; 3 Department of Medical Specialties, Clinical Nutrition Unit, Sahlgrenska University Hospital, Region Västra Götaland, Gothenburg, Sweden; 4 Unit of Cardiovascular and Nutritional Epidemiology, Institute of Environmental Medicine, Karolinska Institutet, Stockholm, Sweden; 5 Department of Life Sciences, Division of Food and Nutrition Science, Chalmers University of Technology, Gothenburg, Sweden; 6 Department of Risk and Benefit Assessment, Swedish Food Agency, Uppsala, Sweden; 7 Department of Food Studies, Nutrition and Dietetics, Uppsala University, Uppsala, Sweden; 8 Department of Global Public Health, Karolinska Institutet, Stockholm, Sweden

**Keywords:** Ultra-processed foods, Nova classification, Dietary assessment, Nutrient density, Diet quality, Overweight and obesity

## Abstract

**Background:**

Ultra-processed foods (UPF) contribute substantially to energy intake in many countries and have been linked to poorer nutrient profiles and increased risk for excess body weight. Swedish data on UPF intake and its relation to diet quality and weight status are lacking.

**Methods:**

Data were derived from the three most recent Swedish national dietary surveys: Riksmaten (RM)–Adults (2010–2011; *n* = 1797), RM–Adolescents (2016–2017; *n* = 3099), and RM–Young Children (2021–2024; *n* = 1820). Classification of UPFs was based on the Nova system using a transparent protocol incorporating recipe disaggregation, independent classification, and alternative classification scenarios to address uncertainty. Associations between energy percent (E%) from UPF and diet quality indicators and micronutrient intakes were examined using quantile regression. Associations between E% UPF and body mass index (BMI or BMI z-score) were assessed using linear regression, and prevalence ratios (PR) of overweight or obesity were estimated using Poisson regression with robust variance. Models were adjusted for sociodemographic factors, with additional adjustment for total energy intake and diet quality.

**Results:**

According to the primary classification scenario, the mean dietary share of UPF was 45 E% among adults, 52 E% among adolescents, and 46 E% among young children. With stricter classification of ambiguous food items, estimates ranged between 23 and 29 E%. A higher dietary share of UPF was associated with less favourable diet quality across most indicators, except for salt and saturated fat in all age groups and iron and vitamin E in young children. Among adults, each 10 E% increment in UPF corresponded to 0.61% higher BMI (95% CI: 0.04–1.18; *p* = 0.036). The prevalence of overweight or obesity was higher in the top versus bottom tertiles of UPF intake after energy adjustment (PR = 1.16; 95% CI: 1.03–1.30; *p* = 0.018). No associations were observed among adolescents or young children.

**Conclusions:**

UPF contributed substantially to energy intake in all age groups, though estimates differed markedly depending on the classification scenario. UPF intake was inversely associated with diet quality across most, but not all, indicators. No robust relationships with BMI were observed.

**Clinical trial number:**

Not applicable.

**Supplementary information:**

The online version contains supplementary material available at https://doi.org/10.1186/s12937-026-01408-5.

## Background

Over recent decades, the global food environment has undergone major changes, with traditional dietary patterns and home-prepared meals increasingly replaced by industrially manufactured products designed for convenience, affordability and palatability [1]. To capture this shift, the degree of food processing has been proposed as a key dimension in evaluating diet quality. The Nova classification system, which categorises foods into four groups based on the nature, extent, and purpose of industrial processing [2] remains the most widely adopted framework, although its underlying assumptions and implications have been the subject of debate [3]. Within this framework, the term ultra-processed foods (UPF) refers to“formulations of ingredients, mostly of exclusive industrial use, typically created by a series of industrial techniques and processes” [4]. In high-income Western countries, UPF accounts for a substantial share of total energy intake (EI), exceeding 50% among adults in the United Kingdom and the United States [5, 6] and reaching approximately 65–67% among children and adolescents [7, 8]. A growing body of research links higher consumption of total UPF with a range of adverse health outcomes; to date, associations have been reported with more than 30 conditions, including cardiometabolic disorders and increased all-cause mortality [9].

In parallel with shifts in the global food environment, the prevalence of overweight and obesity has risen, increasing the burden of related comorbidities and healthcare costs. Current estimates indicate that obesity affects more than two billion individuals worldwide [10]. In Sweden, over half of the adult population is classified as having overweight or obesity, with rising trends across all age groups [11]. Among the modifiable risk factors, dietary patterns play a central role in the accumulation of excess weight. Diets high in UPF are typically energy-dense, low in dietary fibre, and high in sodium, saturated fat and added sugars, while providing fewer essential nutrients [12–15]. In combination with processing-related characteristics such as soft texture and high palatability, this imbalanced nutrient profile has been hypothesized to promote excessive energy intake and contribute to the development of diet-related non-communicable diseases [16]. Associations between UPF consumption and overweight or obesity have been demonstrated in ecological and cohort studies [9, 17–19] and supported by experimental evidence showing that diets high in UPFs promote increased energy intake compared with minimally processed ones [20–22]. However, findings in paediatric populations remain inconsistent, with fewer studies available and mixed results reported [23, 24].

The classification of foods based on processing level has been subject to scrutiny, with concerns about limited precision leading to a heterogeneous category that includes items with potentially beneficial health implications [3, 25]. Health-associated outcomes have been reported to vary between UPF-categories [26] and from a public health policy perspective, it remains debated whether categorizing UPF adds value beyond existing dietary guidelines and nutrient profiling models [27]. Furthermore, commonly used dietary assessment methods and food databases often lack the granularity needed to accurately assess the type and degree of food processing and subsequent effects thereof. Inconsistencies in classification between raters have been highlighted in some studies [28], while others have found more reliable agreement [29].

A previous analysis of national dietary survey data positioned Sweden among the highest consumers of UPF in Europe [30]. However, given the level of detail available for UPF classification, these estimates warrant cautious interpretation, particularly when comparing results across surveys with differing methodologies. In addition, data on UPF consumption among Swedish children and adolescents remain unavailable. To date, no study has examined the relationship between UPF intake, dietary nutrient profile, and weight status in a Swedish context using individual level data from nationally representative samples. The present study was designed to address these gaps while applying a systematic and transparent protocol for UPF classification that explicitly accounts for the inherent uncertainties in defining and categorizing foods according to the Nova system.

### Aim

The primary aim of the present study was to characterize the intake of UPF in the Swedish population using the Nova classification and assess its association with indicators of diet quality, as well as micronutrient intake and density. Additionally, we aimed to investigate the association between UPF consumption and BMI and weight status.

## Methods

### Subjects and study design

Riksmaten (RM) is a recurring, cross-sectional national dietary survey conducted by the Swedish Food Agency. The survey is stratified by age group, with its most recent iterations carried out in 2010–2011 for adults, 2016–2017 for adolescents and 2021–2024 for young children. Their respective methodologies have been described in detail elsewhere [31–33]. In summary, sampling was done in collaboration with the government agency Statistics Sweden with the goal of achieving nationally representative samples. RM–Adults (*n* = 1797 with complete dietary intake data, participation rate 36.6%) consisted of participants 18–80 years old, for RM–Adolescents (*n* = 3099 with complete dietary intake data, participation rate 60%), sampling was based on school level targeting students in grades five, eight and eleven (median ages 11, 14 and 17 years), and RM–Young Children comprised of children in age groups 18 months and 4 years (*n* = 1820 with complete dietary intake data, participation rate 11%).

### Dietary assessment

In RM–Adults, the intake of foods and drinks during four consecutive days was registered prospectively using the Riksmaten Method, a validated web-based food diary complemented by a questionnaire [34, 35]. Besides 1766 participants with four complete registration days, eleven participants with three days and 20 participants with two days were included in the analysis. RM–Adolescents used a novel, biomarker validated, web-based tool (RiksmatenFlex) that includes a dietary registration method based on the repeated 24-hour multiple pass recall [36]. Field staff paid visits to participating schools, by which participants completed their first dietary recall day retrospectively. A second day was registered in part prospectively, and a third day of retrospective registration was randomly assigned to each participant. Only the two retrospective days were included in the analysis.

The same RiksmatenFlex method was used for dietary assessment in RM–Young Children, in the form of a prospective food diary on two non-consecutive days, of which the second day was randomly assigned. Information was provided by the children’s parents or legal guardians. If the child attended daycare, daycare staff recorded the intake, and this was subsequently reported by the parents. For children who were partially breastfed (*n* = 155 in the 18 months age group), the number of breastfeeding-occasions during the registration period were reported and each occasion estimated to provide 59 ml of mother’s milk [37].

Proportionality between weekend and weekdays was achieved in RM–Adults and RM–Adolescents, whereas a larger proportion of weekend-days were registered in RM–Young Children [31–33]. In all three surveys registration periods were planned to reflect variations between seasons. The web-based dietary registration tools were linked to population-specific versions of the Swedish food composition database (Riksmaten adults 2010–2011, adolescents 2016–2017, and young children 2021–2024), comprising 1909, 778, and 1218 food items, respectively [31, 37, 38]. Amounts of reported foods and beverages were estimated using images of portion sizes and household measures.

### Anthropometry and non-dietary variables

In RM–Adults, height in cm and weight in kg were self-reported as integer values. BMI was calculated as kg/m^2^ and values < 18.5 were classified as underweight, 18.5–24.9 as normal weight, 25.0–29.9 as overweight and ≥30 as obesity [39]. In RM–Adolescents, weight and height were measured by Swedish Food Agency field staff to the nearest decimal point at the school visits. For RM–Young Children, parents or legal guardians were asked to report on the weight and height as measured at the last visit to the Children’s Healthcare Centre. In the absence of a recent visit, they were asked to measure it themselves at home. For participants between two and eighteen years old, BMI *z*-score was calculated with the *childsds* R package, using sex, exact age and measured BMI as inputs and reference curves according to Cole & Lobstein [40]. Weight status was assessed according to the International Obesity Task Force (IOTF) cut-offs (BMI *z*-score ≥ 1.310 and 1.244 for overweight and ≥2.288 and 2.192 for obesity for boys and girls respectively).

Information on physical activity and leisure activity was gathered from questionnaires. For adults and adolescents, reported leisure-time physical activity was re-coded into four categories (sedentary, light, moderate and hard). For young children and adolescents, the time spent per day on screen-based activities (including movies, television, computer gaming and social media) was reported for weekdays and weekends. These were combined and weighed to yield a measure of average daily screentime. The parents of the adolescents and young children provided information about their own educational attainment, which was then combined into two groups: ≤12 years representing no university degree and >12 years indicating a university degree or higher. In RM–Adults, information on education was obtained from the population register, whereas smoking status was self-reported [31–33].

### UPF classification

Food and beverage items were classified according to the Nova system, with all items from Nova groups 1–3 grouped together and categorised as non-UPF while category 4 items were classified as UPF. The term UPF in this study comprises both solid foods and beverages. Classification followed an a priori established protocol (available in Additional file 1) based on the operational classification criteria [4, 41] and published best practices guidelines [42]. Briefly, an item was classified as ultra-processed if it was identified as industrially produced and contained at least one ingredient characteristic of UPF production. Such marker ingredients include food substances of no or rare culinary use (e.g. a variety of isolated and modified carbohydrates, proteins and fats) as well as classes of food additives with a cosmetic function, i.e. added to increase the palatability, visual appeal, or other sensory properties of the final product [4].

Classification was done independently by two researchers with context-specific knowledge about the food supply; results were compared and consensus reached through discussion. A comprehensive list of all unique food and beverage items reported across the three surveys was compiled, and each item classified into one of three categories: (i) UPF (Nova Group 4), (ii) non-UPF (Nova Groups 1–3), or (iii) culinary preparations suspected to contain UPF components. Culinary preparations were defined as composite dishes, baked goods or other multi-ingredient items, prepared in a non-industrial setting primarily from ingredients from Nova groups 1–3. When expected to contain UPF-components, they were disaggregated based on recipes retrieved from the Swedish Food Agency’s food composition database (version 2025–06-09), or secondly from a widely used Swedish cookbook [43], and their fraction of UPF expressed as E% was calculated.

When an industrially produced food item could not unequivocally be identified as UPF or non-UPF based on the available dietary information, comparable products from two major Swedish food retailers (Ica AB and Coop Sverige AB) were reviewed to capture the range of commercially available products. If the majority of products were identified as UPF based on the presence of marker ingredients, the food item was classified as UPF in the primary classification and retained for subsequent sensitivity analyses. A conservative approach assuming a lower degree of processing was generally favoured. In a few select cases (notably for unspecified minced or ground meat preparations such as meatballs and patties, and for unspecified pizzas) an assumption of a 50/50 split between UPF and non-UPF was considered most appropriate. For sensitivity analysis, alternative classifications were applied according to three scenarios: The middle-bound scenario represents the most likely degree of processing and is used for all main analyses in this study; the lower-bound assumes the lowest, and the upper-bound the highest realistic degree of processing for each food item. A similar approach has previously been used in large cohorts [44, 45] and is supported by best practice guidelines [42].

Alcoholic drinks and supplements were not included in the original Nova classification system. For this analysis, alcoholic beverages were classified as non-UPF when derived from fermentation of basic ingredients and not containing any marker ingredients, and as UPF if they contained marker ingredients or were produced through distillation [42]. Dietary supplements were classified as UPF. A complete list of food items and their classification according to the three scenarios is available in Additional file 2.

### Food groups

Food items were categorised into 19 main groups according to the FoodEx2 system developed by the European Food Safety Association (EFSA) [46]. To increase granularity, larger groups – such as *Grains and grain-based products*, *Milk and dairy products* and *Composite dishes* – were further divided into subgroups, resulting in a total of 39 food groups. Although sugar-sweetened beverages are not a distinct category in FoodEx2, they were separated from the broader group *Water-based beverages* due to their particular relevance in public health research. In this study, *Sugar-sweetened beverages* are defined as soft drink, syrup-based drinks and sugar-sweetened fruit drinks, with or without carbonation, whereas 100% fruit and vegetable juices are included in the group *Juices*. The group referred to here as *Sandwich, pizza and similar* also encompasses a wide range of “stuffed bread-like cereal products”, including hamburgers, döner kebabs, savoury pies and pastries (Additional file 3).

### Classification of generally encouraged food choices

All food items were dichotomously classified as Generally Encouraged Food-choices (GEF) or not by a researcher not involved in the UPF-classification. Classification criteria were modified from a front-of-pack labelling system developed by the Swedish Food Agency, using a Keyhole symbol to guide consumers to healthier food choices at the points of sale [47]. The Keyhole criteria are tailored to each food category, based on how well an item aligns with official dietary guidelines concerning fat quality, vegetable content and key nutrients such as added sugars, salt, fibre and wholegrain. A limited number of additional food groups were included in this classification of GEF, despite not being eligible for Keyhole-classification under current regulations (details in Additional file 3).

### Dietary variables

As indicators of diet quality, energy-adjusted intakes of protein (E%), dietary fibre (g/1000 kcal), wholegrains (g/1000 kcal), saturated fat (SFA, E%), added sugars (E%) and salt (sodium chloride [NaCl], g/1000 kcal) were calculated. For calculating non-beverage energy density (ED, kcal/g), beverages were excluded whereas soups, yoghurt and similar were retained. The micronutrients assessed were vitamins D, E, C, B_12_ and folate, as well as minerals iron, calcium, potassium and selenium. Nutrient selection was based on those identified as of concern in the Swedish population or in specific subgroups [48–50]. Values for added sugars were not available in RM–Adults or RM–Adolescents and therefore calculated as described elsewhere [51].

Vitamins and minerals were analysed both in energy-adjusted form (expressed as units per 1000 kcal) and as absolute intake compared to the age- and sex-specific Average Requirements (AR) from Nordic Nutrition Recommendation 2023 (NNR 2023) [50]. Provisional AR-values were used for vitamin E, vitamin B_12_, potassium and selenium where AR have not been established (Additional file 3). Micronutrient intakes are presented as originally reported, in contrast to previous publications where the Multiple Source Method has been applied to adjust for long-term intake variability in RM–Adolescents and RM–Young Children [37, 49]. Supplements of vitamins and minerals were not included in RM–Adults or RM–Adolescents. Vitamin D-supplements are recommended in Sweden for all children under the age of 2 years [50] and were included as reported in RM–Young Children.

As a composite measure of nutrient density, a Swedish adaptation of the Nutrient Rich Foods Index 11.3 (NRF11.3-S) was used [52, 53]. It awards positive scores for eleven qualifying nutrients (protein, fibre, iron, folate, vitamins A, C, D, E, magnesium, calcium and potassium) and negative scores for three disqualifying nutrients (SFA, salt and added sugars). Nutrient intake was expressed per 100 kcal to reflect nutrient density rather than adequacy (**Equation A**). Reference values used as denominators in the calculation were derived from NNR 2023 and expressed per 100 kcal based on age- and sex-specific reference EI, using recommended daily intake (RI or similar) for qualifying nutrients and maximum recommended intake (MRI) for disqualifying nutrients (Additional file 3). Component scores for qualifying nutrients were capped at 100% of the reference intake, with the exception of dietary fibre.

[A] $$NRF_{n,m}=\sum\nolimits_{i=1}^{n}\min\left(\frac{\mathrm{Nutrient}_{i,100\,\mathrm{kcal}}}{\mathrm{RI}_{i,100\,\mathrm{kcal}}},1\right)-\sum\nolimits_{j=1}^{m}\frac{\mathrm{Nutrient}_{j,100\,\mathrm{kcal}}}{\mathrm{MRI}_{j,100\,\mathrm{kcal}}}$$

### Ethical considerations

Handling and processing of data was done in pseudonymized form in a secure research environment with limited web access provided by the University of Gothenburg, and all research was conducted according to the guidelines laid down in the Declaration of Helsinki. Ethical approvals for RM–Adults and RM–Adolescents were attained from Uppsala Regional Ethics Committee (No. 2015/190 and No. 2010/060 respectively), and from the Swedish Ethical Review Authority for RM–Young Children (No. 2021–03591) with additional approval to analyse the data at the University of Gothenburg (No. 2025–06848-01). Written or verbal informed consent was obtained from all participants or legal guardians of participants younger than 15 years old.

### Statistical analyses

Analyses were conducted separately for each survey: adults (subgroups 18–39, 40–59, and 60–80 years), adolescents (school grades 5, 8, and 11), and young children (18 months and 4-year). No survey sample weights were applied. Variables were assessed for normality by visual inspection of histograms and Q–Q plots. Numerical variables were reported as either means and ± standard deviations (SD) or medians with interquartile ranges (IQRs) while categorical variables were described using counts and percentages.

For normally distributed data, comparisons between groups were conducted using Welch’s t-test, while Mann–Whitney U test was applied for non-normally distributed data. Two-tailed tests with global significance level of *α* = 0.05 were applied throughout. To account for multiple comparisons, *p*-values for diet quality as well as UPF and nutrient intake were adjusted for false discovery rate using the Benjamini–Hochberg method.

Food group intake data are presented descriptively as population-level E%, without measures of dispersion due to large proportions of zero-reporters and highly skewed distributions. Inter-rater reliability for UPF classification was assessed using weighted Cohen’s kappa (κ) and interpreted according to the criteria of Landis & Koch where κ = 0.41–0.60 is considered“moderate” and κ = 0.61–0.80“substantial” strength of agreement [54]. Statistical analyses and data transformations were performed in the R statistical computing environment (v.4.5.0; R Core Team, Vienna, Austria).

Associations between UPF intake, modelled as a continuous variable, and study outcomes were evaluated using regression models appropriate to the outcome distribution. For diet quality outcomes, associations were estimated using multivariable quantile regression at the median (τ = 0.5), with 2,000 bootstrap replications (R package *quantreg*). Quantile regression was used to obtain robust estimates under skewed nutrient intake distributions and to limit the influence of extreme values. Linearity of UPF intake in quantile regression models was evaluated using exploratory restricted cubic spline analyses, which did not indicate meaningful departures from linearity. Quintiles of UPF intake were used for descriptive purposes only. For BMI or BMI z-score outcomes, multivariable ordinary least squares regression was applied. BMI was natural log-transformed in RM–Adults to improve adherence to model assumptions, and results were back-transformed and presented on the original scale with 95% confidence intervals (CIs). Prevalence ratios (PR) for overweight or obesity (yes/no) across UPF tertiles were estimated using Poisson regression with robust variance [55, 56].

Potential confounders were selected based on prior literature and subject-matter knowledge regarding factors likely to be associated with both UPF intake and the study outcomes, while maintaining a consistent modelling strategy across the three study populations. Three models were constructed: (1) a model including age, sex and educational attainment of adult participants or caregiver of children and adolescents (binary: ≤12 vs. >12 years). For adults and adolescents, leisure-time physical activity (sedentary, light, regular, hard) was also included, while daily screen time (continuous) served as a proxy for young children. Smoking status (never, former, occasional or daily) was additionally included for adults; (2) model 1 plus total EI to distinguish associations related to dietary composition from those potentially explained by differences in overall reported EI, and (3) model 2 plus NRF11.3-S score as an indicator of diet quality. Model 1 was considered the primary model in all analyses.

#### Exclusion and imputation

Analyses of nutrient intakes in relation to UPF intake were conducted on a complete-case basis, excluding participants with missing covariate data (7.5% of adults, 6.5% of adolescents, 0.9% of young children). For weight outcomes, 209 adolescents (6.7%) aged > 18 years were excluded to ensure a consistent analytical sample across BMI z-score and weight status analyses, as the selected BMI z-score reference curves and corresponding weight status cut-offs are defined only up to 18 years of age. Similarly, the 18-month-old age group from RM-Young Children was excluded because these reference curves and cut-offs are not defined for children younger than two years [40].

Across all age strata, participants with missing data for either weight or height were excluded (adults: *n* = 124 [6.9%]; adolescents: *n* = 25 [0.9%]; 4-year-olds: *n* = 78 [10.5%]). Two adults and three 4-year-olds were excluded due to implausible values (BMI < 12 kg/m^2^, BMI *z*-score < −5 or > +5). Final analytical samples for the analyses of weight outcomes were: adults; *n* = 1671, adolescents; *n* = 2865, and 4-year-olds *n* = 665 (flowchart in Additional file 3). Missing covariates were imputed using multiple imputation by chained equations (*mice* R package) with 20 datasets and pooled by Rubin’s rules [57]. Imputed values were; adults – education (*n* = 2 [0.1%]), leisure-time physical activity (*n* = 10 [0.6%]), smoking (*n* = 4 [0.2%]); adolescents – parental education (*n* = 170 [5.8%]), leisure-time physical activity (*n* = 20 [0.7%]); 4-year-olds – screen time (*n* = 2 [0.3%]).

#### Sensitivity analyses

To address uncertainties in UPF classification, analyses were repeated under alternative lower- and upper-bound classification scenarios. To account for potential misreporting in analyses of BMI, BMI *z*-score or weight status, a predictive interval of plausible EI was calculated as proposed by Bajunaid et al. [58] (Additional file 3). Participants with reported EI outside the predictive interval were flagged as possible misreporters and excluded in a sensitivity analysis. In the analytical samples, the number of potential misreporters were *n* = 308 (18.4%) for adults, of which 303 underreporters, *n* = 560 (19.5%) for adolescents, of which 491 underreporters, and *n* = 4 (0.6%) among 4-year-olds (1 underreporter) (Additional file 3).

## Results

### Population characteristics

The characteristics of the three study populations are presented in Table 1. In both RM–Adults and RM–Adolescents, females made up a slight majority, whereas RM–Young Children showed the opposite pattern. Educational attainment was higher than in the general population among adults and parents of young children, while parents of the adolescent sample were more representative in this respect.

**Table 1:** Characteristics of the study populations

	RM–Adults (*n* = 1797)			RM–Adolescents (*n* = 3099)						RM–Young Children (*n* = 1820)			
				School grade 11 (*n* = 1000; [>18 ys *n* = 209])		School grade 8 (*n* = 1050)		School grade 5 (*n* = 1049)		4 years (*n* = 746)		18 months (*n* = 1074)	
Sex, girls/females, *n (%)*	1005	(55.9)		577	(57.7)	574	(54.7)	559	(53.3)	364	(48.8)	510	(47.5)
Age^*^, ys, *median (min;max)*	48	(18; 80)		17	(16; 21)	14	(11; 17)	11	(10; 13)	4	(3; 4)	1	(1; 1)
Weight, kg, *median (IQR)*	74.0	(64.5–85.0)		65.6	(58.7–67.9)	56.9	(51.1–64.0)	42	(36.3–48.7)	17.0	(15.6–18.4)	11.2	(10.3–12.2)
Missing *n (%)*	7	(0.4)		5	(0.5)	3	(0.3)	18	(1.7)	73	(9.8)	70	(6.5)
Height, cm, *median (IQR)*	171	(165–179)		171.4	(165.1–179.1)	167.2	(162.4–173)	151.0	(146.1–156.5)	104.0	(101.0–107.0)	81.0	(78.0–84.0)
Missing, *n (%)*	123	(6.8)		5	(0.5)	3	(0.3)	18	(1.7)	73	(9.8)	72	(6.7)
BMI, (>18 ys), *median (IQR)*	24.7	(22.5–27.6)		22.4	(20.3–25.0)	-	-	-	-	-	-	-	-
Missing, *n (%)*	124	(6.9)		1	(0.5)	-	-	-	-	-	-	-	-
BMI *z*-score, (2–18 ys), *median (IQR)* -		-		0.54	(−0.07–1.22)	0.38	(−0.25–1.00)	0.39	(−0.28–1.15)	0.06	(−0.52–0.68)	-	-
Missing, *n (%)*	-	-		4	(0.5)	3	(0.3)	18	(1.7)	78	(10.5)		
Weight status^†^													
Underweight, *n (%)*	26	(1.4)		56	(5.6)	76	(7.2)	82	(7.8)	87	(11.7)	-	-
Normal weight, *n(%)*	867	(48.2)		704	(70.4)	792	(75.4)	723	(68.9)	520	(69.7)	-	-
Overweight, *n (%)*	573	(31.9)		180	(18.0)	146	(13.9)	188	(17.9)	49	(6.6)	-	-
Obesity, *n (%)*	207	(11.5)		55	(5.5)	33	(3.1)	38	(3.6)	12	(1.6)	-	-
Missing, *n (%)*	124	(6.9)		5	(0.5)	3	(0.3)	18	(1.7)	78	(10.5)	-	-
Not applicable, *n (%)*	-	-		-	-	-	-	-	-	-	-	1074	(100)
Education^‡^													
≤12 years, *n (%)*	1000	(55.6)		401	(40.1)	351	(33.4)	372	(35.5)	102	(13.7)	136	(12.7)
>12 years, *n (%)*	794	(44.2)		531	(53.1)	613	(58.4)	637	(60.7)	644	(86.3)	938	(87.3)
Missing, *n (%)*	3	(0.2)		68	(6.8)	86	(8.2)	40	(3.8)	0	(0)	0	(0)
Leisure time physical activity level													
Sedentary, *n (%)*	167	(9.3)		310	(31.0)	244	(23.2)	182	(17.3)	-	-	-	-
Light, *n (%)*	710	(39.5)		216	(21.6)	172	(16.4)	238	(22.7)	-	-	-	-
Moderate, *n (%)*	394	(21.9)		234	(23.4)	372	(35.4)	433	(41.3)	-	-	-	-
Hard, *n (%)*	509	(28.3)		233	(23.3)	257	(24.5)	184	(17.5)	-	-	-	-
Missing, *n (%)*	17	(0.9)		7	(0.7)	5	(0.5)	12	(1.1)	-	-	-	-
Screentime, hours/d, *median (IQR)* -		-		-	-	-	-	-	-	1.3	(0.8–1.7)	0.5	(0.2–0.9)
Missing, *n (%)*	-	-		-	-	-	-	-	-	5	(0.7)	6	(0.6)
Smoking status^§^													
Never smoked, *n (%)*	906	(50.4)		-	-	-	-	-	-	-	-	-	-
Former smoker, *n (%)*	503	(28.0)		-	-	-	-	-	-	-	-	-	-
Occasionally, *n (%)*	123	(6.8)		-	-	-	-	-	-	-	-	-	-
Daily, *n (%)*	144	(8.0)		-	-	-	-	-	-	-	-	-	-
Missing, *n (%)*	121	(6.7)		-	-	-	-	-	-	-	-	-	-

Age presented as completed years. ^†^ Weight status not evaluated in the 18 months age group; ^‡^Highest attained education among parents in RM–Adolescents and RM–Young Children; ^§^Smoking status not evaluated in adolescents. Abbreviations: IQR, interquartile range (p25–p75); RM, Riksmaten (Swedish national dietary survey)

### Intake and major sources of UPF

Of the 2218 unique food items reported in the three RM surveys, 990 (44.6%) were classified entirely as UPF according to the middle-bound scenario and an additional 370 (16.7%) were presumed to contain some UPF components. Overall inter-rater reliability for UPF classification was interpreted as“substantial”, with a weighted Cohen’s *κ* of 0.75 (95% CI: 0.72–0.78) and 86.9% agreement between two raters. Reliability varied between food groups (Additional File 3). For example, *κ* was 0.38 (95% CI: 0.17–0.58; 61% agreement) for *Sandwiches, pizzas and similar*, whereas *Sugar-sweetened beverages* and *Children’s food* showed 100% agreement (*κ* not estimable and *κ* = 1.0, respectively). In total, 788 items (35.5%) were classified according to alternative scenarios for sensitivity analysis.

The mean contribution of UPF to total EI was 45 E% in RM–Adults, 52 E% in RM–Adolescents and 46 E% in RM–Young Children according to the middle-bound scenario, compared to 23 E%, 26 E% and 29 E%, respectively under the lower-bound scenario (Table 2). Intake according to the upper-bound scenario was 52 E%, 63 E% and 53 E%, respectively. When energy-adjusted, significant sex differences in UPF intake were observed only in RM–Adults, with higher E% among men. Mean UPF intake varied across age groups. The proportion of energy derived from UPF increased from childhood through adolescence, reached its highest value in Grade 11, and declined in older age groups, with the lowest mean intake observed among adults aged 60–80 years.

**Table 2:** Absolute and relative intakes of ultra-processed food by age group and sex, with unadjusted comparisons between sexes

	n	Middle bound scenario^‡^				Lower bound scenario^‡^				Upper bound scenario^‡^			
		Kcal		E%		Kcal		E%		Kcal		E%	
		Median (IQR)		Mean (±SD)		Median (IQR)		Mean (±SD)		Median (IQR)		Mean (±SD)	
**RM–Adults**	**1797**	**847**	**(600–1146)**	**44.9**	**(±13.3)**	**409**	**(255–602)**	**22.5**	**(±11.0)**	**970**	**(715–1283)**	**51.5**	**(±13.7)**
Women	1005	741	(543–992)	43.9	(±13.6)	353	(219–510)	21.9	(±11.2)	876	(635–1130)	50.6	(±14.0)
Men	792	999	(728–1332)	46.2	(±12.8)	479	(319–699)	23.2	(±10.7)	1162	(837–1504)	52.5	(±13.4)
Difference (95% CI)		−250	(−287, −213)	−2.3	(−3.6, −1.1)	−122	(−146, −99)	−1.3	(−2.3, −0.3)	−269	(−309, −228)	−1.9	(−3.2, −0.6)
* p* for sex		*p < 0.001*		*p = 0.001*		*p < 0.001*		*p = 0.040*		*p < 0.001*		*p = 0.011*	
* Age groups*													
60–80 years	533	734	(526–993)	40.9	(±12.1)	342	(213–485)	19.4	(±9.2)	875	(623–1122)	46.9	(±12.2)
40–59 years	678	862	(620–1138)	44.6	(±13.3)	415	(256–627)	22.7	(±11.4)	977	(723–1283)	51.0	(±13.7)
18–39 years	586	938	(687–1284)	48.8	(±13.4)	466	(303–681)	25.1	(±11.3)	1092	(814–1442)	56.2	(±13.7)
**RM–Adolescents**	**3099**	**1026**	**(702–1399)**	**51.8**	**(±15.8)**	**477**	**(244–767)**	**25.9**	**(±15.4)**	**1254**	**(884–1684)**	**63.3**	**(±15.6)**
Girls	1710	959	(651–1290)	52.1	(±15.6)	433	(233–703)	25.9	(±14.9)	1166	(837–1536)	63.5	(±15.1)
Boys	1389	1112	(774–1562)	51.4	(±16.2)	527	(273–852)	25.9	(±16.0)	1389	(1002–1910)	63.1	(±16.2)
Difference (95% CI)		−174	(−211, −137)	0.7	(−0.5, 1.8)	−79	(−105, −53)	0.0	(−1.1, 1.1)	−232	(−274, −189)	0.3	(−0.8, 1.4)
* p* for sex		*p < 0.001*		*p = 0.258*		*p < 0.001*		*p = 0.942*		*p < 0.001*		*p = 0.570*	
* Age groups*													
11th grade	1000	1109	(758–1549)	52.9	(±16.7)	511	(265–830)	26.4	(±15.6)	1391	(973–1863)	64.9	(±16.2)
8th grade	1050	1040	(715–1434)	51.2	(±15.4)	480	(256–789)	25.8	(±15.7)	1271	(902–1712)	62.6	(±15.6)
5th grade	1049	937	(644–1252)	51.2	(±15.3)	441	(229–696)	25.5	(±14.9)	1147	(831–1490)	62.5	(±15.0)
**RM–Young Children**	**1820**	**567**	**(412–747)**	**46.3**	**(±15.5)**	**347**	**(211–497)**	**29.2**	**(±14.9)**	**647**	**(477–842)**	**52.7**	**(±15.5)**
Girls	874	548	(394–712)	45.7	(±15.4)	334	(204–476)	28.9	(±14.8)	617	(459–808)	52.2	(±15.4)
Boys	946	589	(423–786)	46.8	(±15.5)	359	(219–525)	29.4	(±15.0)	673	(493–876)	53.2	(±15.6)
Difference (95% CI)		−48	(−71, −24)	−1.1	(−2.5, 0.4)	−27	(−46, −8)	−0.5	(−1.9, 0.9)	−50	(−74, −25)	−1.1	(−2.5, 0.4)
* p* for sex		*p < 0.001*		*p = 0.215*		*p = 0.005*		*p = 0.711*		*p < 0.001*		*p = 0.222*	
*Age groups*													
Four years	746	662	(487–857)	47.7	(±14.5)	386	(254–543)	28.9	(±13.6)	759	(575–953)	54.6	(±14.7)
18 months	1074	507	(365–663)	45.3	(±16.0)	322	(191–467)	29.4	(±15.7)	575	(437–750)	51.4	(±16.0)

Middle-bound representing the most likely degree of processing, lower-bound the lowest realistic degree of processing and upper-bound the highest realistic degree of processing; Difference refers to median or mean differences between sexes expressed as Female–Male. Hodges-Lehman (HL) median difference with 95% CI and Mann–Whitney U test *p*-value for skewed outcomes; Mean difference with 95% CI and *p*-value from Welch’s t-test for approximately normal outcomes. Benjamini-Hochberg correction for multiple comparisons was applied for both tests. Abbreviations: E%, energy percentage; IQR, inter quartile range; RM, Riksmaten (Swedish national dietary survey); SD, standard deviation

**Fig. 1:**
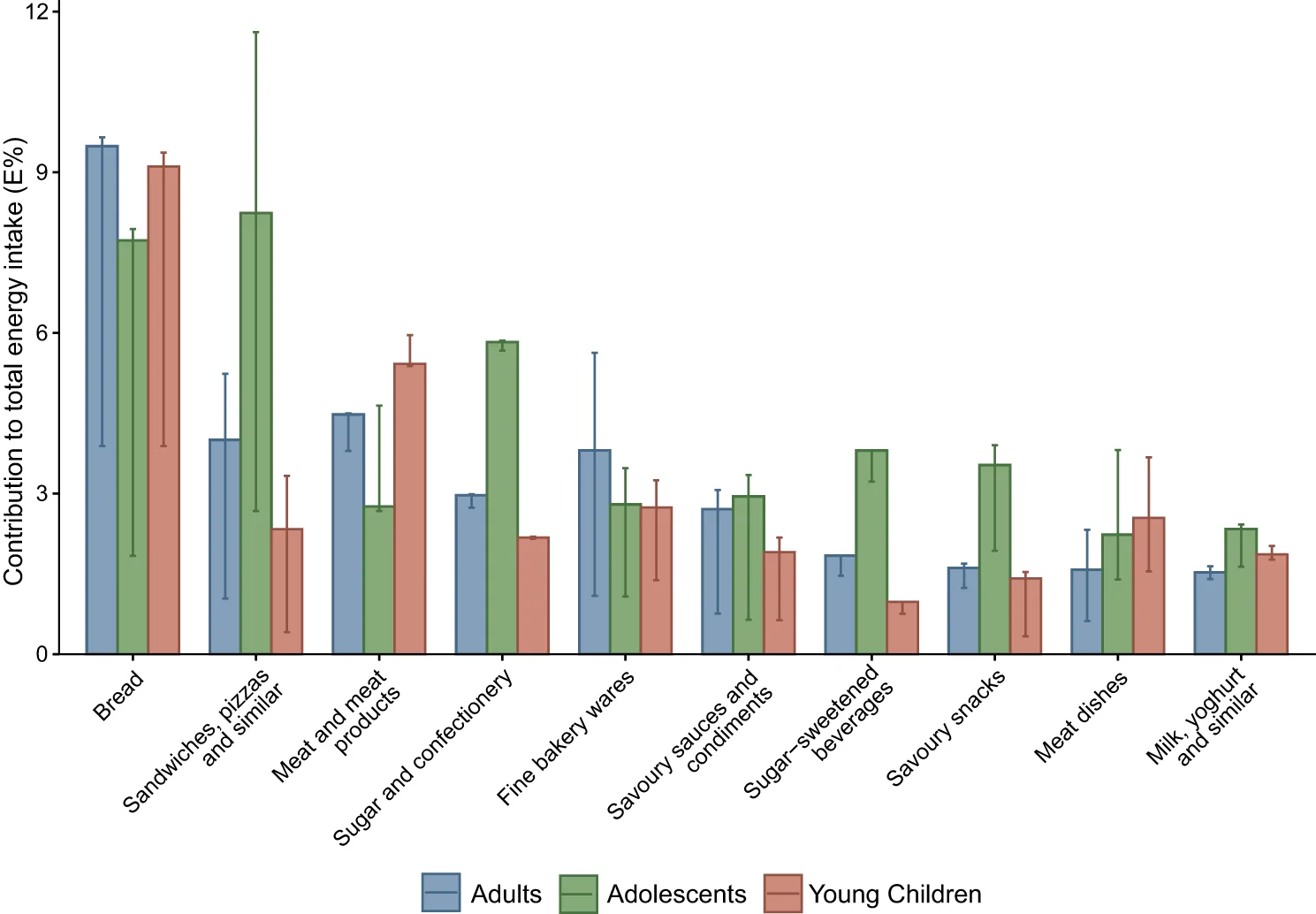
Top ten food groups for energy contribution from ultra-processed food in Riksmaten (RM)–adults, RM–adolescents and RM–young children. Error-bars show alternative classification scenarios (lower-bound assuming lowest realistic degree of processing and upper-bound assuming the highest realistic degree of processing). Values calculated at population-level and expressed as percentage of total energy intake. Details in Additional file 3

At the population level, *Bread* was the largest contributor of UPF-derived calories in RM–Adults and RM–Young Children, representing 9.5% and 9.1% of total EI respectively. *Sandwiches, pizzas and similar* was the main contributor in RM–Adolescents with 8.2% of EI (Fig. 1 &amp ; Additional file 3). *Sugar and confectionery, Sugar-sweetened beverages* and *Savoury snacks* ranked higher among adolescents than in other age groups. Among 18 months old children, *Children’s cereal products* were the main source of energy from UPF (10.5 E%). Under the lower-bound scenario, *Sugar and confectionery* and *Sugar-sweetened beverages* were the two dominant sources of UPF in RM–Adolescents, while the contribution of *Bread* decreases to 3.9% of EI in RM–Adults and RM–Young Children. Sex differences were observed from 5th grade onwards: females derived a greater share of UPF energy from *Sugar and confectionery* and *Fine bakery wares*, whereas males derived more from *Meat and meat products*, *Meat dishes*, and *Sandwiches, pizzas and similar* (Additional file 3).

### Association between UPF-intake with diet quality and nutrient intake

**Fig. 2:**
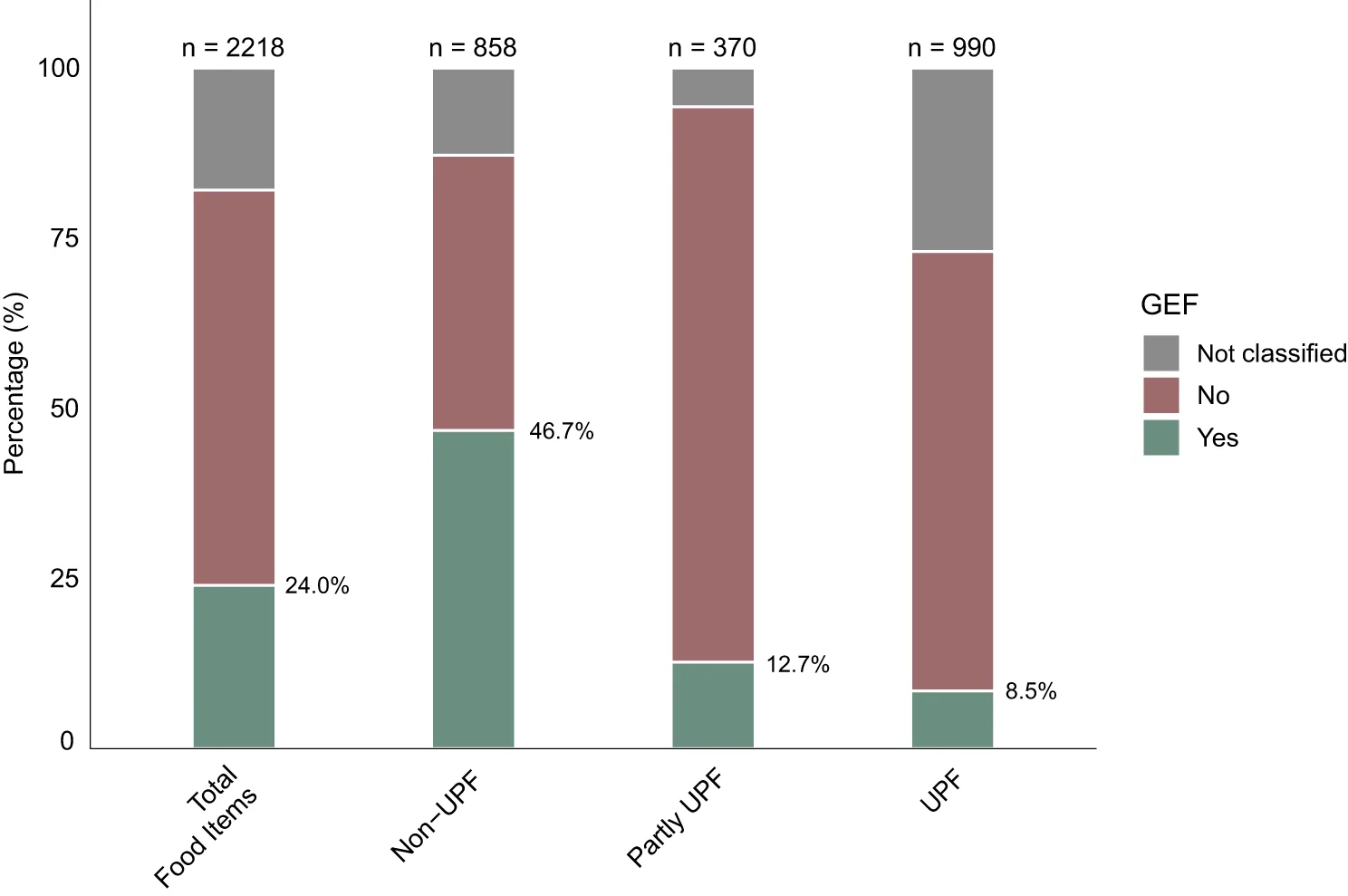
Proportion of food items from the Swedish national dietary surveys that meet the criteria for generally encouraged food choices (GEF), grouped by their classification as ultra-processed food (UPF) according to the Nova-system. “Not classified” refers to items from food groups not eligible for classification

Overall, 24% of food items met the criteria for GEF. (Fig. 2). Of food items that were classified as UPF, less than 10% (8.5%) were considered GEF. Of food items that were classified as non-UPF, less than half (46.7%) were considered GEF. When expressed as share of energy, GEF contributed 22.7 (IQR: 15.3–31.8) E% among adults, 11.5 (IQR: 6.0–19.0) E% among adolescents and 19.2 (IQR: 12.3–28.2) E% among young children. Within the fractions consisting of GEF, the median E% from items classified as UPF were 17.4 (IQR: 4.5–33.0), 0.0 (IQR: 0.0–0.5) and 4.6 (IQR: 0.0–19.6) among adults, adolescents and young children respectively.

**Fig. 3:**
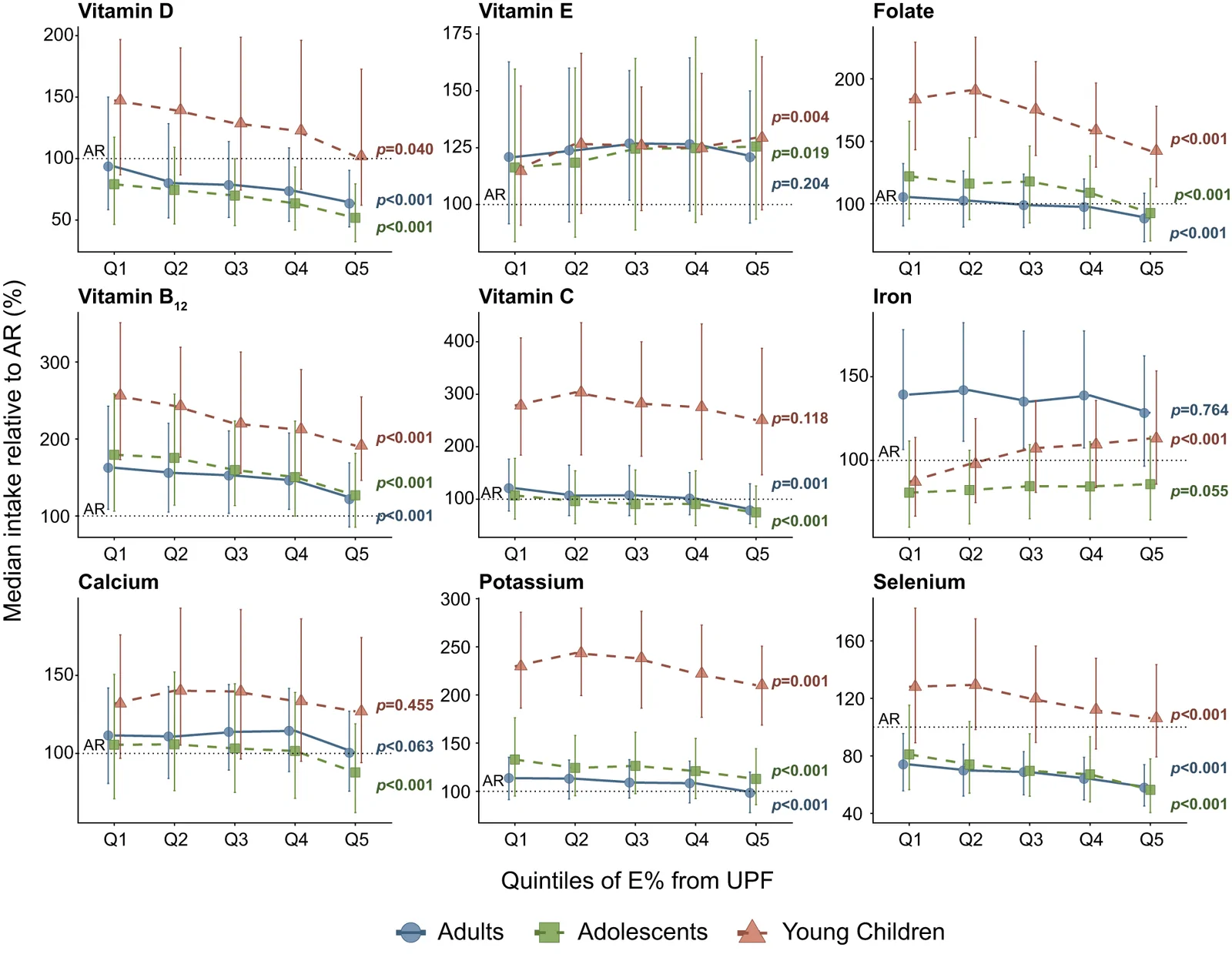
Median (p25, p75) intake of vitamins and minerals by quintiles of E% from UPF per survey. Values are expressed relative to age- and sex-specific average requirements, AR (vitamin D, vitamin C, folate, calcium, iron) or provisional AR (vitamin E, vitamin B_12_, potassium, selenium). *p*-values obtained from multivariable quantile regression at the median (τ = 0.5), modelling UPF intake as a continuous variable (per 10 E% increase), and adjusted for covariates as follows: adults—age, sex, education, leisure-time physical activity and smoking; adolescents—age group, sex, parental education and leisure-time physical activity; young children—age group, sex, parental education, and average daily screen time. *p*-values adjusted for multiple testing using the Benjamini–Hochberg method. Full estimates, with and without energy adjustment, and 95% confidence intervals are provided in Additional file 3. Abbreviations: AR, average requirement; Q, quintile; UPF, ultra-processed foods

Selected indicators of diet quality in relation to E% from UPF are presented in Table 3. Across age strata, a higher dietary share from UPF was associated with reduced diet quality for the majority of variables. Exceptions included no association with SFA among adults and adolescents, decreasing E% from SFA among young children and decreasing dietary density of salt across all age groups. Nutrient density of most included vitamins and minerals decreased with increasing E% from UPF (Table 4). Notable positive associations were observed in young children for iron and vitamin E. Intakes of vitamins and minerals relative to AR or provisional AR are presented in Figure 3 with details in Additional file 3.

**Table 3:** Diet quality indicators by quintiles of energy from ultra-processed foods, with adjusted quantile regression associations

	Quintiles of E% from UPF (range) Median					Per 10 E% higher UPF		*p-*value^‡^
	I	II	III	IV	V	β^†^	95% CI	
**RM–Adults**	**(3.5–33.4)**	**(33.5–41.3)**	**(41.3–48.4)**	**(48.5–56.0)**	**(56.0–98.5)**			
** n =**	**360**	**360**	**359**	**359**	**359**			
** UPF, E%**	**27.6**	**37.6**	**44.7**	**51.9**	**62.0**			
Energy intake, *kcal/day*	1736	1874	1976	2062	1997	72	(46.2, 97.2)	<0.001
Energy density, *kcal/g*	1.31	1.47	1.55	1.63	1.80	0.11	(0.10, 0.13)	<0.001
Protein, *E%*	18.2	16.8	16.2	15.7	14.9	−1.0	(−1.10, −0.81)	<0.001
Dietary fibre, *g/1000 kcal*	11.7	10.3	10.0	9.4	8.6	−0.6	(−0.68, −0.43)	<0.001
Wholegrain, *g/1000 kcal*	25	22	20	18	13	−2.3	(−2.90, −1.73)	<0.001
Saturated fat, *E%*	12.4	13.0	13.3	13.7	13.0	0.1	(−0.05, 0.31)	0.161
Added sugars, *E%*	3.7	5.6	6.5	7.6	9.3	1.5	(1.37, 1.67)	<0.001
Salt (NaCl), *g/1000 kcal*	4.0	3.9	3.9	3.7	3.7	−0.1	(−0.13, −0.05)	<0.001
GEF, *E%*	32.2	25.8	22.9	19.3	15.5	−3.7	(−4.23, −3.08)	<0.001
NRF11.3-S	7.0	6.6	6.2	5.9	5.3	−0.4	(−0.42, −0.32)	<0.001
**RM–Adolescents**	**(0.0–38.3)**	**(38.3–47.7)**	**(47.7–56.1)**	**(56.1–65.3)**	**(65.4–100)**			
** n =**	**620**	**620**	**620**	**620**	**619**			
** UPF, E%**	**31.0**	**43.4**	**52.0**	**60.5**	**72.4**			
Energy intake, *kcal/day*	1795	1939	2061	2091	2099	80	(60.4, 100.0)	<0.001
Energy density, *kcal/g*	1.47	1.64	1.71	1.84	2.00	0.12	(0.11, 0.13)	<0.001
Protein, *E%*	19.1	17.4	16.4	15.4	13.7	−1.2	(−1.31, −1.13)	<0.001
Dietary fibre, *g/1000 kcal*	9.4	8.6	8.3	7.8	7.1	−0.5	(−0.6, −0.41)	<0.001
Wholegrain, *g/1000 kcal*	14.3	13.0	9.5	9.2	5.9	−1.9	(−2.25, −1.46)	<0.001
Saturated fat, *E%*	13.1	14.2	13.8	14.0	13.5	0	(−0.06, 0.15)	0.404
Added sugars, *E%*	4.6	7.4	9.2	11.5	14.3	2.1	(1.95, 2.24)	<0.001
Salt (NaCl), *g/1000 kcal*	4.3	4.1	4.0	3.7	3.4	−0.2	(−0.23, −0.18)	<0.001
GEF, *E%*	18.9	14.2	12.0	9.7	6.8	−2.7	(−2.93, −2.38)	<0.001
NRF11.3-S	5.7	5.4	4.9	4.6	3.9	−0.4	(−0.44, −0.37)	<0.001
**RM–Young Children**	**(5.1–32.2)**	**(32.2–42.3)**	**(42.3–50.4)**	**(50.5–60.0)**	**(60.0–94.0)**			
** n =**	**364**	**364**	**364**	**364**	**364**			
** UPF, E%**	**26.3**	**37.8**	**46.4**	**54.7**	**66.3**			
Energy intake, *kcal/day*	1165	1268	1254	1249	1235	4	(−7.0, 15.8)	0.448
Energy density, *kcal/g*	1.25	1.36	1.41	1.45	1.58	0.07	(0.06, 0.07)	<0.001
Protein, *E%*	15.5	15.1	14.6	14.1	13.4	−0.6	(−0.66, −0.44)	<0.001
Dietary fibre, *g/1000 kcal*	11.7	11.6	10.7	10.3	9.4	−0.5	(−0.64, −0.42)	<0.001
Wholegrain, *g/1000 kcal*	23.5	21.0	21.1	20.4	16.2	−1.4	(−2.02, −0.72)	<0.001
Saturated fat, *E%*	13.9	13.5	13.4	13.3	12.9	−0.3	(−0.41, −0.13)	<0.001
Added sugars, *E%*	2.5	3.9	4.8	5.6	6.6	0.8	(0.66, 0.85)	<0.001
Salt (NaCl), *g/1000 kcal*	3.6	3.7	3.5	3.6	3.3	−0.1	(−0.1, −0.02)	0.005
GEF, *E%*	26.8	23.1	19.2	16.5	12.8	−3.2	(−3.59, −2.77)	<0.001
NRF11.3-S	7.4	7.3	7.1	7.0	6.5	−0.2	(−0.20, −0.12)	<0.001

^†^Estimates from multivariable quantile regression at the median (τ = 0.5); ^‡^*p*-values for the association with UPF intake, modelled continuously (per 10 E% increase), with correction for multiple comparisons across nutrients within each age group using the Benjamini–Hochberg method. Covariates: Adults—age, sex, education, leisure-time physical activity, smoking; Adolescents—age group, sex, parental education, leisure-time physical activity; Young Children—age group, sex, parental education, average daily screen time. Medians are shown for the full samples; regression estimates are based on complete cases. Abbreviations: CI, confidence interval; GEF, generally encouraged food choices; NRF11.3-S, Nutrient Rich Foods index 11.3–Swedish version; RM, Riksmaten (Swedish national dietary survey)

**Table 4:** Density of selected vitamins and minerals by quintiles of energy from ultra-processed foods, adjusted quantile regression

	Quintiles of E% from UPF (range) Median					Per 10 E% higher UPF		*p-*value^‡^
	I	II	III	IV	V	β^†^	95% CI^b^	
**RM–Adults**	**(3.5–33.4)**	**(33.5–41.3)**	**(41.3–48.4)**	**(48.5–56.0)**	**(56.0–98.5)**			
** n =**	**360**	**360**	**359**	**359**	**359**			
** UPF, E%**	**27.6**	**37.6**	**44.7**	**51.9**	**62.0**			
Vitamin D, *µg/1000 kcal*	4.1	3.2	3.0	2.5	2.3	−0.3	(−0.42, −0.25)	<0.001
Vitamin E, *α-TE/1000 kcal*	5.9	5.5	5.4	5.1	5.0	−0.2	(−0.22, −0.10)	<0.001
Folate, *µg/1000 kcal*	153	134	127	119	110	−9	(−10.42, −7.43)	<0.001
Vitamin B12, *µg/1000 kcal*	3.1	2.6	2.5	2.3	1.9	−0.3	(−0.32, −0.22)	<0.001
Vitamin C, *mg/1000 kcal*	57.0	46.1	45.1	41.5	34.0	−3.5	(−4.67, −2.39)	<0.001
Iron, *mg/1000 kcal*	5.7	5.4	5.0	4.8	4.7	−0.2	(−0.31, −0.19)	<0.001
Calcium, *mg/1000 kcal*	472	443	447	428	391	−24	(−30.4, −18.6)	<0.001
Potassium, *mg/1000 kcal*	1847	1676	1593	1488	1378	−112	(−126.5, −98.5)	<0.001
Selenium, *µg/1000 kcal*	27.9	24.3	22.1	19.9	18.5	−2.4	(−2.81, −2.04)	<0.001
**RM–Adolescents**	**(0.0–38.3)**	**(38.3–47.7)**	**(47.7–56.1)**	**(56.1–65.3)**	**(65.4–100)**			
** n =**	**620**	**620**	**620**	**620**	**619**			
** UPF, E%**	**31.0**	**43.4**	**52.0**	**60.5**	**72.4**			
Vitamin D, *µg/1000 kcal*	3.0	2.9	2.6	2.3	1.8	−0.3	(−0.33, −0.26)	<0.001
Vitamin E, *α-TE/1000 kcal*	5.6	5.2	5.2	5.2	5.1	−0.1	(−0.13, −0.01)	0.020
Folate, *µg/1000 kcal*	148	133	123	113	98	−12	(−12.77, −10.49)	<0.001
Vitamin B12, *µg/1000 kcal*	2.7	2.6	2.3	2.1	1.7	−0.2	(−0.26, −0.19)	<0.001
Vitamin C, *mg/1000 kcal*	38.0	32.4	28.9	27.3	25.0	−3.0	(−3.66, −2.32)	<0.001
Iron, *mg/1000 kcal*	4.2	3.9	3.9	3.7	3.5	−0.1	(−0.16, −0.11)	<0.001
Calcium, *mg/1000 kcal*	556	541	503	473	392	−37	(−43.37, −31.57)	<0.001
Potassium, *mg/1000 kcal*	1537	1394	1324	1241	1130	−89	(−97.69, −80.35)	<0.001
Selenium, *µg/1000 kcal*	23.1	20.2	18.6	17.3	14.1	−2.0	(−2.22, −1.79)	<0.001
**RM–Young Children**	**(5.1–32.2)**	**(32.2–42.3)**	**(42.3–50.4)**	**(50.5–60.0)**	**(60.0–94.0)**			
** n =**	**364**	**364**	**364**	**364**	**364**			
** UPF, E%**	**26.3**	**37.8**	**46.4**	**54.7**	**66.3**			
Vitamin D, *µg/1000 kcal*	9.5	7.5	7.1	7.4	5.9	−0.4	(−0.52, −0.20)	<0.001
Vitamin E, *α-TE/1000 kcal*	6.1	6.4	6.2	6.5	6.6	0.1	(0.03, 0.20)	0.012
Folate, *µg/1000 kcal*	150	141	134	126	112	−9	(−10.46, −7.51)	<0.001
Vitamin B12, *µg/1000 kcal*	2.8	2.4	2.2	2.2	2.0	−0.2	(−0.22, −0.15)	<0.001
Vitamin C, *mg/1000 kcal*	55.0	52.5	50.7	53.8	48.4	−0.9	(−2.01, 0.18)	0.106
Iron, *mg/1000 kcal*	4.1	4.2	4.4	4.6	4.9	0.2	(0.18, 0.32)	<0.001
Calcium, *mg/1000 kcal*	553	561	559	544	503	−10	(−17.53, −3.03)	0.007
Potassium, *mg/1000 kcal*	1514	1455	1435	1391	1314	−49	(−61.35, −37.37)	<0.001
Selenium, *µg/1000 kcal*	17.7	17.3	15.6	15.3	14.2	−0.9	(−1.13, −0.67)	<0.001

Estimates from multivariable quantile regression at the median (τ = 0.5); ^‡^ *p*-values for the association with UPF intake, modelled continuously (per 10 E% increase), with correction for multiple comparisons across nutrients within each age group using the Benjamini–Hochberg method. Covariates: Adults—age, sex, education, leisure-time physical activity, smoking; Adolescents—age group, sex, parental education, leisure-time physical activity; Young Children—age group, sex, parental education, average daily screen time. Medians are shown for the full samples; regression estimates are based on complete cases. Abbreviations: CI, confidence interval; RM, Riksmaten (Swedish national dietary survey)

### Association between UPF-intake and weight outcomes

In adults alone, each ten-unit increase in E% UPF was associated with a 0.61% higher BMI (95% CI: 0.04–1.18, *p* = 0.036) in the main model. The association strengthened slightly after adjustment for energy intake and remained virtually unchanged after additional adjustment for diet quality. No significant associations were found between E% UPF and BMI *z*-score among adolescents or 4-year-olds (Additional file 3).

No significant differences in prevalence of overweight/obesity across tertiles of E% from UPF was found in any age stratum (Table 5). However, after energy adjustment, adults in the highest tertile had a 16% higher prevalence of overweight/obesity compared with those in the lowest tertile (PR = 1.16; 95% CI: 1.03–1.30; *p* = 0.018), which persisted after additional adjustment for diet quality. Among adolescents or 4-year-olds, no significant associations were observed in any model.

**Table 5:** Adjusted prevalence ratios for overweight or obesity by tertiles of energy from ultra-processed foods

	Tertiles of dietary contribution from UPF (E%)							
	I (reference)		II			III		
			PR	95% CI	*p*-value	PR	95% CI	*p*-value
**RM–Adults**								
* n (%) with overweight/obesity*	*262*	*(46.7)*	*245*	*(43.9)*		*272*	*(49.3)*	
Model 1^a^			0.95	(0.84, 1.07)	0.408	1.12	(1.00, 1.27)	0.060
Model 2^d^			0.98	(0.86, 1.11)	0.699	1.16	(1.03, 1.30)	0.018
Model 3^e^			0.99	(0.87, 1.12)	0.839	1.18	(1.04, 1.35)	0.010
**RM–Adolescents**								
* n (%) with overweight/obesity*	*201*	*(21.1)*	*201*	*(21.0)*		*186*	*(19.4)*	
Model 1^b^			1.00	(0.84, 1.18)	0.967	0.89	(0.74, 1.06)	0.183
Model 2^d^			1.04	(0.88, 1.24)	0.647	0.94	(0.79, 1.13)	0.524
Model 3^e^			1.00	(0.84, 1.19)	0.969	0.84	(0.70, 1.02)	0.086
**RM–Young Children, 4ys**								
* n (%) with overweight/obesity*	*16*	*(7.3)*	*17*	*(7.7)*		*26*	*(11.6)*	
Model 1^c^			0.88	(0.45, 1.72)	0.716	1.37	(0.74, 2.51)	0.312
Model 2^d^			0.88	(0.45, 1.70)	0.695	1.34	(0.73, 2.47)	0.344
Model 3^e^			0.83	(0.43, 1.60)	0.582	1.18	(0.62, 2.25)	0.611

Estimates obtained from Poisson regression with robust variance. Covariates included in the models

^a^Model 1: age, sex, education, leisure-time physical activity and smoking

^b^Model 1: age group, sex, education and leisure-time physical activity

^c^Model 1: age, sex, education and average daily screentime

^d^Model 1+energy intake

^e^Model 2+diet quality as captured by the NRF11.3-S score

Abbreviations: CI, confidence interval; E%, energy percentage; PR, prevalence ratio; RM, Riksmaten (Swedish national dietary survey); UPF, ultra-processed food

### Sensitivity analyses

The linear associations between UPF intake quintiles and markers of diet quality and nutrient density were largely preserved in the sensitivity analysis using the lower-bound classification scenario for UPF. Notable exceptions were observed among young children, for whom associations with wholegrain, vitamin E, and calcium density were attenuated, while vitamin C density became positively associated with UPF intake. In addition, a significant increase in E% from SFA across quintiles emerged among adolescents (Additional file 3). In contrast, the upper-bound classification scenario produced no substantial changes. In adults, estimates for the linear association between E% UPF and BMI were similar across classification scenarios, although they did not reach statistical significance in the sensitivity analyses. In adolescents, estimates were inconsistent, with a significant negative association observed only under the lower-bound scenario, while no association were observed in young children (Additional file 3).

The exclusion of potential misreporters resulted in similar estimations of E% from UPF and did not alter the effect size or significance level for the association between E% UPF and BMI z-score or overweight/obesity in 4-year-olds. In adults, it resulted in a slightly stronger linear relationship with BMI (β = 0.87%; 95% CI: 0.24–1.50; *p* = 0.007). In adolescents, a positive association between E% UPF and BMI z-score was observed (β = 0.03; 95% CI: 0.00–0.05; *p* = 0.030), along with a prevalence ratio of 1.26 (95% CI: 1.00–1.59; *p* = 0.048) for overweight or obesity in the second compared with the first tertile of UPF intake. Regression models restricted to complete cases yielded estimates consistent with the main findings. Given the low prevalence of overweight and obesity among young children, odds ratios were additionally estimated as a sensitivity analysis; results were similar to those obtained using PR.

## Discussion

This study provides the first comprehensive assessment of UPF consumption in Sweden across adults, adolescents, and young children using data from the most recent national dietary surveys. Under the primary classification scenario, UPF contributed approximately half of total energy intake in all age groups, compared with about one quarter under a stricter scenario, highlighting the importance of explicitly addressing uncertainty in UPF classification. Higher UPF consumption was generally associated with a less favourable nutrient intake, with the exceptions of inconsistent associations with saturated fat, lower salt intake across all age groups, and higher iron and vitamin E intakes in young children. Among adults, a higher dietary share of UPF was weakly associated with higher BMI and prevalence of overweight or obesity, whereas no such associations were observed in adolescents or 4-year-old children.

### Dietary share and classification of UPF

The proportion of energy derived from UPF observed in the middle-bound scenario in the present study is relatively high compared with estimates from other national dietary surveys [12]. Nevertheless, the level was comparable to those reported in Australia (42 E%) [59] and Canada (48 E%) [60], and lower than those observed in the Netherlands (61 E%) [61], the United Kingdom (54 E%) and the United States (56 E%) [5]. UPF intake according to the stricter lower-bound scenario is, however, low compared with estimates from most other high-income countries. Whether classification approaches used in other surveys are more comparable to our middle- or lower-bound scenario is often difficult to assess, underscoring the importance of explicitly describing classification procedures, particularly when dietary assessment methods were not designed to capture UPF intake.

The highest levels of UPF consumption were observed in late adolescence, with lower levels in older age groups, a pattern consistent with previous findings [62], and with the high proportion of discretionary foods previously reported among Swedish adolescents [63]. Studies from the UK and US have similarly reported a higher contribution of sweetened products to UPF intake among women compared with men [6, 64].

Bread has generally been identified as the dominant source of UPF-derived calories, at levels comparable to those in our study, in countries where it is a common staple and assumed to be predominantly industrially produced [6, 13, 65, 66]. In the present study, a substantial share of bread products was classified as ultra-processed owing to the presence of single marker ingredients—such as isolated gluten or syrup—despite an overall favourable nutrient profile, and despite syrup being a common ingredient in Swedish home-baking recipes. This illustrates the nutritional heterogeneity within the UPF fraction of the diet and points to important methodological limitations when classification relies primarily on marker ingredients, and on a global definition that may not fully capture regional variation. Beyond occasionally placing UPF classifications at odds with official dietary guidelines, this approach may arguably risk diluting the core conceptual meaning of UPF as“… *formulations made mostly or entirely from substances derived from foods and additives …”* [41]. Conversely, reliance on ingredient lists may fail to capture foods that have undergone extensive processing resulting in major alterations of texture and physical structure when such changes are not evident from declared ingredients. It should also be noted that, given the vast diversity of food supplies, marker-ingredient-based criteria necessarily involve interpretative choices, thereby complicating the development of a fully standardized and unified classification approach across studies.

In adults, the present study estimated that UPFs contributed 43.9 E% of total energy intake among women and 46.2 E% among men. These estimates were slightly higher than those reported by Mertens et al. [30], who used the same dataset but a very different classification approach and estimated corresponding intakes of 43.8 E% and 40.6 E%, respectively. In that analysis, all composite dishes were coded as UPF, and most breads as non-UPF, whereas each food item was assessed individually in our study. Surprisingly, despite these methodological differences, our overall estimate was similar, highlighting the need for caution when comparing results across studies and for recognizing the limited precision of applying the Nova system to dietary data which was collected at a level of detail not intended to capture processing level. Consequently, comparable UPF shares, even derived from the same data, may in fact represent vastly different underlying dietary compositions, potentially obscuring associations with health outcomes.

Applying the Nova system to dietary intake data can involve considerable uncertainty, both because information on food processing and preparation is often incomplete and because a single food description may correspond to commercially available products with different degrees of processing. To improve the transparency and reproducibility of the classification process, we developed and implemented a systematic protocol incorporating recipe disaggregation, independent classification and explicit decision rules. Rather than relying on a single deterministic classification, potentially ambiguous food items were classified according to alternative scenarios. The resulting range in estimated UPF intake illustrates the uncertainty that may arise when operationalizing the Nova system using dietary survey data, while providing a transparent assessment of its potential influence on the study findings. Under the stricter lower-bound scenario dominant food groups such as breads and composite dishes were largely coded as non-UPF. Interestingly, this did not strengthen observed associations, suggesting that the relationships observed were primarily driven by foods consistently and unambiguously classified as UPF. Explicitly considering classification uncertainty in this way may improve the transparency and reproducibility of future epidemiological studies applying the Nova system.

### Diet quality in relation to UPF intake

The decline in overall diet quality with increasing dietary share from UPF observed in our study aligns with previous findings [12, 66, 67]. Added sugars showed the strongest association with UPF intake, and together with the concomitant decline in wholegrain intake and dietary fibre, this points to a particularly pronounced impact on overall carbohydrate quality. Although the E% of added sugars increased markedly, average intakes among adults and young children remained within recommended limits in all quintiles of UPF-intake.

In line with studies from other countries with significant dairy consumption [13, 60, 68, 69], the intake of SFA was above recommended limits across all UPF quintiles. However, in the present study, UPF intake was not associated with SFA intake among adults and adolescents. The inverse association observed in young children may reflect higher consumption of fortified fruit- and cereal-based children’s foods, which are both low in SFA and classified as UPF. This pattern likely also contributes to age-related divergencies in overall nutrient density under the lower-bound scenario. Contrary to expectations, an inverse association between salt and UPF intake was observed. This finding should be interpreted cautiously, as salt intake from homemade dishes was estimated using standard recipes. The result may also be influenced by the relatively large contribution of bread to the UPF fraction of the diet. The increase in iron intake with higher UPF consumption among young children is noteworthy and suggests that certain UPF may facilitate achievement of recommended intakes for micronutrients of concern in specific population groups, as observed in previous studies [70], although this potential benefit should be interpreted in the context of overall diet quality.

The modest proportion of food items meeting both the criteria for UPF and GEF indicates that most UPF are nutritionally distinct from foods promoted as healthier choices under current dietary guidelines. This interpretation is further supported by the clear inverse relationship between intake of GEF and UPF across all age groups. At the same time, the non-negligible overlap demonstrates that UPF classification does not fully correspond to nutrient criteria underpinning these guidelines, reflecting heterogeneity within the UPF category as well as the conceptual differences between processing-based and nutrient-based classification systems.

### Association between UPF intake and weight outcomes

The previously observed relationship between UPF intake and excess weight likely involves multiple mechanisms, including high energy density, soft texture, palatability, convenience and marketing appeal [71]. Cross-sectional studies report mostly positive associations between UPF intake and BMI or excess weight in adults [6, 59, 64], but weaker or absent associations in children [23]. While the magnitude of the association among adults in the present study is unlikely to be clinically meaningful, the observed direction is consistent with experimental and longitudinal evidence suggesting that high UPF intake may contribute to excess energy intake and weight gain over time. The cross-sectional design of the current study precludes causal inference, and reverse causality or selective underreporting among individuals with excess weight cannot be excluded. In this context, the stronger associations observed in adults and adolescents in sensitivity analyses excluding suspected misreporters suggest that such reporting biases may have attenuated associations in the main analyses. Potential differences in the composition of the UPF fraction of the diet between populations warrant consideration when comparing findings across studies. Although estimated UPF intake varied substantially across the alternative classification scenarios in our study, the overall pattern of associations with weight outcomes remained broadly similar, suggesting that classification uncertainty is unlikely to explain the observed results. The low prevalence of overweight or obesity among young children further reduced power to detect associations.

### Strengths and limitations

Key strengths of the current study include the use of high-quality dietary assessment methods, rigorous food classification with recipe disaggregation, and a systematic classification protocol incorporating alternative classification scenarios to explicitly address uncertainty in UPF categorization, supported by independent classification, assessment of inter-rater reliability, and extensive sensitivity analyses.

Nevertheless, some limitations warrant consideration. Misclassification of UPF cannot be ruled out, as brand-level information and preparation details were incomplete. In addition, the web-based dietary assessment tools included many generic food items, particularly for adolescents and young children, limiting the level of detail available for classification and necessitating reliance on assumptions. Reformulation of products over time may also introduce bias. Dietary intake was self-reported and therefore subject to established measurement error, including recall bias and behavioural reactivity. In addition to general energy misreporting, foods perceived as unhealthy may have been selectively underreported due to social desirability bias, which could have influenced the estimated associations between UPF intake and the study outcomes. Although the surveys were nationally representative, data collected in 2010–2011 (adults) and 2016–2017 (adolescents) may not fully reflect current consumption patterns.

Low participation rates, particularly among adults and young children, together with the absence of sampling weights, may have reduced the representativeness of the analytical samples, potentially limiting the generalizability of the findings. This was particularly evident with regard to the higher educational attainment among participants compared with the target population [33]. As higher educational attainment is generally associated with lower prevalence of overweight and obesity in Sweden [11], estimates of overweight and obesity prevalence may therefore be somewhat conservative. The potential direction of any bias in estimated UPF intake is less clear, given the inconsistent associations between educational attainment and UPF intake reported across nationally representative studies [72]. Finally, a higher proportion of weekend days among young children may have modestly inflated UPF estimates in this group, although total discretionary food intake remained low.

## Conclusions

In conclusion, UPF contributed substantially to energy intake in all age groups, though estimates differed markedly across classification scenarios, reflecting underlying uncertainty. Higher UPF intake was associated with less favourable diet quality across most indicators, but not consistently with SFA. In contrast, higher intake of UPF was also associated with lower intake of salt in all age groups, and higher intake of iron and vitamin E in young children. No robust associations were identified between UPF intake and BMI, BMI *z*-score or prevalence of overweight and obesity. These findings highlight the methodological challenges of applying the Nova classification to dietary assessment data and the importance of having a systematic and transparent approach to handling classification uncertainty. Future studies would benefit from dietary assessment tools that better capture food processing and from continued refinement of transparent classification protocols.

## Electronic supplementary material

Below is the link to the electronic supplementary material.

Supplementary Material 1:Protocol for classification of ultra-processed foods.

Supplementary Material 2:Food items list. List of unique food items reported in the three national dietary surveys, with corresponding classifications.

Supplementary Material 3: The file contains all supplements referred to in the main text, with exception of the classification protocol and food items list presented separately.

## Data Availability

Annotated analytic code sufficient to reproduce the main analyses is available in the GitHub repository TorstenS77/Riksmaten_UPF, [https://github.com/TorstenS77/Riksmaten_UPF]. Raw data for RM–Adults and RM–Adolescents are publicly available from the Swedish Food Agency, [https://www.livsmedelsverket.se/om-oss/psidata/apiMatvanor/]. Raw data for RM–Young Children are available from the Swedish Food Agency upon reasonable request and subject to approval.

## Abbreviations

AR: Average requirement
CI: Confidence interval
E%: Energy percentage
ED: Energy density
EI: Energy intake
GEF: Generally encouraged food choices
IQR: Interquartile range (p25-p75)
MRI: Maximum recommended intake
NNR: Nordic Nutrition Recommendations
NRF: Nutrient Rich Foods index
PR: Prevalence ratio
RI: Recommended intake
RM: Riksmaten (Swedish national dietary survey)
SD: Standard deviation
SFA: Saturated fatty acids
UPF: Ultra-processed food

## Declarations

### Declaration on the use of generative AI

During the preparation of this work, Chat-GPT-5 was used to assist in developing code for statistical analyses and to improve the structure and readability of the text. The first author takes full responsibility for the content of the publication.
