# Supplementary material for: Intake of ultra-processed foods in Sweden and its association with diet quality and weight status: cross-sectional analyses from three national dietary surveys
Source: Nutr J. 2026 Oct 13;25:110. doi: 10.1186/s12937-026-01408-5 (PMC13663064; doi:10.1186/s12937-026-01408-5)
Supplement: Supplementary file 1 [file 12937_2026_1408_MOESM1_ESM.pdf]

Supplement S1. Protocol for the classification of ultra-processed food

Step 1. Food items are classified into three categories (numbered 0, 1, or 2) by two independent researchers:

0	Non-UPF	• Nova Group 1, 2 and 3 or in combination.<br>• Composite dishes, baked goods and other multi-ingredient items that are identified as culinary preparations made predominantly from Nova Group 1, 2 and 3-ingredients in a non-industrial setting.<br>• Industrially manufactured composite dishes, baked goods and other multi-ingredient items that do not contain any markers of UPF.
1	UPF	• Nova Group 4, identified by the "operational classification criteria" proposed by Monteiro and colleagues[1] based on the presence of marker ingredients as summarized in table 1 below. For further guidelines, refer to Martinez-Steele and colleagues[2].<br>• Composite dishes, baked goods and other multi-ingredient items that are identified as industrially manufactured and contain at least one marker of UPF are classified entirely as UPF.
2	To be disaggregated	• Composite dishes, baked goods and other multi-ingredient items that are identified as culinary preparations as above but additionally contain ingredients classified as UPF to some extent.

Step 2. Flag uncertain food items that will need to be discussed in subsequent steps, as well as uncertain food items that will have to be subject to sensitivity analysis using alternative classification scenarios.

Step 3. The classifications of the two researchers are compared. Cohen's weighted Kappa is calculated to assess inter-rater reliability. Discrepancies and uncertainties are resolved by discussion. A senior researcher is involved if needed. In case of uncertainty, a conservative approach is preferred for the main analysis meaning that the lower degree of processing is assumed.

Step 4. Food items assigned to group 2 are disaggregated by one researcher. Use recipes and ingredients from the Swedish Food Composition Database if available. If else, use recipes from "Vår Kokbok" cookbook or popular online recipes.

---

[1] Monteiro, C.A. *et al.* (2019) 'Ultra-processed foods: what they are and how to identify them', *Public health nutrition*, 22(5), pp. 936–941.

[2] Martinez-Steele, E., Khandpur, N., Batis, C. et al. (2023) 'Best practices for applying the Nova food classification system'. *Nat Food* 4, 445–448.

Step 5. Ingredients in the disaggregated recipes are classified as in step 1 into categories 0 or 1.

Step 6. Categorize items flagged for sensitivity analysis into alternative categories based on different scenarios for processing level:

Lower bound	Middle bound	Upper bound
Assuming the lowest, realistic level of processing.	Assuming the most likely level of processing. In cases of uncertainty, the more conservative option is preferred. A 50/50 split can be appropriate in select cases.	Assuming the highest, realistic level of processing.

Figure 1. Flowchart for categorizing composite dishes and multi-ingredient food items.

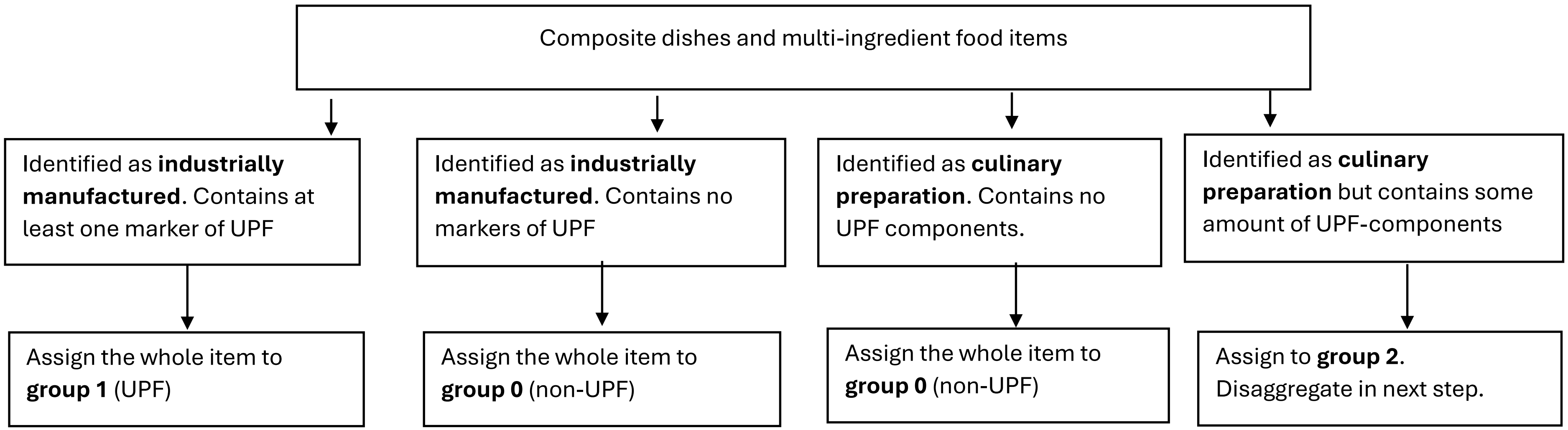

Table 1. Marker ingredients of ultra-processed foods

Additives with cosmetic function	Aromas and extracts[a]	Ingredients of no or rare culinary use. Isolated fractions or highly processed forms protein, carbohydrates or fats. List not exhaustive.		
Functional classes		Protein	Carbohydrates	Fats
Colours<br>Carbonating agents<br>Emulsifiers<br>Thickeners<br>Gelling agents<br>Bulking agents.<br>Flavours<br>Flavour enhancers<br>Sweeteners<br>Glazing agents<br>Foaming- and antifoaming agents.<br><br>Stabilisers[a]<br>Nitrates & nitrites (typically listed as preservatives) [a]	Aromas, natural or synthetic.<br>Extracts, e.g. from smoke, malt, yeast, herbs and spices.	Hydrolysed proteins<br>Vegetable protein isolates<br>Gluten<br>Casein<br>Whey protein<br>Gelatine<br>“Mechanically separated meat”	All syrups (excluding 100% maple syrup and molasses).<br>Fructose<br>Dextrose, glucose<br>Lactose<br>“High-fructose corn syrup”<br>“Fruit juice concentrate”<br>Invert sugar<br>Maltodextrin<br>Soluble or insoluble fibre in isolated form.<br>Modified starches	Modified oils, e.g:<br>Interesterified oil<br>Hydrogenated oil

[a]Subject to study-specific decision (see below)

Decisions

Below is a list of study-specific decisions made during the classification process. These decisions were informed by the current formulation of the Nova framework and its foundational literature, with the objective to operationalise these principles as faithfully as possible. However, certain decisions required interpretation and may therefore differ from those adopted in other studies.

A
- Food additives can have more than one functional property. They are considered markers of UPF if they can belong to one of the "cosmetic" functional classes according to *Codex Alimentarius* and it's reasonable to believe that they at least partly have this function in the food item of interest. Refer to the online search function in the link to see a list of possible functions for each additive: https://www.fao.org/gsfaonline/additives/results.html

B
- Some composite dishes may be impossible to identify as "industrial products" or "culinary preparations" from the information in the dataset. E.g. "Pizza from restaurant" could come from a pizza chain outlet selling industrial products, or from a small scale, artisanal pizzeria where dishes are freshly prepared from basic ingredients.

- In uncertain cases, the pragmatic approach is to assign the item to the most likely alternative and flag for sensitivity analysis. The most likely alternative could be informed e.g. by market research, knowledge about local food habits or the locations where the food was reportedly consumed.

- In select cases, the most appropriate solution may be to split the item 50/50 in the middle-bound analysis. This approach was most notably applied to pizzas and minced meat preparations (meatballs and patties) when information suggesting level of processing was absent.

C
- Examples of food outlets that are assumed to serve culinary preparations (as opposed to industrial products) include full-service restaurants or artisanal bakeries and cafés where food items are prepared in-house from scratch using predominantly Nova group 1, 2 and 3 ingredients.

D
- Certain processed foods can be either UPF or Nova group 3 depending on their ingredient lists. Search for the product at two major online grocery retailers (ica.se and coop.se), filter by popularity. Consider the most popular items from each retailer, scan the ingredient lists and classify them as UPF or non-UPF based on this.

E
- Cooking fat used in the recipes of composite dishes may be UPF (solid or liquid margarin) or non-UPF (butter, oils). In disaggregated recipes, the proportion of cooking fat classified as UPF was based on questionnaire data in which participants reported the type of cooking fat predominantly used in their household. The proportion reporting predominant use of solid or liquid margarin was 43% in RM-Adults, 36% in RM-Adolescents and 20% in RM-Young Children.

  - Solid fats in recipes for home-made fine bakery wares are generally assumed to be butter.
  - When cream is used in culinary preparations, we assume it to be full fat "heavy cream"/"whipping cream" unless specified otherwise.

For single food-items reported as “pan-fried” or “creamed” the amount of fat in the finished product is estimated based on similar items in the Swedish food composition database. Unbreaded, whole cuts of fish and meat are estimated to contain no added fat in their cooked form.

F
- For composite dishes and mixed meals served and consumed in institutional settings (e.g. school canteen, hospital, nursing homes and similar) a high level of processing is generally assumed in the middle-bound analysis. They are usually reported as main components of the meal in the survey and evaluated at this level. Refer to product information from major wholesale food grocers (e.g. menigo.se, martinservera.se).

G
- Substances listed as “aromas” or “extracts” within the European Union are considered markers of UPF when added for flavour- or colouring purposes. Extracts warrant special consideration, as extraction *per se* can be a traditional processing method and is indeed mentioned among the methods used to obtain Nova G2 foods. However, the extracts commonly used for industrial applications - such as from barley-malt, yeast, smoke, herbs and spices - are more comparable in nature to that of other flavour additives considered as marker ingredients for UPF.

H
- Stabilisers are not specifically mentioned among the additives with cosmetic function in original Nova literature. However, additives labelled as stabilisers often serve multiple purposes, and can in practice be hard to distinguish from e.g. thickeners or emulsifiers. Stabilisers were therefore considered markers of ultra-processing.

I
- Nitrites and nitrates were classified as UPF markers, although commonly listed as preservatives on food labels. While they have long been used to ensure microbiological safety and retain colour, they are considered to influence the sensory properties of foods beyond the preservation of their natural characteristics.

J
- Plant based milk substitutes can be classified as either UPF or non-UPF based on the inclusion of cosmetic additives or other markers of UPF such as fiber isolates or protein isolates. For this application, potassium phosphates were considered as UPF markers. Although they are typically labelled as “acidity regulators”, they may also function as emulsifiers, stabilizers or thickeners.
- Tofu is considered non-UPF irrespective of the curdling-agent used (unless containing other markers of UPF).

K
- Calcium chloride in cheese is not considered a cosmetic additive. It is frequently used to adjust the calcium content of the milk prior to curdling, to optimize consistency and yield.

L
- Children’s food, including: Canned, mixed dishes with modified texture, breast-milk substitute or follow-on formulas, ready to eat smoothies or porridges sold in pouches (“klämmis”) and gruel (“välling”):
  - Are generally assumed to be industrially produced if not explicitly “home-made”.
  - Same rules apply as for adult foods regarding markers of UPF.

M
- Powdered plain milk is not considered UPF, irrespective of fat content. However, isolated constituents of milk (such as whey-powder or isolated lactose) are markers of UPF.
