# Supplementary material for: Intake of ultra-processed foods in Sweden and its association with diet quality and weight status: cross-sectional analyses from three national dietary surveys
Source: Nutr J. 2026 Oct 13;25:110. doi: 10.1186/s12937-026-01408-5 (PMC13663064; doi:10.1186/s12937-026-01408-5)
Supplement: Supplementary file 2 [file 12937_2026_1408_MOESM2_ESM.pdf]

Food-ID	Swedish name	English name	Proportion of UPF by E% Lower	Middle-bound Young children	Middle-bound Adolescents	Middle-bound Adults	Upper	GEF (x=not classified)	Foodgroup	Beverage (yes/no)
3	Ister gris	Pork lard	0.00	0.00	0.00	0.00	0.00	no	Fat	no
4	Kokosfett	Coconut fat	0.00	1.00	1.00	1.00	1.00	no	Fat	no
6	Matfettsblandning fett 60% berikad typ Bregott mellan	Fat spread blend, 60% fat, enriched (Bregott Mellan type)	0.00	0.00	0.00	0.00	0.00	no	Fat	no
7	Bordsmargarin fett 60% berikad typ Milda	Margarine spread, 60% fat, fortified (Milda type)	1.00	1.00	1.00	1.00	1.00	yes	Fat	no
8	Bordsmargarin extrasaltat fett 60% berikad typ Milda	Margarine spread, extra salted, 60% fat, fortified (Milda type)	1.00	1.00	1.00	1.00	1.00	no	Fat	no
10	Flytande margarin fett 82% berikad typ Milda culinesse	Liquid margarin fat 82% enriched	1.00	1.00	1.00	1.00	1.00	no	Fat	no
13	Hushållsmargarin fett 80% berikad typ Milda	Household margarine, 80% fat, fortified (e.g. Milda)	1.00	1.00	1.00	1.00	1.00	no	Fat	no
17	Lättmargarin fett 38% berikad typ Becel	Light margarine, 38% fat, fortified (e.g., Becel)	1.00	1.00	1.00	1.00	1.00	no	Fat	no
18	Lättmargarin m växtsterol 7,5% fett 35% berik typ Becel proactiv	Light margarine with plant sterols, 7.5% sterol, 35% fat, fortified (e.g., Becel ProActiv)	1.00	1.00	1.00	1.00	1.00	yes	Fat	no
20	Lättmargarin fett 40% berikad typ Lätt & lagom	Light margarine, 40% fat, fortified (e.g., Lätt & Lagom)	1.00	1.00	1.00	1.00	1.00	no	Fat	no
21	Majonnäs fett 90%	Mayonnaise, 90% fat	0.00	1.00	1.00	1.00	1.00	no	Savoury sauces and condiments	no
23	Gravlaxsås hovmästarsås	Gravlax sauce (Swedish mustard-dill sauce)	0.00	1.00	1.00	1.00	1.00	no	Savoury sauces and condiments	no
25	Remouladsås	Remoulade sauce	0.00	1.00	1.00	1.00	1.00	no	Savoury sauces and condiments	no
26	Vinägrett dressing fett 45%	Vinaigrette dressing, 45% fat	0.00	0.00	0.00	0.00	0.00	no	Savoury sauces and condiments	no
28	Lättmargarin fett 40% berikad typ Lätt & lagom omega 3	Light margarine, 40% fat, fortified (e.g., Lätt & Lagom Omega 3)	1.00	1.00	1.00	1.00	1.00	no	Fat	no
29	Smör fett 80 %	Butter, 80% fat	0.00	0.00	0.00	0.00	0.00	no	Fat	no
30	Smör extrasaltat fett 80%	Butter, extra salted (80% fat)	0.00	0.00	0.00	0.00	0.00	no	Fat	no
31	Smör osaltat fett 80%	Butter, unsalted (80% fat)	0.00	0.00	0.00	0.00	0.00	no	Fat	no
32	Matolja berik	Cooking oil, fortified	0.00	0.00	0.00	0.00	0.00	yes	Fat	no
34	Majsolja	Corn oil	0.00	0.00	0.00	0.00	0.00	yes	Fat	no
35	Olivolja	Olive oil	0.00	0.00	0.00	0.00	0.00	yes	Fat	no
38	Sesamolja	Sesame oil	0.00	0.00	0.00	0.00	0.00	yes	Fat	no
40	Solrosolja	Sunflower oil	0.00	0.00	0.00	0.00	0.00	yes	Fat	no
47	Dressing olja fett ca 25%	Dressing, oil-based, approx. 25% fat	0.00	1.00	1.00	1.00	1.00	no	Savoury sauces and condiments	no
50	Majonnäs fett 80%	Mayonnaise, 80% fat	0.00	1.00	1.00	1.00	1.00	no	Savoury sauces and condiments	no
51	Gurkmajonnäs el gurksallad gatukök	Cucumber mayonnaise or cucumber salad (fast food style)	1.00	1.00	1.00	1.00	1.00	no	Savoury sauces and condiments	no
52	Räkmajonnäs el räksallad gatukök	Shrimp mayonnaise or shrimp salad (fast food style)	1.00	1.00	1.00	1.00	1.00	no	Savoury sauces and condiments	no
53	Majonnäs lätt fett 30%	Mayonnaise, light, 30% fat	1.00	1.00	1.00	1.00	1.00	no	Savoury sauces and condiments	no
54	Grönsaksmajonnäs	Vegetable mayonnaise	1.00	1.00	1.00	1.00	1.00	no	Savoury sauces and condiments	no
55	Mimosasallad	Mimosa salad	0.00	1.00	1.00	1.00	1.00	no	Savoury sauces and condiments	no
56	Rödbetssallad m. gräddfil	Beetroot salad, with sour cream	0.00	0.80	0.80	0.80	1.00	no	Savoury sauces and condiments	no
58	Bearnaisesås	Béarnaise sauce	0.00	1.00	1.00	1.00	1.00	no	Savoury sauces and condiments	no
60	Rhode Island sås	Rhode Island sauce	0.06	1.00	1.00	1.00	1.00	no	Savoury sauces and condiments	no
63	Bearnaisesås fryst el pulver tillagad	Béarnaise sauce, frozen or powdered, prepared	1.00	1.00	1.00	1.00	1.00	no	Savoury sauces and condiments	no
65	Mesost fett 17% berik typ Fjällbrynt	Whey cheese, 17% fat, enriched (Fjällbrynt type)	0.00	0.00	0.00	0.00	0.00	no	Cheese	no
66	Mesost fett ca 30% berik	Whey cheese, approx. 30% fat, enriched	0.00	0.00	0.00	0.00	0.00	no	Cheese	no
67	Mesost lätt fett 9% berik	Whey cheese, light, 9% fat, fortified	1.00	1.00	1.00	1.00	1.00	no	Cheese	no
68	Messmör fett 5% berik	Whey butter, 5% fat, fortified	1.00	1.00	1.00	1.00	1.00	no	Cheese	no
70	Keso färskost fett 4%	Cottage cheese, 4% fat	0.00	0.00	0.00	0.00	0.00	yes	Cheese	no
71	Keso färskost ananas apelsin fett 3%	Cottage cheese, pineapple and orange, 3% fat	1.00	1.00	1.00	1.00	1.00	no	Cheese	no
72	Keso färskost chili pesto lök feta fett 3,5-5%	Cottage cheese, chili, pesto, onion, feta, 3.5–5% fat	1.00	1.00	1.00	1.00	1.00	no	Cheese	no
75	Kvarg färskost fett 1% Kesella lätt	Quark, cottage cheese, 1% fat (Kesella light)	0.00	0.00	0.00	0.00	0.00	yes	Cheese	no
76	Kvarg färskost fett 10% Kesella	Quark, fresh cheese, 10% fat (Kesella)	0.00	0.00	0.00	0.00	0.00	no	Cheese	no
77	Ost hårdost fett 10%	Cheese, hard, 10% fat	0.00	0.00	0.00	0.00	0.00	yes	Cheese	no
78	Ost hårdost fett 23%	Cheese, hard, 23% fat	0.00	0.00	0.00	0.00	0.00	no	Cheese	no
80	Ost hårdost fett 38%	Cheese, hard, 38% fat	0.00	0.00	0.00	0.00	0.00	no	Cheese	no
86	Mjukost smältost fett ca 16%	Soft cheese, processed, approx. 16% fat	1.00	1.00	1.00	1.00	1.00	no	Cheese	no
87	Mjukost smältost fett 4%	Soft cheese, processed, 4% fat	1.00	1.00	1.00	1.00	1.00	no	Cheese	no
88	Margarinost fett 16%	Margarine cheese, 16% fat	1.00	1.00	1.00	1.00	1.00	no	Meat and dairy imitations	no
89	Margarinost fett 30%	Margarine cheese, 30% fat	1.00	1.00	1.00	1.00	1.00	no	Meat and dairy imitations	no
94	Salladsost fett 22%	Salad cheese, 22% fat	0.00	0.00	0.00	0.00	0.00	no	Cheese	no
95	Ost hårdost fett 17%	Cheese, hard, 17% fat	0.00	0.00	0.00	0.00	0.00	yes	Cheese	no
96	Ost hårdost fett 28%	Cheese, hard, 28% fat	0.00	0.00	0.00	0.00	0.00	no	Cheese	no
97	Ost hårdost fett 31%	Cheese, hard, 31% fat	0.00	0.00	0.00	0.00	0.00	no	Cheese	no
99	Fetaost fett 25%	Feta cheese, 25% fat	0.00	0.00	0.00	0.00	0.00	no	Cheese	no

100	Halloumiost fett 22% ej stekt	Halloumi cheese fat 22% not panfried	0.00	0.00	0.00	0.00	0.00	no	Cheese	no
103	Parmesanost hårdost fett 30%	Parmesan cheese, hard cheese fat 30%	0.00	0.00	0.00	0.00	0.00	no	Cheese	no
105	Camembert vitmögelost lätt fett 11%	Camembert, white rind cheese, light fat 11%	0.00	0.00	0.00	0.00	0.00	yes	Cheese	no
107	Färskost fett 27% typ Philadelphia	Cream cheese fat 27%, such as Philadelphia	0.00	1.00	1.00	1.00	1.00	no	Cheese	no
108	Färskost cream cheese lätt fett 15%	Cream cheese light fat 15%	1.00	1.00	1.00	1.00	1.00	no	Cheese	no
109	Färskost extra light fett 5% typ Philadelphia	Cream cheese extra light 5% such as Philadelphia	1.00	1.00	1.00	1.00	1.00	no	Cheese	no
110	Camembert vitmögelost friterad	Camembert, white rind cheese, deep fried	0.00	1.00	1.00	1.00	1.00	no	Cheese	no
111	Getost chèvre vitmögelost fett 25%	Goats cheese chèvre, white rind cheese fat 25%	0.00	0.00	0.00	0.00	0.00	no	Cheese	no
112	Getmjölk	Goat milk	0.00	0.00	0.00	0.00	0.00	no	Cheese	yes
113	Bröstmjölk humanmjölk	Breast milk (human milk)	0.00	0.00	0.00	0.00	0.00	x	Milk, yoghurt and similar	yes
114	Filmjölk fett 3%	Fermented milk, 3% fat	0.00	0.00	0.00	0.00	0.00	no	Milk, yoghurt and similar	no
116	Kondenserad mjölk konserv konc sockrad fett ca	Condensed milk, canned, sweetened, approx. fat	0.00	1.00	1.00	1.00	1.00	no	Milk, yoghurt and similar	no
118	Mjölkpulver fett 1%	Powdered milk, fat 1%	0.00	0.00	0.00	0.00	0.00	x	Milk, yoghurt and similar	no
119	Långfil fett 3%	Fermented milk (local specialty), fat 3%	0.00	0.00	0.00	0.00	0.00	no	Milk, yoghurt and similar	no
120	Lättfil fett 0,5%	Fermented milk, light 0.5%	0.00	0.00	0.00	0.00	0.00	yes	Milk, yoghurt and similar	no
121	Lättmjölk fett 0,5% berik A- och D-vitamin	Low-fat milk, 0.5% fat, fortified with vitamins A and D	0.00	0.00	0.00	0.00	0.00	yes	Milk, yoghurt and similar	yes
122	Milkshake choklad jordgubb	Milkshake, chocolate or strawberry	0.72	1.00	1.00	1.00	1.00	no	Dairy based desserts	yes
123	Mjölk fett 3%	Milk, 3% fat	0.00	0.00	0.00	0.00	0.00	no	Milk, yoghurt and similar	yes
124	Yoghurt naturell fett 3 %	Plain yoghurt, 3% fat	0.00	0.00	0.00	0.00	0.00	no	Milk, yoghurt and similar	no
126	Fruktyoghurt lätt fett 0,5%	Fruit yoghurt, low-fat, 0.5% fat	1.00	1.00	1.00	1.00	1.00	no	Milk, yoghurt and similar	no
127	Mjölk fett 4,2% typ lantmjölk	Milk, 4.2% fat (farm-style milk)	0.00	0.00	0.00	0.00	0.00	no	Milk, yoghurt and similar	yes
128	Filmjölk A-fil fett 3% berikad	Fermented milk (local specialty), fat 3%, fortified	0.00	0.00	0.00	0.00	0.00	no	Milk, yoghurt and similar	no
129	Mellanmjölk fett 1,5% berik A- och D-vitamin	Semi-skimmed milk, 1.5% fat, fortified with vitamins A and D	0.00	0.00	0.00	0.00	0.00	no	Milk, yoghurt and similar	yes
130	Mellanfil fett 1,5%	Semi-skimmed fermented milk, 1.5% fat, fortified with vitamins A and D	0.00	0.00	0.00	0.00	0.00	yes	Milk, yoghurt and similar	no
131	Minimjölk fett < 0,1% berik	Skimmed milk, <0.1% fat, fortified	0.00	0.00	0.00	0.00	0.00	yes	Milk, yoghurt and similar	yes
133	Mjölkdryck jordgubb fett 1,5% berik	Strawberry milk drink, 1.5% fat, fortified	1.00	1.00	1.00	1.00	1.00	no	Milk, yoghurt and similar	yes
135	Lättyoghurt naturell fett 0,5%	Low-fat plain yoghurt, 0.5% fat	0.00	0.00	0.00	0.00	1.00	yes	Milk, yoghurt and similar	no
136	Fruktyoghurt fett 2%	Fruit yoghurt, 2% fat	1.00	1.00	1.00	1.00	1.00	no	Milk, yoghurt and similar	no
137	Delikatessyoghurt fett 7 %	Gourmet yoghurt, 7% fat	1.00	1.00	1.00	1.00	1.00	no	Milk, yoghurt and similar	no
138	Fruktyoghurt lätt fett < 0,05%	Fruit yoghurt, low-fat < 0.05%	1.00	1.00	1.00	1.00	1.00	no	Milk, yoghurt and similar	no
144	Filmjölk m jordgubbssmak fett 2,7%	Sour milk with strawberry flavor, 2.7% fat	1.00	1.00	1.00	1.00	1.00	no	Milk, yoghurt and similar	no
145	Lättfil m jordgubbsmak 0,5% fett	Low-fat sour milk with strawberry flavor, 0.5% fat	1.00	1.00	1.00	1.00	1.00	no	Milk, yoghurt and similar	no
147	Mild lättyoghurt vanilj 0,5% fett	Mild low-fat vanilla yoghurt, 0.5% fat	1.00	1.00	1.00	1.00	1.00	no	Milk, yoghurt and similar	no
148	Mild yoghurt m vanilj 2% fett	Mild vanilla yoghurt, 2% fat	1.00	1.00	1.00	1.00	1.00	no	Milk, yoghurt and similar	no
156	Chokladdryck standardmjölk	Chocolate drink with standard milk	0.00	0.48	0.48	0.48	0.48	no	Milk, yoghurt and similar	yes
157	Varm choklad mjölk fett 1,5%	Hot chocolate with milk, 1.5% fat	0.00	0.55	0.55	0.55	0.55	no	Milk, yoghurt and similar	yes
158	Chokladdryck lättmjölk	Chocolate drink with low-fat milk	0.00	0.59	0.59	0.59	0.59	no	Milk, yoghurt and similar	yes
160	Smoothie m bär yoghurt banan juice	Smoothie with berries, yoghurt, banana, and juice	0.00	0.00	0.00	0.00	0.00	x	Milk, yoghurt and similar	yes
161	Matlagningsyoghurt fett 8%	Cooking yoghurt, 8% fat	0.00	0.00	0.00	0.00	1.00	no	Milk, yoghurt and similar	no
163	Hårt bröd fullkorn råg fibrer ca 18% Ryvita mörkt	Crispbread, whole grain rye, approx. 18% fiber (Ryvita dark)	0.00	0.00	0.00	0.00	0.00	yes	Bread	no
166	Hårt bröd fullkorn råg fibrer 16% Crisp o Finn crisp	Crispbread, whole grain rye, 16% fiber (Crisp or Finn Crisp)	0.00	0.00	0.00	0.00	0.00	no	Bread	no
167	Hårt bröd fullkorn råg fibrer ca 15% Kavli flatbröd	Crispbread, whole grain rye, approx. 15% fiber (Kavli flatbread)	0.00	0.00	0.00	0.00	0.00	yes	Bread	no
175	Hårt bröd fullkorn råg fibrer 15,5% Wasa delikatess	Crispbread, whole grain rye, 15.5% fiber (Wasa Delikatess)	0.00	0.00	0.00	0.00	0.00	yes	Bread	no
178	Hårt bröd fullkorn havre vete råg mjölk fibrer 8% Wasa havre	Crispbread with whole grain oats, wheat, rye, milk, 8% fiber (Wasa oat)	1.00	1.00	1.00	1.00	1.00	yes	Bread	no
179	Hårt bröd vete fibrer ca 5% Wasa vete	Crispbread, wheat, approx. 5% fiber (Wasa wheat)	0.00	0.00	0.00	0.00	0.00	no	Bread	no
183	Hårt bröd fullkorn vete havre socker kli örtkryddor fibrer 10,5% Wasa knecks	Crispbread with whole grain wheat, oats, sugar, bran, herbs, 10.5% fiber (Wasa Knecks)	0.00	0.00	0.00	0.00	1.00	no	Bread	no
184	Grahamsbröd fullkorn fibrer ca 6%	Graham bread, whole grain, approx. 6% fiber	0.00	1.00	1.00	1.00	1.00	no	Bread	no
185	Bröd fullkorn korn fibrer 3%	Whole grain barley bread, 3% fiber	0.00	0.00	0.00	0.00	1.00	no	Bread	no
186	Grahamsscones fullkorn	Whole grain graham scones	0.00	0.00	0.00	0.00	0.00	no	Bread	no
187	Rågbröd typ pumpernickel fullkorn fibrer ca 10%	Rye bread, pumpernickel type, whole grain, approx. 10% fiber	0.00	0.00	0.00	0.00	0.00	yes	Bread	no
189	Hårt bröd fullkorn vete råg mjölk socker fibrer ca 5% Wasa Bagateller	Crispbread with whole grain wheat, rye, milk, sugar, approx. 5% fiber (Wasa Bagateller)	0.00	0.00	0.00	0.00	0.00	no	Bread	no
193	Vitt bröd vatten typ pitabröd fibrer ca 3,5%	White bread with water (e.g., pita bread), approx. 3.5% fiber	0.00	0.00	0.00	0.00	0.00	no	Bread	no
194	Korvbröd	Hot dog bun	1.00	1.00	1.00	1.00	1.00	no	Bread	no
198	Vitt bröd osötat typ italienskt fibrer ca 2,5%	Unsweetened white bread (e.g., Italian style), approx. 2.5% fiber	0.00	1.00	1.00	1.00	1.00	no	Bread	no
200	Vitt bröd vete baguette	White wheat baguette	0.00	1.00	1.00	1.00	1.00	no	Bread	no

201 Bröd vitt fullkorn surdeg fibrer 4,4% bakad i butik typ grov baguette	White whole grain sourdough bread, 4.4% fiber, bakery-made (e.g., coarse baguette)	0.00	1.00	1.00	1.00	1.00	no	Bread	no
202 Bröd vitt fibrer 3,5%	White bread, 3.5% fiber	0.00	1.00	1.00	1.00	1.00	no	Bread	no
203 Vitt bröd tortilla fibrer 4,6%	White tortilla bread, 4.6% fiber	1.00	1.00	1.00	1.00	1.00	no	Bread	no
205 Scones vitt bröd vete	Scones, white wheat bread	0.00	0.00	0.00	0.00	1.00	no	Bread	no
207 Bröd fullkorn rågsikt fibrer ca 7% typ kavring	Whole grain rye sifted bread, approx. 7% fiber (e.g., kavring)	0.00	1.00	1.00	1.00	1.00	no	Bread	no
208 Rågsiktsbröd osötat fibrer ca 3,5%	Unsweetened sifted rye bread, approx. 3.5% fiber	0.00	1.00	1.00	1.00	1.00	no	Bread	no
210 Bröd fullkorn graham m. mjölk fibrer ca 5%	Whole grain graham bread with milk, approx. 5% fiber	0.00	1.00	1.00	1.00	1.00	yes	Bread	no
211 Rågbröd fullkorn osötat fibrer ca 9%	Whole grain rye bread, unsweetened, approx. 9% fiber	0.00	1.00	1.00	1.00	1.00	no	Bread	no
213 Bröd fullkorn råg typ rallarhalvor	Whole grain rye bread (e.g., "Rallarhalvor" type)	1.00	1.00	1.00	1.00	1.00	yes	Bread	no
214 Rågbröd fullkorn osötat fibrer ca 5%	Whole grain rye bread, unsweetened, approx. 5% fiber	0.00	1.00	1.00	1.00	1.00	no	Bread	no
215 Hårt tunnbröd vete korn socker fiber ca 3% Wasa Tunnbröd	Crisp flatbread with wheat, barley, sugar, approx. 3% fiber (Wasa)	0.00	0.00	0.00	0.00	1.00	no	Bread	no
216 Hårt tunnbröd fullkorn vete råg korn havre mjölk fibrer 7%	Crisp flatbread with whole grain wheat, rye, barley, oats, milk, 7% fiber	0.00	0.00	0.00	0.00	1.00	no	Bread	no
217 Tunnbröd vitt bröd mjukt mjölk fibrer ca 4%	Soft white flatbread with milk, approx. 4% fiber	0.00	1.00	1.00	1.00	1.00	no	Bread	no
222 Bröd vitt vatten fibrer ca 3% typ ostfralla	White bread with water, approx. 3% fiber (e.g., cheese roll)	0.00	1.00	1.00	1.00	1.00	no	Bread	no
224 Riskakor fullkorn solrosfrön majs fett 4%	Whole grain rice cakes with sunflower seeds and corn, 4% fat	0.00	0.00	0.00	0.00	0.00	no	Bread	no
225 Riskakor fullkorn smaksatta fett 18%	Flavored whole grain rice cakes, 18% fat	1.00	1.00	1.00	1.00	1.00	no	Fine bakery wares	no
226 Bröd vitt glutenfritt	Gluten-free white bread	1.00	1.00	1.00	1.00	1.00	no	Bread	no
227 Bröd mörkt glutenfritt	Gluten-free dark bread	1.00	1.00	1.00	1.00	1.00	yes	Bread	no
229 Croissant fransk giffel	Croissant (French crescent roll)	0.00	1.00	1.00	1.00	1.00	no	Bread	no
231 Höstpotatis kokt m salt	Autumn potatoes, boiled with salt	0.00	0.00	0.00	0.00	0.00	yes	Starchy roots and tubers	no
232 Potatis skalad kokt m salt	Peeled potatoes, boiled with salt	0.00	0.00	0.00	0.00	0.00	yes	Starchy roots and tubers	no
233 Potatis m skal bakad u salt	Potatoes with skin, baked without salt	0.00	0.00	0.00	0.00	0.00	yes	Starchy roots and tubers	no
236 Färskpotatis kokt u salt	New potatoes, boiled without salt	0.00	0.00	0.00	0.00	0.00	yes	Starchy roots and tubers	no
238 Klyftpotatis u skal ugnsstekt m rapsolja	Potato wedges without skin, oven-roasted with rapeseed oil	0.00	0.00	0.00	0.00	0.00	yes	Starchy roots and tubers	no
240 Potatis stekt	Panfried potatoes	0.00	0.05	0.09	0.11	0.25	no	Starchy roots and tubers	no
241 Potatis råstekt	Pan-roasted raw potatoes	0.00	0.05	0.09	0.11	0.26	no	Starchy roots and tubers	no
244 Pommes frites friterad potatis fett ca 7% fryst värmd i ugn	French fries, deep-fried, 7% fat, frozen and oven-heated	0.00	1.00	1.00	1.00	1.00	no	Savoury snacks	no
245 Pommes frites friterad potatis fett ca 11% fryst	French fries, deep-fried, 11% fat, frozen	0.00	1.00	1.00	1.00	1.00	no	Savoury snacks	no
246 Pommes frites friterad potatis fett ca 17% gatukök	French fries, deep-fried, 17% fat, fast food	1.00	1.00	1.00	1.00	1.00	no	Savoury snacks	no
249 Pytt i panna gjutjärnsstekpanna hemlagad	Homemade Swedish hash in cast iron pan	0.22	0.26	0.29	0.30	0.42	no	Meat dish	no
250 Sjömansbiff hemlagad	Beef, onion and potato stew, homemade	0.01	0.06	0.09	0.10	0.18	no	Meat dish	no
251 Kroppkakor o pitepalt	Potato dumplings and pitepalt (local specialties)	0.30	1.00	1.00	1.00	1.00	no	Meat dish	no
252 Lapskojs	Swedish hash (mashed meat and potatoes)	0.01	0.39	0.40	0.41	0.46	no	Meat dish	no
255 Pytt i panna tillagad frysvara	Meat and potato hash, ready to eat, frozen	1.00	1.00	1.00	1.00	1.00	no	Meat dish	no
257 Janssons frestelse	Jansson’s temptation (Swedish potato and anchovy gratin)	0.30	0.33	0.35	0.36	0.43	no	Potato dishes	no
258 Laxpudding	Salmon pudding	0.00	0.00	0.00	0.00	0.00	no	Fish dishes	no
261 Potatissallad gräddfil majonnäs	Potato salad with sour cream and mayonnaise	0.00	1.00	1.00	1.00	1.00	no	Potato dishes	no
262 Potatis- o purjolökssoppa	Potato and leek soup	0.00	0.00	0.00	0.00	1.00	no	Soups and salads	no
263 Pytt i panna vegetarisk rotfrukt svamp	Vegetarian hash with root vegetables and mushrooms	0.00	0.07	0.12	0.15	0.35	yes	Legume dishes	no
264 Potatissallad vinägrettsås	Potato salad with vinaigrette	0.00	0.00	0.00	0.00	0.00	no	Potato dishes	no
269 Potatismos hemlagat	Homemade mashed potatoes	0.00	0.03	0.05	0.06	0.15	no	Potato dishes	no
271 Raggmunkar potatisplättar potatispannkakor hemlagade	Potato pancakes, homemade	0.00	0.04	0.04	0.04	0.10	no	Potato dishes	no
272 Potatisstuvning	Creamed potatoes	0.00	0.06	0.06	0.06	0.14	no	Potato dishes	no
273 Potatisbullar stekta	Panfried potato patties	0.00	1.00	1.00	1.00	1.00	no	Potato dishes	no
274 Potatismos	Mashed potatoes	0.00	0.06	0.06	0.06	1.00	no	Potato dishes	no
275 Potatisgratäng m. mjölk ost hemlagad	Homemade potato gratin with milk and cheese	0.00	0.00	0.00	0.00	0.00	no	Potato dishes	no
281 Schweizisk potatiskaka rösti fryst värmd	Swiss-style potato cake (rösti), frozen and heated	1.00	1.00	1.00	1.00	1.00	no	Potato dishes	no
282 Potatiskroketter frysta värmda	Potato croquettes, frozen heated	1.00	1.00	1.00	1.00	1.00	no	Savoury snacks	no
283 Potatismos pulver berik tillagad	Mashed potatoes, powdered fortified, prepared	1.00	1.00	1.00	1.00	1.00	no	Potato dishes	no
284 Potatisgratäng grädde ost fryst värmd	Frozen potato gratin with cream and cheese, heated	1.00	1.00	1.00	1.00	1.00	no	Potato dishes	no
288 Kålrot	Rutabaga	0.00	0.00	0.00	0.00	0.00	yes	Vegetables and vegetable products	no
289 Morot	Carrot	0.00	0.00	0.00	0.00	0.00	yes	Vegetables and vegetable products	no
290 Palsternacka	Parsnip	0.00	0.00	0.00	0.00	0.00	yes	Vegetables and vegetable products	no
291 Pepparrot	Horseradish	0.00	0.00	0.00	0.00	0.00	yes	Vegetables and vegetable products	no
292 Rotselleri	Celeriac	0.00	0.00	0.00	0.00	0.00	yes	Vegetables and vegetable products	no

293 Rädisa	Radish	0.00	0.00	0.00	0.00	0.00	yes	Vegetables and vegetable products	no
294 Rödbeta	Beetroot	0.00	0.00	0.00	0.00	0.00	yes	Vegetables and vegetable products	no
297 Majrova	Turnip	0.00	0.00	0.00	0.00	0.00	yes	Vegetables and vegetable products	no
301 Morotsjuice	Carrot juice	0.00	0.00	0.00	0.00	0.00	x	Juices	no
302 Rotmos hemlagad	Homemade mashed root vegetables	0.00	0.06	0.06	0.06	0.14	yes	Vegetable dishes	no
304 Jordärtskockspuré	Jerusalem artichoke purée	0.00	0.06	0.06	0.06	0.14	no	Vegetable dishes	no
305 Morot kokt m. salt	Carrot, boiled with salt	0.00	0.00	0.00	0.00	0.00	yes	Vegetables and vegetable products	no
306 Morötter stuvade	Carrots, creamed	0.01	0.07	0.07	0.07	0.15	yes	Vegetable dishes	no
307 Morotssoppa	Carrot soup	0.06	0.06	0.06	0.06	1.00	yes	Soups and salads	no
310 Jordärtskocka kokt m. salt	Jerusalem artichoke, boiled with salt	0.00	0.00	0.00	0.00	0.00	yes	Vegetables and vegetable products	no
311 Kålrot kokt	Rutabaga, boiled	0.00	0.00	0.00	0.00	0.00	yes	Vegetables and vegetable products	no
312 Rotselleri kokt	Boiled celeriac	0.00	0.00	0.00	0.00	0.00	yes	Vegetables and vegetable products	no
315 Rotsaksbiff stekt	Root vegetable patty, panfried	0.00	0.00	0.00	0.00	1.00	no	Vegetable dishes	no
316 Rödbeta kokt u. salt	Beetroot, boiled without salt	0.00	0.00	0.00	0.00	0.00	yes	Vegetables and vegetable products	no
317 Rödbetssoppa typ Borsjtj	Beetroot soup, Borscht-style	0.10	0.22	0.22	0.22	0.33	yes	Soups and salads	no
318 Rotmos pulver berik tillagad	Mashed root vegetables, fortified (powdered, prepared)	1.00	1.00	1.00	1.00	1.00	no	Vegetable dishes	no
319 Rödbeta inlagd u lag	Beetroot, pickled without brine	0.00	1.00	1.00	1.00	1.00	no	Vegetables and vegetable products	no
320 Avokado	Avocado	0.00	0.00	0.00	0.00	0.00	yes	Vegetables and vegetable products	no
321 Stjälkselleri	Celery stalk	0.00	0.00	0.00	0.00	0.00	yes	Vegetables and vegetable products	no
322 Blomkål	Cauliflower	0.00	0.00	0.00	0.00	0.00	yes	Vegetables and vegetable products	no
324 Bondbönor färska kokta u. salt	Broad beans, fresh, boiled without salt	0.00	0.00	0.00	0.00	0.00	yes	Vegetables and vegetable products	no
325 Broccoli	Broccoli	0.00	0.00	0.00	0.00	0.00	yes	Vegetables and vegetable products	no
331 Gröna bönor	Green beans	0.00	0.00	0.00	0.00	0.00	yes	Vegetables and vegetable products	no
333 Champinjoner färska	Mushrooms, fresh	0.00	0.00	0.00	0.00	0.00	yes	Vegetables and vegetable products	no
335 Endivesallat	Endive lettuce	0.00	0.00	0.00	0.00	0.00	yes	Vegetables and vegetable products	no
336 Fänkål	Fennel	0.00	0.00	0.00	0.00	0.00	yes	Vegetables and vegetable products	no
337 Grönkål	Kale	0.00	0.00	0.00	0.00	0.00	yes	Vegetables and vegetable products	no
339 Gurka	Cucumber	0.00	0.00	0.00	0.00	0.00	yes	Vegetables and vegetable products	no
340 Isbergssallat	Iceberg lettuce	0.00	0.00	0.00	0.00	0.00	yes	Vegetables and vegetable products	no
343 Kålrabbi	Kohlrabi	0.00	0.00	0.00	0.00	0.00	yes	Vegetables and vegetable products	no
344 Lök gul	Onion, yellow	0.00	0.00	0.00	0.00	0.00	yes	Vegetables and vegetable products	no
347 Majskorn frysta	Corn kernels, frozen	0.00	0.00	0.00	0.00	0.00	yes	Vegetables and vegetable products	no
348 Mangold	Swiss chard	0.00	0.00	0.00	0.00	0.00	yes	Vegetables and vegetable products	no
352 Persilja blad	Parsley, leaves	0.00	0.00	0.00	0.00	0.00	yes	Vegetables and vegetable products	no
354 Purjolök	Leek	0.00	0.00	0.00	0.00	0.00	yes	Vegetables and vegetable products	no
355 Rödkål	Red cabbage	0.00	0.00	0.00	0.00	0.00	yes	Vegetables and vegetable products	no
356 Huvudsallat	Head lettuce	0.00	0.00	0.00	0.00	0.00	yes	Vegetables and vegetable products	no
357 Savojkål	Savoy cabbage	0.00	0.00	0.00	0.00	0.00	yes	Vegetables and vegetable products	no
358 Salladskål	Salad cabbage	0.00	0.00	0.00	0.00	0.00	yes	Vegetables and vegetable products	no
359 Sockerärtor	Sugar snap peas	0.00	0.00	0.00	0.00	0.00	yes	Vegetables and vegetable products	no
360 Spenat	Spinach	0.00	0.00	0.00	0.00	0.00	yes	Vegetables and vegetable products	no
362 Squash	Squash (Zucchini)	0.00	0.00	0.00	0.00	0.00	yes	Vegetables and vegetable products	no
364 Tomat	Tomato	0.00	0.00	0.00	0.00	0.00	yes	Vegetables and vegetable products	no
365 Soltorkade tomater i olja	Sun-dried tomatoes in oil	0.00	1.00	1.00	1.00	1.00	no	Vegetables and vegetable products	no
366 Trädgårdskrasse	Garden cress	0.00	0.00	0.00	0.00	0.00	yes	Vegetables and vegetable products	no
370 Vitkål	White cabbage	0.00	0.00	0.00	0.00	0.00	yes	Vegetables and vegetable products	no
371 Vitlök	Garlic	0.00	0.00	0.00	0.00	0.00	yes	Vegetables and vegetable products	no
373 Gröna ärtor	Green peas	0.00	0.00	0.00	0.00	0.00	yes	Vegetables and vegetable products	no
376 Alfalfagroddar	Alfalfa sprouts	0.00	0.00	0.00	0.00	0.00	yes	Vegetables and vegetable products	no
377 Dill	Dill	0.00	0.00	0.00	0.00	0.00	yes	Vegetables and vegetable products	no
378 Gräslök	Chives	0.00	0.00	0.00	0.00	0.00	yes	Vegetables and vegetable products	no
379 Basilika färsk	Basil, fresh	0.00	0.00	0.00	0.00	0.00	yes	Vegetables and vegetable products	no
384 Linsgroddar	Lentil sprouts	0.00	0.00	0.00	0.00	0.00	yes	Vegetables and vegetable products	no
389 Grönsaksblandning ärter majs paprika fryst	Vegetable mix, peas, corn, bell pepper, frozen	0.00	0.00	0.00	0.00	0.00	yes	Vegetables and vegetable products	no
390 Grönsallat gurka tomat u dressing	Green salad, cucumber, tomato, no dressing	0.00	0.00	0.00	0.00	0.00	yes	Vegetables and vegetable products	no
392 Paprika grön gul röd	Bell pepper, green/yellow/red	0.00	0.00	0.00	0.00	0.00	yes	Vegetables and vegetable products	no
393 Bambuskott konserv u lag	Bamboo shoots, canned, drained	0.00	0.00	0.00	0.00	0.00	yes	Vegetables and vegetable products	no
399 Löksoppa pulver tillagad	Onion soup, powdered, prepared	1.00	1.00	1.00	1.00	1.00	no	Soups and salads	no
400 Majskorn konserv u lag	Corn kernels, canned, drained	0.00	0.00	0.00	0.00	0.00	yes	Vegetables and vegetable products	no
401 Oliver gröna m paprikafyllning avrunna	Green olives, stuffed with pepper, drained	1.00	1.00	1.00	1.00	1.00	no	Vegetables and vegetable products	no

403 Grönsaksblandning inlagda el pickels sötad u lag	Vegetable mix, pickled or sweetened, drained	1.00	1.00	1.00	1.00	1.00	no	Vegetables and vegetable products	no
404 Sparris vit konserv u lag	Asparagus, white, canned, drained	0.00	0.00	0.00	0.00	0.00	no	Vegetables and vegetable products	no
405 Sparrissoppa redd konserv tillagad	Asparagus soup, thickened, canned, prepared	1.00	1.00	1.00	1.00	1.00	no	Soups and salads	no
406 Surkål konserv m lag	Sauerkraut, canned, with brine	0.00	0.00	0.00	0.00	0.00	no	Vegetables and vegetable products	no
407 Tomater hela konserv m lag	Tomatoes, whole, canned, with brine	0.00	0.00	0.00	0.00	0.00	yes	Vegetables and vegetable products	no
408 Tomatjuice konserv drickf	Tomato juice, canned, ready to drink	1.00	1.00	1.00	1.00	1.00	x	Juices	yes
410 Tomatpuré konc konserv	Tomato purée, concentrated, canned	0.00	0.00	0.00	0.00	0.00	yes	Vegetables and vegetable products	no
411 Vaxbönor konserv u lag	Wax beans, canned, drained	0.00	0.00	0.00	0.00	0.00	no	Vegetables and vegetable products	no
413 Gröna ärter konserv u lag	Green peas, canned, drained	0.00	0.00	0.00	0.00	0.00	no	Vegetables and vegetable products	no
417 Ajvar relish grönsakspasta m paprika aubergine	Ajvar relish, vegetable paste with pepper and eggplant	0.00	0.00	0.00	0.00	1.00	no	Savoury sauces and condiments	no
418 Svamp konserv u lag	Mushrooms, canned, drained	0.00	0.00	0.00	0.00	0.00	yes	Vegetables and vegetable products	no
419 Kantareller konserv u lag	Chanterelles, canned, drained	0.00	0.00	0.00	0.00	0.00	no	Vegetables and vegetable products	no
420 Syltlök inlagd	Pickled pearl onions	1.00	1.00	1.00	1.00	1.00	no	Vegetables and vegetable products	no
421 Tomater gröna syltade	Tomatoes, green, pickled	0.00	0.00	0.00	0.00	0.00	no	Vegetables and vegetable products	no
422 Tomater krossade konserv m lag	Tomatoes, crushed, canned, with brine	0.00	0.00	0.00	0.00	0.00	yes	Vegetables and vegetable products	no
423 Fefferoni inlagd konserv	Fefferoni peppers, pickled, canned	0.00	0.00	0.00	0.00	1.00	no	Vegetables and vegetable products	no
424 Wokgrönsaker Asiatiska wokade i rapsolja	Wok vegetables, Asian style, stir-fried in rapeseed oil	0.00	0.00	0.00	0.00	0.00	no	Vegetables and vegetable products	no
425 Wokgrönsaker Classic wokade i rapsolja	Wok vegetables, Classic style, stir-fried in rapeseed oil	0.00	0.00	0.00	0.00	0.00	yes	Vegetables and vegetable products	no
427 Grönsaksjuice konserv el pastöriserad drickf	Vegetable juice, canned or pasteurized, ready to drink	0.00	0.00	0.00	0.00	1.00	x	Juices	yes
428 Champinjoner konserv u lag	Mushrooms, canned, drained	0.00	0.00	0.00	0.00	0.00	no	Vegetables and vegetable products	no
431 Spenatsoppa hemlagad	Spinach soup, homemade	0.03	0.09	0.09	0.09	0.17	yes	Soups and salads	no
432 Vitkålsstuvning hemlagad	White cabbage stew, homemade	0.01	0.07	0.07	0.07	0.15	yes	Vegetable dishes	no
434 Blomkål kokt m. salt	Cauliflower, boiled with salt	0.00	0.00	0.00	0.00	0.00	yes	Vegetables and vegetable products	no
435 Blomkålsstuvning	Cauliflower stew	0.00	0.03	0.06	0.07	0.17	yes	Vegetable dishes	no
437 Grönkålssoppa	Kale soup	0.03	0.09	0.09	0.09	0.17	no	Soups and salads	no
438 Grönsakssoppa redd ängamat	Vegetable soup, thickened (Ängamat style)	0.00	1.00	1.00	1.00	1.00	no	Soups and salads	no
439 Grönsaksbiff stekt	Vegetable patty, panfried	0.00	1.00	1.00	1.00	1.00	no	Vegetable dishes	no
444 Grönsallat gurka tomat paprika u dressing	Green salad, cucumber, tomato, bell pepper, no dressing	0.00	0.00	0.00	0.00	0.00	yes	Soups and salads	no
446 Lök stekt	Onion, panfried	0.00	0.03	0.06	0.07	0.16	no	Vegetables and vegetable products	no
447 Löksås	Onion sauce	0.24	0.35	0.40	0.42	0.86	no	Savoury sauces and condiments	no
448 Lök kokt	Onion, boiled	0.00	0.00	0.00	0.00	0.00	yes	Vegetables and vegetable products	no
450 Nässelsoppa	Nettle soup	0.03	0.09	0.09	0.09	0.17	no	Soups and salads	no
451 Paprika förvälld	Bell pepper, blanched	0.00	0.00	0.00	0.00	0.00	yes	Vegetables and vegetable products	no
452 Purjolök kokt	Leek, boiled	0.00	0.00	0.00	0.00	0.00	yes	Vegetables and vegetable products	no
453 Rödkål tillagad	Red cabbage, cooked	0.00	0.00	0.00	0.00	0.00	no	Vegetables and vegetable products	no
454 Sparrisstuvning	Asparagus stew	0.01	0.07	0.07	0.07	0.17	yes	Vegetable dishes	no
455 Spenat fräst	Spinach, sautéed	0.00	0.15	0.15	0.15	0.35	no	Vegetable dishes	no
456 Spenatstuvning	Spinach stew	0.00	1.00	1.00	1.00	1.00	no	Vegetable dishes	no
457 Champinjoner stekta	Mushrooms, panfried	0.00	0.00	0.00	0.00	0.00	no	Vegetable dishes	no
458 Champinjonstuvning	Mushroom stew	0.00	0.00	0.00	0.00	1.00	no	Vegetable dishes	no
459 Champinjonsås	Mushroom sauce	0.00	1.00	1.00	1.00	1.00	no	Savoury sauces and condiments	no
462 Tomatsalsa kall	Tomato salsa, cold	0.00	1.00	1.00	1.00	1.00	no	Savoury sauces and condiments	no
464 Vitkålssoppa vegetarisk	White cabbage soup, vegetarian	0.04	0.12	0.12	0.12	0.24	yes	Vegetable dishes	no
465 Vitkål kokt	White cabbage, boiled	0.00	0.00	0.00	0.00	0.00	yes	Vegetables and vegetable products	no
466 Brunkål	Brown cabbage (local specialty preparation)	0.30	1.00	1.00	1.00	1.00	no	Vegetable dishes	no
467 Vitkålssallad pizzasallad	White cabbage salad, pizza salad	0.00	0.00	0.00	0.00	1.00	yes	Soups and salads	no
470 Aubergine stekt	Eggplant, panfried	0.00	0.13	0.24	0.29	0.67	no	Vegetables and vegetable products	no
471 Ärtor morötter tillagade	Peas and carrots, cooked	0.00	0.00	0.00	0.00	0.00	yes	Vegetables and vegetable products	no
473 Ärtpurésoppa gröna ärtor tillagad veg.	Pea purée soup, green peas, cooked, vegetarian	0.10	0.13	0.16	0.17	0.26	no	Soups and salads	no
476 Grönsaker stuvade ospec.	Vegetables, stewed, unspecified	0.01	0.07	0.07	0.07	0.15	no	Vegetable dishes	no
477 Grönsaker kokta ospec.	Vegetables, boiled, unspecified	0.00	0.00	0.00	0.00	0.00	yes	Vegetables and vegetable products	no
478 Kikärtsgryta grönsaksgryta	Chickpea stew / Vegetable stew	0.03	0.09	0.09	0.09	0.17	yes	Vegetable dishes	no
482 Grönsakssoppa klar konserv tillagad	Vegetable soup, clear, canned, prepared	0.00	1.00	1.00	1.00	1.00	no	Soups and salads	no
484 Grönsakssoppa redd pulver tillagad m vatten mjölk	Vegetable soup, thickened, powdered, prepared with water and milk	1.00	1.00	1.00	1.00	1.00	no	Soups and salads	no
487 Gurka inlagd	Cucumber, pickled	0.00	1.00	1.00	1.00	1.00	no	Vegetables and vegetable products	no
488 Bostongurka	Boston cucumber relish	1.00	1.00	1.00	1.00	1.00	no	Vegetables and vegetable products	no
490 Saltgurka u lag	Salted cucumber, drained	1.00	1.00	1.00	1.00	1.00	no	Vegetables and vegetable products	no
491 Ättiksgurka u lag	Vinegar cucumber, drained	1.00	1.00	1.00	1.00	1.00	no	Vegetables and vegetable products	no

492	Kronärtskocka kokt	Artichoke, boiled	0.00	0.00	0.00	0.00	0.00	yes	Vegetables and vegetable products	no
493	Champinjonsoppa pulver tillagad m vatten mjölk	Mushroom soup, powdered, prepared with water and milk	1.00	1.00	1.00	1.00	1.00	no	Soups and salads	no
494	Champinjonsoppa redd konserv tillagad m mjölk	Mushroom soup, thickened, canned, prepared with milk	1.00	1.00	1.00	1.00	1.00	no	Soups and salads	no
495	Majskolv kokt	Corn cob, boiled	0.00	0.00	0.00	0.00	0.00	no	Vegetables and vegetable products	no
496	Blomkålssoppa pulver tillagad m vatten mjölk	Cauliflower soup, powdered, prepared with water and milk	1.00	1.00	1.00	1.00	1.00	no	Soups and salads	no
497	Tomatsoppa pulver tillagad m vatten	Tomato soup, powdered, prepared with water	1.00	1.00	1.00	1.00	1.00	no	Soups and salads	no
498	Tomatsoppa konserv tillagad m vatten mjölk	Tomato soup, canned, prepared with water and milk	1.00	1.00	1.00	1.00	1.00	no	Soups and salads	no
499	Sparrissoppa pulver tillagad m vatten mjölk	Asparagus soup, powdered, prepared with water and milk	1.00	1.00	1.00	1.00	1.00	no	Soups and salads	no
500	Rödkål konserv.	Red cabbage, canned	0.00	1.00	1.00	1.00	1.00	no	Vegetables and vegetable products	no
501	Champinjonsås pulver tillagad m mjölk smör	Mushroom sauce, powdered, prepared with milk and butter	1.00	1.00	1.00	1.00	1.00	no	Savoury sauces and condiments	no
502	Grönsaker blandade fermenterade	Vegetables, mixed, fermented	0.00	0.00	0.00	0.00	0.00	no	Vegetables and vegetable products	no
506	Minestronesoppa	Minestrone soup	0.00	0.50	0.50	0.50	1.00	no	Soups and salads	no
512	Kålpudding	Cabbage pudding	0.11	0.11	0.11	0.11	1.00	no	Meat dish	no
513	Caesarsallad kycklingsallad m dressing	Caesar salad, chicken salad with dressing	0.00	0.17	0.17	0.17	1.00	no	Soups and salads	no
515	Moussaka ugnsbakad	Moussaka, oven-baked	0.02	0.08	0.08	0.08	0.15	no	Meat dish	no
518	Wokgrönsaker m kyckling nudlar	Wok vegetables, with chicken and noodles	0.02	0.08	0.08	0.08	0.16	no	Meat dish	no
520	Granatäpple	Pomegranate	0.00	0.00	0.00	0.00	0.00	yes	Fruit	no
521	Grapefrukt	Grapefruit	0.00	0.00	0.00	0.00	0.00	yes	Fruit	no
523	Hallon	Raspberries	0.00	0.00	0.00	0.00	0.00	yes	Fruit	no
525	Hjortron	Cloudberries	0.00	0.00	0.00	0.00	0.00	yes	Fruit	no
526	Jordgubbar	Strawberries	0.00	0.00	0.00	0.00	0.00	yes	Fruit	no
529	Grekisk sallad	Greek salad	0.00	0.00	0.00	0.00	0.00	no	Soups and salads	no
530	Grönsakssufflé	Vegetable soufflé	0.00	0.00	0.00	0.00	0.00	no	Vegetable dishes	no
536	Kelp	Kelp	0.00	0.00	0.00	0.00	0.00	no	Vegetables and vegetable products	no
538	Quorn svampprotein färs bitar filé	Quorn, mushroom protein, mince/pieces/fillet	1.00	1.00	1.00	1.00	1.00	yes	Meat and dairy imitations	no
543	Wokgrönsaker m nudlar	Wok vegetables, with noodles	0.02	0.08	0.08	0.08	0.15	no	Pasta dishes	no
544	Vegetabilisk pastej typ tartex	Vegetable pâté, Tartex type	1.00	1.00	1.00	1.00	1.00	no	Savoury sauces and condiments	no
545	Grönsaksbuljong ätf.	Vegetable broth, ready to eat	1.00	1.00	1.00	1.00	1.00	x	Savoury sauces and condiments	no
546	Honungsmelon	Melon, honeydew	0.00	0.00	0.00	0.00	0.00	yes	Fruit	no
547	Nätmelon	Melon, cantaloupe	0.00	0.00	0.00	0.00	0.00	yes	Fruit	no
548	Rabarber	Rhubarb	0.00	0.00	0.00	0.00	0.00	yes	Fruit	no
549	Vattenmelon	Melon, watermelon	0.00	0.00	0.00	0.00	0.00	yes	Fruit	no
550	Ananas	Pineapple	0.00	0.00	0.00	0.00	0.00	yes	Fruit	no
551	Apelsin	Orange	0.00	0.00	0.00	0.00	0.00	yes	Fruit	no
552	Aprikos	Apricot	0.00	0.00	0.00	0.00	0.00	yes	Fruit	no
553	Banan	Banana	0.00	0.00	0.00	0.00	0.00	yes	Fruit	no
554	Björnbär	Blackberry	0.00	0.00	0.00	0.00	0.00	yes	Fruit	no
555	Blåbär	Blueberry	0.00	0.00	0.00	0.00	0.00	yes	Fruit	no
556	Blåbär frysta	Blueberries, frozen	0.00	0.00	0.00	0.00	0.00	yes	Fruit	no
558	Cherimoya	Cherimoya	0.00	0.00	0.00	0.00	0.00	yes	Fruit	no
559	Citron	Lemon	0.00	0.00	0.00	0.00	0.00	yes	Fruit	no
560	Småcitrus clementiner mandariner tangeriner	Small citrus fruits, clementines/mandarins/tangerines	0.00	0.00	0.00	0.00	0.00	yes	Fruit	no
561	Fikon	Fig	0.00	0.00	0.00	0.00	0.00	yes	Fruit	no
565	Kiwi grön	Kiwi, green	0.00	0.00	0.00	0.00	0.00	yes	Fruit	no
566	Krusbär	Gooseberry	0.00	0.00	0.00	0.00	0.00	yes	Fruit	no
567	Physalis	Physalis	0.00	0.00	0.00	0.00	0.00	yes	Fruit	no
570	Surkörsbär	Sour cherry	0.00	0.00	0.00	0.00	0.00	yes	Fruit	no
571	Sötkörsbär	Sweet cherry	0.00	0.00	0.00	0.00	0.00	yes	Fruit	no
572	Lime	Lime	0.00	0.00	0.00	0.00	0.00	yes	Fruit	no
573	Lingon	Lingonberries	0.00	0.00	0.00	0.00	0.00	yes	Fruit	no
574	Mango	Mango	0.00	0.00	0.00	0.00	0.00	yes	Fruit	no
576	Nektarin	Nectarine	0.00	0.00	0.00	0.00	0.00	yes	Fruit	no
577	Papaya	Papaya	0.00	0.00	0.00	0.00	0.00	yes	Fruit	no
579	Passionsfrukt	Passion fruit	0.00	0.00	0.00	0.00	0.00	yes	Fruit	no
580	Persika nektarin	Peach / Nectarine	0.00	0.00	0.00	0.00	0.00	yes	Fruit	no
581	Sharon	Persimmon (Sharon fruit)	0.00	0.00	0.00	0.00	0.00	yes	Fruit	no
582	Plommon	Plum	0.00	0.00	0.00	0.00	0.00	yes	Fruit	no
583	Päron	Pear	0.00	0.00	0.00	0.00	0.00	yes	Fruit	no
584	Tranbär	Cranberries	0.00	0.00	0.00	0.00	0.00	yes	Fruit	no
585	Röda vinbär	Red currants	0.00	0.00	0.00	0.00	0.00	yes	Fruit	no

586	Svarta vinbär	Black currants	0.00	0.00	0.00	0.00	0.00	yes	Fruit	no
587	Vindruvor	Grapes	0.00	0.00	0.00	0.00	0.00	yes	Fruit	no
588	Äpple m skal	Apple, with peel	0.00	0.00	0.00	0.00	0.00	yes	Fruit	no
589	Äpple u. skal	Apple, peeled	0.00	0.00	0.00	0.00	0.00	yes	Fruit	no
590	Hallon frysta	Raspberries, frozen	0.00	0.00	0.00	0.00	0.00	yes	Fruit	no
591	Björnbär frysta	Blackberries, frozen	0.00	0.00	0.00	0.00	0.00	yes	Fruit	no
593	Jordgubbar frysta	Strawberries, frozen	0.00	0.00	0.00	0.00	0.00	yes	Fruit	no
594	Bär färska	Berries, fresh	0.00	0.00	0.00	0.00	0.00	yes	Fruit	no
595	Frukt färsk	Fruit, fresh	0.00	0.00	0.00	0.00	0.00	yes	Fruit	no
596	Citrusfrukter	Citrus fruits	0.00	0.00	0.00	0.00	0.00	yes	Fruit	no
599	Aprikoser torkade	Apricots, dried	0.00	0.00	0.00	0.00	0.00	x	Fruit products	no
602	Citronskal	Lemon zest	0.00	0.00	0.00	0.00	0.00	yes	Fruit products	no
603	Dadlar torkade	Dates, dried	0.00	0.00	0.00	0.00	0.00	x	Fruit products	no
604	Fikon torkade	Figs, dried	0.00	0.00	0.00	0.00	0.00	x	Fruit products	no
605	Katrinplommon 32,5 % vatten	Prunes, 32.5% water	0.00	0.00	0.00	0.00	0.00	x	Fruit products	no
610	Russin	Raisins	0.00	0.00	0.00	0.00	0.00	x	Fruit products	no
611	Äpplen torkade	Apples, dried	0.00	0.00	0.00	0.00	0.00	x	Fruit products	no
612	Frukt torkad	Fruit, dried	0.00	0.00	0.00	0.00	0.00	x	Fruit products	no
614	Ananas konserv m juice	Pineapple, canned in juice	0.00	0.00	0.00	0.00	0.00	x	Fruit products	no
619	Aprikoser konserv m medelsöt lag	Apricots, canned in medium-sweet syrup	0.00	1.00	1.00	1.00	1.00	x	Fruit products	no
625	Persikor konserv m medelsöt lag	Peaches, canned in medium-sweet syrup	1.00	1.00	1.00	1.00	1.00	x	Fruit products	no
628	Päron konserv m medelsöt lag	Pears, canned in medium-sweet syrup	0.00	0.00	0.00	0.00	1.00	x	Fruit products	no
632	Fruktcocktail konserv m medelsöt lag	Fruit cocktail, canned in medium-sweet syrup	1.00	1.00	1.00	1.00	1.00	x	Fruit products	no
633	Mandarin konserv m lättsöt lag	Mandarins, canned in lightly sweetened syrup	0.00	0.00	0.00	0.00	0.00	x	Fruit products	no
634	Mango konserv m medelsöt lag	Mango, canned in medium-sweet syrup	1.00	1.00	1.00	1.00	1.00	x	Fruit products	no
636	Frukt konserverad	Fruit, canned	0.00	1.00	1.00	1.00	1.00	x	Fruit products	no
638	Fruktsallad	Fruit salad	0.00	0.00	0.00	0.00	0.00	yes	Fruit products	no
643	Apelsinjuice drickf	Orange juice, ready to drink	0.00	1.00	1.00	1.00	1.00	x	Juices	yes
645	Citronjuice färskpressad	Lemon juice, freshly pressed	0.00	0.00	0.00	0.00	0.00	x	Juices	no
647	Småcitrusjuice färskpressad	Small citrus juice, freshly pressed	0.00	0.00	0.00	0.00	0.00	x	Juices	yes
649	Grapefruktjuice färskpressad	Grapefruit juice, freshly pressed	0.00	0.00	0.00	0.00	0.00	x	Juices	yes
652	Limejuice färskpressad	Lime juice, freshly pressed	0.00	0.00	0.00	0.00	0.00	x	Juices	no
655	Risgrynsgröt	Rice porridge	0.00	0.00	0.00	0.00	0.00	no	Pasta dishes	no
658	Druvjuice konserv drickf	Grape juice, canned, ready to drink	1.00	1.00	1.00	1.00	1.00	x	Juices	yes
659	Äppeljuice drickf	Apple juice, ready to drink	0.00	1.00	1.00	1.00	1.00	x	Juices	yes
660	Ananasjuice konserv drickf	Pineapple juice, canned, ready to drink	0.00	1.00	1.00	1.00	1.00	x	Juices	yes
661	Apelsinjuice drickf pastöriserad	Orange juice, pasteurized, ready to drink	0.00	0.00	0.00	0.00	0.00	x	Juices	yes
662	Juice ospec	Juice, unspecified	0.00	1.00	1.00	1.00	1.00	x	Juices	yes
663	Juice ej citrus ospec	Juice, non-citrus, unspecified	0.00	1.00	1.00	1.00	1.00	x	Juices	yes
667	Multivitamindryck tillverkad av konc drickf	Multivitamin drink, made from concentrate, ready to drink	1.00	1.00	1.00	1.00	1.00	x	Sugar sweetened beverages	yes
668	Apelsinnektar osötad drickf pastöriserad berik	Orange nectar, unsweetened, pasteurized, fortified	1.00	1.00	1.00	1.00	1.00	x	Sugar sweetened beverages	yes
669	Nektar drickf	Nectar, ready to drink	1.00	1.00	1.00	1.00	1.00	x	Sugar sweetened beverages	yes
672	Havredryck jordgubb/skogsbär berikad Oatly	Oat drink, strawberry/forest berries, fortified (Oatly)	1.00	1.00	1.00	1.00	1.00	no	Meat and dairy imitations	yes
675	Bovetegröt m. russin	Buckwheat porridge, with raisins	0.00	0.00	0.00	0.00	0.00	no	Breakfast cereals	no
676	Grahamsgröt	Graham porridge	0.00	0.00	0.00	0.00	0.00	yes	Breakfast cereals	no
677	Havregrynsgröt fullkorn	Oatmeal porridge, whole grain	0.00	0.00	0.00	0.00	0.00	yes	Breakfast cereals	no
678	Molinogröt grahamsmjöl torkad frukt linfrö	Molino porridge, graham flour, dried fruit, flaxseed	0.00	0.00	0.00	0.00	0.00	no	Breakfast cereals	no
679	Frukostflingor puffat rostat ris Vårgårda ris	Breakfast cereal, puffed toasted rice (Vårgårda rice)	0.00	0.00	0.00	0.00	0.00	no	Breakfast cereals	no
680	Rågkross fullkorn rågflingor ångprep	Crushed rye, whole grain rye flakes, steam-prepared	0.00	0.00	0.00	0.00	0.00	yes	Breakfast cereals	no
681	Kornmjölsgröt	Barley porridge	0.00	0.00	0.00	0.00	0.00	yes	Breakfast cereals	no
682	Mannagrynsgröt	Semolina porridge	0.00	0.00	0.00	0.00	0.00	no	Breakfast cereals	no
683	Kruskagröt fullkorn russin havregryn veteflingor vetekli	Kruska porridge, whole grain, with raisins, oats, wheat flakes, wheat bran	0.00	0.00	0.00	0.00	0.00	no	Breakfast cereals	no
684	Rågmjölsgröt	Rye porridge	0.00	0.00	0.00	0.00	0.00	yes	Breakfast cereals	no
694	Välling för vuxna fullkorn berik	Porridge drink for adults, whole grain, fortified	0.00	1.00	1.00	1.00	1.00	no	Breakfast cereals	yes
695	Havrevälling	Oat porridge drink	0.00	0.00	0.00	0.00	1.00	no	Breakfast cereals	yes
699	Välling för vuxna fullkorn osockrad berik	Porridge drink for adults, whole grain, unsweetened, fortified	1.00	1.00	1.00	1.00	1.00	no	Breakfast cereals	yes
700	Havredryck fett 1,5% berikad	Oat drink, 1.5% fat, fortified	0.00	1.00	1.00	1.00	1.00	yes	Meat and dairy imitations	yes
702	Havregryn fullkorn ångprep el havremjöl fullkorn	Oats, whole grain, steam-prepared or oat flour, whole grain	0.00	0.00	0.00	0.00	0.00	yes	Breakfast cereals	no
703	Fiberhavregryn fullkorn ångprep	Fiber oats, whole grain, steam-prepared	0.00	0.00	0.00	0.00	0.00	yes	Breakfast cereals	no

704 Havrekli	Oat bran	0.00	0.00	0.00	0.00	0.00	yes	Cereals	no
706 Frukostflingor typ corn flakes majs berik	Breakfast cereal, corn flakes type, corn-based, fortified	0.00	1.00	1.00	1.00	1.00	no	Breakfast cereals	no
708 Vetegroddar	Wheat germ	0.00	0.00	0.00	0.00	0.00	yes	Cereals	no
709 Vetekli	Wheat bran	0.00	0.00	0.00	0.00	0.00	yes	Cereals	no
710 Frukostflingor fullkornsvete Weetabix	Breakfast cereal, whole grain wheat (Weetabix)	1.00	1.00	1.00	1.00	1.00	yes	Breakfast cereals	no
711 Kruskakli grovt vetekli	Kruska bran, coarse wheat bran	0.00	0.00	0.00	0.00	0.00	yes	Cereals	no
712 Frukostflingor flingblandning fullkorn typ basmüsli	Breakfast cereal, mixed flakes, whole grain (basic muesli type)	0.00	1.00	1.00	1.00	1.00	yes	Breakfast cereals	no
713 Frukostflingor berik Rice Krispies	Breakfast cereal, fortified (Rice Krispies)	0.00	0.00	0.00	0.00	0.00	no	Breakfast cereals	no
714 Frukostflingor vetekli malt rostat Allbran Plus	Breakfast cereal, wheat bran, malted and toasted (Allbran Plus)	1.00	1.00	1.00	1.00	1.00	no	Breakfast cereals	no
715 Frukostflingor fullkorn berik Havrefras	Breakfast cereal, whole grain, fortified (Havrefras)	1.00	1.00	1.00	1.00	1.00	yes	Breakfast cereals	no
716 Frukostflingor ris socker kakao Coco Pops	Breakfast cereal, rice, sugar, cocoa (Coco Pops)	1.00	1.00	1.00	1.00	1.00	no	Breakfast cereals	no
721 Frukostflingor flingblandning müsli fullkorn frukt nötter socker el honung	Breakfast cereal, muesli mix, whole grain, fruit, nuts, sugar or honey	0.00	1.00	1.00	1.00	1.00	no	Breakfast cereals	no
726 Frukostflingor berik Kalaspuffar	Breakfast cereal, fortified (Kalaspuffar)	1.00	1.00	1.00	1.00	1.00	no	Breakfast cereals	no
727 Frukostflingor berik Frosties	Breakfast cereal, fortified (Frosties)	0.00	1.00	1.00	1.00	1.00	no	Breakfast cereals	no
729 Frukostflingor corn flakes ospec	Breakfast cereal, corn flakes, unspecified	0.00	1.00	1.00	1.00	1.00	no	Breakfast cereals	no
731 Pannkaka tunn hemlagad	Pancake, thin, homemade	0.00	0.00	0.00	0.00	0.00	no	Fine bakery wares	no
732 Fattiga riddare	French toast	0.00	0.53	0.57	0.59	0.73	no	Fine bakery wares	no
735 Ugnspannkaka mellanmjölk	Oven pancake, made with semi-skimmed milk	0.00	0.05	0.09	0.10	0.24	no	Egg dishes	no
736 Våfflor äggvåfflor	Waffles, with egg	0.00	0.10	0.17	0.21	0.49	no	Fine bakery wares	no
737 Våffla u. ägg	Waffle, without egg	0.00	0.03	0.06	0.07	1.00	no	Fine bakery wares	no
739 Fläskpannkaka	Pork pancake	0.31	0.33	0.35	0.36	0.42	no	Egg dishes	no
740 Räkcrêpe mellanmjölk mjölk vispgrädde (crêpe 56 % fyllning 44 %) hemlagad	Shrimp crêpe, with semi-skimmed milk, milk, and cream (56% crêpe, 44% filling), homemade	0.00	0.08	0.08	0.08	0.18	no	Fish dishes	no
742 Champinjoncrêpe mellanmjölk mjölk vispgrädde (crêpe 56% fyllning 44 %) hemlagad	Mushroom crêpe, with semi-skimmed milk, milk, and cream (56% crêpe, 44% filling), homemade	0.00	0.00	0.00	0.00	0.00	no	Vegetable dishes	no
744 Ciabatta med mozzarella soltorkad tomat sallad	Ciabatta, with mozzarella, sun-dried tomato, and salad	0.00	0.72	0.72	0.72	1.00	no	Sandwiches, pizzas and similar	no
748 Crêpes pannkaka tunn fylld ospec.	Crêpe, thin pancake, filled, unspecified	0.00	0.00	0.00	0.00	1.00	no	Fine bakery wares	no
750 Vårrulle fryst värmd	Spring roll, frozen, heated	0.00	1.00	1.00	1.00	1.00	no	Sandwiches, pizzas and similar	no
751 Vårrulle värmd tillagad på restaurang	Spring roll, heated, restaurant-prepared	0.00	1.00	1.00	1.00	1.00	no	Sandwiches, pizzas and similar	no
756 Paj köttfärs	Pie, minced meat	0.00	1.00	1.00	1.00	1.00	no	Sandwiches, pizzas and similar	no
757 Paj kyckling bacon	Pie, chicken and bacon	0.00	1.00	1.00	1.00	1.00	no	Sandwiches, pizzas and similar	no
758 Pirog m köttfärsfyllning	Pirogue, with minced meat filling	0.00	1.00	1.00	1.00	1.00	no	Sandwiches, pizzas and similar	no
759 Pizza m skinka salami köttfärs	Pizza, with ham, salami, and minced meat	0.08	0.50	0.50	0.50	1.00	no	Sandwiches, pizzas and similar	no
761 Pirog köttpirog fryst värmd	Pirogue, meat-filled, frozen, heated	1.00	1.00	1.00	1.00	1.00	no	Sandwiches, pizzas and similar	no
762 Pizza Capricciosa rökt skinka fryst el kyld värmd	Pizza, Capricciosa with smoked ham, frozen/chilled, heated	1.00	1.00	1.00	1.00	1.00	no	Sandwiches, pizzas and similar	no
765 Pizza pan pizza rökt griskött fryst värmd	Pan pizza, with smoked pork, frozen, heated	1.00	1.00	1.00	1.00	1.00	no	Sandwiches, pizzas and similar	no
766 Pizza pan pizza m rökt griskött restaurang	Pan pizza, with smoked pork, restaurant	0.02	1.00	1.00	1.00	1.00	no	Sandwiches, pizzas and similar	no
768 Paj m. lax spenat	Pie, with salmon and spinach	0.00	0.05	0.09	0.11	0.25	no	Sandwiches, pizzas and similar	no
771 Skaldjurspaj fryst värmd	Seafood pie, frozen, heated	1.00	1.00	1.00	1.00	1.00	no	Sandwiches, pizzas and similar	no
775 Kycklingburgare McDonalds	Chicken burger, McDonald's	1.00	1.00	1.00	1.00	1.00	no	Sandwiches, pizzas and similar	no
776 Fiskburgare McDonalds	Fish burger, McDonald's	1.00	1.00	1.00	1.00	1.00	no	Sandwiches, pizzas and similar	no
788 Pitabröd m falafel sallad sesam pepperoni	Pita bread, with falafel, salad, sesame, and pepperoni	0.00	1.00	1.00	1.00	1.00	no	Sandwiches, pizzas and similar	no
793 Matmuffins grov fullkorn m getost fårost broccoli, spenat soltorkade tomater	Savory muffin, coarse whole grain, with goat cheese, feta, broccoli, spinach, sun-dried tomatoes	0.00	0.00	0.00	0.00	0.00	no	Sandwiches, pizzas and similar	no
794 Pirog ospec.	Pirogue, unspecified	1.00	1.00	1.00	1.00	1.00	no	Sandwiches, pizzas and similar	no
796 Paj ost skinka	Pie, cheese and ham	1.00	1.00	1.00	1.00	1.00	no	Sandwiches, pizzas and similar	no
797 Smörgåstårta landgång	Sandwich cake / Open sandwich platter	0.34	1.00	1.00	1.00	1.00	no	Sandwiches, pizzas and similar	no
799 Pizza ospec.	Pizza, unspecified	0.00	0.50	0.50	0.50	1.00	no	Sandwiches, pizzas and similar	no
818 Ris vildris kokt	Rice, wild, cooked	0.00	0.00	0.00	0.00	0.00	no	Cereals	no
820 Ris olika sorter kokt m salt	Rice, various types, cooked with salt	0.00	0.00	0.00	0.00	0.00	no	Cereals	no
824 Vinbladsdolma m ris konserv	Stuffed vine leaves, with rice, canned	0.00	0.00	0.00	0.00	0.00	no	Pasta dishes	no
830 Bulgur kokt	Bulgur, cooked	0.00	0.00	0.00	0.00	0.00	no	Cereals	no
832 Couscous tillagad	Couscous, prepared	0.00	0.00	0.00	0.00	0.00	no	Cereals	no
833 Korngryn ångprep. fullkorn kokt m. salt	Barley groats, steam-prepared, whole grain, cooked with salt	0.00	0.00	0.00	0.00	0.00	yes	Cereals	no
834 Hirs kokt m. salt fullkorn	Millet, cooked with salt, whole grain	0.00	0.00	0.00	0.00	0.00	no	Cereals	no
835 Majsgryn polenta kokt	Corn grits, polenta, cooked	0.00	0.00	0.00	0.00	0.00	no	Cereals	no
837 Äppelris	Apple rice	0.00	0.00	0.00	0.00	0.00	no	Pasta dishes	no
838 Ris à la Malta	Rice pudding (Swedish style)	0.00	0.00	0.00	0.00	0.00	no	Pasta dishes	no
839 Risgrynskaka el risgrynspudding	Rice cake or rice pudding	0.00	0.00	0.00	0.00	0.00	no	Pasta dishes	no

842	Paella	Paella	0.02	0.08	0.08	0.08	0.16	no	Pasta dishes	no
853	Stuvade makaroner	Macaroni, creamed	0.01	0.07	0.07	0.07	0.15	no	Pasta dishes	no
856	Makaronipudding m skinka falukorv äggstanning	Macaroni casserole, with ham, Falukorv, and egg mixture	0.16	0.17	0.17	0.17	0.19	no	Pasta dishes	no
861	Ravioli m sås fryst el konserv	Ravioli, with sauce, frozen or canned	1.00	1.00	1.00	1.00	1.00	no	Pasta dishes	no
862	Tortellini m köttfyllning	Tortellini, with meat filling	0.00	1.00	1.00	1.00	1.00	no	Pasta dishes	no
866	Lasagne vegetarisk spenat chèvre	Lasagna, vegetarian, with spinach and chèvre	0.00	0.10	0.10	0.10	1.00	no	Pasta dishes	no
872	Tortellini ravioli m ostfyllning ospec.	Tortellini/ravioli, with cheese filling, unspecified	0.00	1.00	1.00	1.00	1.00	no	Pasta dishes	no
873	Nudelsoppa olika smaker kryddad	Noodle soup, various flavors, seasoned	1.00	1.00	1.00	1.00	1.00	no	Soups and salads	no
883	Mungbönor torkade kokta u salt	Mung beans, dried, cooked without salt	0.00	0.00	0.00	0.00	0.00	yes	Nuts and legumes	no
888	Sojabönor torkade kokta u. salt	Soybeans, dried, cooked without salt	0.00	0.00	0.00	0.00	0.00	yes	Nuts and legumes	no
892	Bruna bönor kokta	Brown beans, cooked	0.00	1.00	1.00	1.00	1.00	no	Nuts and legumes	no
897	Ärtsoppa m fläsk hemlagad	Pea soup, with pork, homemade	0.39	0.39	0.39	0.39	0.39	yes	Soups and salads	no
898	Chili con carne	Chili con carne	0.03	0.09	0.09	0.09	0.17	no	Meat dish	no
900	Ärtsoppa m fläsk konserv tillagad m vatten	Pea soup, with pork, canned, prepared with water	0.00	1.00	1.00	1.00	1.00	no	Soups and salads	no
901	Vita bönor i tomatsås konserv	White beans, in tomato sauce, canned	0.00	1.00	1.00	1.00	1.00	no	Legume dishes	no
903	Linssoppa	Lentil soup	0.04	0.12	0.12	0.12	0.24	no	Soups and salads	no
904	Ärtsoppa vegetarisk	Pea soup, vegetarian	0.00	1.00	1.00	1.00	1.00	yes	Soups and salads	no
905	Tofu sojabönsost	Tofu, soy curd	0.00	0.00	0.00	0.00	0.00	yes	Meat and dairy imitations	no
907	Sojadryck	Soy drink	0.00	1.00	1.00	1.00	1.00	yes	Meat and dairy imitations	yes
908	Miso sojabönspasta fermenterad	Miso, fermented soy paste	0.00	0.00	0.00	0.00	0.00	x	Savoury sauces and condiments	no
909	Sojasås	Soy sauce	0.00	0.00	0.00	0.00	1.00	no	Savoury sauces and condiments	no
912	Glass sojaglass mjölkfri fett 11%	Ice cream, soy-based, milk-free, 11% fat	1.00	1.00	1.00	1.00	1.00	no	Sugar and confectionery	no
915	Sojabiff stekt	Soy protein patty, panfried	1.00	1.00	1.00	1.00	1.00	no	Meat and dairy imitations	no
919	Kikärtsbiffar falafel friterade	Chickpea patties / Falafel, deep-fried	0.00	0.00	0.00	0.00	1.00	no	Legume dishes	no
998	Häst kött hamburgerkött rökt	Horse meat, smoked hamburger meat	1.00	1.00	1.00	1.00	1.00	no	Meat	no
999	Nöt saltkött rimmat kokt	Beef, salted and cured, boiled	1.00	1.00	1.00	1.00	1.00	yes	Meat	no
1000	Nötkött rökt	Smoked beef	0.00	1.00	1.00	1.00	1.00	no	Meat	no
1001	Renkött rökt	Smoked reindeer meat	0.00	0.00	0.00	0.00	0.00	no	Meat	no
1002	Ren kött torkat	Dried reindeer meat	0.00	0.00	0.00	0.00	0.00	no	Meat	no
1004	Gris bacon stekt	Bacon from pork, panfried	1.00	1.00	1.00	1.00	1.00	no	Meat	no
1005	Fläskbog gris konserv gelé 6-8%	Pork shoulder, canned with jelly 6-8%	1.00	1.00	1.00	1.00	1.00	no	Meat	no
1006	Gris kassler kotlettrad rökt	Pork loin, brined and hot smoked	1.00	1.00	1.00	1.00	1.00	no	Meat	no
1008	Gris skinka italiensk lufttorkad	Air dried ham from pork, Italian style	0.00	0.00	0.00	0.00	0.00	no	Meat	no
1009	Rökt skinka gris fett 6%	Smoked ham from pork, fat 6%	1.00	1.00	1.00	1.00	1.00	no	Meat	no
1010	Gris skinka fett 1-3% skivad rökt	Smoked ham from pork, fat 1-3%, sliced	1.00	1.00	1.00	1.00	1.00	no	Meat	no
1011	Rökt sidfläsk gris	Smoked pork belly	1.00	1.00	1.00	1.00	1.00	no	Meat	no
1014	Biff ryggbiff nöt stekt	Beef sirloin, panfried	0.00	0.00	0.00	0.00	0.00	yes	Meat	no
1015	Gris skinka julskinka fett ca 4,5% rimmad kokt	Christmas ham from pork, fat approx 4.5%, brined and boiled	1.00	1.00	1.00	1.00	1.00	no	Meat	no
1016	Gris kassler kotlettrad rökt stekt el värmd	Pork loin, brined and hot smoked, panfried or heated	1.00	1.00	1.00	1.00	1.00	no	Meat	no
1017	Nöt entrecôte stekt	Beef rib eye, panfried	0.00	0.00	0.00	0.00	0.00	yes	Meat	no
1018	Bogfläsk gris stekt	Pork shoulder, panfried	0.00	0.00	0.00	0.00	0.00	yes	Meat	no
1020	Gris sidfläsk rimmat stekt	Pork belly, brined and panfried	1.00	1.00	1.00	1.00	1.00	no	Meat	no
1023	Fläskfilé gris stekt i skivor	Pork tenderloin, panfried in slices	0.00	0.00	0.00	0.00	0.00	yes	Meat	no
1025	Fläskkotlett gris stekt	Pork chop, panfried	0.00	0.00	0.00	0.00	0.00	no	Meat	no
1027	Fläsklägg gris u svål kokt	Ham Hocks from pork, no rind, boiled	0.00	0.00	0.00	0.00	0.00	yes	Meat	no
1033	Kalvkotlett stekt	Veal chop, panfried	0.00	0.00	0.00	0.00	0.00	yes	Meat	no
1036	Lammkotlett stekt	Lamb chop, panfried	0.00	0.00	0.00	0.00	0.00	no	Meat	no
1037	Lammstek	Lamb roast	0.00	0.00	0.00	0.00	0.00	yes	Meat	no
1042	Rimmad oxbringa kokt	Beef brisket, brined and boiled	0.00	1.00	1.00	1.00	1.00	no	Meat	no
1043	Oxrulad nöt stekt	Beef roll, panfried	0.16	0.16	0.16	0.16	0.16	yes	Meat	no
1044	Nöt rostbiff ugnsstekt	Roast beef, oven roasted	0.00	0.00	0.00	0.00	0.00	yes	Meat	no
1045	Gris revbensspjäll ugnsstekt grillat	Pork spare ribs, grilled	0.00	1.00	1.00	1.00	1.00	no	Meat	no
1047	Rådjurskött ugnsstekt	Venison, roasted	0.00	0.00	0.00	0.00	0.00	yes	Meat	no
1048	Renkött kokt	Reindeer meat, boiled	0.00	0.00	0.00	0.00	0.00	yes	Meat	no
1050	Älgstek	Moose roast	0.00	0.00	0.00	0.00	0.00	yes	Meat	no
1055	Fläskkarré gris stekt u steksky	Pork neck, panfried without pan jus	0.00	0.00	0.00	0.00	0.00	no	Meat	no
1056	Skinkstek gris stekt fryst u steksky	Ham roast of pork, frozen without pan jus	0.00	0.00	0.00	0.00	0.00	yes	Meat	no
1057	Stek nöt stekt fryst u steksky	Beef steak, panfried frozen without pan jus	0.00	0.00	0.00	0.00	0.00	yes	Meat	no
1061	Kalops nötkött lök morötter sås hemlagad	Beef stew ("Kalops") with onion, carrots, sauce, homemade	0.00	0.00	0.00	0.00	0.00	no	Meat dish	no
1062	Biff Stroganoff nötkött	Beef Stroganoff, beef meat	0.00	0.00	0.00	0.00	0.00	no	Meat dish	no

1066 Wienerschnitzel gris	Wiener schnitzel from pork	0.00	1.00	1.00	1.00	1.00	no	Meat dish	no
1068 Gulasch nötkött	Gulasch beef	0.03	0.09	0.09	0.09	0.17	yes	Meat dish	no
1073 Köttsoppa nötkött	Meat soup, beef meat	0.03	0.03	0.03	0.03	1.00	yes	Soups and salads	no
1080 Renskav brynt m. matfett salt	Reindeer shavings, panfried with fat salt	0.00	0.00	0.00	0.00	0.00	no	Meat	no
1081 Renskavsgryta	Reindeer shavings, stew	0.02	0.06	0.06	0.06	0.12	no	Meat dish	no
1096 Köttsoppa nötkött konserv tillagad m vatten	Meat soup, beef , canned prepared w. water	1.00	1.00	1.00	1.00	1.00	no	Soups and salads	no
1100 Kycklingköttbullar fett 12% stekta frysta	Chicken meat balls, fat 12%, panfried frozen	1.00	1.00	1.00	1.00	1.00	no	Meat dish	no
1104 Burgare veg. soja- och veteprotein tillagad	Burger veg. soy- and wheat protein cooked	1.00	1.00	1.00	1.00	1.00	no	Meat and dairy imitations	no
1106 Köttbullar stekta hemlagade	Meatballs panfried homemade	0.00	0.00	0.00	0.00	0.00	yes	Meat dish	no
1109 Pannbiff gris stekt	Pork patties, panfried	0.00	0.00	0.00	0.00	0.00	no	Meat dish	no
1110 Hamburgare fett ca 9% stekt	Hamburger, approx. 9% fat, frid	0.00	1.00	1.00	1.00	1.00	no	Meat dish	no
1112 Köttfärsjärpar blandfärs 70/30 stekta	Meat patties, mixed mince (70% beef / 30% pork), panfried	0.00	0.00	0.00	0.00	1.00	no	Meat dish	no
1113 Kalvfrikadeller	Veal meatballs	0.00	0.00	0.00	0.00	0.00	yes	Meat dish	no
1115 Köttfärssås nöt	Gound beef sauce (e.g., Bolognese)	0.04	0.06	0.08	0.09	0.15	no	Meat dish	no
1119 Köttbullar nöt stekta	Beef meatballs, panfried	0.00	0.50	0.50	0.50	1.00	yes	Meat dish	no
1120 Köttbullar gris stekta	Pork meatballs, panfried	0.00	0.00	0.00	0.00	0.00	no	Meat dish	no
1124 Nöt färs tacokryddad stekt	Ground beef, taco-seasoned, panfried	0.06	0.08	0.09	0.10	0.15	no	Meat dish	no
1125 Pannbiff lamm stekt	Lamb patties, panfried	0.00	0.00	0.00	0.00	0.00	no	Meat dish	no
1126 Köttfärslimpa grisfärs	Meatloaf ground pork	0.00	0.00	0.00	0.00	0.00	no	Meat dish	no
1127 Köttfärslimpa blandfärs	Meatloaf mixed mince	0.00	0.00	0.00	0.00	0.00	no	Meat dish	no
1128 Pannbiff nöt stekt	Beef patties, panfried	0.00	1.00	1.00	1.00	1.00	no	Meat dish	no
1129 Pannbiff blandfärs stekt	Meat patties, mixed mince, panfried	0.00	1.00	1.00	1.00	1.00	no	Meat dish	no
1134 Kycklingburgare fryst stekt	Chicken burger, frozen, panfried	0.00	1.00	1.00	1.00	1.00	no	Meat dish	no
1138 Hamburgare nöt stekt frysvara	Beff hamburger, panfried, frozen product	0.00	1.00	1.00	1.00	1.00	no	Meat dish	no
1146 Kåldolmar stekta frysta u sås	Stuffed cabbage rolls, panfried, frozen, without sauce	0.00	0.00	0.00	0.00	1.00	no	Meat dish	no
1152 Anka	Duck	0.00	0.00	0.00	0.00	0.00	yes	Meat	no
1170 Kycklingbröstfilé u skinn ugnsstekt	Chicken breast fillet, skinless, oven-roasted	0.00	0.00	0.00	0.00	0.00	yes	Meat	no
1172 Kycklingbröstfilé u skinn wokad strimlor rapsolja	Chicken breast fillet, skinless, stripes stir-fried in rapeseed oil	0.00	0.00	0.00	0.00	0.00	yes	Meat	no
1177 Strutsfilé stekt	Ostrich fillet, panfried	0.00	0.00	0.00	0.00	0.00	yes	Meat	no
1178 Kalkon kokt	Turkey, boiled	0.00	1.00	1.00	1.00	1.00	yes	Meat	no
1181 Kyckling kokt	Chicken, boiled	0.00	0.00	0.00	0.00	0.00	yes	Meat	no
1182 Kyckling m skinn grillad	Chicken with skin, grilled	0.00	1.00	1.00	1.00	1.00	no	Meat	no
1184 Gås stekt	Goose, roasted	0.00	0.00	0.00	0.00	0.00	no	Meat	no
1186 Kalkon m skinn stekt	Turkey with skin, panfried	0.00	0.00	0.00	0.00	0.00	yes	Meat	no
1190 Kyckling kött kokt stekt grillad ospec	Chicken meat, boiled fried grilled not specified	0.00	0.00	0.00	0.00	1.00	yes	Meat	no
1191 Kalkon rökt	Turkey, smoked	1.00	1.00	1.00	1.00	1.00	yes	Meat	no
1193 Friterad kyckling	Deep fried chicken	0.00	1.00	1.00	1.00	1.00	no	Meat dish	no
1198 Ostsufflé	Cheese soufflé	0.00	0.00	0.00	0.00	0.00	no	Egg dishes	no
1199 Bondomelett	Omelet with potato and bacon	0.21	0.26	0.31	0.33	0.48	no	Egg dishes	no
1206 Kycklingsoppa thailändsk	Chicken soup, Thai style	0.02	0.37	0.38	0.38	0.42	no	Soups and salads	no
1207 Kyckling friterad Chicken McNuggets	Chicken deep fried Chicken McNuggets	1.00	1.00	1.00	1.00	1.00	no	Meat dish	no
1208 Kycklingsoppa redd pulver tillagad m vatten mjölk	Creamy chicken soup, powdered, prepared with water and milk	1.00	1.00	1.00	1.00	1.00	no	Soups and salads	no
1209 Kycklingsoppa redd pulver tillagad m vatten typ Varma koppen	Creamy chicken soup, powdered, prepared with water (e.g., "Varma koppen" type)	1.00	1.00	1.00	1.00	1.00	no	Soups and salads	no
1223 Köttbuljong ätf.	Beef broth, ready-to-eat	1.00	1.00	1.00	1.00	1.00	x	Savoury sauces and condiments	no
1224 Hönsbuljong ätfärdig	Chicken broth, ready-to-eat	1.00	1.00	1.00	1.00	1.00	x	Savoury sauces and condiments	no
1225 Ägg konventionellt rått	Egg, conventional, raw	0.00	0.00	0.00	0.00	0.00	x	Egg and egg products	no
1226 Äggula konventionellt	Egg yolk, conventional	0.00	0.00	0.00	0.00	0.00	x	Egg and egg products	no
1227 Äggvita	Egg white	0.00	0.00	0.00	0.00	0.00	x	Egg and egg products	no
1229 Fransk omelett m. grädde	French omelette with cream	0.00	0.04	0.07	0.09	0.20	no	Egg dishes	no
1230 Fransk omelett vatten	French omelette with water	0.00	0.06	0.11	0.13	0.31	no	Egg dishes	no
1233 Ägg konventionellt stekt	Egg, conventional, panfried	0.00	0.04	0.08	0.09	0.21	x	Egg dishes	no
1234 Äggröra m mjöl	Scrambled eggs with flour	0.00	0.10	0.18	0.21	0.50	x	Egg dishes	no
1252 Sik urtagen varmrökt	Whitefish, gutted, hot-smoked	0.00	0.00	0.00	0.00	0.00	yes	Fish and fish products	no
1265 Ansjovis skarpsill inlagd	Anchovy (sprat), pickled	1.00	1.00	1.00	1.00	1.00	no	Fish and fish products	no
1266 Böckling varmrökt	Hot-smoked herring ("böckling" type)	0.00	0.00	0.00	0.00	0.00	yes	Fish and fish products	no
1269 Lax kallrökt	Cold-smoked salmon	0.00	0.00	0.00	0.00	0.00	yes	Fish and fish products	no
1271 Sardiner i olja konserv	Sardines in oil, canned	0.00	0.00	0.00	0.00	0.00	yes	Fish and fish products	no
1272 Sardiner i tomatsås konserv	Sardines in tomato sauce, canned	0.00	0.00	0.00	0.00	0.00	yes	Fish and fish products	no
1276 Tonfisk i olja konserv avrunnen	Tuna in oil, canned, drained	0.00	0.00	0.00	0.00	0.00	yes	Fish and fish products	no

1278 Tonfisk i vatten konserv	Tuna in water, canned	0.00	0.00	0.00	0.00	0.00	yes	Fish and fish products	no
1281 Matjessill konserv	Matjes herring, canned	0.00	1.00	1.00	1.00	1.00	no	Fish and fish products	no
1285 Lax gravad	Gravlax (cured salmon)	0.00	0.00	0.00	0.00	0.00	yes	Fish and fish products	no
1286 Lax rimmad	Lightly salted salmon	0.00	0.00	0.00	0.00	0.00	no	Fish and fish products	no
1288 Lax urtagen varmrökt	Salmon, gutted, hot-smoked	0.00	0.00	0.00	0.00	0.00	yes	Fish and fish products	no
1289 Makrillfilé varmrökt	Mackerel fillet, hot-smoked	0.00	0.00	0.00	0.00	0.00	yes	Fish and fish products	no
1294 Fiskpinnar stekta	Fish fingers, panfried	0.00	1.00	1.00	1.00	1.00	yes	Fish dishes	no
1296 Makrillfilé konserv i tomatsås	Mackerel fillet in tomato sauce, canned	0.00	0.00	0.00	0.00	0.00	yes	Fish and fish products	no
1297 Surströmming	Fermented herring ("surströmming", local specialty)	0.00	0.00	0.00	0.00	0.00	no	Fish and fish products	no
1298 Lutfisk kokt	Lutefisk, cooked	0.00	0.00	0.00	0.00	0.00	yes	Fish and fish products	no
1299 Salt sill panerad stekt hemlagad	Salted herring, breaded and panfried, homemade	0.00	0.10	0.10	0.10	0.25	no	Fish dishes	no
1303 Ål ugnsstekt hemlagad	Eel, oven-baked, homemade	0.00	0.00	0.00	0.00	0.00	yes	Fish and fish products	no
1306 Fisk friterad	Fish, deep-fried	0.00	1.00	1.00	1.00	1.00	yes	Fish and fish products	no
1307 Fiskpanetter fiskpinnar stekta	Breaded fish patties / fish fingers, panfried	0.00	1.00	1.00	1.00	1.00	yes	Fish dishes	no
1316 Lax stekt	Salmon, panfried	0.00	0.00	0.00	0.00	0.00	yes	Fish and fish products	no
1317 Lax kokt	Salmon, boiled	0.00	0.00	0.00	0.00	0.00	yes	Fish and fish products	no
1319 Makrill panerad stekt	Mackerel, breaded and panfried	0.00	0.03	0.05	0.06	0.13	yes	Fish and fish products	no
1320 Makrill opanerad stekt	Mackerel, unbreaded and panfried	0.00	0.00	0.00	0.00	0.00	yes	Fish and fish products	no
1328 Sill panerad stekt	Herring, breaded and panfried	0.00	0.04	0.08	0.09	0.21	no	Fish and fish products	no
1329 Strömming kokt	Baltic herring, boiled	0.00	0.00	0.00	0.00	0.00	yes	Fish and fish products	no
1330 Strömming panerad stekt	Baltic herring, breaded and panfried	0.00	0.04	0.08	0.09	0.21	no	Fish and fish products	no
1332 Kräftströmming	Baltic herring in crayfish seasoning	0.00	0.00	0.00	0.00	0.00	no	Fish dishes	no
1333 Strömming filé stekt	Baltic herring fillet, panfried	0.00	0.00	0.00	0.00	0.00	yes	Fish and fish products	no
1340 Fisk varmrökt	Fish, hot-smoked	0.00	0.00	0.00	0.00	0.00	yes	Fish and fish products	no
1344 Fiskbullar konserv. u. buljong	Fish balls, canned, without broth	1.00	1.00	1.00	1.00	1.00	no	Fish dishes	no
1345 Fiskbullar m hummersås konserv tillagad	Fish balls with lobster sauce, canned, prepared	1.00	1.00	1.00	1.00	1.00	no	Fish dishes	no
1347 Böcklingpastej	Smoked herring pâté	1.00	1.00	1.00	1.00	1.00	no	Fish dishes	no
1348 Fiskpudding m ris hemlagad	Fish pudding with rice, homemade	0.00	0.06	0.06	0.06	0.14	no	Fish dishes	no
1351 Fiskbullar panerade stekta	Fish balls, breaded and panfried	0.43	0.50	0.55	0.58	0.77	no	Fish dishes	no
1352 Fiskbullar i buljong konserv stuvade m mjölk	Fish balls in broth, canned, creamed with milk	0.47	0.51	0.54	0.55	0.66	no	Fish dishes	no
1354 Bouillabaisse fisksoppa	Bouillabaisse fish soup	0.00	0.00	0.00	0.00	0.00	no	Fish dishes	no
1356 Fiskpaté m räkor	Fish pâté with shrimp	0.00	0.00	0.00	0.00	0.00	no	Fish dishes	no
1358 Fiskgratäng m dillsås	Fish gratin with dill sauce	0.00	0.02	0.04	0.05	1.00	no	Fish dishes	no
1362 Sillsallad	Herring salad	0.00	1.00	1.00	1.00	1.00	no	Fish dishes	no
1363 Sushi nigiri m. lax	Sushi nigiri with salmon	0.00	0.00	0.00	0.00	0.00	no	Fish dishes	no
1365 Sushi nigiri m. tonfisk	Sushi nigiri with tuna	0.00	0.00	0.00	0.00	0.00	no	Fish dishes	no
1366 Sushi rulle m. crabfish gurka	Sushi roll with crabfish and cucumber	0.08	0.08	0.08	0.08	1.00	no	Fish dishes	no
1368 Sushi olika sorter	Sushi, various types	0.00	0.00	0.00	0.00	0.00	no	Fish dishes	no
1370 Gryta fiskgryta m. tomat purjolök morötter fänkål ärter	Fish stew with tomato, leek, carrots, fennel, and peas	0.03	0.09	0.09	0.09	0.17	no	Fish dishes	no
1371 Fisk m mandel o ströbröd fryst	Fish with almonds and breadcrumbs, frozen	0.00	0.00	0.00	0.00	0.00	yes	Fish dishes	no
1372 Fiskgratäng m potatismos o dillsås fryst tillagad	Fish gratin with mashed potatoes and dill sauce, frozen and prepared	1.00	1.00	1.00	1.00	1.00	yes	Fish dishes	no
1377 Kaviar orökt	Unsmoked spreadable caviar	0.00	0.00	0.00	0.00	0.00	no	Fish and fish products	no
1378 Påläggskaviar original	Spreadable caviar, original	0.00	0.00	0.00	0.00	0.00	no	Fish and fish products	no
1379 Lättkaviar Kalles	Light spreadable caviar, Kalles brand	1.00	1.00	1.00	1.00	1.00	no	Fish and fish products	no
1381 Stenbitsrom	Lumpfish roe	1.00	1.00	1.00	1.00	1.00	no	Fish and fish products	no
1382 Löjrom saltad	Salted vendace roe	0.00	0.00	0.00	0.00	0.00	no	Fish and fish products	no
1386 Ostron	Oysters	0.00	0.00	0.00	0.00	0.00	yes	Fish and fish products	no
1390 Musslor konserv u lag	Mussels, canned without brine	0.00	0.00	0.00	0.00	0.00	yes	Fish and fish products	no
1394 Kräfta kokt	Crayfish, cooked	0.00	0.00	0.00	0.00	0.00	yes	Fish and fish products	no
1395 Räkor	Shrimp	0.00	0.00	0.00	0.00	0.00	yes	Fish and fish products	no
1396 Hummer kokt el. konserv.	Lobster, cooked or canned	0.00	0.00	0.00	0.00	0.00	yes	Fish and fish products	no
1397 Krabba kokt	Crab, cooked	0.00	0.00	0.00	0.00	0.00	yes	Fish and fish products	no
1400 Räka konserv. u. lag	Shrimp, canned without brine	0.00	0.00	0.00	0.00	0.00	yes	Fish and fish products	no
1401 Räkor kokta	Shrimp, cooked	0.00	0.00	0.00	0.00	0.00	yes	Fish and fish products	no
1402 Kräfta konserv el kokt	Crayfish, canned or cooked	0.00	1.00	1.00	1.00	1.00	yes	Fish and fish products	no
1406 Friterade räkor restaurang	Fried shrimp, restaurant-style	0.00	1.00	1.00	1.00	1.00	no	Fish dishes	no
1411 Räkstuvning	Shrimp stew	0.12	0.06	0.10	0.12	0.26	no	Fish dishes	no
1415 Fiskpastej	Fish pâté	0.00	1.00	1.00	1.00	1.00	no	Fish dishes	no

1417 Skaldjurspaté	Shellfish pâté	0.00	0.00	0.00	0.00	0.00	no	Fish dishes	no
1421 Fisksås pulver tillagad m mjölk vatten smör typ Smögen	Fish sauce powder prepared with milk, water, and butter ("Smögen" type)	1.00	1.00	1.00	1.00	1.00	no	Fish dishes	no
1422 Fiskbuljong ätf.	Fish broth, ready-to-eat	1.00	1.00	1.00	1.00	1.00	x	Savoury sauces and condiments	no
1425 Blodbröd fullkorn hårt	Blood bread, whole grain, crisp	1.00	1.00	1.00	1.00	1.00	yes	Bread	no
1427 Blodpalt	Blood dumplings	0.00	0.00	0.00	0.00	1.00	yes	Meat dish	no
1431 Blodpudding blodkorv fett 13% stekt	Blood pudding/blood sausage, 13% fat, panfried	1.00	1.00	1.00	1.00	1.00	no	Meat dish	no
1432 Blodpudding blodkorv fett 8% stekt	Blood pudding/blood sausage, 8% fat, panfried	1.00	1.00	1.00	1.00	1.00	no	Meat dish	no
1433 Paltbröd kokt	Palt bread, boiled	0.00	1.00	1.00	1.00	1.00	yes	Meat dish	no
1457 Kycklinglever stekt	Chicken liver, panfried	0.00	0.00	0.00	0.00	0.00	yes	Meat	no
1459 Nötlever ej panerad stekt	Beef liver, unbreaded, panfried	0.00	0.00	0.00	0.00	0.00	yes	Meat	no
1461 Leverstuvning hemlagad	Liver stew, homemade	0.02	0.07	0.07	0.07	0.15	yes	Meat dish	no
1463 Korvkaka leverkaka	Sausage cake/liver cake	0.00	0.00	0.00	0.00	0.00	yes	Meat dish	no
1464 Kycklinglever stuvad	Chicken liver, creamed	0.02	0.07	0.07	0.07	0.15	yes	Meat dish	no
1474 Leverpastej bredbar fett ca 24%	Spreadable liver pâté, approx. 24% fat	1.00	1.00	1.00	1.00	1.00	no	Meat	no
1475 Leverpastej skivbar fett 26%	Sliceable liver pâté, 26% fat	1.00	1.00	1.00	1.00	1.00	no	Meat	no
1477 Leverpastej bredbar fett ca 10%	Spreadable liver pâté, approx. 10% fat	1.00	1.00	1.00	1.00	1.00	yes	Meat	no
1479 Leverbiff mald lever stekt hemlagad	Liver patty, ground liver, panfried, homemade	0.00	0.00	0.00	0.00	0.00	yes	Meat	no
1480 Paté på köttfärs kycklinglever	Pâté made from minced meat and chicken liver	0.00	0.00	0.00	0.00	0.00	no	Meat dish	no
1483 Grönpepparpaté	Green pepper pâté	0.00	1.00	1.00	1.00	1.00	no	Meat dish	no
1484 Lever stekt	Liver, panfried	0.00	0.00	0.00	0.00	0.00	yes	Meat	no
1491 Korv kött 48% kokt typ fläskkorv	Sausage, 48% meat, boiled (e.g., pork sausage)	1.00	1.00	1.00	1.00	1.00	no	Meat	no
1496 Medvurst fett ca 23% kokt	Cooked medwurst, approx. 23% fat	1.00	1.00	1.00	1.00	1.00	no	Meat	no
1497 Rökt medvurst fett ca 36%	Smoked medwurst, approx. 36% fat	1.00	1.00	1.00	1.00	1.00	no	Meat	no
1498 Påläggskorv salami fett 34-44%	Cold cut salami, 34–44% fat	1.00	1.00	1.00	1.00	1.00	no	Meat	no
1513 Korv kött 73% stekt typ Chorizo	Sausage, 73% meat, panfried (e.g., chorizo type)	1.00	1.00	1.00	1.00	1.00	no	Meat	no
1517 Salami importerad fett 45%	Imported salami, 45% fat	0.00	1.00	1.00	1.00	1.00	no	Meat	no
1522 Värmlandskorv fett ca 18% kokt	Värmland sausage (local specialty), approx. 18% fat, boiled	1.00	1.00	1.00	1.00	1.00	no	Meat	no
1523 Bacon magert gris rökt stekt	Lean pork bacon, smoked and panfried	1.00	1.00	1.00	1.00	1.00	no	Meat	no
1526 Frukostkorv fett ca 24% kokt	Breakfast sausage, approx. 24% fat, boiled	1.00	1.00	1.00	1.00	1.00	no	Meat	no
1528 Falukorv i skivor kokt	Falu sausage (type of emulsified sausage, local specialty), sliced and boiled	1.00	1.00	1.00	1.00	1.00	no	Meat	no
1529 Falukorv stekt	Falu sausage (type of emulsified sausage, local specialty), panfried	1.00	1.00	1.00	1.00	1.00	no	Meat	no
1530 Frukostkorv stekt	Breakfast sausage, panfried	1.00	1.00	1.00	1.00	1.00	no	Meat	no
1531 Korv fermenterad kött 59% stekt typ isterband	Fermented sausage, 59% meat, fried (e.g., isterband)	1.00	1.00	1.00	1.00	1.00	no	Meat	no
1532 Stångkorv stekt	Coarse meat sausage (local specialty), panfried	1.00	1.00	1.00	1.00	1.00	no	Meat	no
1534 Korv varmkorv kokt	Hot dog, boiled	1.00	1.00	1.00	1.00	1.00	no	Meat	no
1535 Varmkorv stekt	Hot dog, panfried	1.00	1.00	1.00	1.00	1.00	no	Meat	no
1541 Pölsa värmd stekt	Pölsa (Swedish hash), heated and panfried	1.00	1.00	1.00	1.00	1.00	no	Meat	no
1542 Korvpålägg ospec	Sausage cold cuts, unspecified	1.00	1.00	1.00	1.00	1.00	no	Meat	no
1553 Kalvsylta	Jellied veal	1.00	1.00	1.00	1.00	1.00	yes	Meat	no
1557 Cashewnötter rostade u. salt	Roasted cashew nuts, unsalted	0.00	0.00	0.00	0.00	0.00	yes	Nuts and legumes	no
1558 Hasselnötter	Hazelnuts	0.00	0.00	0.00	0.00	0.00	yes	Nuts and legumes	no
1559 Jordnötssmör	Peanut butter	0.00	0.00	0.00	0.00	1.00	x	Nuts and legumes	no
1560 Jordnötter torkade	Dried peanuts	0.00	0.00	0.00	0.00	0.00	yes	Nuts and legumes	no
1562 Jordnötter rostade saltade	Roasted salted peanuts	0.00	0.00	0.00	0.00	0.00	x	Nuts and legumes	no
1564 Kokosflingor torkade	Dried coconut flakes	0.00	0.00	0.00	0.00	0.00	yes	Nuts and legumes	no
1566 Kokosmjölk fett ca 6%	Coconut milk, approx. 6% fat	0.00	1.00	1.00	1.00	1.00	x	Juices	no
1567 Linfrö hela	Whole flaxseeds	0.00	0.00	0.00	0.00	0.00	yes	Nuts and legumes	no
1568 Paranötter	Brazil nuts	0.00	0.00	0.00	0.00	0.00	yes	Nuts and legumes	no
1569 Pekannötter	Pecans	0.00	0.00	0.00	0.00	0.00	yes	Nuts and legumes	no
1570 Pistaschnötter	Pistachios	0.00	0.00	0.00	0.00	0.00	yes	Nuts and legumes	no
1571 Pumpafrö	Pumpkin seeds	0.00	0.00	0.00	0.00	0.00	yes	Nuts and legumes	no
1573 Sesamfrön torkade u skal	Dried sesame seeds, without shell	0.00	0.00	0.00	0.00	0.00	yes	Nuts and legumes	no
1574 Solrosfrö	Sunflower seeds	0.00	0.00	0.00	0.00	0.00	yes	Nuts and legumes	no
1575 Sötmandel torkad	Sweet almonds, dried	0.00	0.00	0.00	0.00	0.00	yes	Nuts and legumes	no
1576 Valnötter	Walnuts	0.00	0.00	0.00	0.00	0.00	yes	Nuts and legumes	no
1577 Kastanjer rostade	Roasted chestnuts	0.00	0.00	0.00	0.00	0.00	yes	Nuts and legumes	no
1579 Torkade saltade kikärter snacks	Dried salted chickpeas, snack	0.00	0.00	0.00	0.00	0.00	x	Nuts and legumes	no

1580 Jordnötsbågar jordnötsringar	Peanut puffs/peanut rings	1.00	1.00	1.00	1.00	1.00	x	Savoury snacks	no
1581 Tortilla chips	Tortilla chips	0.00	1.00	1.00	1.00	1.00	x	Savoury snacks	no
1582 Ostbågar	Cheese puffs	1.00	1.00	1.00	1.00	1.00	x	Savoury snacks	no
1583 Popcorn poppade	Popped popcorn	0.00	0.00	0.00	0.00	1.00	x	Savoury snacks	no
1584 Chips potatis m. havssalt	Potato chips with sea salt	0.00	0.00	0.00	0.00	0.00	x	Savoury snacks	no
1585 Salta pinnar	Salt sticks	1.00	1.00	1.00	1.00	1.00	x	Savoury snacks	no
1586 Popcorn mikro fett 22%	Microwave popcorn, 22% fat	0.00	0.00	0.00	0.00	1.00	x	Savoury snacks	no
1587 Chips smaksatta fett 34%	Flavored chips, 34% fat	1.00	1.00	1.00	1.00	1.00	x	Savoury snacks	no
1588 Chips light fett 25%	Light chips, 25% fat	1.00	1.00	1.00	1.00	1.00	x	Savoury snacks	no
1589 Japanmix nötter typ Exotic snacks	Japanese nut mix (e.g., Exotic Snacks brand)	1.00	1.00	1.00	1.00	1.00	x	Savoury snacks	no
1590 Kokosmjölk fett ca 24%	Coconut milk, approx. 24% fat	0.00	1.00	1.00	1.00	1.00	x	Juices	no
1594 Sött vetebröd kanelbullar hembakta	Sweet wheat buns, homemade cinnamon buns	0.00	0.00	0.00	0.00	0.00	no	Fine bakery wares	no
1599 Sött vetebröd slätt	Sweet wheat buns, plain	0.00	1.00	1.00	1.00	1.00	no	Fine bakery wares	no
1602 Fyllt vetebröd bullar längd ospec.	Filled wheat buns, unspecified type	0.00	1.00	1.00	1.00	1.00	no	Fine bakery wares	no
1603 Veteskorpor	Wheat rusks	0.00	1.00	1.00	1.00	1.00	no	Fine bakery wares	no
1604 Fullkornsskorpor osötade	Whole grain rusks, unsweetened	0.00	1.00	1.00	1.00	1.00	no	Fine bakery wares	no
1605 Mandelkubb	Almond cookie	0.00	1.00	1.00	1.00	1.00	no	Fine bakery wares	no
1606 Munkar u fyllning	Donuts without filling	0.00	1.00	1.00	1.00	1.00	no	Fine bakery wares	no
1607 Munkar fyllda m äppelmos vaniljkräm	Donuts filled with apple sauce and vanilla cream	0.00	1.00	1.00	1.00	1.00	no	Fine bakery wares	no
1608 Wienerbröd fyllt m vaniljkräm sylt florsocker	Danish pastry filled with vanilla cream, jam, and icing sugar	0.00	1.00	1.00	1.00	1.00	no	Fine bakery wares	no
1610 Saffransbröd	Saffron bread, sweet	0.00	1.00	1.00	1.00	1.00	no	Fine bakery wares	no
1612 Wafers fyllda rån kex	Filled wafer biscuits	1.00	1.00	1.00	1.00	1.00	no	Fine bakery wares	no
1613 Kex fullkorn Hob-nobs	Whole grain biscuits (Hob-nobs)	1.00	1.00	1.00	1.00	1.00	no	Fine bakery wares	no
1614 Kex Marie	Marie biscuits	1.00	1.00	1.00	1.00	1.00	no	Fine bakery wares	no
1615 Kex Brago	Brago biscuits	1.00	1.00	1.00	1.00	1.00	no	Fine bakery wares	no
1616 Kex Singoalla	Singoalla biscuits	1.00	1.00	1.00	1.00	1.00	no	Fine bakery wares	no
1617 Kex Ballerina	Ballerina biscuits	1.00	1.00	1.00	1.00	1.00	no	Fine bakery wares	no
1618 Maräng m. choklad	Meringue with chocolate	0.48	0.48	0.48	0.48	1.00	x	Sugar and confectionery	no
1619 Småkakor ospec.	Cookies, unspecified	0.00	1.00	1.00	1.00	1.00	no	Fine bakery wares	no
1621 Amerikanska cookies choklad	American-style chocolate cookies	1.00	1.00	1.00	1.00	1.00	no	Fine bakery wares	no
1623 Smörgåskex	Savoury crackers	1.00	1.00	1.00	1.00	1.00	no	Fine bakery wares	no
1624 Pepparkakor	Gingerbread cookies	1.00	1.00	1.00	1.00	1.00	no	Fine bakery wares	no
1625 Spettekaka	Spettekaka (local spit roasted pastry specialty)	0.00	0.00	0.00	0.00	1.00	no	Fine bakery wares	no
1626 Maräng	Meringue	0.00	1.00	1.00	1.00	1.00	no	Sugar and confectionery	no
1627 Fyllda kex	Filled biscuits	1.00	1.00	1.00	1.00	1.00	no	Fine bakery wares	no
1629 Kex salta	Salted crackers	1.00	1.00	1.00	1.00	1.00	no	Fine bakery wares	no
1633 Smörgåsrån	Sandwich wafers	1.00	1.00	1.00	1.00	1.00	no	Fine bakery wares	no
1636 Våffelstrut glasstrut ofylld	Ice cream cone, unfilled	1.00	1.00	1.00	1.00	1.00	no	Fine bakery wares	no
1638 Mandelbiskvier	Almond macaroons	1.00	1.00	1.00	1.00	1.00	no	Fine bakery wares	no
1640 Smördeg gräddad	Baked puff pastry	0.00	1.00	1.00	1.00	1.00	x	Fine bakery wares	no
1645 Gräddtårta	Cream cake	0.00	0.00	0.00	0.00	1.00	no	Fine bakery wares	no
1647 Bärtårta grädde vaniljkräm sylt gele	Berry cake with cream, vanilla custard, jam, and jelly	0.01	0.07	0.07	0.07	1.00	no	Fine bakery wares	no
1648 Prinsesstårta fylld m grädde vaniljkräm hallonsylt marsipan	Princess cake filled with cream, vanilla custard, raspberry jam, and marzipan	0.18	1.00	1.00	1.00	1.00	no	Fine bakery wares	no
1651 Frukttårta hallonsylt vaniljkräm mandelmassa frukter gele	Fruit cake with raspberry jam, vanilla custard, almond paste, fruits, and jelly	0.26	1.00	1.00	1.00	1.00	no	Fine bakery wares	no
1652 Chokladbiskvi mandelbotten smörkräm choklad	Chocolate biscuit with almond base and buttercream	0.66	1.00	1.00	1.00	1.00	no	Fine bakery wares	no
1653 Mazariner mördegsbotten mazarinfyllning glasyr	Mazarin tart with shortcrust base, almond filling, and icing	0.00	1.00	1.00	1.00	1.00	no	Fine bakery wares	no
1654 Drömrulltårta	Swiss roll with cacao and buttercream	0.00	0.00	0.00	0.00	1.00	no	Fine bakery wares	no
1655 Rulltårta m sylt	Swiss roll with jam	0.00	0.23	0.23	0.23	1.00	no	Fine bakery wares	no
1656 Punschrulle	Punch roll (Swedish pastry with marzipan and chocolate)	0.62	1.00	1.00	1.00	1.00	no	Fine bakery wares	no
1657 Chokladboll	Chocolate ball	0.00	1.00	1.00	1.00	1.00	no	Fine bakery wares	no
1658 Gräddtårta hembakad jordgubb banan	Homemade cream cake with strawberries and banana	0.00	0.00	0.00	0.00	0.00	no	Fine bakery wares	no
1664 Mjuk kaka med glasyr typ Ambrosiakaka	Soft cake with icing (e.g., Ambrosia cake)	0.00	0.00	0.00	0.00	1.00	no	Fine bakery wares	no
1666 Arraksboll	Arrack ball (Swedish pastry with arrack flavor)	0.00	1.00	1.00	1.00	1.00	no	Fine bakery wares	no
1668 Semla	Semla (Swedish cream bun)	0.14	1.00	1.00	1.00	1.00	no	Fine bakery wares	no
1671 Toscakaka	Tosca cake (almond caramel topping)	0.00	1.00	1.00	1.00	1.00	no	Fine bakery wares	no
1673 Chokladtårta sachertårta	Chocolate cake (e.g., Sachertorte)	0.41	1.00	1.00	1.00	1.00	x	Fine bakery wares	no
1674 Tårta bakelse konditoribit ospec.	Cake or pastry, bakery item, unspecified	0.00	1.00	1.00	1.00	1.00	x	Fine bakery wares	no
1677 Amerikanska muffins ospec	American-style muffins, unspecified	0.00	1.00	1.00	1.00	1.00	no	Fine bakery wares	no

1678	Mjuk kaka sockerkaka mjuk pepparkaka	Soft cake, sponge cake, soft gingerbread	0.00	1.00	1.00	1.00	1.00	no	Fine bakery wares	no
1680	Mjuk chokladkaka	Soft chocolate cake	0.00	0.00	0.00	0.00	1.00	x	Fine bakery wares	no
1682	Mjuk pepparkaka	Soft gingerbread cake	0.00	0.00	0.00	0.00	1.00	no	Fine bakery wares	no
1683	Mjuk nötkaka	Soft nut cake	0.00	0.00	0.00	0.00	1.00	no	Fine bakery wares	no
1685	Sockerkaka, saftig	Moist sponge cake	0.00	0.00	0.00	0.00	1.00	no	Fine bakery wares	no
1686	Kex digestive fullkorn 23%	Digestive biscuits, whole grain, 23%	1.00	1.00	1.00	1.00	1.00	no	Fine bakery wares	no
1687	Mjuk fruktkaka	Soft fruit cake	0.00	1.00	1.00	1.00	1.00	no	Fine bakery wares	no
1692	Glasstrut fett ca 15%	Ice cream cone, approx. 15% fat	1.00	1.00	1.00	1.00	1.00	no	Fine bakery wares	no
1695	Glass m chokladkex GB Sandwich	Ice cream with chocolate biscuit (GB Sandwich)	1.00	1.00	1.00	1.00	1.00	no	Dairy based desserts	no
1698	Glass lättglass fett ca 5%	Light ice cream, approx. 5% fat	1.00	1.00	1.00	1.00	1.00	no	Dairy based desserts	no
1703	Mjukglass bägare	Soft serve ice cream in a cup	1.00	1.00	1.00	1.00	1.00	no	Dairy based desserts	no
1705	Glasstårta fett 17% typ Vienetta	Ice cream cake, 17% fat (e.g., Vienetta)	1.00	1.00	1.00	1.00	1.00	no	Dairy based desserts	no
1707	Glass havreglass m. vanilji fett 13%	Oat-based vanilla ice cream, 13% fat	1.00	1.00	1.00	1.00	1.00	no	Dairy based desserts	no
1708	Glass parfait	Parfait ice cream	0.00	0.00	0.00	0.00	0.00	no	Dairy based desserts	no
1712	Matlagningsbas m. havre fett 13%	Cooking base with oats, 13% fat	1.00	1.00	1.00	1.00	1.00	no	Meat and dairy imitations	no
1713	Gräddfil fett 12%	Sour cream, 12% fat	0.00	0.00	0.00	0.00	0.00	no	Milk, yoghurt and similar	no
1714	Kaffegrädde fett 12%	Coffee cream, 12% fat	0.00	0.00	0.00	0.00	0.00	no	Milk, yoghurt and similar	no
1715	Vispgrädde fett 40%	Whipping cream, 40% fat	0.00	0.00	0.00	0.00	0.00	no	Milk, yoghurt and similar	no
1716	Mellangrädde fett 27%	Medium cream, 27% fat	1.00	1.00	1.00	1.00	1.00	no	Milk, yoghurt and similar	no
1717	Matlagningsgrädde fett 15%	Cooking cream, 15% fat	0.00	1.00	1.00	1.00	1.00	no	Milk, yoghurt and similar	no
1718	Matlagningsgrädde fett 5% typ Laga lätt	Cooking cream, 5% fat (e.g., Laga Lätt)	1.00	1.00	1.00	1.00	1.00	yes	Milk, yoghurt and similar	no
1719	Crème fraiche fett 34%	Crème fraîche, 34% fat	0.00	0.00	0.00	0.00	0.00	no	Milk, yoghurt and similar	no
1721	Crème fraiche lätt fett 15%	Light crème fraîche, 15% fat	1.00	1.00	1.00	1.00	1.00	no	Milk, yoghurt and similar	no
1726	Gräddfilssås med kryddgrönt	Sour cream sauce with herbs	0.00	0.00	0.00	0.00	0.00	no	Savoury sauces and condiments	no
1734	Blåbärssoppa hemlagad	Homemade blueberry soup	0.00	0.00	0.00	0.00	0.00	x	Fruit products	no
1736	Fruktsoppa blandad torkad fruk	Fruit soup with mixed dried fruits	0.00	0.00	0.00	0.00	0.00	x	Fruit products	no
1739	Nyponsoppa hemlagad	Homemade rosehip soup	0.00	0.00	0.00	0.00	0.00	x	Fruit products	no
1742	Äppelkräm	Apple compote	0.00	1.00	1.00	1.00	1.00	x	Fruit products	no
1744	Kräm bär frukt ospec.	Berry/fruit compote, unspecified	0.00	1.00	1.00	1.00	1.00	x	Fruit products	no
1748	Saftsås	Fruit syrup sauce	1.00	1.00	1.00	1.00	1.00	x	Fruit products	no
1749	Fruktdryck blåbär	Blueberry fruit drink	1.00	1.00	1.00	1.00	1.00	x	Sugar sweetened beverages	yes
1750	Fruktsoppa ätf blandad torkad frukt pastöriserad	Fruit soup, ready-to-eat, mixed dried fruit, pasteurized	1.00	1.00	1.00	1.00	1.00	x	Fruit products	no
1751	Nyponsoppa ätf pastöriserad el på pulver berik	Rosehip soup, ready-to-eat, pasteurized or powdered, fortified	1.00	1.00	1.00	1.00	1.00	x	Fruit products	no
1754	Saftkräm pastöriserad berik	Fruit syrup cream, pasteurized and fortified	1.00	1.00	1.00	1.00	1.00	x	Fruit products	no
1756	Bär- fruktkräm bär- fruktsoppa sötad m sorbitol	Berry/fruit compote or soup sweetened with sorbitol	1.00	1.00	1.00	1.00	1.00	x	Fruit products	no
1759	Kalvdans mjölkpulver ägg	Calf's dance (Swedish milk pudding) with milk powder and egg	0.00	0.00	0.00	0.00	0.00	no	Fruit products	no
1761	Marängsviss hovdessert	Eaton's mess	0.00	0.53	0.53	0.53	1.00	x	Dairy based desserts	no
1762	Brylépudding	Crème brûlée	1.00	1.00	1.00	1.00	1.00	x	Dairy based desserts	no
1764	Chokladpudding	Chocolate pudding	1.00	1.00	1.00	1.00	1.00	x	Dairy based desserts	no
1768	Chokladsås	Chocolate sauce	0.00	1.00	1.00	1.00	1.00	x	Savoury sauces and condiments	no
1769	Kolasås	Caramel sauce	0.00	1.00	1.00	1.00	1.00	x	Savoury sauces and condiments	no
1771	Vaniljsås m grädde mjölk	Vanilla sauce with cream and milk	0.00	1.00	1.00	1.00	1.00	x	Savoury sauces and condiments	no
1773	Ostkaka lätt osockrad fett 3%	Low-fat unsweetened cheesecake, 3% fat	1.00	1.00	1.00	1.00	1.00	x	Dairy based desserts	no
1775	Citronfromage	Lemon mousse	1.00	1.00	1.00	1.00	1.00	x	Dairy based desserts	no
1776	Vaniljsås m. havre fett 11%	Vanilla sauce, oat based, 11% fat	1.00	1.00	1.00	1.00	1.00	x	Savoury sauces and condiments	no
1781	Rabarberpaj	Rhubarb pie	0.00	0.00	0.00	0.00	0.00	x	Fine bakery wares	no
1782	Äppelpaj	Apple pie	0.00	0.00	0.00	0.00	1.00	x	Fine bakery wares	no
1784	Cheesecake m botten av digestivekex	Cheesecake with digestive biscuit base	0.18	0.64	0.66	0.67	1.00	x	Dairy based desserts	no
1787	Paj efterrätt ospec	Sweet pie, unspecified	0.00	0.00	0.00	0.00	1.00	x	Fine bakery wares	no
1789	Friterad äppelpaj	Deep-fried apple pie	1.00	1.00	1.00	1.00	1.00	x	Fine bakery wares	no
1790	Friterad banan	Deep-fried banana	0.00	0.00	0.00	0.00	0.00	x	Sugar and confectionery	no
1791	Ugnsstekt äpple	Oven-baked apple	0.00	0.00	0.00	0.00	0.00	x	Fruit products	no
1793	Apelsinmarmelad	Orange marmalade	0.00	1.00	1.00	1.00	1.00	x	Fruit products	no
1794	Gelé röda vinbär	Red currant jelly	0.00	1.00	1.00	1.00	1.00	x	Fruit products	no
1795	Svartvinbärsgelé	Black currant jelly	0.00	1.00	1.00	1.00	1.00	x	Fruit products	no
1796	Apelsinmarmelad lättsockrad	Lightly sweetened orange marmalade	0.00	1.00	1.00	1.00	1.00	x	Fruit products	no
1797	Marmelad aprikos björnbär jordgubbar	Apricot, blackberry, and strawberry marmalade	0.00	1.00	1.00	1.00	1.00	x	Fruit products	no
1798	Lingonsylt	Lingonberry jam	0.00	1.00	1.00	1.00	1.00	x	Fruit products	no
1799	Sylt	Jam	0.00	1.00	1.00	1.00	1.00	x	Fruit products	no
1800	Lingonsylt sötningsm.	Lingonberry jam with sweetener	1.00	1.00	1.00	1.00	1.00	x	Fruit products	no

1801 Jordgubbssylt	Strawberry jam	0.00	1.00	1.00	1.00	1.00	x	Fruit products	no
1802 Björnbärssylt	Blackberry jam	0.00	1.00	1.00	1.00	1.00	x	Fruit products	no
1803 Blåbärssylt	Blueberry jam	0.00	1.00	1.00	1.00	1.00	x	Fruit products	no
1804 Hallonsylt	Raspberry jam	0.00	1.00	1.00	1.00	1.00	x	Fruit products	no
1805 Hjortronsylt	Cloudberry jam	0.00	1.00	1.00	1.00	1.00	x	Fruit products	no
1807 Sylt osötad el. m. sötningsm.	Unsweetened jam or jam with sweetener	0.00	1.00	1.00	1.00	1.00	x	Fruit products	no
1809 Äppelmos	Applesauce	0.00	0.50	0.50	0.50	1.00	x	Fruit products	no
1810 Äppelmos lättsockrad	Lightly sweetened applesauce	0.00	1.00	1.00	1.00	1.00	x	Fruit products	no
1811 Äppelmos osötad el. sötningsm.	Unsweetened applesauce or with sweetener	0.00	1.00	1.00	1.00	1.00	x	Fruit products	no
1812 Cumberlandsås	Cumberland sauce	1.00	1.00	1.00	1.00	1.00	no	Savoury sauces and condiments	no
1818 Måltidsdryck drickf	Meal replacement drink (ready-to-drink)	1.00	1.00	1.00	1.00	1.00	x	Sugar sweetened beverages	yes
1820 Saft drickf	Fruit cordial (ready-to-drink)	0.00	1.00	1.00	1.00	1.00	x	Sugar sweetened beverages	yes
1828 Läsk m kolsyra	Carbonated soft drink	1.00	1.00	1.00	1.00	1.00	x	Sugar sweetened beverages	yes
1829 Coladryck	Cola drink	1.00	1.00	1.00	1.00	1.00	x	Sugar sweetened beverages	yes
1830 Lightläsk	Light soft drink	1.00	1.00	1.00	1.00	1.00	x	Water-based beverages	yes
1831 Cola light	Diet cola	1.00	1.00	1.00	1.00	1.00	x	Water-based beverages	yes
1832 Sportdryck drickf	Sports drink	1.00	1.00	1.00	1.00	1.00	x	Sugar sweetened beverages	yes
1834 Saft sötad m sötningsmedel drickf	Fruit cordial sweetened with sweetener (ready-to-drink)	1.00	1.00	1.00	1.00	1.00	x	Water-based beverages	yes
1839 Måltidsdricka äpple lingon drickf berik	Apple-lingonberry meal drink, fortified	1.00	1.00	1.00	1.00	1.00	x	Sugar sweetened beverages	yes
1843 Isglass	Ice lolly	1.00	1.00	1.00	1.00	1.00	no	Sugar and confectionery	no
1844 Sorbet	Sorbet	1.00	1.00	1.00	1.00	1.00	no	Sugar and confectionery	no
1846 Chokladdryck m. vatten	Chocolate drink with water	1.00	1.00	1.00	1.00	1.00	x	Coffee, tea and similar	yes
1847 Chokladdryck Pucko	Chocolate drink (Pucko brand)	1.00	1.00	1.00	1.00	1.00	no	Coffee, tea and similar	yes
1848 Mjölkchoklad m hackade hasselnötter Schweizernöt	Milk chocolate with chopped hazelnuts	1.00	1.00	1.00	1.00	1.00	x	Sugar and confectionery	no
1849 Mjölkchoklad fylld m. mandelkrokant	Milk chocolate filled with almond brittle	1.00	1.00	1.00	1.00	1.00	x	Sugar and confectionery	no
1850 Mjölkchoklad m. mjuk kolafyllning	Milk chocolate with soft caramel filling	1.00	1.00	1.00	1.00	1.00	x	Sugar and confectionery	no
1851 Kex m. mjölkchokladöverdrag	Biscuits with milk chocolate coating	1.00	1.00	1.00	1.00	1.00	no	Sugar and confectionery	no
1852 Kex m. kola mjölkchokladöverdrag	Biscuits with caramel and milk chocolate coating	1.00	1.00	1.00	1.00	1.00	no	Sugar and confectionery	no
1853 Mjuk nougat m. kolasås jordnötter mjölkchokladöverdrag	Soft nougat with caramel sauce, peanuts, and milk chocolate coating	1.00	1.00	1.00	1.00	1.00	x	Sugar and confectionery	no
1854 Mjuk chokladmaräng m. kola mjölkchokladöverdrag	Soft chocolate meringue with caramel and milk chocolate coating	1.00	1.00	1.00	1.00	1.00	x	Sugar and confectionery	no
1858 Chokladpraliner	Chocolate pralines	1.00	1.00	1.00	1.00	1.00	x	Sugar and confectionery	no
1859 Mjuk kokos m. mjölkchokladöverdrag	Soft coconut with milk chocolate coating	1.00	1.00	1.00	1.00	1.00	x	Sugar and confectionery	no
1860 Nöt- och chokladkräm Nutella	Nut and chocolate spread (Nutella)	1.00	1.00	1.00	1.00	1.00	x	Sugar and confectionery	no
1861 Choklad u tillsatt socker	Chocolate without added sugar	1.00	1.00	1.00	1.00	1.00	x	Sugar and confectionery	no
1862 Smågodis utan choklad	Candy without chocolate	1.00	1.00	1.00	1.00	1.00	x	Sugar and confectionery	no
1863 Gelékonfekt	Jelly candy	1.00	1.00	1.00	1.00	1.00	x	Sugar and confectionery	no
1864 Karameller syrliga	Sour candies	1.00	1.00	1.00	1.00	1.00	x	Sugar and confectionery	no
1865 Kola	Toffee	1.00	1.00	1.00	1.00	1.00	x	Sugar and confectionery	no
1866 Lakritskonfekt	Liquorice confectionery	1.00	1.00	1.00	1.00	1.00	x	Sugar and confectionery	no
1867 Marmeladkonfekt	Marmalade candy	1.00	1.00	1.00	1.00	1.00	x	Sugar and confectionery	no
1868 Skumgodis	Foam candy	1.00	1.00	1.00	1.00	1.00	x	Sugar and confectionery	no
1869 Polkagris	Peppermint stick	1.00	1.00	1.00	1.00	1.00	x	Sugar and confectionery	no
1870 Tuggummi	Chewing gum	1.00	1.00	1.00	1.00	1.00	x	Sugar and confectionery	no
1872 Tabletter	Lozenges or candy tablets	1.00	1.00	1.00	1.00	1.00	x	Sugar and confectionery	no
1873 Saltlakrits	Salty liquorice	1.00	1.00	1.00	1.00	1.00	x	Sugar and confectionery	no
1874 Supersalt lakrits	Extra salty liquorice	1.00	1.00	1.00	1.00	1.00	x	Sugar and confectionery	no
1875 Chokladkola mörk m chokladöverdrag Riesen	Dark chocolate toffee with chocolate coating (Riesen)	1.00	1.00	1.00	1.00	1.00	x	Sugar and confectionery	no
1876 Kexchoklad	Chocolate-covered wafer	1.00	1.00	1.00	1.00	1.00	x	Sugar and confectionery	no
1877 Fruktkola	Fruit-flavored toffee	1.00	1.00	1.00	1.00	1.00	x	Sugar and confectionery	no
1878 Skumbilar	Foam candy cars (Swedish candy)	1.00	1.00	1.00	1.00	1.00	x	Sugar and confectionery	no
1879 Chokladboll med kokos	Chocolate ball with coconut	1.00	1.00	1.00	1.00	1.00	no	Fine bakery wares	no
1880 Mums-mums	Marshmallow treat with chocolate coating	1.00	1.00	1.00	1.00	1.00	no	Fine bakery wares	no
1881 Energibar choklad nötter Start	Energy bar with chocolate and nuts (Start brand)	1.00	1.00	1.00	1.00	1.00	x	Sugar and confectionery	no
1882 Karameller sockerfria	Sugar-free candies	1.00	1.00	1.00	1.00	1.00	x	Sugar and confectionery	no
1883 Tabletter sockerfria	Sugar-free lozenges	1.00	1.00	1.00	1.00	1.00	x	Sugar and confectionery	no
1884 Tuggummi sockerfritt	Sugar-free chewing gum	1.00	1.00	1.00	1.00	1.00	x	Sugar and confectionery	no
1885 Mandelmassa	Almond paste	1.00	1.00	1.00	1.00	1.00	x	Sugar and confectionery	no
1886 Kakaopulver fett 20-22%	Cocoa powder, 20–22% fat	0.00	0.00	0.00	0.00	0.00	x	Coffee, tea and similar	no
1887 Chokladdryckspulver m. socker fett 2,5%	Chocolate drink powder with sugar, 2.5% fat	1.00	1.00	1.00	1.00	1.00	x	Coffee, tea and similar	no

1888	Chokladbollar	Chocolate balls	0.00	1.00	1.00	1.00	1.00	no	Fine bakery wares	no
1889	Godis inkl choklad	Candy including chocolate	1.00	1.00	1.00	1.00	1.00	x	Sugar and confectionery	no
1892	Socker	Sugar	0.00	0.00	0.00	0.00	0.00	x	Sugar and confectionery	no
1893	Brun farin	Brown sugar	1.00	1.00	1.00	1.00	1.00	x	Sugar and confectionery	no
1894	Ljus sirap	Light syrup	1.00	1.00	1.00	1.00	1.00	x	Sugar and confectionery	no
1895	Godis från hälsokostaffärer torkad frukt nötter m el u överdrag av yoghurt choklad	Health store candy: dried fruit and nuts with or without yoghurt/chocolate coating	0.00	1.00	1.00	1.00	1.00	x	Sugar and confectionery	no
1896	Honung	Honey	0.00	0.00	0.00	0.00	0.00	x	Sugar and confectionery	no
1897	Druvsocker	Glucose	1.00	1.00	1.00	1.00	1.00	x	Sugar and confectionery	no
1900	Svagdricka vol. % 2,3	Low-alcohol malt beverage, 2.3% vol	1.00	1.00	1.00	1.00	1.00	x	Alcoholic beverages	yes
1901	Öl lättöl vol. % 2,3	Light beer, 2.3% vol	0.00	0.00	0.00	0.00	1.00	x	Alcoholic beverages	yes
1902	Öl pilsner vol. % 3,5	Pilsner beer, 3.5% vol	0.00	1.00	1.00	1.00	1.00	x	Alcoholic beverages	yes
1903	Cider vol. % 1	Cider, 1% vol	1.00	1.00	1.00	1.00	1.00	x	Alcoholic beverages	yes
1904	Vitt vin vol. % 1	White wine, 1% vol	1.00	1.00	1.00	1.00	1.00	x	Alcoholic beverages	yes
1906	Öl starköl el exportöl vol. % 5,4	Strong or export beer, 5.4% vol	0.00	0.00	0.00	0.00	0.00	x	Alcoholic beverages	yes
1907	Rödvin vol. % 14	Red wine, 14% vol	0.00	0.00	0.00	0.00	0.00	x	Alcoholic beverages	yes
1908	Vitt vin vol. % 12	White wine, 12% vol	0.00	0.00	0.00	0.00	0.00	x	Alcoholic beverages	yes
1912	Vinglögg vol. % 10	Mulled wine, 10% vol	0.20	1.00	1.00	1.00	1.00	x	Alcoholic beverages	yes
1916	Sherry halvtorr vol. % 17	Semi-dry sherry, 17% vol	1.00	1.00	1.00	1.00	1.00	x	Alcoholic beverages	yes
1917	Starkvinsglögg vol. % 16	Strong mulled wine, 16% vol	1.00	1.00	1.00	1.00	1.00	x	Alcoholic beverages	yes
1918	Brännvin renat el vodka vol. % 40	Brännvin or vodka, 40% vol	1.00	1.00	1.00	1.00	1.00	x	Alcoholic beverages	yes
1919	Likör söt vol. % 24	Sweet liqueur, 24% vol	1.00	1.00	1.00	1.00	1.00	x	Alcoholic beverages	yes
1921	Konjak el brandy vol. % 40	Cognac or brandy, 40% vol	1.00	1.00	1.00	1.00	1.00	x	Alcoholic beverages	yes
1922	Punsch vol. % 26	Swedish punch, 26% vol	1.00	1.00	1.00	1.00	1.00	x	Alcoholic beverages	yes
1923	Rom vol. % 40	Rum, 40% vol	1.00	1.00	1.00	1.00	1.00	x	Alcoholic beverages	yes
1925	Gin vol. % 40	Gin, 40% vol	1.00	1.00	1.00	1.00	1.00	x	Alcoholic beverages	yes
1926	Whisky vol. % 40	Whisky, 40% vol	1.00	1.00	1.00	1.00	1.00	x	Alcoholic beverages	yes
1929	Irish coffee	Irish coffee	0.67	0.67	0.67	0.67	0.67	x	Alcoholic beverages	yes
1951	Mineralvatten	Mineral water	0.00	1.00	1.00	1.00	1.00	x	Water-based beverages	yes
1953	Kranvatten	Tap water	0.00	0.00	0.00	0.00	0.00	x	Water-based beverages	yes
1954	Källvatten förpackat	Bottled spring water	0.00	0.00	0.00	0.00	0.00	x	Water-based beverages	yes
1955	Snabbkaffe pulver	Instant coffee powder	0.00	0.00	0.00	0.00	0.00	x	Coffee, tea and similar	no
1956	Snabbkaffe drickf	Instant coffee, ready-to-drink	0.00	0.00	0.00	0.00	0.00	x	Coffee, tea and similar	yes
1957	Kaffe bryggt	Brewed coffee	0.00	0.00	0.00	0.00	0.00	x	Coffee, tea and similar	yes
1958	Bryggt kaffe espresso drickf	Brewed espresso coffee, ready-to-drink	0.00	0.00	0.00	0.00	0.00	x	Coffee, tea and similar	yes
1959	Snabbkaffe koffeinfritt drickf	Decaffeinated instant coffee, ready-to-drink	0.00	0.00	0.00	0.00	0.00	x	Coffee, tea and similar	yes
1962	Te bryggt	Brewed tea	0.00	0.00	0.00	0.00	1.00	x	Coffee, tea and similar	yes
1963	Örtte drickf.	Herbal tea, ready-to-drink	0.00	0.00	0.00	0.00	0.00	x	Coffee, tea and similar	yes
1964	Nyponte drickf. m. svarta vinbär	Rosehip tea with blackcurrant, ready-to-drink	0.00	0.00	0.00	0.00	0.00	x	Coffee, tea and similar	yes
1966	Vinäger ättiksyra 7 %	Vinegar, acetic acid 7%	0.00	0.00	0.00	0.00	0.00	x	Savoury sauces and condiments	no
1967	Äppelcidervinäger ättiksyra 7 %	Apple cider vinegar, acetic acid 7%	0.00	0.00	0.00	0.00	0.00	x	Savoury sauces and condiments	no
1968	Chilisås tomat	Tomato chili sauce	1.00	1.00	1.00	1.00	1.00	no	Savoury sauces and condiments	no
1969	Ketchup	Ketchup	1.00	1.00	1.00	1.00	1.00	no	Savoury sauces and condiments	no
1970	HP-sås	HP sauce	1.00	1.00	1.00	1.00	1.00	no	Savoury sauces and condiments	no
1972	Senap svensk	Swedish mustard	0.00	0.00	0.00	0.00	1.00	no	Savoury sauces and condiments	no
1973	Senap fransk	French mustard	0.00	0.00	0.00	0.00	1.00	yes	Savoury sauces and condiments	no
1974	Sötsur sås	Sweet and sour sauce	1.00	1.00	1.00	1.00	1.00	no	Savoury sauces and condiments	no
1985	Béchamelsås	Béchamel sauce	0.00	0.09	0.09	0.09	1.00	no	Savoury sauces and condiments	no
1987	Ljus sås buljong	Light broth-based sauce	0.04	0.09	0.13	0.15	1.00	no	Savoury sauces and condiments	no
1989	Ostsås ädelost	Cheese sauce with blue cheese	0.00	1.00	1.00	1.00	1.00	no	Savoury sauces and condiments	no
1991	Brunsås mjölk	Brown sauce with milk	0.00	1.00	1.00	1.00	1.00	no	Savoury sauces and condiments	no
1992	Sky	Gravy	0.00	0.22	0.22	0.22	0.22	yes	Savoury sauces and condiments	no
1995	Currysås	Curry sauce	0.00	1.00	1.00	1.00	1.00	no	Savoury sauces and condiments	no
1996	Brunsås kaffegrädde	Brown sauce with coffee cream	0.12	0.18	0.18	0.18	0.18	no	Savoury sauces and condiments	no
1998	Brunsås pulver tillagad m mjölk vatten	Brown sauce powder prepared with milk and water	1.00	1.00	1.00	1.00	1.00	no	Savoury sauces and condiments	no
1999	Gräddsås pulver tillagad m mjölk vatten smör	Cream sauce powder prepared with milk, water, and butter	1.00	1.00	1.00	1.00	1.00	no	Savoury sauces and condiments	no
2000	Tomatsås	Tomato sauce	0.00	0.50	0.50	0.50	1.00	yes	Vegetables and vegetable products	no
2002	Äggsås m persilja	Egg sauce with parsley	0.03	0.07	0.10	0.12	0.23	no	Savoury sauces and condiments	no
2004	Pestosås m basilika pinjenötter cashewnötter	Pesto sauce with basil, pine nuts, and cashews	0.00	1.00	1.00	1.00	1.00	no	Savoury sauces and condiments	no
2007	Sweet chilisås	Sweet chili sauce	1.00	1.00	1.00	1.00	1.00	no	Savoury sauces and condiments	no

2009	Pastasås olika märken	Pasta sauce, various brands	0.00	1.00	1.00	1.00	1.00	no	Vegetables and vegetable products	no
2010	Sweet&sour sås Uncle Ben	Sweet & sour sauce (Uncle Ben's)	1.00	1.00	1.00	1.00	1.00	no	Savoury sauces and condiments	no
2012	Teriyakisås	Teriyaki sauce	1.00	1.00	1.00	1.00	1.00	no	Savoury sauces and condiments	no
2025	Frukostflingor fullkorn müsli Bär	Whole grain breakfast cereal/muesli with berries	0.00	1.00	1.00	1.00	1.00	yes	Breakfast cereals	no
2027	Frukostflingor fullkorn frukt nötter kokosolja socker typ Start hasselnöt	Whole grain cereal with fruit, nuts, coconut oil, sugar (e.g., Start hazelnut)	1.00	1.00	1.00	1.00	1.00	no	Breakfast cereals	no
2028	Frukostflingor fullkorn frukt nötter kokosolja socker crunchy russin	Whole grain cereal with fruit, nuts, coconut oil, sugar, crunchy raisins	1.00	1.00	1.00	1.00	1.00	no	Breakfast cereals	no
2029	Frukostflingor frukt nötter/kokos olja socker: ICA Crunchy naturell	Cereal with fruit, nuts/coconut oil, sugar (ICA Crunchy natural)	1.00	1.00	1.00	1.00	1.00	no	Breakfast cereals	no
2031	Frukostflingor fullkorn frukt olja socker typ Signum früsli crisp jordgubb	Whole grain cereal with fruit, oil, sugar (e.g., Signum früsli crisp strawberry)	1.00	1.00	1.00	1.00	1.00	no	Breakfast cereals	no
2033	Frukostflingor fullkorn frukt olja F-müsli banan	Whole grain cereal with fruit, oil (F-müsli banana)	0.00	1.00	1.00	1.00	1.00	yes	Breakfast cereals	no
2035	Crab sticks Ullmo	Crab sticks (Ullmo brand)	1.00	1.00	1.00	1.00	1.00	no	Fish and fish products	no
2036	Matlagningsbas fett 15%	Cooking base, 15% fat	1.00	1.00	1.00	1.00	1.00	no	Milk, yoghurt and similar	no
2037	Matlagningsbas fett 7%	Cooking base, 7% fat	1.00	1.00	1.00	1.00	1.00	no	Milk, yoghurt and similar	no
2038	Milda Fraiche 24% fett	Milda Fraiche, 24% fat	1.00	1.00	1.00	1.00	1.00	no	Milk, yoghurt and similar	no
2039	Vispgrädde ersättningsprod. Mjölk olja fett 20%	Whipping cream substitute with milk and oil, 20% fat	1.00	1.00	1.00	1.00	1.00	no	Milk, yoghurt and similar	no
2040	Milda kaffe 10% fett	Milda coffee cream, 10% fat	1.00	1.00	1.00	1.00	1.00	no	Milk, yoghurt and similar	no
2041	Milda Vaniljvisp 12% fett	Milda vanilla whip, 12% fat	1.00	1.00	1.00	1.00	1.00	x	Savoury sauces and condiments	no
2043	Creme Fraiche fett 13% smaksatt	Flavored crème fraîche, 13% fat	1.00	1.00	1.00	1.00	1.00	no	Milk, yoghurt and similar	no
2044	Creme Fraiche fett 28% smaksatt	Flavored crème fraîche, 28% fat	1.00	1.00	1.00	1.00	1.00	no	Milk, yoghurt and similar	no
2046	Mini Fraiche fett 5%	Mini Fraiche, 5% fat	1.00	1.00	1.00	1.00	1.00	yes	Milk, yoghurt and similar	no
2048	Fjällfil naturell fett 4,2%	Fermented milk, plain (local specialty), 4.2% fat	0.00	0.00	0.00	0.00	0.00	no	Milk, yoghurt and similar	no
2049	Fjällyoghurt fett 3,6 % , olika smaksättningar	Yoghurt, (Fjällyoghurt, type) 3.6% fat, various flavors	1.00	1.00	1.00	1.00	1.00	no	Milk, yoghurt and similar	no
2051	Vit choklad	White chocolate	1.00	1.00	1.00	1.00	1.00	x	Sugar and confectionery	no
2052	Mörk choklad kakao >70 %	Dark chocolate, cocoa >70%	0.00	1.00	1.00	1.00	1.00	x	Sugar and confectionery	no
2053	Mörk choklad kakao < 70%	Dark chocolate, cocoa <70%	1.00	1.00	1.00	1.00	1.00	x	Sugar and confectionery	no
2055	Mjölkchoklad ospec.	Milk chocolate, unspecified	1.00	1.00	1.00	1.00	1.00	x	Sugar and confectionery	no
2058	Rostad lök	Roasted onion	0.00	0.00	0.00	0.00	0.00	no	Vegetables and vegetable products	no
2062	Knäckemacka vete olika fyllningar typ Wasa	Crispbread sandwich with wheat, various fillings (e.g., Wasa)	1.00	1.00	1.00	1.00	1.00	no	Sandwiches, pizzas and similar	no
2063	Knäckemacka fullkorn råg olika fyllningar	Crispbread sandwich with whole grain rye, various fillings	0.00	0.00	0.00	0.00	1.00	no	Sandwiches, pizzas and similar	no
2064	Falafel kikärtskroketter frysta	Frozen falafel chickpea croquettes	0.00	0.00	0.00	0.00	1.00	no	Legume dishes	no
2065	Pytt i panna m. sojaprotein veg.	Swedish hash with soy protein, vegetarian	0.33	1.00	1.00	1.00	1.00	yes	Legume dishes	no
2066	Sojakorv	Soy sausage	1.00	1.00	1.00	1.00	1.00	no	Meat and dairy imitations	no
2070	Matlagningsbas m. soja fett 17%	Cooking base with soy, 17% fat	1.00	1.00	1.00	1.00	1.00	no	Meat and dairy imitations	no
2072	Müslibar berikad typ Special K Chocolate	Fortified muesli bar (e.g., Special K Chocolate)	1.00	1.00	1.00	1.00	1.00	x	Cereals	no
2073	Jordnötssås	Peanut sauce	0.00	1.00	1.00	1.00	1.00	no	Savoury sauces and condiments	no
2074	Bordsmargarin ospec.	Table margarine, unspecified	1.00	1.00	1.00	1.00	1.00	no	Fat	no
2075	Coleslaw kall sås	Coleslaw, cold sauce	0.00	1.00	1.00	1.00	1.00	no	Savoury sauces and condiments	no
2079	Äggnudlar kokta m salt	Egg noodles, boiled with salt	0.00	0.00	0.00	0.00	0.00	no	Pasta and similar	no
2081	Kantarellstuvning	Chanterelle stew	0.00	0.06	0.10	0.12	0.29	no	Vegetable dishes	no
2083	Ratatouille	Ratatouille	0.03	0.09	0.09	0.09	0.17	no	Vegetable dishes	no
2092	Dansk sjömansbiff	Danish beef and potato casserole	0.00	0.03	0.03	0.03	0.03	no	Meat dish	no
2096	Flygande Jakob Kyckling bacon jordnötter banan	"Flying Jacob" (chicken, bacon, peanuts, banana)	0.13	0.13	0.13	0.13	1.00	no	Meat dish	no
2097	Prinskorv stekt	Fried cocktail sausage	1.00	1.00	1.00	1.00	1.00	no	Meat	no
2098	Skinkröra kall	Cold ham spread	0.25	0.53	0.53	0.53	1.00	no	Savoury sauces and condiments	no
2099	Hamburgare blandfärs stekt	Fried hamburger with mixed minced meat	0.00	0.00	0.00	0.00	1.00	no	Meat dish	no
2104	Carbonara sås	Carbonara sauce	0.33	0.33	0.33	0.33	1.00	no	Savoury sauces and condiments	no
2105	Pastagratäng med fisk eller skaldjur	Pasta gratin with fish or seafood	0.00	0.00	0.00	0.00	0.00	no	Pasta dishes	no
2106	Thaigryta fisk och skaldjur	Thai stew with fish and seafood	0.00	0.33	0.33	0.33	0.33	no	Fish dishes	no
2107	Skagenröra	Skagen mix (shrimp and mayo salad)	0.00	1.00	1.00	1.00	1.00	no	Savoury sauces and condiments	no
2109	Tonfiskröra mager	Tuna salad, light	0.00	0.47	0.47	0.47	1.00	no	Savoury sauces and condiments	no
2123	Havregrynsgröt fullkorn fiberhavregryn	Whole grain oatmeal with fiber oats	0.00	0.00	0.00	0.00	0.00	yes	Breakfast cereals	no
2125	Glasnudlar kokta m salt	Glass noodles, boiled with salt	0.00	0.00	0.00	0.00	0.00	no	Pasta and similar	no
2129	Morotskaka	Carrot cake	0.00	0.11	0.11	0.11	1.00	no	Fine bakery wares	no
2132	Radiokaka lördagskaka kexkaka källarkaka	Chocolate biscuit cake (traditional recipe)	0.78	0.78	0.78	0.78	0.78	x	Fine bakery wares	no
2133	Pannkaka på sojadryck	Pancake made with soy drink	0.16	0.21	0.24	0.26	0.39	no	Fine bakery wares	no
2134	Paj m. broccoli skinka	Pie with broccoli and ham	0.07	1.00	1.00	1.00	1.00	no	Sandwiches, pizzas and similar	no
2139	Pizza Hawaii restaurang	Hawaiian pizza (restaurant)	0.05	0.50	0.50	0.50	1.00	no	Sandwiches, pizzas and similar	no

2142	Ost- och skinksås	Cheese and ham sauce	0.17	0.20	0.23	0.24	1.00	no	Savoury sauces and condiments	no
2144	Tzatziki	Tzatziki	0.00	1.00	1.00	1.00	1.00	no	Savoury sauces and condiments	no
2162	Palsternacka kokt m. salt	Parsnip, boiled with salt	0.00	0.00	0.00	0.00	0.00	yes	Vegetables and vegetable products	no
2164	Fänkål kokt	Fennel, boiled	0.00	0.00	0.00	0.00	0.00	yes	Vegetables and vegetable products	no
2184	Ostkaka fett 7%	Cheesecake, 7% fat	1.00	1.00	1.00	1.00	1.00	x	Dairy based desserts	no
2189	Rapsolja	Rapeseed oil (Canola oil)	0.00	0.00	0.00	0.00	0.00	yes	Fat	no
2190	Matfettsblandning fett 75% berikad typ Bregott	Fat blend, 75% fat, fortified (e.g., Bregott)	0.00	0.00	0.00	0.00	0.00	no	Fat	no
2197	Äggröra	Scrambled eggs	0.00	0.05	0.08	0.10	0.23	x	Egg and egg products	no
2205	Ägg konventionellt kokt	Conventionally boiled egg	0.00	0.00	0.00	0.00	0.00	x	Egg and egg products	no
2223	Kex digestive fullkorn glutenfri	Gluten-free whole grain digestive biscuit	1.00	1.00	1.00	1.00	1.00	no	Fine bakery wares	no
2228	Bearnaisesås fett 8% Kelda	Bearnaise sauce, 8% fat (Kelda)	1.00	1.00	1.00	1.00	1.00	no	Savoury sauces and condiments	no
2229	Klassisk gräddsås fett 6% Kelda	Classic cream sauce, 6% fat (Kelda)	1.00	1.00	1.00	1.00	1.00	no	Savoury sauces and condiments	no
2230	Ostsås fett 8%	Cheese sauce, 8% fat	1.00	1.00	1.00	1.00	1.00	no	Savoury sauces and condiments	no
2231	Bearnaise sås typ Spisa Rydbergs	Bearnaise sauce (e.g., Spisa Rydbergs)	1.00	1.00	1.00	1.00	1.00	no	Savoury sauces and condiments	no
2232	Rödvin sås fett 1,5% Spisa Rydbergs	Red wine sauce, 1.5% fat (Spisa Rydbergs)	1.00	1.00	1.00	1.00	1.00	no	Savoury sauces and condiments	no
2233	Kebabsås vit fett 30%	White kebab sauce, 30% fat	1.00	1.00	1.00	1.00	1.00	no	Savoury sauces and condiments	no
2246	Mintplattor m. mörkchokladöverdrag	Mint thins with dark chocolate coating	1.00	1.00	1.00	1.00	1.00	x	Sugar and confectionery	no
2247	Tofutti (creamy smooth) original	Tofutti (creamy smooth) original	1.00	1.00	1.00	1.00	1.00	no	Meat and dairy imitations	no
2251	Matfettsblandning extrasaltad fett 80% berikad typ Bregott	Extra salted fat blend, 80% fat, fortified (e.g., Bregott)	0.00	0.00	0.00	0.00	0.00	no	Fat	no
2255	Mozzarella ost fett 16 %	Mozzarella cheese, 16% fat	0.00	0.00	0.00	0.00	0.00	no	Cheese	no
2256	Yoghurtdryck fett 0,5 % Yoggi Yalla!	Yoghurt drink, 0.5% fat (Yoggi Yalla!)	1.00	1.00	1.00	1.00	1.00	no	Milk, yoghurt and similar	no
2265	Ekologisk Minimjölk fett < 0,1 % berik (milko)	Organic skim milk, <0.1% fat, fortified (Milko)	0.00	0.00	0.00	0.00	0.00	yes	Milk, yoghurt and similar	yes
2272	Kapris	Capers	0.00	0.00	0.00	0.00	0.00	no	Vegetables and vegetable products	no
2294	Kebabkött	Kebab meat	1.00	1.00	1.00	1.00	1.00	no	Meat	no
2298	Dippsås	Dipping sauce	0.00	1.00	1.00	1.00	1.00	no	Savoury sauces and condiments	no
2301	Sportdryck ProViva Active berik	Sports drink (ProViva Active), fortified	1.00	1.00	1.00	1.00	1.00	x	Sugar sweetened beverages	yes
2302	Energidryck typ Red Bull berikad	Energy drink (e.g., Red Bull), fortified	1.00	1.00	1.00	1.00	1.00	x	Sugar sweetened beverages	yes
2303	Ice tea drickf	Iced tea, ready-to-drink	1.00	1.00	1.00	1.00	1.00	x	Sugar sweetened beverages	yes
2305	Alkoholfri öl	Non-alcoholic beer	0.00	1.00	1.00	1.00	1.00	x	Alcoholic beverages	yes
2306	Kanel	Cinnamon	0.00	0.00	0.00	0.00	0.00	x	Savoury sauces and condiments	no
2402	Bordsmargarin fett 60% berikad typ Carlshamn Runda Bords	Table margarine, 60% fat, fortified (e.g., Carlshamn Runda Bords)	1.00	1.00	1.00	1.00	1.00	no	Fat	no
2414	Skaldjurspaj fyllning 66%	Seafood pie, 66% filling	0.00	1.00	1.00	1.00	1.00	no	Sandwiches, pizzas and similar	no
2470	Flytande margarin fett 80% berikad typ Mjuka	Liquid margarine, 80% fat, fortified (e.g., Mjuka)	1.00	1.00	1.00	1.00	1.00	no	Fat	no
2490	Bordsmargarin fett 70% berikad typ Becel Gold	Table margarine, 70% fat, fortified (e.g., Becel Gold)	1.00	1.00	1.00	1.00	1.00	no	Fat	no
2506	Kebab m bröd sallad sås	Kebab with bread, salad, and sauce	0.39	1.00	1.00	1.00	1.00	no	Sandwiches, pizzas and similar	no
2507	Kebab special m pommes frites	Kebab special with French fries	0.78	1.00	1.00	1.00	1.00	no	Sandwiches, pizzas and similar	no
2510	Hamburgare dubbel bröd ost sallad dressing	Double hamburger with bun, cheese, salad, and dressing	0.01	1.00	1.00	1.00	1.00	no	Sandwiches, pizzas and similar	no
2511	Hamburgare enkel bröd inlagd gurka	Single hamburger with bun and pickled cucumber	0.02	1.00	1.00	1.00	1.00	no	Sandwiches, pizzas and similar	no
2513	Basmatiris kokt	Basmati rice, cooked	0.00	0.00	0.00	0.00	0.00	no	Cereals	no
2514	Jasminris kokt	Jasmine rice, cooked	0.00	0.00	0.00	0.00	0.00	no	Cereals	no
2516	Ris långkornigt parboiled kokt	Long-grain, parboiled rice, cooked	0.00	0.00	0.00	0.00	0.00	no	Cereals	no
2517	Råris fullkorn långkornigt kokt m salt	Whole grain brown rice, cooked with salt	0.00	0.00	0.00	0.00	0.00	yes	Cereals	no
2520	Pasta tagliatelle m. ägg kokt m. salt glutenfri	Gluten-free tagliatelle pasta with egg, cooked with salt	0.00	1.00	1.00	1.00	1.00	no	Pasta and similar	no
2521	Pasta makaroner spagetti kokt m. salt glutenfri	Gluten-free macaroni/spaghetti, cooked with salt	0.00	1.00	1.00	1.00	1.00	no	Pasta and similar	no
2522	Pasta kokt m. salt majs 100% glutenfri	Gluten-free corn pasta, cooked with salt	0.00	0.00	0.00	0.00	0.00	no	Pasta and similar	no
2542	Lasagne nötfärs eller blandfärs	Lasagna with ground beef or mixed meat	0.00	1.00	1.00	1.00	1.00	no	Pasta dishes	no
2548	Gryta kalops älg	Elk stew with onion and carrots (local recipe)	0.00	0.04	0.07	0.08	0.19	no	Meat dish	no
2557	Tacoskal	Taco shell	0.00	0.00	0.00	0.00	0.00	no	Savoury snacks	no
2559	Romansallat	Romaine lettuce	0.00	0.00	0.00	0.00	0.00	yes	Vegetables and vegetable products	no
2560	Ekbladssallat	Oak leaf lettuce	0.00	0.00	0.00	0.00	0.00	yes	Vegetables and vegetable products	no
2561	Ruccolasallat	Roquette lettuce	0.00	0.00	0.00	0.00	0.00	yes	Vegetables and vegetable products	no
2562	Mangold fräst	Swiss chard, sautéed	0.00	0.15	0.15	0.15	0.35	no	Vegetables and vegetable products	no
2563	Friterade lökringar	Fried onion rings	1.00	1.00	1.00	1.00	1.00	no	Savoury snacks	no
2564	Chili cheese	Chili cheese bites	1.00	1.00	1.00	1.00	1.00	no	Savoury snacks	no
2565	Okra kokt u. salt	Okra boiled without salt	0.00	0.00	0.00	0.00	0.00	yes	Vegetables and vegetable products	no
2566	Paprika marinerad	Marinated bell pepper	0.00	0.00	0.00	0.00	0.00	no	Vegetables and vegetable products	no
2569	Litchi	Lychee	0.00	0.00	0.00	0.00	0.00	yes	Fruit	no
2571	Exotisk frukt	Exotic fruit	0.00	0.00	0.00	0.00	0.00	yes	Fruit	no
2575	Kvittenmarmelad	Quince marmalade	0.00	1.00	1.00	1.00	1.00	x	Fruit products	no
2576	Mango torkad	Dried mango	0.00	0.00	0.00	0.00	0.00	x	Fruit products	no

2578 Papaya torkad med socker	Dried papaya with sugar	0.00	0.00	0.00	0.00	0.00	x	Fruit products	no
2593 Matbanan, friterad eller stekt	Plantain, fried or sautéed	0.00	0.00	0.00	0.00	0.00	x	Starchy roots and tubers	no
2598 Fruktsoppa eller saftsoppa på pulver	Fruit soup or cordial soup from powder	1.00	1.00	1.00	1.00	1.00	x	Fruit products	no
2599 Blåbärssoppa på pulver	Blueberry soup from powder	1.00	1.00	1.00	1.00	1.00	x	Fruit products	no
2600 Hjortron frysta	Frozen cloudberries	0.00	0.00	0.00	0.00	0.00	yes	Fruit	no
2602 Aprikosmarmelad	Apricot marmalade	1.00	1.00	1.00	1.00	1.00	x	Fruit products	no
2605 Blomkål stekt eller wokad	Cauliflower, fried or stir-fried	0.00	0.11	0.19	0.23	0.53	no	Vegetables and vegetable products	no
2606 Broccoli stekt eller wokad	Broccoli, fried or stir-fried	0.00	0.08	0.15	0.17	0.40	no	Vegetables and vegetable products	no
2607 Fänkål stekt eller wokad	Fennel, fried or stir-fried	0.00	0.15	0.15	0.15	0.35	no	Vegetables and vegetable products	no
2608 Morot stekt eller wokad	Carrot, fried or stir-fried	0.00	0.11	0.19	0.23	0.53	no	Vegetables and vegetable products	no
2609 Palsternacka stekt el. wokad	Parsnip, fried or stir-fried	0.00	0.04	0.08	0.10	0.22	no	Vegetables and vegetable products	no
2610 Paprika stekt grillad eller wokad	Bell pepper, fried, grilled, or stir-fried	0.00	0.12	0.22	0.26	0.60	no	Vegetables and vegetable products	no
2611 Pumpa eller squash, stekt eller wokad	Pumpkin or squash, fried or stir-fried	0.00	0.15	0.15	0.15	0.35	no	Vegetables and vegetable products	no
2612 Purjolök stekt eller wokad	Leek, fried or stir-fried	0.00	0.10	0.17	0.20	0.48	no	Vegetables and vegetable products	no
2613 Rotselleri stekt eller wokad	Celeriac, fried or stir-fried	0.00	0.09	0.17	0.20	0.46	no	Vegetables and vegetable products	no
2614 Rödbeta stekt eller wokad	Beetroot, fried or stir-fried	0.00	0.06	0.10	0.12	0.28	no	Vegetables and vegetable products	no
2615 Savojkål stekt eller wokad	Savoy cabbage, fried or stir-fried	0.00	0.15	0.15	0.15	0.35	no	Vegetables and vegetable products	no
2616 Salladskål stekt eller wokad	Lettuce cabbage, fried or stir-fried	0.00	0.15	0.15	0.15	0.35	no	Vegetables and vegetable products	no
2617 Vitkål stekt eller wokad	White cabbage, fried or stir-fried	0.00	0.15	0.15	0.15	0.35	no	Vegetables and vegetable products	no
2618 Champinjoner stekta	Fried mushrooms	0.00	0.15	0.15	0.15	0.35	no	Vegetables and vegetable products	no
2619 Kantareller stekta	Fried chanterelles	0.00	0.15	0.15	0.15	0.35	no	Vegetables and vegetable products	no
2620 Ratatouille grönsaksröra	Ratatouille vegetabledish	0.03	0.09	0.09	0.09	0.17	yes	Vegetable dishes	no
2623 Svampgryta	Mushroom stew	0.02	0.06	0.06	0.06	0.17	yes	Vegetable dishes	no
2628 Grönsaksgratäng ospec.	Vegetable gratin, unspecified	0.01	0.07	0.07	0.07	0.15	no	Vegetable dishes	no
2629 Grönsaksgratäng med skinka	Vegetable gratin with ham	0.12	0.16	0.19	0.20	0.65	no	Vegetable dishes	no
2630 Grönsaksgratäng med skaldjur eller fisk	Vegetable gratin with seafood or fish	0.01	0.07	0.07	0.07	0.15	no	Vegetable dishes	no
2631 Blomkål gratinerad	Cauliflower gratin	0.01	0.07	0.07	0.07	0.15	yes	Vegetable dishes	no
2632 Blomkål gratinerad med skinka	Cauliflower gratin with ham	0.11	0.14	0.16	0.17	0.25	no	Vegetable dishes	no
2633 Blomkål gratinerad med skaldjur eller fisk	Cauliflower gratin with seafood or fish	0.01	0.07	0.07	0.07	0.15	no	Vegetable dishes	no
2634 Broccolilåda rökt skinka	Broccoli casserole with smoked ham	0.33	0.36	0.38	0.39	0.55	no	Vegetable dishes	no
2635 Broccolilåda utan skinka eller kött	Broccoli casserole without ham or meat	0.01	0.07	0.07	0.07	0.15	no	Vegetable dishes	no
2638 Linsgryta böngryta	Lentil stew, bean stew	0.03	0.09	0.09	0.09	0.17	no	Legume dishes	no
2639 Sallat, olika sorter	Lettuce, various types	0.00	0.00	0.00	0.00	0.00	yes	Vegetables and vegetable products	no
2641 Moussaka vegetarisk	Moussaka vegetarian	0.19	0.19	0.19	0.19	0.19	no	Vegetable dishes	no
2649 Vegetarisk "chili con carne" sojafärs	Vegetarian chili con carne with soy mince	0.25	0.28	0.30	0.31	1.00	yes	Legume dishes	no
2650 Taco-kryddad köttfärs	Taco-seasoned ground meat	0.02	0.06	0.06	0.06	0.12	no	Meat dish	no
2651 Sojaprotein färs tacokryddad	Taco-seasoned soy protein mince	1.00	1.00	1.00	1.00	1.00	no	Meat dish	no
2652 Potatis pressad	Pressed potatoes	0.00	0.00	0.00	0.00	0.00	yes	Starchy roots and tubers	no
2653 Klyftpotatis frysvara	Frozen potato wedges	0.00	0.00	0.00	0.00	1.00	yes	Potato dishes	no
2654 Potatisbullar	Potato patties	0.00	1.00	1.00	1.00	1.00	no	Potato dishes	no
2658 Potatisgratäng kylvara eller frysvara	Chilled or frozen potato gratin	0.00	1.00	1.00	1.00	1.00	no	Potato dishes	no
2662 Bönor marinerade	Beans, marinated	0.00	0.00	0.00	0.00	0.00	no	Nuts and legumes	no
2664 Potatissallad, över 10 % fett	Potato salad with over 10% fat	0.00	1.00	1.00	1.00	1.00	no	Potato dishes	no
2669 Vegetarisk färssås sojafärs	Vegetarian mince sauce with soy mince	0.56	0.60	0.63	0.64	0.75	no	Legume dishes	no
2671 Vegetarisk schnitzel stekt	Vegetarian schnitzel, fried	1.00	1.00	1.00	1.00	1.00	no	Legume dishes	no
2672 Ugnsrostade grönsaker och rotfrukter	Oven-roasted vegetables and root vegetables	0.00	0.15	0.15	0.15	0.35	yes	Vegetables and vegetable products	no
2673 Quornbullar stekta	Quorn balls, fried	1.00	1.00	1.00	1.00	1.00	no	Meat and dairy imitations	no
2674 Quornfilé stekt	Quorn fillet, fried	1.00	1.00	1.00	1.00	1.00	no	Meat and dairy imitations	no
2675 Quorn burgare, stekta	Quorn burger, fried	1.00	1.00	1.00	1.00	1.00	no	Meat and dairy imitations	no
2677 Pytt i panna m. mykoprotein veg.	Vegetarian hash with mycoprotein	1.00	1.00	1.00	1.00	1.00	yes	Vegetable dishes	no
2678 Quorn schnitzel	Quorn schnitzel	1.00	1.00	1.00	1.00	1.00	no	Meat and dairy imitations	no
2679 Quorn lasagne	Quorn lasagna	1.00	1.00	1.00	1.00	1.00	no	Pasta dishes	no
2680 Quornkorv stekt	Fried Quorn sausage	1.00	1.00	1.00	1.00	1.00	yes	Meat and dairy imitations	no
2683 Ris persiskt	Persian rice	0.02	0.08	0.08	0.08	0.16	no	Pasta dishes	no
2685 Rispytt med kött kyckling eller korv	Rice hash with meat, chicken, or sausage	0.02	0.25	0.26	0.26	0.29	no	Pasta dishes	no
2686 Ris kokt med fräst lök och buljong, pilaffris	Pilaf rice cooked with sautéed onion and broth	0.02	0.08	0.08	0.08	0.16	no	Pasta dishes	no
2687 Risotto italiensk (med parmesanost)	Italian risotto (with parmesan cheese)	0.02	0.08	0.08	0.08	0.16	no	Pasta dishes	no
2688 Risnudlar glasnudlar wokade	Stir-fried rice noodles or glass noodles	0.02	0.08	0.08	0.08	0.16	no	Pasta dishes	no
2689 Äggnudlar wokade	Stir-fried egg noodles	0.02	0.08	0.08	0.08	0.16	no	Pasta and similar	no
2695 Pastagratäng med ostsås	Pasta gratin with cheese sauce	0.00	0.47	0.47	0.47	0.47	no	Pasta dishes	no

2696	Pastagratäng med ostsås och skinka	Pasta gratin with cheese sauce and ham	0.14	0.55	0.55	0.55	0.55	no	Pasta dishes	no
2698	Pastagratäng med broccoli	Pasta gratin with broccoli	0.02	0.08	0.08	0.08	0.15	no	Pasta dishes	no
2699	Pastagratäng med blandade grönsaker	Pasta gratin with mixed vegetables	0.02	0.08	0.08	0.08	0.15	no	Pasta dishes	no
2700	Linser marinerade	Lentils, marinated	0.00	0.00	0.00	0.00	0.00	no	Nuts and legumes	no
2701	Kikärtor marinerade	Chickpeas, marinated	0.00	0.00	0.00	0.00	0.00	no	Nuts and legumes	no
2705	Hallon blåbär frysta tinade	Raspberries blueberries, thawed frozen	0.00	0.00	0.00	0.00	0.00	yes	Fruit	no
2706	Ciabatta och focaccia, grov	Ciabatta and focaccia, coarse	0.00	1.00	1.00	1.00	1.00	no	Bread	no
2711	Tekaka, ljus	Tea cake, light	0.00	1.00	1.00	1.00	1.00	no	Bread	no
2712	Tekaka, grov	Tea cake, coarse	0.00	1.00	1.00	1.00	1.00	no	Bread	no
2713	Vetekaka hällakaka grov	Wheat flatbread, coarse	0.00	1.00	1.00	1.00	1.00	no	Bread	no
2718	Vitlök marinerad	Marinated garlic	0.00	1.00	1.00	1.00	1.00	no	Vegetables and vegetable products	no
2719	Sparris kokt	Asparagus, boiled	0.00	0.00	0.00	0.00	0.00	no	Vegetables and vegetable products	no
2720	Knäckis knäckebrödskex	Crispbread crackers	1.00	1.00	1.00	1.00	1.00	no	Fine bakery wares	no
2721	Start Fullkornslight eller crunchy naturell	Start cereal, whole grain light or crunchy natural	1.00	1.00	1.00	1.00	1.00	yes	Breakfast cereals	no
2723	Formbröd, grovt och nyckelhålsmärkt vitt skivat bröd	Sliced bread, coarse and keyhole-labeled white bread	0.00	1.00	1.00	1.00	1.00	yes	Bread	no
2724	Boveteflingor	Buckwheat flakes	0.00	0.00	0.00	0.00	0.00	no	Cereals	no
2725	Lättmjölk ekologisk (0,5 % fett)	Low-fat milk (0.5% fat), organic	0.00	0.00	0.00	0.00	0.00	yes	Milk, yoghurt and similar	yes
2726	Mellanmjölk ekologisk (1,5 % fett)	Semi-skimmed milk (1.5% fat), organic	0.00	0.00	0.00	0.00	0.00	no	Milk, yoghurt and similar	yes
2728	Lättmargarin fett ca 40%	Light margarine, approx. 40% fat	1.00	1.00	1.00	1.00	1.00	no	Fat	no
2729	Lättmargarin Minilätta LättLätt fett 28-30%	Light margarine (Minilätta/LättLätt), 28–30% fat	1.00	1.00	1.00	1.00	1.00	no	Fat	no
2730	Smör- & rapsolja	Butter and rapeseed oil blend	0.00	1.00	1.00	1.00	1.00	no	Fat	no
2731	Mjölk ekologisk (3 % fett)	Whole milk (3% fat), organic	0.00	0.00	0.00	0.00	0.00	no	Milk, yoghurt and similar	yes
2732	Matlagningsyoghurt lätt 3,5 % fett	Cooking yoghurt, light (3.5% fat)	0.00	0.00	0.00	0.00	1.00	no	Milk, yoghurt and similar	no
2733	Grekisk eller turkisk yoghurt (10 % fett)	Greek or Turkish yoghurt (10% fat)	0.00	0.00	0.00	0.00	0.00	no	Milk, yoghurt and similar	no
2735	Lättfil ekologisk 0,5 % fett	Low-fat fermented milk (0.5% fat), organic	0.00	0.00	0.00	0.00	0.00	yes	Milk, yoghurt and similar	no
2736	Mellanfil 1,5% fett ekologisk	Semi-fat fermented milk (1.5% fat), organic	0.00	0.00	0.00	0.00	0.00	yes	Milk, yoghurt and similar	no
2737	Filmjölk ekologisk fett 3 %	Fermented milk, 3% fat, organic	0.00	0.00	0.00	0.00	0.00	no	Milk, yoghurt and similar	no
2744	Varmkorv grillkorv wienerkorv mager kokt	Lean hot dog/grill sausage/wiener, boiled	1.00	1.00	1.00	1.00	1.00	yes	Meat	no
2745	Varmkorv grillkorv wienerkorv mager stekt eller grillad	Lean hot dog/grill sausage/wiener, fried or grilled	1.00	1.00	1.00	1.00	1.00	yes	Meat	no
2746	Prinskorv mager	Lean cocktail sausage	1.00	1.00	1.00	1.00	1.00	yes	Meat	no
2747	Frukostkorv lunchkorv mager (fett högst 10 %)	Lean breakfast/lunch sausage (max 10% fat)	1.00	1.00	1.00	1.00	1.00	yes	Meat	no
2748	Isterband mager (högst 10 % fett)	Lean fermented sausage (max 10% fat)	1.00	1.00	1.00	1.00	1.00	yes	Meat	no
2749	Kryddig korv typ chorizo mager (högst 10 % fett)	Lean spicy sausage (e.g., chorizo, max 10% fat)	1.00	1.00	1.00	1.00	1.00	no	Meat	no
2750	Köttkorv fläskkorv mager (högst 10 % fett) kokt	Lean meat/pork sausage (max 10% fat), boiled	1.00	1.00	1.00	1.00	1.00	yes	Meat	no
2751	Lasagne m. kyckling el. kalkon	Lasagna with chicken or turkey	0.00	1.00	1.00	1.00	1.00	no	Pasta dishes	no
2752	Köttfärssås kycklingfärs kalkonfärs	Meat sauce with chicken or turkey mince	0.02	0.06	0.06	0.06	0.12	no	Meat dish	no
2753	Taco-kryddad kycklingfärs kalkonfärs	Taco-seasoned chicken or turkey mince	0.02	0.06	0.06	0.06	0.12	yes	Meat dish	no
2756	Vit sås béchamelsås standardmjölk	White sauce (béchamel) with standard milk	0.00	0.04	0.08	0.09	0.22	no	Savoury sauces and condiments	no
2757	Sötsur sås	Sweet and sour sauce	0.00	1.00	1.00	1.00	1.00	no	Savoury sauces and condiments	no
2758	Sushi vegetarisk	Vegetarian sushi	0.00	0.00	0.00	0.00	1.00	no	Fish dishes	no
2759	Currysås kylvara	Curry sauce, chilled	1.00	1.00	1.00	1.00	1.00	no	Savoury sauces and condiments	no
2761	Ost- och broccolisoppa	Cheese and broccoli soup	0.00	0.00	0.00	0.00	1.00	no	Soups and salads	no
2762	Ost- och broccolisoppa på pulver	Cheese and broccoli soup powder	1.00	1.00	1.00	1.00	1.00	no	Soups and salads	no
2763	Ost- och broccolisoppa kylvara	Cheese and broccoli soup, chilled	1.00	1.00	1.00	1.00	1.00	no	Soups and salads	no
2765	Sparrissoppa	Asparagus soup	0.00	0.00	0.00	0.00	1.00	no	Soups and salads	no
2766	Sparrissoppa kylvara	Asparagus soup, chilled	1.00	1.00	1.00	1.00	1.00	no	Soups and salads	no
2767	Sparrissoppa frysvara	Asparagus soup, frozen	1.00	1.00	1.00	1.00	1.00	no	Soups and salads	no
2768	Minestronesoppa på pulver	Minestrone soup powder	1.00	1.00	1.00	1.00	1.00	no	Soups and salads	no
2769	Pannkaka tunn stekt	Pancake, thin fried	0.00	0.04	0.08	0.09	1.00	no	Fine bakery wares	no
2770	Broccoli kokt med salt	Broccoli, boiled with salt	0.00	0.00	0.00	0.00	0.00	no	Vegetables and vegetable products	no
2771	Nyponsoppa på pulver	Rosehip soup powder	1.00	1.00	1.00	1.00	1.00	x	Fruit products	no
2772	Brysselkål kokt med salt	Brussels sprouts, boiled with salt	0.00	0.00	0.00	0.00	0.00	no	Vegetables and vegetable products	no
2773	Blomkål kokt med salt	Cauliflower, boiled with salt	0.00	0.00	0.00	0.00	0.00	no	Vegetables and vegetable products	no
2774	Grönsaksblandning ärtor morötter bönor blomkål fryst kokt med salt	Frozen vegetable mix (peas, carrots, beans, cauliflower), boiled with salt	0.00	0.00	0.00	0.00	0.00	no	Vegetables and vegetable products	no
2775	Grönsaksblandning ärter morötter fryst kokt med salt	Frozen vegetable mix (peas and carrots), boiled with salt	0.00	0.00	0.00	0.00	0.00	no	Vegetables and vegetable products	no
2776	Vaxbönor kokta med salt	Wax beans, boiled with salt	0.00	0.00	0.00	0.00	0.00	no	Vegetables and vegetable products	no
2777	Svartrot kokt m. salt	Salsify, boiled with salt	0.00	0.00	0.00	0.00	0.00	no	Vegetables and vegetable products	no
2778	Ciderliknande dryck, light	Cider-like beverage, light	1.00	1.00	1.00	1.00	1.00	x	Water-based beverages	yes
2780	Energidryck typ Red Bull sockerfri	Sugar-free energy drink (e.g., Red Bull)	1.00	1.00	1.00	1.00	1.00	x	Water-based beverages	yes

2781	Linfrö krossat	Crushed flaxseed	0.00	0.00	0.00	0.00	0.00	yes	Nuts and legumes	no
2788	Broccoligratäng	Broccoli gratin	0.01	0.07	0.07	0.07	0.15	no	Vegetable dishes	no
2789	Gröna bönor kokta med salt	Green beans, boiled with salt	0.00	0.00	0.00	0.00	0.00	no	Vegetables and vegetable products	no
2790	Gröna ärter frysta kokta med salt	Frozen green peas, boiled with salt	0.00	0.00	0.00	0.00	0.00	no	Vegetables and vegetable products	no
2791	Gari (inlagd japansk ingefära)	Gari (pickled Japanese ginger)	0.00	1.00	1.00	1.00	1.00	no	Vegetables and vegetable products	no
2792	Bovete kokt med salt	Buckwheat, boiled with salt	0.00	0.00	0.00	0.00	0.00	yes	Cereals	no
2793	Majstortilla	Corn tortilla	0.00	1.00	1.00	1.00	1.00	no	Bread	no
2796	Matvete kokt	Wheat berries, boiled	0.00	0.00	0.00	0.00	0.00	yes	Cereals	no
2797	Kamutvete kokt	Kamut wheat, boiled	0.00	0.00	0.00	0.00	0.00	no	Cereals	no
2799	Mathavre kokt	Barley groats, boiled	0.00	0.00	0.00	0.00	0.00	yes	Cereals	no
2803	Broccoli gratinerad med skinka	Broccoli gratin with ham	0.32	0.35	0.38	0.39	0.55	no	Vegetable dishes	no
2805	Grönsaksbiff stekt	Vegetable patty, fried	0.00	1.00	1.00	1.00	1.00	no	Vegetable dishes	no
2806	Smörgåskex fullkorn	Whole grain sandwich crackers	1.00	1.00	1.00	1.00	1.00	no	Fine bakery wares	no
2808	Rismål m. sylt	Rice pudding with jam	1.00	1.00	1.00	1.00	1.00	x	Pasta dishes	no
2809	Rismål m. sylt u. socker m. sötningsmedel	Rice pudding with jam, sugar-free, with sweetener	1.00	1.00	1.00	1.00	1.00	x	Pasta dishes	no
2810	Mannafrutti	Semolina dessert (Mannafrutti brand)	1.00	1.00	1.00	1.00	1.00	x	Pasta dishes	no
2812	Onaka grönt te (filmjölk med honung och grönt te)	Onaka green tea (fermented milk with honey and green tea)	1.00	1.00	1.00	1.00	1.00	x	Coffee, tea and similar	no
2813	Fruktkvarg mager	Quark cottage cheese, fruit flavour, light	1.00	1.00	1.00	1.00	1.00	x	Milk, yoghurt and similar	no
2815	Polentagröt kokt på mellanmjölk	Polenta porridge cooked with semi-skimmed milk	0.00	0.00	0.00	0.00	0.00	no	Cereals	no
2816	Caffè latte	Caffè latte	0.00	0.00	0.00	0.00	0.00	x	Coffee, tea and similar	yes
2818	Café au lait	Café au lait	0.00	0.00	0.00	0.00	0.00	x	Coffee, tea and similar	yes
2819	Cappuccino	Cappuccino	0.00	0.00	0.00	0.00	0.00	x	Coffee, tea and similar	yes
2822	Bagels	Bagels	0.00	1.00	1.00	1.00	1.00	no	Bread	no
2824	Fullkornslight Start eller crunchy med bär	Granola with berries, wholegrain, light	1.00	1.00	1.00	1.00	1.00	no	Breakfast cereals	no
2825	Start Premium Sensation	Granola, "Start Premium Sensation"	1.00	1.00	1.00	1.00	1.00	no	Breakfast cereals	no
2826	Lövbiff stekt	Beef steak, thinly sliced, fried	0.00	0.00	0.00	0.00	0.00	yes	Meat	no
2828	Renkött stekt	Reindeer meat, fried	0.00	0.00	0.00	0.00	0.00	yes	Meat	no
2829	Stek tillagad	Roast meat, cooked	0.00	0.00	0.00	0.00	0.00	yes	Meat	no
2830	Renskav stekt	Reindeer shavings, fried	0.00	0.00	0.00	0.00	0.00	yes	Meat	no
2831	Älgskav stekt	Elk shavings, fried	0.00	0.00	0.00	0.00	0.00	yes	Meat	no
2832	Skav stekt	Game shavings, fried	0.00	0.00	0.00	0.00	0.00	yes	Meat	no
2833	Wienerschnitzel m griskött	Pork wiener schnitzel	0.00	1.00	1.00	1.00	1.00	no	Meat dish	no
2835	Kalvschnitzel	Veal schnitzel	0.00	0.10	0.10	0.10	0.25	no	Meat dish	no
2843	Vinbär	Currants	0.00	0.00	0.00	0.00	0.00	no	Fruit	no
2844	Köttgryta nöt och gris	Beef and pork stew	0.03	0.09	0.09	0.09	0.17	no	Meat dish	no
2846	Renskavsgryta med grädde	Reindeer shavings, stew with cream	0.00	0.19	0.21	0.22	0.46	no	Meat dish	no
2847	Renskavsgryta utan grädde	Reindeer shavings, stew without cream	0.03	0.09	0.09	0.09	0.12	no	Meat dish	no
2850	Älgskav viltskav gryta (utan grädde)	Elk/game shavings, stew without cream	0.03	0.09	0.09	0.09	0.12	no	Meat dish	no
2851	Älgskav viltskav gryta med grädde	Elk/game shavings, stew with cream	0.00	0.19	0.21	0.22	0.46	no	Meat dish	no
2853	Korv Stroganoff m grädde	Sausage stew "Stroganoff" with cream	0.62	0.63	0.64	0.64	0.97	no	Meat dish	no
2860	Gryta kycklinggryta m. tomat	Chicken stew with tomato	0.00	0.00	0.00	0.00	1.00	no	Meat dish	no
2862	Gryta kycklinggryta m. kokosmjölk	Chicken stew with coconut milk	0.00	0.42	0.44	0.45	1.00	no	Meat dish	no
2863	Kycklinggryta med grädde/crème fraiche	Chicken stew with cream/crème fraîche	0.00	0.00	0.00	0.00	1.00	no	Meat dish	no
2864	Kycklingköttbullar hemgjorda	Chicken meatballs, homemade	0.00	0.00	0.00	0.00	0.00	no	Meat dish	no
2865	Kycklingjärpar	Chicken patties	0.00	0.00	0.00	0.00	0.00	no	Meat dish	no
2866	Chicken à la King	Chicken à la King	0.03	0.09	0.09	0.09	0.17	no	Meat dish	no
2867	Chokladdryck utan tillsatt socker	Chocolate drink without added sugar	1.00	1.00	1.00	1.00	1.00	no	Coffee, tea and similar	yes
2868	Chokladdryck från automat	Chocolate drink from vending machine	1.00	1.00	1.00	1.00	1.00	no	Coffee, tea and similar	yes
2869	Sportdryck med protein	Protein sports drink	1.00	1.00	1.00	1.00	1.00	x	Sugar sweetened beverages	yes
2878	Tropisk juice	Tropical juice	0.00	1.00	1.00	1.00	1.00	x	Juices	yes
2879	Järpar kylvara frysvara	Patties, chilled or frozen	1.00	1.00	1.00	1.00	1.00	no	Meat dish	no
2885	Toscaäpplen toscapäron	Apples or pears, baked with caramellized almond topping	0.00	0.13	0.16	0.17	0.29	x	Sugar and confectionery	no
2886	Tiramisu	Tiramisu	0.33	1.00	1.00	1.00	1.00	x	Dairy based desserts	no
2887	Aubergineröra	Eggplant dip	0.00	1.00	1.00	1.00	1.00	no	Vegetables and vegetable products	no
2888	Tahinisås sesamsås	Tahini sesame sauce	0.00	0.00	0.00	0.00	0.00	no	Nuts and legumes	no
2890	Potatisklyftor m skal ugnssekt m rapsolja	Potato wedges, oven roasted with skin and rapeseed oil	0.00	0.00	0.00	0.00	0.00	no	Starchy roots and tubers	no
2891	Potatisgnocchi	Potato gnocchi	0.00	1.00	1.00	1.00	1.00	no	Pasta and similar	no
2897	Blåbärspaj	Blueberry pie	0.00	0.00	0.00	0.00	1.00	x	Fine bakery wares	no
2899	Halloumi stekt eller grillad	Halloumi, fried or grilled	0.00	0.00	0.00	0.00	0.00	no	Cheese	no
2900	Ost dessertost	Cheese, dessert cheese	0.00	0.00	0.00	0.00	0.00	no	Cheese	no

2902	Bärsås	Berry sauce	0.00	0.00	0.00	0.00	0.00	no	Savoury sauces and condiments	no
2904	Fetaost fårost light (fett högst 10 %)	Feta cheese, sheep milk, light (max 10% fat)	0.00	0.00	0.00	0.00	1.00	no	Cheese	no
2907	Kesella dessert	Quark dessert	1.00	1.00	1.00	1.00	1.00	no	Dairy based desserts	no
2908	Lammrevbensspjäll	Lamb spare ribs	0.00	0.00	0.00	0.00	0.00	no	Meat	no
2909	Viltskav stekt	Game meat shavings, fried	0.00	0.00	0.00	0.00	0.00	yes	Meat	no
2912	Gryta köttgryta nöt gris m. kokosmjölk	Meat stew (beef/pork) with coconut milk	0.00	0.34	0.38	0.39	0.53	no	Meat dish	no
2913	Köttgryta med grädde/crème fraiche	Meat stew with cream/crème fraîche	0.02	0.06	0.06	0.06	0.12	no	Meat dish	no
2915	Köttbullar lammfärs stekta	Lamb meatballs, fried	0.00	0.00	0.00	0.00	1.00	no	Meat dish	no
2916	Köttbullar vilt stekta	Game meatballs, fried	0.00	0.00	0.00	0.00	1.00	yes	Meat dish	no
2917	Köttbullar kylvara/frysvara	Meatballs, chilled/frozen	1.00	1.00	1.00	1.00	1.00	yes	Meat dish	no
2918	Lammfärsjärpar	Lamb patties	0.00	0.00	0.00	0.00	0.00	no	Meat dish	no
2923	Köttfärsgratäng typ tacogratäng	Minced meat casserole, taco-style	0.02	0.07	0.07	0.07	0.15	no	Meat dish	no
2925	Havrekex med choklad	Oat biscuits with chocolate	0.19	1.00	1.00	1.00	1.00	no	Fine bakery wares	no
2926	Moussaka med potatis	Moussaka with potatoes	0.02	0.07	0.07	0.07	0.15	no	Meat dish	no
2930	Vilt färs stekt tacokryddad	Taco-seasoned game mince, fried	0.02	0.06	0.06	0.06	0.12	yes	Meat dish	no
2931	Taco-kryddad lammfärs	Taco-seasoned lamb mince	0.02	0.06	0.06	0.06	0.12	no	Meat dish	no
2932	Taco-kryddad köttfärs kylvara/frysvara	Taco-seasoned minced meat, chilled/frozen	1.00	1.00	1.00	1.00	1.00	no	Meat dish	no
2941	Vit fisk kokt	White fish, boiled	0.00	0.00	0.00	0.00	0.00	yes	Fish and fish products	no
2942	Vit fisk stekt	White fish, fried	0.00	0.00	0.00	0.00	0.00	yes	Fish and fish products	no
2943	Vit fisk panerad och stekt	White fish, breaded and fried	0.00	0.06	0.10	0.12	0.28	no	Fish and fish products	no
2944	Övrig fisk kokt	Other fish, boiled	0.00	0.00	0.00	0.00	0.00	yes	Fish and fish products	no
2945	Övrig fisk stekt	Other fish, fried	0.00	0.00	0.00	0.00	0.00	yes	Fish and fish products	no
2946	Övrig fisk panerad stekt	Other fish, breaded and fried	0.00	0.10	0.10	0.10	0.25	no	Fish and fish products	no
2947	Laxbullar laxburgare	Salmon balls/burgers	0.00	0.00	0.00	0.00	1.00	yes	Fish dishes	no
2948	Fisk och skaldjursgryta	Fish and seafood stew	0.00	0.06	0.06	0.06	0.12	no	Fish dishes	no
2949	Fisk och skaldjursgryta med grädde/crème fraiche	Fish and seafood stew with cream/crème fraîche	0.02	0.06	0.06	0.06	0.12	no	Meat dish	no
2950	Fiskgratäng med spenat eller andra grönsaker	Fish gratin with spinach or other vegetables	0.03	0.05	0.07	0.07	1.00	no	Fish dishes	no
2952	Fisk med frasigt täcke	Fish with crispy topping	0.00	0.10	0.10	0.10	0.25	yes	Fish dishes	no
2954	Laxpytt	Salmon hash	0.00	1.00	1.00	1.00	1.00	no	Fish dishes	no
2957	Knäckebröd	Crispbread	0.00	0.00	0.00	0.00	0.00	yes	Bread	no
2959	Choklad med någon fyllning	Chocolate with filling	1.00	1.00	1.00	1.00	1.00	x	Sugar and confectionery	no
2960	Nougat	Nougat	1.00	1.00	1.00	1.00	1.00	x	Sugar and confectionery	no
2961	Marsipan med choklad	Marzipan with chocolate	1.00	1.00	1.00	1.00	1.00	x	Sugar and confectionery	no
2963	Klubba	Lollipop	1.00	1.00	1.00	1.00	1.00	x	Sugar and confectionery	no
2964	Godisremmar	Candy strips	1.00	1.00	1.00	1.00	1.00	x	Sugar and confectionery	no
2965	Gräddsås	Cream sauce	0.02	1.00	1.00	1.00	1.00	no	Savoury sauces and condiments	no
2966	Vitvinssås	White wine sauce	0.03	0.07	0.07	0.07	0.17	no	Savoury sauces and condiments	no
2969	Skaldjurssås	Seafood sauce	0.00	1.00	1.00	1.00	1.00	no	Savoury sauces and condiments	no
2971	Vinägrett fettfri	Vinaigrette, fat-free	0.00	1.00	1.00	1.00	1.00	no	Savoury sauces and condiments	no
2972	Dressing typ Rhode Island, lätt (fett högst 5 %)	Rhode Island dressing, light (max 5% fat)	1.00	1.00	1.00	1.00	1.00	no	Savoury sauces and condiments	no
2973	Hamburgerdressing	Hamburger dressing	0.01	1.00	1.00	1.00	1.00	no	Savoury sauces and condiments	no
2975	Ketchup lättsöt. el. osötad	Ketchup, lightly sweetened or unsweetened.	1.00	1.00	1.00	1.00	1.00	no	Savoury sauces and condiments	no
2977	Gulaschsoppa	Goulash soup	0.00	0.00	0.00	0.00	1.00	no	Soups and salads	no
2980	Gulaschsoppa på pulver	Goulash soup, from powder	1.00	1.00	1.00	1.00	1.00	no	Soups and salads	no
2981	Fisksoppa skaldjurssoppa kylvara	Fish and seafood soup, chilled	1.00	1.00	1.00	1.00	1.00	no	Soups and salads	no
2982	Fisksoppa skaldjurssoppa	Fish and seafood soup	0.00	0.00	0.00	0.00	0.00	no	Soups and salads	no
2984	Fisksoppa skaldjurssoppa konserv	Fish and seafood soup, canned	0.00	1.00	1.00	1.00	1.00	no	Soups and salads	no
2986	Kycklingsoppa frysvara	Chicken soup, frozen	1.00	1.00	1.00	1.00	1.00	yes	Soups and salads	no
2987	Kycklingsoppa konserv	Chicken soup, canned	1.00	1.00	1.00	1.00	1.00	yes	Soups and salads	no
2988	Kycklingsoppa m. buljongbas	Chicken soup with broth base	0.00	0.00	0.00	0.00	0.00	yes	Soups and salads	no
2989	Redd grönsakssoppa kyld	Vegetable soup, thickened, chilled	1.00	1.00	1.00	1.00	1.00	yes	Soups and salads	no
2991	Löksoppa	Onion soup	0.00	0.00	0.00	0.00	1.00	no	Soups and salads	no
2992	Misosoppa	Miso soup	0.00	1.00	1.00	1.00	1.00	no	Soups and salads	no
2994	Tomatsoppa	Tomato soup	0.00	0.00	0.00	0.00	1.00	no	Soups and salads	no
2997	Ostsoppa på pulver	Cheese soup, from powder	1.00	1.00	1.00	1.00	1.00	no	Soups and salads	no
2999	Tomatsoppa kylvara	Tomato soup, chilled	0.00	1.00	1.00	1.00	1.00	no	Soups and salads	no
3001	Blomkålssoppa	Cauliflower soup	0.03	0.09	0.09	0.09	0.17	yes	Soups and salads	no
3002	Svampsoppa	Mushroom soup	0.03	0.09	0.09	0.09	0.17	no	Soups and salads	no
3003	Svampsoppa kylvara	Mushroom soup, chilled	1.00	1.00	1.00	1.00	1.00	no	Soups and salads	no
3006	Potatissoppa	Potato soup	0.06	0.09	0.11	0.12	0.20	no	Soups and salads	no

3007	Potatissoppa på pulver	Potato soup powder	1.00	1.00	1.00	1.00	1.00	no	Soups and salads	no
3008	Pannacotta	Panna cotta	0.01	0.20	0.20	0.20	1.00	x	Dairy based desserts	no
3009	Ostpaj	Cheese pie	0.00	1.00	1.00	1.00	1.00	no	Sandwiches, pizzas and similar	no
3011	Grönsakspaj	Vegetable pie	0.00	0.04	0.08	0.09	1.00	no	Sandwiches, pizzas and similar	no
3012	Svamppaj	Mushroom pie	0.00	0.09	0.09	0.09	0.22	no	Sandwiches, pizzas and similar	no
3014	Korv pålägg rökt mager	Smoked sausage, lean	1.00	1.00	1.00	1.00	1.00	no	Meat	no
3015	Pannkaka tunn med fläsk	Pancake, thin with pork	0.00	0.44	0.46	0.47	0.55	no	Meat dish	no
3017	Lamm eller får rökt	Smoked lamb or mutton	1.00	1.00	1.00	1.00	1.00	no	Meat	no
3018	Älgkött eller annat viltkött rökt	Smoked elk or other game meat	0.00	0.00	0.00	0.00	0.00	no	Meat	no
3019	Kött rökt	Smoked meat	1.00	1.00	1.00	1.00	1.00	no	Meat	no
3024	Levergryta kycklinglever	Chicken liver stew	0.02	0.06	0.06	0.06	0.12	no	Meat dish	no
3027	Levergryta älg eller annat vilt	Elk or other game liver stew	0.02	0.09	0.09	0.09	0.12	no	Meat dish	no
3032	Leverbiff älglever eller annat vilt	Elk or other game liver patties	0.00	0.00	0.00	0.00	0.00	yes	Meat dish	no
3034	Inlagd sill med tomatsås	Pickled herring with tomato sauce	0.00	1.00	1.00	1.00	1.00	no	Fish and fish products	no
3035	Inlagd sill m gräddfilssås	Pickled herring with sour cream sauce	0.00	1.00	1.00	1.00	1.00	no	Fish and fish products	no
3036	Senapssill m sås	Mustard herring with sauce	0.00	1.00	1.00	1.00	1.00	no	Fish and fish products	no
3037	Gubbröra	Egg and anchovy salad (traditional recipe)	0.42	0.42	0.42	0.42	0.42	no	Fish dishes	no
3038	Bläckfisk grillad	Squid, grilled	0.00	0.00	0.00	0.00	0.00	yes	Fish and fish products	no
3039	Musslor vinkokta - Moules marinière	Wine-steamed mussels (Moules marinière)	0.00	0.00	0.00	0.00	0.00	yes	Fish dishes	no
3040	Musslor eller ostron gratinerade	Gratinated mussels or oysters	0.00	0.00	0.00	0.00	0.00	no	Fish dishes	no
3043	Gustafskorv	"Gustaf" sausage (local specialty)	1.00	1.00	1.00	1.00	1.00	no	Meat	no
3045	Pan pizza fullkorn kylvara/frysvara	Pan pizza, whole grain, chilled/frozen	1.00	1.00	1.00	1.00	1.00	no	Sandwiches, pizzas and similar	no
3046	Pizza fullkorn kylvara/frysvara	Pizza, whole grain, chilled/frozen	1.00	1.00	1.00	1.00	1.00	no	Sandwiches, pizzas and similar	no
3048	Västkustsallad röra	Seafood salad, mayonnaise based	1.00	1.00	1.00	1.00	1.00	no	Savoury sauces and condiments	no
3049	Dallassallad röra	Dallas salad, mayonnaise based	1.00	1.00	1.00	1.00	1.00	no	Savoury sauces and condiments	no
3050	Guacamole avokadoröra	Guacamole (avocado dip)	0.00	0.00	0.00	0.00	1.00	no	Savoury sauces and condiments	no
3051	Hummus - kikärtsröra	Hummus (chickpea dip)	0.00	0.00	0.00	0.00	1.00	no	Savoury sauces and condiments	no
3052	Aioli vitlöksmajonnäs	Aioli (garlic mayonnaise)	0.00	1.00	1.00	1.00	1.00	no	Savoury sauces and condiments	no
3053	Tapenade olivröra	Tapenade (olive spread)	0.00	0.00	0.00	0.00	1.00	no	Savoury sauces and condiments	no
3055	Muffins	Muffins	0.00	1.00	1.00	1.00	1.00	no	Fine bakery wares	no
3056	Chokladmuffins	Chocolate muffins	0.00	1.00	1.00	1.00	1.00	no	Fine bakery wares	no
3057	Sockerkaka marmorerad typ Tigerkaka	Marble cake (Tiger cake style)	0.00	0.00	0.00	0.00	1.00	x	Fine bakery wares	no
3058	Kardemummakaka	Cardamom cake	0.00	0.00	0.00	0.00	1.00	x	Fine bakery wares	no
3059	Sesamkakor sesamkex	Sesame cookies/crackers	1.00	1.00	1.00	1.00	1.00	no	Fine bakery wares	no
3060	Tabouleh	Tabouleh	0.00	0.00	0.00	0.00	0.00	no	Pasta dishes	no
3061	Boveteflingor flakes	Buckwheat flakes	0.00	0.00	0.00	0.00	1.00	no	Cereals	no
3062	Ost- och skinksallad med bulgur couscous eller andra gryn	Cheese and ham salad with bulgur, couscous or other grains	0.10	0.10	0.10	0.10	0.10	no	Soups and salads	no
3063	Pizzabullar	Pizza rolls	0.07	0.08	0.09	0.09	0.70	no	Fine bakery wares	no
3064	Brända mandlar	Candied almonds	0.00	0.00	0.00	0.00	1.00	x	Sugar and confectionery	no
3065	Jordnötter kanderade (med socker)	Candied peanuts (with sugar)	0.00	0.00	0.00	0.00	1.00	x	Nuts and legumes	no
3066	Cashewnötter kanderade (med socker)	Candied cashews (with sugar)	0.00	0.00	0.00	0.00	1.00	x	Nuts and legumes	no
3067	Cashewnötter saltade	Cashews, salted	0.00	0.00	0.00	0.00	0.00	x	Nuts and legumes	no
3068	Pistaschnötter saltade	Pistachios, salted	0.00	0.00	0.00	0.00	0.00	x	Nuts and legumes	no
3069	Chilinötter	Chili nuts	1.00	1.00	1.00	1.00	1.00	x	Nuts and legumes	no
3071	Pinjefrö	Pine nuts	0.00	0.00	0.00	0.00	0.00	yes	Nuts and legumes	no
3072	Macadamianötter saltade	Macadamia nuts, salted	0.00	0.00	0.00	0.00	0.00	x	Nuts and legumes	no
3074	Pumpafrön saltade	Pumpkin seeds, salted	0.00	0.00	0.00	0.00	0.00	x	Nuts and legumes	no
3075	Solrosfrön saltade	Sunflower seeds, salted	0.00	0.00	0.00	0.00	0.00	x	Nuts and legumes	no
3079	Råsocker	Raw sugar	0.00	0.00	0.00	0.00	0.00	x	Sugar and confectionery	no
3081	Sallad med rostbiff eller annat kött och bulgur, couscous eller andra gryn	Salad with roast beef or other meat and bulgur, couscous or other grains	0.02	0.31	0.31	0.31	0.31	no	Soups and salads	no
3082	Kycklingsallad med bulgur couscous andra gryn	Chicken salad with bulgur, couscous or other grains	0.00	0.01	0.01	0.01	0.41	no	Soups and salads	no
3083	Tonfisksallad med bulgur couscous eller andra gryn	Tuna salad with bulgur, couscous or other grains	0.00	0.00	0.00	0.00	0.00	no	Soups and salads	no
3084	Skaldjurssallad med bulgur couscous eller andra gryn	Seafood salad with bulgur, couscous or other grains	0.00	0.00	0.00	0.00	0.00	no	Soups and salads	no
3085	Grekisk sallad med bulgur couscous eller andra gryn	Greek salad with bulgur, couscous or other grains	0.00	0.00	0.00	0.00	0.00	no	Soups and salads	no
3086	Pastasallad med fetaost och oliver	Pasta salad with feta cheese and olives	0.00	0.00	0.00	0.00	0.00	no	Soups and salads	no
3092	Pastasallad med kyckling	Pasta salad with chicken	0.00	0.00	0.00	0.00	1.00	no	Soups and salads	no
3093	Kycklingsallad	Chicken salad	0.00	0.00	0.00	0.00	0.00	no	Soups and salads	no
3094	Räksallad	Shrimp salad	0.00	0.00	0.00	0.00	0.00	yes	Soups and salads	no
3095	Laxsallad	Salmon salad	0.00	0.00	0.00	0.00	0.00	no	Soups and salads	no

3096 Tonfisksallad	Tuna salad	0.00	0.00	0.00	0.00	0.00	yes	Soups and salads	no
3097 Ost- och skinksallad	Cheese and ham salad	0.23	0.23	0.23	0.23	0.23	no	Soups and salads	no
3098 Rostbiffssallad	Roast beef salad	0.00	0.81	0.81	0.81	0.81	no	Soups and salads	no
3099 Bön- och kikärtssallad	Bean and chickpea salad	0.00	0.00	0.00	0.00	0.00	yes	Soups and salads	no
3103 Pastasallad med ost och skinka	Pasta salad with cheese and ham	0.06	0.34	0.34	0.34	0.34	no	Soups and salads	no
3104 Pastasallad med rostbiff	Pasta salad with roast beef	0.00	0.45	0.45	0.45	0.45	no	Soups and salads	no
3110 Pastasallad med räkor	Pasta salad with shrimp	0.00	0.00	0.00	0.00	0.00	no	Soups and salads	no
3111 Cider med ca 4.5 % alkohol	Cider with approx. 4.5% alcohol	1.00	1.00	1.00	1.00	1.00	x	Alcoholic beverages	yes
3112 Sötningsmedel (till kaffe, te m.m.)	Sweetener (for coffee, tea etc.)	1.00	1.00	1.00	1.00	1.00	x	Sugar and confectionery	no
3115 Grillspett med kött och grönsaker	Skewers with meat and vegetables	0.00	0.00	0.00	0.00	0.00	no	Meat dish	no
3122 Måltidsersättning intensiv (gjord på pulver)	Meal replacement, intensive (powder-based)	1.00	1.00	1.00	1.00	1.00	x	Supplements	yes
3123 Måltidsersättning drickfärdig	Meal replacement, ready to drink	1.00	1.00	1.00	1.00	1.00	x	Supplements	yes
3125 Glasspinne aggregat	Ice cream lolly (aggregate)	1.00	1.00	1.00	1.00	1.00	no	Dairy based desserts	no
3126 Glasstrut aggregat	Ice cream cone (aggregate)	1.00	1.00	1.00	1.00	1.00	no	Dairy based desserts	no
3127 Glass grädd	Ice cream, cream based	1.00	1.00	1.00	1.00	1.00	no	Dairy based desserts	no
3128 Glass i bägare	Ice cream in a cup	1.00	1.00	1.00	1.00	1.00	no	Dairy based desserts	no
3129 Glassbars	Ice cream bars	1.00	1.00	1.00	1.00	1.00	no	Dairy based desserts	no
3130 Mjukglass i strut	Soft serve ice cream in cone	1.00	1.00	1.00	1.00	1.00	no	Dairy based desserts	no
3131 Te - grönt	Green tea	0.00	0.00	0.00	0.00	0.00	x	Coffee, tea and similar	yes
3132 Te - rött	Red tea	0.00	0.00	0.00	0.00	0.00	x	Coffee, tea and similar	yes
3133 Kaffe kokt	Coffee, brewed	0.00	0.00	0.00	0.00	0.00	x	Coffee, tea and similar	yes
3135 Lammkorv pålägg	Lamb sausage (cold cuts)	1.00	1.00	1.00	1.00	1.00	no	Meat	no
3136 Bulgur kokt	Bulgur, cooked	0.00	0.00	0.00	0.00	0.00	no	Pasta and similar	no
3138 Alkoläsk	Alcoholic soda-pop	1.00	1.00	1.00	1.00	1.00	x	Alcoholic beverages	yes
3139 Nötter rostade saltade	Nuts, roasted salted	0.00	0.00	0.00	0.00	1.00	x	Nuts and legumes	no
3140 Nötter blandade	Mixed nuts	0.00	0.00	0.00	0.00	0.00	yes	Nuts and legumes	no
3141 Renkorv	Reindeer sausage	0.00	1.00	1.00	1.00	1.00	no	Meat	no
3142 Riskakor med choklad	Rice cakes with chocolate	1.00	1.00	1.00	1.00	1.00	x	Fine bakery wares	no
3143 Riskaka osaltad	Unsalted rice cake	0.00	0.00	0.00	0.00	0.00	no	Fine bakery wares	no
3146 Moussaka	Moussaka	0.02	0.08	0.08	0.08	0.15	no	Meat dish	no
3147 Lingonsylt lättsockrad	Lingonberry jam, light	1.00	1.00	1.00	1.00	1.00	x	Fruit products	no
3148 Sylt lättsockrad	Jam, light	1.00	1.00	1.00	1.00	1.00	x	Fruit products	no
3149 Gelé vinbär el rönnbär	Currant or rowanberry jelly	0.00	1.00	1.00	1.00	1.00	x	Fruit products	no
3150 Fisksoppa skaldjurssoppa pulver	Fish and seafood soup powder	1.00	1.00	1.00	1.00	1.00	no	Soups and salads	no
3154 Ärtsoppa m fläsk kylvara	Pea soup with pork, chilled	1.00	1.00	1.00	1.00	1.00	no	Soups and salads	no
3155 Skinka kokt	Boiled ham	1.00	1.00	1.00	1.00	1.00	no	Meat	no
3160 Champinjonstuvning	Paris mushroom stew	0.01	0.07	0.07	0.07	0.15	no	Vegetable dishes	no
3161 Champinjonsås	Paris mushroom sauce	0.00	1.00	1.00	1.00	1.00	no	Savoury sauces and condiments	no
3163 Sojakorv konserv stek	Soy protein sausage, canned fried	1.00	1.00	1.00	1.00	1.00	no	Meat and dairy imitations	no
3164 Potatisgratäng på mager grädde	Potato gratin with low-fat cream	0.00	0.41	0.41	0.41	0.41	no	Potato dishes	no
3165 Potatisgratäng ospec.	Potato gratin, unspecified	0.00	0.00	0.00	0.00	1.00	no	Potato dishes	no
3166 Frukosthjärtan	Breakfast hearts	0.00	1.00	1.00	1.00	1.00	no	Breakfast cereals	no
3167 Fullkornsflingor	Whole grain flakes	0.00	1.00	1.00	1.00	1.00	no	Breakfast cereals	no
3213 Lammkorv	Lamb sausage	0.00	1.00	1.00	1.00	1.00	no	Meat	no
3214 Falukorv mager stekt	Falu korv (emulsified sausage), fried	1.00	1.00	1.00	1.00	1.00	yes	Meat	no
3238 Frasiga figurer typ skruvar (snacks)	Crispy twists (snacks)	1.00	1.00	1.00	1.00	1.00	x	Savoury snacks	no
3239 Wasabinötter	Wasabi nuts	1.00	1.00	1.00	1.00	1.00	x	Nuts and legumes	no
3245 Grönsaksblandning broccoli ärter	Vegetable mix (broccoli, peas)	0.00	0.00	0.00	0.00	0.00	no	Vegetables and vegetable products	no
3247 Brödkrutonger	Bread croutons	0.00	1.00	1.00	1.00	1.00	no	Fine bakery wares	no
3248 Bars müsli frukt nötter eller choklad	Muesli bars with fruit, nuts or chocolate	1.00	1.00	1.00	1.00	1.00	x	Breakfast cereals	no
3249 Bars måltidsersättning typ Nutrilett	Meal replacement bars (e.g., Nutrilett)	1.00	1.00	1.00	1.00	1.00	x	Supplements	no
3250 Proteinbar	Protein bar	1.00	1.00	1.00	1.00	1.00	x	Supplements	no
3252 Grissini	Grissini (breadsticks)	0.00	0.00	0.00	0.00	1.00	no	Bread	no
3253 Ostbågar med mindre fett	Cheese curls, reduced fat	1.00	1.00	1.00	1.00	1.00	x	Savoury snacks	no
3255 Majssnacks typ Sombreros	Corn snacks, Sombreros type	0.00	0.00	0.00	0.00	0.00	x	Savoury snacks	no
3257 Marsipan	Marzipan	1.00	1.00	1.00	1.00	1.00	x	Sugar and confectionery	no
3258 Nöt och frukt bärmix	Nut and fruit, berry mix	1.00	1.00	1.00	1.00	1.00	x	Savoury snacks	no
3261 Mandlar rostade saltade	Almonds, roasted and salted	0.00	0.00	0.00	0.00	1.00	x	Nuts and legumes	no
3262 Nötter kanderade (med socker)	Nuts, candied (with sugar)	0.00	0.00	0.00	0.00	1.00	x	Nuts and legumes	no
3263 Kex med chokladöverdrag	Biscuits, chocolate-coated	1.00	1.00	1.00	1.00	1.00	no	Fine bakery wares	no

3264 Hamburgare med grovt bröd utan ost	Hamburger, whole grain bun, no cheese	0.02	0.72	0.72	0.72	0.72	no	Sandwiches, pizzas and similar	no
3265 Hamburgare med grovt bröd ost sallad dressing	Hamburger, whole grain bun, cheese, lettuce, dressing	0.02	0.59	0.59	0.59	0.59	no	Sandwiches, pizzas and similar	no
3266 Vegetarisk burgare med bröd sallad dressing	Veggie burger, bun, lettuce, dressing	0.53	1.00	1.00	1.00	1.00	no	Sandwiches, pizzas and similar	no
3267 Baklava	Baklava	0.00	1.00	1.00	1.00	1.00	no	Fine bakery wares	no
3269 Broccolisoppa	Broccoli soup	0.03	0.09	0.09	0.09	0.17	yes	Soups and salads	no
3270 Gulaschsoppa konserv	Goulash soup, canned	1.00	1.00	1.00	1.00	1.00	no	Soups and salads	no
3272 Risifrutti original	Risifrutti, original	1.00	1.00	1.00	1.00	1.00	x	Pasta dishes	no
3274 Skaldjurssås kylvara/frysvara	Seafood sauce, chilled/frozen	1.00	1.00	1.00	1.00	1.00	no	Savoury sauces and condiments	no
3275 Risifrutti lättsockrad	Rice pudding (Risifrutti), lightly sweetened	1.00	1.00	1.00	1.00	1.00	x	Pasta dishes	no
3279 Rödvinssås	Red wine sauce	0.00	1.00	1.00	1.00	1.00	no	Savoury sauces and condiments	no
3280 Tomatsås tomatröra kylvara/frysvara	Tomato sauce, stewed tomatoes, chilled/frozen	0.00	1.00	1.00	1.00	1.00	no	Vegetables and vegetable products	no
3281 Jordgubbssylt jordgubbsmarmelad lättsockrad	Strawberry jam/marmalade, lightly sweetened	1.00	1.00	1.00	1.00	1.00	x	Fruit products	no
3282 Jordgubbssylt jordgubbsmarmelad med sötningsmedel	Strawberry jam/marmalade, with sweetener	1.00	1.00	1.00	1.00	1.00	x	Fruit products	no
3283 Marmelad lättsockrad	Marmalade, lightly sweetened	1.00	1.00	1.00	1.00	1.00	x	Fruit products	no
3285 Apelsinmarmelad citrusmarmelad med sötningsmedel	Orange marmalade/citrus marmalade, with sweetener	1.00	1.00	1.00	1.00	1.00	x	Fruit products	no
3286 Aprikosmarmelad lättsockrad	Apricot marmalade, lightly sweetened	1.00	1.00	1.00	1.00	1.00	x	Fruit products	no
3287 Aprikosmarmelad med sötningsmedel	Apricot marmalade, with sweetener	1.00	1.00	1.00	1.00	1.00	x	Fruit products	no
3288 Cider 2,25 %	Cider, 2.25% alcohol	1.00	1.00	1.00	1.00	1.00	x	Alcoholic beverages	yes
3289 Jordgubbskräm färdig på tetra	Strawberry cream, ready-made in carton	1.00	1.00	1.00	1.00	1.00	x	Fruit products	no
3290 Aprikoskräm färdig på tetra	Apricot cream, ready-made in carton	1.00	1.00	1.00	1.00	1.00	x	Fruit products	no
3291 Hallonkräm färdig på tetra	Raspberry cream, ready-made in carton	1.00	1.00	1.00	1.00	1.00	x	Fruit products	no
3293 Äppelkräm färdig på tetra	Apple cream, ready-made in carton	1.00	1.00	1.00	1.00	1.00	x	Fruit products	no
3296 Jordgubbskräm på pulver	Strawberry cream, powdered	1.00	1.00	1.00	1.00	1.00	x	Fruit products	no
3299 Jordgubbskräm	Strawberry cream	0.00	1.00	1.00	1.00	1.00	x	Fruit products	no
3300 Hallonkräm	Raspberry cream	0.00	1.00	1.00	1.00	1.00	x	Fruit products	no
3301 Aprikoskräm	Apricot cream	0.00	1.00	1.00	1.00	1.00	x	Fruit products	no
3302 Kranvatten kolsyrat	Tap water, carbonated	0.00	0.00	0.00	0.00	0.00	x	Water-based beverages	yes
3305 Korv pålägg kokt mager	Sausage, sliced, boiled, lean	1.00	1.00	1.00	1.00	1.00	yes	Meat	no
3308 Sockerärtor kokta m salt	Sugar peas, boiled with salt	0.00	0.00	0.00	0.00	0.00	no	Vegetables and vegetable products	no
3310 Portionsbröd ljust rågsiktsbröd	Bread roll, light rye flour	0.00	1.00	1.00	1.00	1.00	no	Bread	no
3312 Renkorv pålägg	Reindeer sausage, sliced	0.00	1.00	1.00	1.00	1.00	no	Meat	no
3316 Bordsmargarin (80 % fett) Carlshamn FinFin	Table margarine (80% fat), Carlshamn FinFin	1.00	1.00	1.00	1.00	1.00	no	Fat	no
3317 Svamp stekt	Mushrooms, fried	0.00	0.15	0.15	0.15	0.35	no	Vegetables and vegetable products	no
3318 Oxfilé stekt	Beef tenderloin, fried	0.00	0.00	0.00	0.00	0.00	yes	Meat	no
3319 Rårakor rösti frysvara	Potato pancakes/Rösti, frozen	1.00	1.00	1.00	1.00	1.00	no	Potato dishes	no
3320 Stuvade champinjoner	Mushrooms, creamed	0.01	0.07	0.07	0.07	0.15	no	Vegetable dishes	no
3433 Linfröolja	Linseed oil	0.00	0.00	0.00	0.00	0.00	yes	Fat	no
3499 Barnmat klämmis m. yoghurt banan jordgubb konserv.	Baby food pouch, yoghurt, banana, strawberry, canned	1.00	1.00	1.00	1.00	1.00	x	Children's cereal products	no
3500 Inlagd sill	Pickled herring	0.00	1.00	1.00	1.00	1.00	no	Fish and fish products	no
3506 Fiskpanetter fiskpinnar värmda i ugn eller stekta utan fett	Breaded fish patties/fish sticks, oven-heated or fried without fat	0.00	1.00	1.00	1.00	1.00	yes	Fish and fish products	no
3518 Quinoa röd kokt m salt	Quinoa, red, cooked with salt	0.00	0.00	0.00	0.00	0.00	yes	Cereals	no
3522 Bananchips	Banana chips	0.00	0.00	0.00	0.00	1.00	x	Savoury snacks	no
3523 Ciderkaraktär alkoholfri	Cider-style drink, non-alcoholic	1.00	1.00	1.00	1.00	1.00	x	Alcoholic beverages	yes
3526 Fruktdryck utan kolsyra typ Festis	Fruit drink, non-carbonated, Festis type	1.00	1.00	1.00	1.00	1.00	x	Sugar sweetened beverages	yes
3550 Proteintillskott i pulverform Whey-80	Protein supplement, powdered, Whey-80	1.00	1.00	1.00	1.00	1.00	x	Supplements	no
3551 Proteintillskott i pulverform Vegetabilisk Sojaprotein 73%	Protein supplement, powdered, vegetable soy protein 73%	1.00	1.00	1.00	1.00	1.00	x	Supplements	no
3552 Proteintillskott färdigblandad (Whey-80 + vatten)	Protein supplement, ready-mixed (Whey-80 + water)	1.00	1.00	1.00	1.00	1.00	x	Supplements	yes
3556 Lemon Curd	Lemon curd	0.00	1.00	1.00	1.00	1.00	x	Sugar and confectionery	no
3557 Chutney	Chutney	0.00	0.00	0.00	0.00	1.00	x	Savoury sauces and condiments	no
3559 Lammskavsgryta med grädde	Lamb stew, with cream	0.02	0.06	0.06	0.06	0.12	no	Meat dish	no
3560 Lammskavsgryta (utan grädde)	Lamb stew, without cream	0.03	0.09	0.09	0.09	0.17	no	Meat dish	no
3563 Återhämtningsprodukt Gainer-Pro pulver	Recovery product, Gainer-Pro, powdered	1.00	1.00	1.00	1.00	1.00	x	Supplements	no
3564 Bulgur fullkorn kokt u salt	Bulgur, whole grain, cooked without salt	0.00	0.00	0.00	0.00	0.00	yes	Cereals	no
3565 Papadums	Papadums	0.00	0.00	0.00	0.00	0.00	no	Savoury snacks	no
3566 Grillspett köttfärs nötfärs	Skewers, minced meat, ground beef	0.00	0.00	0.00	0.00	0.00	no	Meat dish	no
3569 Grönsaksblandning morötter palsternacka purjolök rotselleri kokt	Vegetable mix, carrots, parsnip, leek, celeriac, boiled	0.00	0.00	0.00	0.00	0.00	no	Vegetables and vegetable products	no
3570 Återhämtningsprodukt Gainer färdigblandad (Gainer-Pro + vatten)	Recovery product, Gainer, ready-mixed (Gainer-Pro + water)	1.00	1.00	1.00	1.00	1.00	x	Supplements	yes
3573 Ljus sås på pulver smaksatt m citron dill	Light sauce, powdered, flavored with lemon and dill	1.00	1.00	1.00	1.00	1.00	no	Savoury sauces and condiments	no

3575 Tomatsås på pulver	Tomato sauce, powdered	1.00	1.00	1.00	1.00	1.00	no	Savoury sauces and condiments	no
3576 Vitvinssås på pulver	White wine sauce, powdered	1.00	1.00	1.00	1.00	1.00	no	Savoury sauces and condiments	no
3577 Ostsås på pulver	Cheese sauce, powdered	1.00	1.00	1.00	1.00	1.00	no	Savoury sauces and condiments	no
3578 Rödvinssås på pulver	Red wine sauce, powdered	1.00	1.00	1.00	1.00	1.00	no	Savoury sauces and condiments	no
3579 Currysås på pulver	Curry sauce, powdered	1.00	1.00	1.00	1.00	1.00	no	Savoury sauces and condiments	no
3580 Carbonara pastasås på pulver	Pasta sauce, carbonara, powdered	1.00	1.00	1.00	1.00	1.00	no	Savoury sauces and condiments	no
3582 Carbonara pastasås kylvara	Pasta sauce, carbonara, chilled	1.00	1.00	1.00	1.00	1.00	no	Savoury sauces and condiments	no
3585 Ljus sås kylvara	White sauce, chilled	1.00	1.00	1.00	1.00	1.00	no	Savoury sauces and condiments	no
3586 Yoghurtsås	Yoghurt sauce	0.00	1.00	1.00	1.00	1.00	no	Savoury sauces and condiments	no
3588 Fruktsalsa	Fruit salsa	0.00	0.00	0.00	0.00	1.00	no	Savoury sauces and condiments	no
3590 Äppelkaka	Apple cake	0.00	0.00	0.00	0.00	1.00	no	Fine bakery wares	no
3591 Kladdkaka	Gooey chocolate cake	0.00	0.00	0.00	0.00	1.00	no	Fine bakery wares	no
3593 Lammfilé stekt	Lamb fillet, fried	0.00	0.00	0.00	0.00	1.00	yes	Meat	no
3594 Kycklingbröstfilé utan skinn kokt	Chicken breast fillet, skinless, boiled	0.00	0.00	0.00	0.00	0.00	yes	Meat	no
3595 Lammentrecôte stekt	Lamb entrecôte, fried	0.00	0.00	0.00	0.00	0.00	yes	Meat	no
3598 Barbequesås	Barbecue sauce	0.00	1.00	1.00	1.00	1.00	no	Savoury sauces and condiments	no
3599 Barbecuesås	Barbecue sauce	0.00	1.00	1.00	1.00	1.00	no	Savoury sauces and condiments	no
3600 Vildsvinsfilé stekt	Wild boar fillet, fried	0.00	0.00	0.00	0.00	0.00	yes	Meat	no
3601 Vildsvinsstek stekt	Wild boar roast, fried	0.00	0.00	0.00	0.00	0.00	yes	Meat	no
3602 Tahini sesampasta	Tahini, sesame paste	0.00	0.00	0.00	0.00	0.00	x	Nuts and legumes	no
3603 Vildsvinskött kokt	Wild boar meat, boiled	0.00	0.00	0.00	0.00	0.00	no	Meat	no
3605 Älgfilé stekt	Moose fillet, fried	0.00	0.00	0.00	0.00	0.00	yes	Meat	no
3606 Fläskkött friterat	Pork, deep-fried	0.00	0.00	0.00	0.00	0.00	no	Meat	no
3607 Köttfärsjärpar nötfärs	Meat patties, ground beef	0.00	0.00	0.00	0.00	0.00	yes	Meat dish	no
3609 Taco-kryddad köttfärs fläskfärs	Minced meat, pork, taco-seasoned	0.02	0.06	0.06	0.06	0.12	no	Meat dish	no
3610 Taco-kryddad köttfärs nötfärs	Minced meat, beef, taco-seasoned	0.02	0.06	0.06	0.06	0.12	no	Meat dish	no
3611 Köttfärslimpa älgfärs viltfärs	Meatloaf, moose/venison mince	0.00	0.00	0.00	0.00	0.00	yes	Meat dish	no
3613 Köttfärslimpa nötfärs	Meatloaf, beef mince	0.00	0.00	0.00	0.00	0.00	yes	Meat dish	no
3614 Köttfärslimpa lammfärs	Meatloaf, lamb mince	0.00	0.00	0.00	0.00	0.00	no	Meat dish	no
3615 Köttfärsbiff kylvara frysvara	Minced meat patty, chilled/frozen	1.00	1.00	1.00	1.00	1.00	no	Meat dish	no
3616 Köttfärs nötfärs stekt	Minced meat, beef, fried	0.00	0.00	0.00	0.00	0.00	yes	Meat dish	no
3618 Köttfärs blandfärs stekt	Minced meat, mixed, fried	0.00	0.00	0.00	0.00	0.00	no	Meat dish	no
3619 Köttfärs fläskfärs stekt	Minced meat, pork, fried	0.00	0.00	0.00	0.00	0.00	no	Meat dish	no
3620 Köttfärs lammfärs stekt	Minced meat, lamb, fried	0.00	0.00	0.00	0.00	0.00	no	Meat dish	no
3621 Köttfärs älgfärs viltfärs stekt	Minced meat, moose/venison, fried	0.00	0.00	0.00	0.00	0.00	yes	Meat dish	no
3623 Köttfärsbiff älgfärs viltfärs	Minced meat patty, moose/venison	0.00	0.00	0.00	0.00	0.00	yes	Meat dish	no
3624 Hamburgare fläskfärs	Hamburger, pork mince	0.00	0.00	0.00	0.00	0.00	no	Meat dish	no
3625 Hamburgare älgfärs viltfärs	Hamburger, moose/venison mince	0.00	0.00	0.00	0.00	0.00	no	Meat dish	no
3626 Hamburgare lammfärs	Hamburger, lamb mince	0.00	0.00	0.00	0.00	0.00	no	Meat dish	no
3627 Färsspett av fläskfärs	Minced meat skewers, pork	0.00	0.00	0.00	0.00	0.00	no	Meat dish	no
3629 Färsspett av lammfärs	Minced meat skewers, lamb	0.00	0.00	0.00	0.00	0.00	no	Meat dish	no
3630 Grillspett köttfärs blandfärs	Skewers, mixed minced meat	0.00	0.00	0.00	0.00	0.00	no	Meat dish	no
3631 Färsspett kylvara frysvara	Minced meat skewers, chilled/frozen	1.00	1.00	1.00	1.00	1.00	no	Meat dish	no
3632 Kycklingpytt	Chicken hash	0.00	1.00	1.00	1.00	1.00	no	Meat dish	no
3636 Lasagne m. fisk skaldjur	Lasagna, fish and seafood	0.02	0.08	0.08	0.08	0.15	no	Pasta dishes	no
3638 Chili con carne kycklingfärs	Chili con carne, chicken mince	0.03	0.09	0.09	0.09	0.17	yes	Meat dish	no
3639 Köttfärssås köttfärs	Meat sauce, minced meat	0.03	0.06	0.06	0.06	0.12	no	Meat dish	no
3640 Rostade majskorn snacks	Corn kernels, roasted, snack	0.00	0.00	0.00	0.00	0.00	no	Savoury snacks	no
3643 Köttfärsbiff kycklingfärs kalkonfärs	Minced meat patty, chicken/turkey	0.00	0.00	0.00	0.00	0.00	yes	Meat dish	no
3644 Färs kyckling kalkon stekt m. salt	Minced meat, chicken/turkey, fried with salt	0.00	0.00	0.00	0.00	0.00	yes	Meat	no
3645 Biff kokt (nötkött)	Beef, boiled	0.00	0.00	0.00	0.00	0.00	yes	Meat	no
3646 Entrecôte nöt kokt	Entrecôte, beef, boiled	0.00	0.00	0.00	0.00	0.00	no	Meat	no
3648 Rådjurskött kokt	Venison, boiled	0.00	0.00	0.00	0.00	0.00	yes	Meat	no
3649 Grillspett kycklingfärs	Skewers, chicken mince	0.00	0.00	0.00	0.00	1.00	yes	Meat	no
3652 Ost sojabaserad hårdost	Cheese, soy-based, hard	1.00	1.00	1.00	1.00	1.00	no	Meat and dairy imitations	no
3654 Viltkött kokt	Game meat, boiled	0.00	0.00	0.00	0.00	0.00	yes	Meat	no
3657 Sojafärsbiff	Soy mince patty	1.00	1.00	1.00	1.00	1.00	no	Meat and dairy imitations	no
3658 Köttbullar sojafärs	Meatballs, soy mince	1.00	1.00	1.00	1.00	1.00	no	Meat and dairy imitations	no
3659 Hamburgare sojafärs	Hamburger, soy mince	1.00	1.00	1.00	1.00	1.00	no	Meat and dairy imitations	no
3660 Quornfärs stekt	Quorn mince, fried	1.00	1.00	1.00	1.00	1.00	no	Meat and dairy imitations	no

3661 Mykoproteinfärs tacokryddad	Mycoprotein mince, taco-seasoned	1.00	1.00	1.00	1.00	1.00	no	Vegetable dishes	no
3664 Quornfärsbiff	Quorn mince patty	1.00	1.00	1.00	1.00	1.00	no	Vegetable dishes	no
3666 Vegetarisk färssås quornfärs	Vegetarian meat sauce, quorn mince	0.45	0.50	0.54	0.56	0.70	no	Vegetable dishes	no
3667 Vegetarisk "chili con carne" quornfärs	Vegetarian chili con carne, quorn mince	0.18	0.22	0.24	0.25	0.34	yes	Vegetable dishes	no
3668 Sojadryck berikad	Soy drink, fortified	1.00	1.00	1.00	1.00	1.00	yes	Meat and dairy imitations	yes
3672 Älgkött stekt	Moose meat, fried	0.00	0.00	0.00	0.00	0.00	yes	Meat	no
3673 Lammstek kokt	Lamb roast, boiled	0.00	0.00	0.00	0.00	0.00	yes	Meat	no
3675 Älgstek kokt	Moose roast, boiled	0.00	0.00	0.00	0.00	0.00	yes	Meat	no
3676 Hjortfilé kokt	Deer fillet, boiled	0.00	0.00	0.00	0.00	0.00	yes	Meat	no
3677 Hjortfilé stekt	Deer fillet, fried	0.00	0.00	0.00	0.00	0.00	yes	Meat	no
3678 Hjortstek stekt	Deer roast, fried	0.00	0.00	0.00	0.00	0.00	yes	Meat	no
3680 Vegetarisk paj med quornfärs	Vegetarian pie, quorn mince	0.14	0.22	0.28	0.31	0.56	no	Sandwiches, pizzas and similar	no
3683 Pirog vegetarisk quorn el soja	Pirogue, vegetarian, quorn or soy	1.00	1.00	1.00	1.00	1.00	no	Sandwiches, pizzas and similar	no
3695 Falukorv mager kokt	Falukorv, lean, boiled	1.00	1.00	1.00	1.00	1.00	yes	Meat	no
3698 Frukostkorv lunchkorv mager (högst 10 % fett) stekt	Breakfast/lunch sausage, lean (max 10% fat), fried	1.00	1.00	1.00	1.00	1.00	yes	Meat	no
3701 Kryddig korv typ chorizo mager (högst 10 % fett) kokt	Spicy sausage, chorizo type, lean (max 10% fat), boiled	1.00	1.00	1.00	1.00	1.00	yes	Meat	no
3702 Kryddig korv typ chorizo kokt	Spicy sausage, chorizo type, boiled	1.00	1.00	1.00	1.00	1.00	no	Meat	no
3705 Kryddig korv av kyckling kalkon eller struts kokt	Spicy sausage, chicken/turkey/ostrich, boiled	1.00	1.00	1.00	1.00	1.00	yes	Meat	no
3707 Kryddig korv av kyckling kalkon eller struts stekt	Spicy sausage, chicken/turkey/ostrich, fried	1.00	1.00	1.00	1.00	1.00	yes	Meat	no
3709 Renkorv kokt	Reindeer sausage, boiled	0.00	0.00	0.00	0.00	0.00	no	Meat	no
3710 Soygurt	Soygurt	1.00	1.00	1.00	1.00	1.00	no	Meat and dairy imitations	no
3711 Quornkorv kokt	Quorn sausage, boiled	1.00	1.00	1.00	1.00	1.00	yes	Meat and dairy imitations	no
3712 Sojakorv kokt	Soy sausage, boiled	1.00	1.00	1.00	1.00	1.00	no	Meat and dairy imitations	no
3713 Sojadryck choklad berikad	Soy drink, chocolate, fortified	1.00	1.00	1.00	1.00	1.00	yes	Meat and dairy imitations	yes
3714 Pastagratäng med kyckling	Pasta gratin, with chicken	0.00	0.00	0.00	0.00	0.00	no	Pasta dishes	no
3718 Fläskfilé kokt	Pork fillet, boiled	0.00	0.00	0.00	0.00	0.00	yes	Meat	no
3719 Skinkstek fläskstek kokt	Ham/pork roast, boiled	0.00	0.00	0.00	0.00	0.00	yes	Meat	no
3720 Havrebaserad yoghurt typ Yosa	Oat-based yoghurt, Yosa type	1.00	1.00	1.00	1.00	1.00	no	Meat and dairy imitations	no
3722 Nötstek kokt	Beef roast, boiled	0.00	0.00	0.00	0.00	0.00	yes	Meat	no
3723 Rostbiff kokt	Roast beef, boiled	0.00	0.00	0.00	0.00	0.00	yes	Meat	no
3724 Korvgryta med grädde/crème fraiche	Sausage stew, with cream/crème fraîche	0.48	0.49	0.50	0.50	0.73	no	Meat dish	no
3727 Oxfilé kokt	Beef tenderloin, boiled	0.00	0.00	0.00	0.00	0.00	yes	Meat	no
3731 Kaviar typ Kalles randiga	Caviar with cream cheese, Kalles striped type	1.00	1.00	1.00	1.00	1.00	no	Savoury sauces and condiments	no
3732 Lättglögg 2,25%	Light mulled wine, 2.25% alcohol	1.00	1.00	1.00	1.00	1.00	x	Alcoholic beverages	yes
3733 Saftglögg	Mulled fruit drink	1.00	1.00	1.00	1.00	1.00	x	Sugar sweetened beverages	yes
3737 Falukorv av kyckling kalkon eller struts kokt	Falu sausage, chicken/turkey/ostrich, boiled	1.00	1.00	1.00	1.00	1.00	no	Meat	no
3738 Korv falukorv m. kyckling kalkon el. struts stekt	Falu sausage, chicken/turkey/ostrich, panfried	1.00	1.00	1.00	1.00	1.00	yes	Meat	no
3739 Varmkorv grillkorv wienerkorv av kyckling kalkon eller struts stekt	Hot dog/grill sausage/wiener, chicken/turkey/ostrich, panfried	1.00	1.00	1.00	1.00	1.00	yes	Meat	no
3740 Varmkorv grillkorv wienerkorv av kyckling kalkon eller struts kokt	Hot dog/grill sausage/wiener, chicken/turkey/ostrich, boiled	1.00	1.00	1.00	1.00	1.00	yes	Meat	no
3741 Korv prinskorv m. kyckling kalkon el. struts stekt	Cocktail sausage, chicken/turkey/ostrich, panfried	1.00	1.00	1.00	1.00	1.00	yes	Meat	no
3744 Lättyoghurt ekologisk (0,5 % fett)	yoghurt, low-fat, organic (0.5% fat)	0.00	1.00	1.00	1.00	1.00	yes	Milk, yoghurt and similar	no
3752 Couscous kokt m. salt fullkorn	Couscous, whole grain, cooked with salt	0.00	0.00	0.00	0.00	0.00	yes	Pasta and similar	no
3756 Pasta kokt m salt	Pasta, cooked with salt	0.00	0.00	0.00	0.00	0.00	no	Pasta and similar	no
3760 Vita bönor torkade kokta m salt	White beans, dried, cooked with salt	0.00	0.00	0.00	0.00	0.00	no	Nuts and legumes	no
3762 Kikärter torkade kokta m salt	Chickpeas, dried, cooked with salt	0.00	0.00	0.00	0.00	0.00	no	Nuts and legumes	no
3769 Sallad med fisk och bulgur couscous eller andra gryn	Salad, with fish and bulgur/couscous/other grains	0.00	0.00	0.00	0.00	0.00	no	Soups and salads	no
3770 Fläskrulad stekt	Pork roll, panfried	0.00	0.00	0.00	0.00	0.00	yes	Meat	no
3771 Sötpotatis ugnsstekt	Sweet potato, oven-roasted	0.00	0.00	0.00	0.00	0.00	no	Starchy roots and tubers	no
3772 Sötpotatis kokt	Sweet potato, boiled	0.00	0.00	0.00	0.00	0.00	yes	Starchy roots and tubers	no
3773 Pastasallad med lax	Pasta salad, with salmon	0.00	0.30	0.30	0.30	1.00	no	Soups and salads	no
3774 Pastasallad med tonfisk	Pasta salad, with tuna	0.00	0.44	0.44	0.44	1.00	no	Soups and salads	no
3776 Vildsvinskött stekt	Wild boar meat, fried	0.00	0.00	0.00	0.00	0.00	yes	Meat	no
3782 Gryn grynblandning ospec. kokta	Grains, mixed, unspecified, cooked	0.00	0.00	0.00	0.00	0.00	no	Cereals	no
3784 Tunga nöt kokt	Beef tongue, boiled	0.00	0.00	0.00	0.00	0.00	no	Meat	no
3787 Kalkonschnitzel	Turkey schnitzel	0.00	0.04	0.08	0.10	1.00	yes	Meat	no
3789 Tranbärsdryck	Cranberry drink	1.00	1.00	1.00	1.00	1.00	x	Sugar sweetened beverages	yes

3790 Rågsiktsbröd fibrer ca 7%	Rye flour bread, approx. 7% fiber	0.00	1.00	1.00	1.00	1.00	no	Bread	no
3791 Bröd vitt fibrer ca 5% typ Jättefranska	White bread, approx. 5% fiber, Jättefranska type	0.00	1.00	1.00	1.00	1.00	no	Bread	no
3794 Bröd fullkorn vete råg fibrer ca 6%	Whole grain bread, wheat/rye, approx. 6% fiber	1.00	1.00	1.00	1.00	1.00	no	Bread	no
3795 Bröd vitt fibrer ca 5% typ limpa	White bread, approx. 5% fiber, loaf type	1.00	1.00	1.00	1.00	1.00	no	Bread	no
3797 Rågbröd fullkorn fibrer ca 7% typ Kornelle Fullkorn plus	Rye bread, whole grain, approx. 7% fiber, Kornelle Fullkorn Plus	1.00	1.00	1.00	1.00	1.00	yes	Bread	no
3798 Korvbröd grovt	Hot dog bun, whole grain	1.00	1.00	1.00	1.00	1.00	no	Bread	no
3814 Mjukt kakbröd fullkorn fiberrikt	Soft cake bread, whole grain, high fiber	0.00	1.00	1.00	1.00	1.00	yes	Bread	no
3815 Kikärter konserv	Chickpeas, canned	0.00	0.00	0.00	0.00	0.00	yes	Nuts and legumes	no
3816 Kidneybönor röda bönor konserv. u. lag	Kidney beans/red beans, canned, drained	0.00	0.00	0.00	0.00	0.00	no	Nuts and legumes	no
3817 Svarta bönor konserv. u. lag	Black beans, canned, drained	0.00	0.00	0.00	0.00	0.00	yes	Nuts and legumes	no
3818 Hamburgerbröd	Hamburger bun	0.00	1.00	1.00	1.00	1.00	no	Bread	no
3820 Bruna bönor rullpack	Brown beans, roll pack	1.00	1.00	1.00	1.00	1.00	no	Legume dishes	no
3825 Hamburgerbröd grovt	Hamburger bun, whole grain	0.00	1.00	1.00	1.00	1.00	yes	Bread	no
3828 Pasta >50% fullkorn kokt m salt	Pasta, >50% whole grain, cooked with salt	0.00	0.00	0.00	0.00	0.00	yes	Pasta and similar	no
3832 Limpa grov med frön eller kärnor	Bread loaf, whole grain, with seeds or kernels	0.00	1.00	1.00	1.00	1.00	no	Bread	no
3833 Portionsbröd grovt med frön eller kärnor	Bread roll, whole grain, with seeds or kernels	0.00	1.00	1.00	1.00	1.00	no	Bread	no
3834 Mjukt kakbröd hålkaka rågsiktskaka grovt fiberrikt	Soft cake bread, hollow rye flour cake, whole grain, high fiber	0.00	1.00	1.00	1.00	1.00	no	Bread	no
3835 Tortilla grov	Tortilla, whole grain	1.00	1.00	1.00	1.00	1.00	yes	Bread	no
3836 Pitabröd grovt	Pita bread, whole grain	0.00	0.00	0.00	0.00	1.00	no	Bread	no
3840 Ägg rått eko.	Egg, raw, organic	0.00	0.00	0.00	0.00	0.00	x	Egg and egg products	no
3844 Krustader utan fyllning	Pastry shells, unfilled	0.00	0.00	0.00	0.00	0.00	no	Fine bakery wares	no
3845 Dinkel speltvete kokt m. salt	Spelt wheat, cooked with salt	0.00	0.00	0.00	0.00	0.00	no	Cereals	no
3848 Muffins med frukt eller bär	Muffins, with fruit or berries	0.00	1.00	1.00	1.00	1.00	no	Fine bakery wares	no
3849 Kokosmjölk aggregat original och light	Coconut milk, original and light	0.00	1.00	1.00	1.00	1.00	no	Juices	no
3850 Grönsaksgryta med kokosmjölk	Vegetable stew, with coconut milk	0.00	0.72	0.72	0.72	0.72	no	Vegetable dishes	no
3851 Gryta linsgryta böngryta m. kokosmjölk	Stew, lentil/bean, with coconut milk	0.00	0.45	0.46	0.46	0.50	no	Legume dishes	no
3852 Kycklingröra	Chicken mix	1.00	1.00	1.00	1.00	1.00	no	Savoury sauces and condiments	no
3854 Yoghurtshot	Yoghurt shot	1.00	1.00	1.00	1.00	1.00	yes	Milk, yoghurt and similar	yes
3855 Smoothie frukt och bär utan mjölk	Smoothie, fruit and berries, without milk	0.00	0.00	0.00	0.00	1.00	x	Juices	yes
3856 Havresmoothie	Oat-based smoothie	0.00	0.43	0.43	0.43	0.43	no	Juices	yes
3858 Råkostsallad med morot vitkål etc	Raw vegetable salad, with carrot, white cabbage, etc.	0.00	0.00	0.00	0.00	0.00	yes	Vegetables and vegetable products	no
3859 Grönsaker rotfrukter stekta wokade	Vegetables/root vegetables, fried or stir-fried	0.00	0.15	0.15	0.15	0.35	no	Vegetables and vegetable products	no
3861 Nudlar fullkorn kokta	Noodles, whole grain, cooked	0.00	0.00	0.00	0.00	0.00	yes	Pasta and similar	no
3863 Pesto med soltorkade tomater	Pesto, with sun-dried tomatoes	0.00	1.00	1.00	1.00	1.00	no	Savoury sauces and condiments	no
3864 Kryddsmör	Herb butter	0.00	0.00	0.00	0.00	0.00	no	Fat	no
3866 Ris med ärter majs paprika typ Kina-Mix	Rice, with peas, corn, bell pepper (China Mix type)	0.00	0.00	0.00	0.00	0.00	no	Pasta dishes	no
3867 Skott	Mixed shoots	0.00	0.00	0.00	0.00	0.00	yes	Vegetables and vegetable products	no
3868 Pepparrotsvisp	Horseradish whip	1.00	1.00	1.00	1.00	1.00	no	Savoury sauces and condiments	no
3869 Caesardressing	Caesar dressing	0.00	1.00	1.00	1.00	1.00	no	Savoury sauces and condiments	no
3870 Räksallad röra mager	Shrimp salad mix, lean	0.22	1.00	1.00	1.00	1.00	no	Savoury sauces and condiments	no
3871 Rödbetssallad	Beetroot salad	0.00	1.00	1.00	1.00	1.00	no	Vegetable dishes	no
3872 Potatissallad	Potato salad	0.00	1.00	1.00	1.00	1.00	no	Potato dishes	no
3873 Skagenröra (med majonnäs)	Seafood dip (Skagen type), with mayonnaise	0.00	1.00	1.00	1.00	1.00	no	Savoury sauces and condiments	no
3874 Tonfiskröra	Tuna dip	0.00	1.00	1.00	1.00	1.00	no	Savoury sauces and condiments	no
3877 Gröt av blandade gryn	Porridge, mixed grains	0.00	0.00	0.00	0.00	0.00	yes	Breakfast cereals	no
3879 Digestivekex fullkorn	Digestive biscuits, whole grain	1.00	1.00	1.00	1.00	1.00	no	Fine bakery wares	no
3883 Hamburgare med bröd	Hamburger, with bun	0.00	1.00	1.00	1.00	1.00	no	Sandwiches, pizzas and similar	no
3884 Hamburgare med bröd och ost	Hamburger, with bun and cheese	0.00	1.00	1.00	1.00	1.00	no	Sandwiches, pizzas and similar	no
3885 Revbensspjäll vildsvin	Ribs, wild boar	0.00	0.00	0.00	0.00	0.00	no	Meat	no
3892 Ärtsoppa vegetarisk kylvara	Pea soup, vegetarian, chilled	0.00	1.00	1.00	1.00	1.00	yes	Soups and salads	no
3893 Risdryck	Rice drink	0.00	0.00	0.00	0.00	1.00	yes	Meat and dairy imitations	yes
3895 Fruktdryck sockerfri u kolsyra typ Festis Free	Fruit drink, sugar-free, non-carbonated (Festis Free type)	1.00	1.00	1.00	1.00	1.00	x	Water-based beverages	yes
3896 Tonfisk och tomatsås	Tuna, with tomato sauce	0.00	0.00	0.00	0.00	1.00	yes	Fish dishes	no
3897 Klyftpotatis med skal frysvara	Potato wedges, with skin, frozen	0.00	0.00	0.00	0.00	1.00	no	Potato dishes	no
3899 Kålrot stekt eller wokad	Rutabaga, fried or stir-fried	0.00	0.15	0.15	0.15	0.35	no	Vegetables and vegetable products	no
3900 Finskorpa mandelskorpa	Crisp roll, almond crisp	1.00	1.00	1.00	1.00	1.00	no	Fine bakery wares	no
3904 Sill opanerad stekt	Herring, unbreaded, fried	0.00	0.00	0.00	0.00	0.00	yes	Fish and fish products	no
3905 Lättdryck måltidsdryck osötad (olika smaker)	Light meal drink, unsweetened (various flavors)	1.00	1.00	1.00	1.00	1.00	x	Sugar sweetened beverages	yes
3907 Sojagryta med kokosmjölk	Soy protein stew, with coconut milk	0.24	0.80	0.80	0.80	0.80	no	Legume dishes	no
3911 Hackkorv	Sausage, coarse chopped meat	1.00	1.00	1.00	1.00	1.00	no	Meat	no

3913	Tortellini ravioli m. grönsaksfyllning	Tortellini/ravioli, vegetable filling	1.00	1.00	1.00	1.00	1.00	no	Pasta dishes	no
3914	Tortellini ravioli m. svampfyllning	Tortellini/ravioli, mushroom filling	1.00	1.00	1.00	1.00	1.00	no	Pasta dishes	no
3917	Digestivekex med choklad	Digestive biscuits, chocolate-coated	1.00	1.00	1.00	1.00	1.00	no	Fine bakery wares	no
3919	Småkaka typ mördegskaka	Cookie, shortcrust type	0.00	1.00	1.00	1.00	1.00	no	Fine bakery wares	no
3920	Småkakor med nötter	Cookies, with nuts	0.00	1.00	1.00	1.00	1.00	x	Fine bakery wares	no
3921	Småkakor med sylt	Cookies, with jam	0.01	1.00	1.00	1.00	1.00	x	Fine bakery wares	no
3922	Kokoskakor kokostoppar	Coconut cookies, coconut peaks	0.00	1.00	1.00	1.00	1.00	x	Fine bakery wares	no
3923	Småkakor med choklad	Cookies, with chocolate	0.28	1.00	1.00	1.00	1.00	x	Fine bakery wares	no
3924	Havrekaka havreflarn	Oat cookie, oat crisp	0.00	1.00	1.00	1.00	1.00	x	Fine bakery wares	no
3925	Linser konserv	Lentils, canned	0.00	0.00	0.00	0.00	0.00	no	Nuts and legumes	no
3926	Linser kokta med salt	Lentils, cooked with salt	0.00	0.00	0.00	0.00	0.00	no	Nuts and legumes	no
3927	Grönsallat gurka tomat majs utan dressing	Green salad, cucumber, tomato, corn, no dressing	0.00	0.00	0.00	0.00	0.00	yes	Soups and salads	no
3929	Frisésallat	Frisée lettuce	0.00	0.00	0.00	0.00	0.00	yes	Vegetables and vegetable products	no
3935	Laxbullar i sås	Salmon balls, in sauce	1.00	1.00	1.00	1.00	1.00	no	Fish and fish products	no
3939	Fågel vildfågel stekt	Poultry, wild bird, fried	0.00	0.00	0.00	0.00	0.00	no	Meat	no
3943	Anka kokt	Duck, boiled	0.00	0.00	0.00	0.00	0.00	yes	Meat	no
3949	Sockerärter stekta eller wokade	Sugar peas, fried or stir-fried	0.00	0.15	0.15	0.15	0.35	no	Vegetables and vegetable products	no
3951	Gröna bönor haricots verts stekta eller wokade	Green beans (haricots verts), fried or stir-fried	0.00	0.15	0.15	0.15	0.35	no	Vegetables and vegetable products	no
3952	Grönsaker syltade	Vegetables, pickled	0.00	1.00	1.00	1.00	1.00	no	Vegetables and vegetable products	no
3954	Hallonkräm med sötningsmedel	Raspberry cream, with sweetener	1.00	1.00	1.00	1.00	1.00	x	Fruit products	no
3956	Blåbärssoppa osötad eller med sötningsmedel	Blueberry soup, unsweetened or with sweetener	1.00	1.00	1.00	1.00	1.00	x	Fruit products	no
3958	Nyponsoppa osötad eller med sötningsmedel	Rosehip soup, unsweetened or with sweetener	1.00	1.00	1.00	1.00	1.00	x	Fruit products	no
3959	Sparris stekt eller wokad	Asparagus, fried or stir-fried	0.00	0.15	0.15	0.15	0.35	no	Vegetables and vegetable products	no
3960	Drinkmix koncentrat	Drink mix, concentrate	1.00	1.00	1.00	1.00	1.00	x	Sugar sweetened beverages	yes
3963	Tårta nöttårta	Cake, nut cake	0.00	1.00	1.00	1.00	1.00	x	Fine bakery wares	no
3964	Plattfisk kokt	Flatfish, boiled	0.00	0.00	0.00	0.00	0.00	yes	Fish and fish products	no
3965	Plattfisk stekt	Flatfish, fried	0.00	0.00	0.00	0.00	0.00	no	Fish and fish products	no
3966	Plattfisk panerad stekt	Flatfish, breaded and fried	0.00	0.10	0.10	0.10	0.25	no	Fish and fish products	no
3967	Fruktyoghurt (3-5% fett)	Fruit yoghurt (3–5% fat)	1.00	1.00	1.00	1.00	1.00	no	Milk, yoghurt and similar	no
3968	Yoghurt smaksatt (mer än 5% fett)	Flavoured yoghurt (more than 5% fat)	1.00	1.00	1.00	1.00	1.00	no	Milk, yoghurt and similar	no
3971	Risdryck choklad, kalciumberikad	Rice drink, chocolate, calcium-fortified	1.00	1.00	1.00	1.00	1.00	x	Meat and dairy imitations	yes
3973	Bönor kokta konserv	Beans, cooked, canned	0.00	0.00	0.00	0.00	0.00	no	Nuts and legumes	no
3974	Aubergine marinerad i olja	Eggplant, marinated in oil	0.00	0.00	0.00	0.00	0.00	no	Vegetables and vegetable products	no
3975	Kronärtskocka marinerad	Artichoke, marinated	0.00	0.00	0.00	0.00	1.00	no	Vegetables and vegetable products	no
3976	Oliver marinerade i olja	Olives, marinated in oil	0.00	0.00	0.00	0.00	1.00	no	Vegetables and vegetable products	no
3977	Grönsaker marinerade	Vegetables, marinated	0.00	0.00	0.00	0.00	0.00	no	Vegetables and vegetable products	no
3978	Yoghurt naturell ekologisk 3 % fett	yoghurt, plain, organic, 3% fat	0.00	0.00	0.00	0.00	0.00	no	Milk, yoghurt and similar	no
3980	Dryck med vitaminer och mineraler typ Vitamin	Drink, vitamin and mineral enriched (Vitamin type)	1.00	1.00	1.00	1.00	1.00	x	Water-based beverages	yes
3983	Nudlar fullkorn wokade	Noodles, whole grain, stir-fried	0.02	0.08	0.08	0.08	0.16	no	Pasta and similar	no
3986	Smörgåskex salta fullkorn	Crackers, salty, whole grain	1.00	1.00	1.00	1.00	1.00	no	Fine bakery wares	no
3988	Kött ospec. kokt	Meat, unspecified, boiled	0.00	0.00	0.00	0.00	0.00	yes	Meat	no
3989	Kött ospec. Stekt	Meat, unspecified, fried	0.00	0.00	0.00	0.00	0.00	yes	Meat	no
3990	Gammaldags mjölk ekologisk fett 4,2 %	Milk, old-fashioned, organic, 4.2% fat	0.00	0.00	0.00	0.00	0.00	no	Milk, yoghurt and similar	yes
3993	Råbiff	Steak tartare	0.00	0.00	0.00	0.00	0.00	no	Meat	no
3996	Räkor stekta eller wokade	Shrimp, fried or stir-fried	0.00	0.00	0.00	0.00	0.00	no	Fish and fish products	no
3999	Pannkakor plättar av havredryck	Pancakes, oat drink-based	0.00	0.24	0.28	0.29	0.42	no	Fine bakery wares	no
4001	Grönsaker ospec. friterade	Vegetables, unspecified, deep-fried	0.00	0.00	0.00	0.00	0.00	no	Vegetables and vegetable products	no
4002	Chips rotfrukter	Chips, root vegetables	0.00	0.00	0.00	0.00	0.00	x	Savoury snacks	no
4003	Vegetariskt bacon	Bacon, vegetarian	1.00	1.00	1.00	1.00	1.00	no	Meat and dairy imitations	no
4004	Grönsaksmos	Vegetable mash	0.00	0.00	0.00	0.00	0.00	yes	Vegetables and vegetable products	no
4006	Drickyoghurt sojabaserad	Drinking yoghurt, soy-based	1.00	1.00	1.00	1.00	1.00	no	Meat and dairy imitations	yes
4007	Löksås kylvara	Onion sauce, chilled	1.00	1.00	1.00	1.00	1.00	no	Savoury sauces and condiments	no
4009	Räkor rökta	Shrimp, smoked	0.00	0.00	0.00	0.00	0.00	yes	Fish and fish products	no
4010	Musslor rökta	Mussels, smoked	0.00	0.00	0.00	0.00	0.00	yes	Fish and fish products	no
4011	Majskrokar	Corn puffs	0.00	0.00	0.00	0.00	0.00	x	Savoury snacks	no
4012	Sparrissoppa pulver tillagad med vatten, berikad	Asparagus soup, powdered, prepared with water, fortified	1.00	1.00	1.00	1.00	1.00	no	Soups and salads	no
4014	Kött exotiskt kokt	Meat, exotic, boiled	0.00	0.00	0.00	0.00	0.00	yes	Meat	no
4018	Grönsakspaté	Vegetable pâté	0.00	0.00	0.00	0.00	0.00	no	Vegetable dishes	no
4020	Bönsalsa	Bean salsa	0.00	0.00	0.00	0.00	1.00	no	Legume dishes	no
4021	Ananas, torkad, sockrad	Pineapple, dried and sweetened	0.00	0.00	0.00	0.00	0.00	x	Fruit products	no

4022 Efterrättssufflé	Dessert soufflé	0.00	0.00	0.00	0.00	0.00	x	Sugar and confectionery	no
4025 Wasabi	Wasabi	1.00	1.00	1.00	1.00	1.00	x	Savoury sauces and condiments	no
4026 Frukt torkad och sötad	Fruit, dried and sweetened	0.00	0.00	0.00	0.00	1.00	x	Fruit products	no
4027 Grönsaksgryta med grädde/crème fraiche	Vegetable stew, with cream/crème fraîche	0.01	0.06	0.06	0.06	0.12	no	Vegetable dishes	no
4029 Linsgryta böngryta med grädde/crème fraiche	Lentil/bean stew, with cream/crème fraîche	0.04	0.03	0.04	0.04	0.06	no	Legume dishes	no
4031 Quorngryta med grädde/crème fraiche	Quorn stew, with cream/crème fraîche	0.23	0.25	0.27	0.28	0.66	no	Vegetable dishes	no
4032 Sojagryta med grädde/crème fraiche	Soy stew, with cream/crème fraîche	0.21	0.24	0.26	0.27	0.66	no	Legume dishes	no
4033 Gryta m. sojaprotein tomatbas veg.	Stew, soy protein, tomato-based, vegetarian	0.54	0.60	0.64	0.66	0.82	no	Legume dishes	no
4034 Quorngryta	Quorn stew	0.56	0.61	0.66	0.67	0.83	yes	Vegetable dishes	no
4036 Korvgryta med kokosmjölk	Sausage stew, with coconut milk	0.51	0.85	0.86	0.87	0.90	no	Meat dish	no
4037 Korvgryta	Sausage stew	0.72	0.73	0.74	0.75	0.79	no	Meat dish	no
4038 Levergryta ospec.	Liver stew, unspecified	0.02	0.06	0.06	0.06	0.12	no	Meat dish	no
4041 Majsbröd, spröda	Corn bread, crisp	0.00	0.00	0.00	0.00	0.00	no	Bread	no
4044 Risbröd spröda	Rice bread, crisp	0.00	0.00	0.00	0.00	0.00	no	Bread	no
4046 Jordgubbar torkad sockrad	Strawberries, dried and sweetened	0.00	0.00	0.00	0.00	1.00	x	Fruit products	no
4049 Tranbär torkade	Cranberries, dried	0.00	0.50	0.50	0.50	1.00	x	Fruit products	no
4052 Mâchesallat	Lamb's lettuce	0.00	0.00	0.00	0.00	0.00	yes	Vegetables and vegetable products	no
4053 Chips vete baconsmak	Chips, wheat, bacon-flavored	1.00	1.00	1.00	1.00	1.00	x	Savoury snacks	no
4055 Mozzarella ost fett 9 %	Mozzarella cheese, 9% fat	0.00	0.00	0.00	0.00	0.00	no	Cheese	no
4056 Keso mini fett 1,5%	Cottage cheese, mini, 1.5% fat	1.00	1.00	1.00	1.00	1.00	yes	Milk, yoghurt and similar	no
4057 Chai latte	Chai latte	0.00	1.00	1.00	1.00	1.00	x	Coffee, tea and similar	yes
4058 Proteintillskott färdigblandad (Whey-80 + lättmjölk)	Protein supplement, ready-mixed (Whey-80 + low-fat milk)	0.60	0.60	0.60	0.60	0.60	x	Supplements	yes
4059 Återhämtningsprodukt Gainer färdigblandad (Gainer-Pro + mjölk)	Recovery product, Gainer, ready-mixed (Gainer-Pro + milk)	0.79	0.79	0.79	0.79	0.79	x	Supplements	yes
4060 Mousserande vin, champagne	Sparkling wine, champagne	0.00	0.00	0.00	0.00	0.00	x	Alcoholic beverages	yes
4061 Vin rosé	Wine, rosé	0.00	0.00	0.00	0.00	0.00	x	Alcoholic beverages	yes
4067 Naan indiskt mjukt bröd	Naan, Indian soft bread	0.00	0.00	0.00	0.00	0.00	no	Bread	no
4070 Ost halloumi light stekt eller grillad	Cheese, halloumi light, fried or grilled	0.00	0.00	0.00	0.00	0.00	no	Cheese	no
4072 Räkchips	Shrimp chips	1.00	1.00	1.00	1.00	1.00	x	Savoury snacks	no
4076 Pilgrimsmusslor kammusslor stekta	Scallops, fried	0.00	0.00	0.00	0.00	0.00	yes	Fish and fish products	no
4079 Pasta vit fiber kokt m salt	Pasta, white fiber, cooked with salt	1.00	1.00	1.00	1.00	1.00	no	Pasta and similar	no
4081 Chips typ Pringles	Chips, Pringles type	1.00	1.00	1.00	1.00	1.00	x	Savoury snacks	no
4088 Musslor kokta	Mussels, boiled	0.00	0.00	0.00	0.00	0.00	yes	Fish and fish products	no
4089 Sojabitar stekta	Soy pieces, fried	1.00	1.00	1.00	1.00	1.00	no	Meat and dairy imitations	no
4090 Nuggets quorn	Nuggets, quorn	1.00	1.00	1.00	1.00	1.00	no	Meat and dairy imitations	no
4091 Nuggets sojaprotein	Nuggets, soy protein	1.00	1.00	1.00	1.00	1.00	no	Meat and dairy imitations	no
4093 Pastasallad med bönor kikärtor	Pasta salad, with beans and chickpeas	0.00	0.00	0.00	0.00	0.00	no	Soups and salads	no
4094 Sallad med bönor kikärter och bulgur couscous eller andra gryn	Salad, with beans, chickpeas, and bulgur/couscous/other grains	0.00	0.00	0.00	0.00	0.00	no	Soups and salads	no
4096 Banan stekt med socker	Banana, fried with sugar	0.00	0.04	0.04	0.04	0.10	x	Sugar and confectionery	no
4097 Fruktsallad stekt	Fruit salad, fried	0.00	0.00	0.00	0.00	0.00	x	Sugar and confectionery	no
4098 Rimmat sidfläsk vildsvin stekt	Bacon, wild boar, cured and fried	0.00	0.00	0.00	0.00	0.00	no	Meat	no
4101 Päronhalvor inkokta	Pear halves, poached	0.00	0.00	0.00	0.00	0.00	x	Fruit products	no
4102 Salsa typ grönsakssalsa	Salsa, vegetable type	0.00	0.00	0.00	0.00	1.00	no	Savoury sauces and condiments	no
4120 Havskräfta kokt	Norway lobster, boiled	0.00	0.00	0.00	0.00	0.00	yes	Fish and fish products	no
4124 Lammlever stekt	Lamb liver, fried	0.00	0.00	0.00	0.00	0.00	yes	Meat	no
4125 Lever älg eller annat vilt stekt	Liver, moose or other game, fried	0.00	0.00	0.00	0.00	0.00	yes	Meat	no
4126 Renlever stekt	Reindeer liver, fried	0.00	0.00	0.00	0.00	0.00	yes	Meat	no
4127 Lever ospec. stekt	Liver, unspecified, fried	0.00	0.00	0.00	0.00	0.00	yes	Meat	no
4128 Hasselbackspotatis bakad	Hasselback potatoes, baked	0.00	0.07	0.12	0.14	0.33	no	Starchy roots and tubers	no
4129 Fruktdryck med ciderkaraktär	Fruit drink, cider-style	1.00	1.00	1.00	1.00	1.00	x	Sugar sweetened beverages	yes
4134 Ägg stekt eko.	Egg, fried, organic	0.00	0.04	0.08	0.09	0.21	x	Egg and egg products	no
4135 Ägg kokt eko.	Egg, boiled, organic	0.00	0.00	0.00	0.00	0.00	x	Egg and egg products	no
4136 Lättmargarin ospec.	Margarine, light, unspecified	1.00	1.00	1.00	1.00	1.00	no	Fat	no
4137 Sashimi	Sashimi	0.00	0.00	0.00	0.00	0.00	yes	Fish dishes	no
4138 Müsli med frukt och bär	Muesli, with fruit and berries	0.00	1.00	1.00	1.00	1.00	yes	Breakfast cereals	no
4139 Müsli med nötter	Muesli, with nuts	0.00	1.00	1.00	1.00	1.00	no	Breakfast cereals	no
4140 Crème fraichesås med kryddgrönt	Crème fraîche sauce, with herbs	0.00	0.00	0.00	0.00	1.00	no	Savoury sauces and condiments	no
4142 Gräddfilsdressing med kryddgrönt	Sour cream dressing, with herbs	0.00	0.00	0.00	0.00	0.00	no	Savoury sauces and condiments	no
4143 Yoghurtdressing med olika smaksättningar	yoghurt dressing, various flavors	0.00	0.96	0.96	0.96	0.96	no	Savoury sauces and condiments	no
4144 Crème fraiche dressing	Crème fraîche dressing	0.00	0.20	0.20	0.20	0.20	no	Savoury sauces and condiments	no

4145	Fisk kallrökt	Fish, cold-smoked	0.00	0.00	0.00	0.00	0.00	yes	Fish and fish products	no
4146	Pastasås såsbas med grädde eller crème fraiche	Pasta sauce, cream or crème fraîche base	1.00	1.00	1.00	1.00	1.00	no	Savoury sauces and condiments	no
4151	Glasspinne light (högst 5,5 % fett)	Ice cream bar, light (max 5.5% fat)	1.00	1.00	1.00	1.00	1.00	x	Dairy based desserts	no
4152	Glasstrut light (högst 5,5 % fett)	Ice cream cone, light (max 5.5% fat)	1.00	1.00	1.00	1.00	1.00	x	Dairy based desserts	no
4153	Smörgåsrån fullkorn	Crispbread, whole grain	1.00	1.00	1.00	1.00	1.00	no	Fine bakery wares	no
4154	Majskorn stekta wokade	Corn kernels, fried or stir-fried	0.00	0.15	0.15	0.15	0.35	no	Vegetables and vegetable products	no
4155	Gröna ärter stekta eller wokade	Green peas, fried or stir-fried	0.00	0.15	0.15	0.15	0.35	no	Vegetables and vegetable products	no
4156	Vaxbönor stekta eller wokade	Wax beans, fried or stir-fried	0.00	0.15	0.15	0.15	0.35	yes	Vegetables and vegetable products	no
4157	Majskolv stekt eller grillad	Corn cob, fried or grilled	0.00	0.15	0.15	0.15	0.35	no	Vegetables and vegetable products	no
4158	Rispytt med fisk eller skaldjur	Rice hash, with fish or seafood	0.02	0.08	0.08	0.08	0.16	no	Pasta dishes	no
4159	Rispytt med grönsaker	Rice hash, with vegetables	0.02	0.08	0.08	0.08	0.16	no	Pasta dishes	no
4160	Nudelwok med kött grönsaker	Noodle wok, with meat and vegetables	0.00	0.22	0.24	0.25	1.00	no	Pasta dishes	no
4161	Nudelwok m. fisk el. skaldjur	Noodle wok, with fish or seafood	0.00	0.04	0.04	0.04	1.00	no	Pasta dishes	no
4162	Nötkött ospec. Stekt	Beef, unspecified, fried	0.00	0.00	0.00	0.00	0.00	yes	Meat	no
4163	Nötkött ospec. kokt	Beef, unspecified, boiled	0.00	0.00	0.00	0.00	0.00	yes	Meat	no
4164	Kalvsadel stekt	Veal saddle, fried	0.00	0.00	0.00	0.00	0.00	yes	Meat	no
4165	Griskött fläskkött stekt	Pork, fried	0.00	0.00	0.00	0.00	0.00	yes	Meat	no
4166	Griskött fläskkött kokt	Pork, boiled	0.00	0.00	0.00	0.00	0.00	yes	Meat	no
4167	Lammkött stekt	Lamb, fried	0.00	0.00	0.00	0.00	0.00	yes	Meat	no
4168	Lammkött kokt	Lamb, boiled	0.00	0.00	0.00	0.00	0.00	yes	Meat	no
4171	Fläskkött friterat	Pork, deepfried	0.00	0.00	0.00	0.00	0.00	yes	Meat	no
4172	Lammkött friterat	Lamb, deepfried	0.00	0.00	0.00	0.00	0.00	no	Meat	no
4174	Carpaccio nötkött	Carpaccio, beef	0.00	0.00	0.00	0.00	0.00	yes	Meat	no
4178	Älgkött eller annat viltkött torkat	Elk- or other game meat, dried	0.00	0.00	0.00	0.00	0.00	no	Meat	no
4182	Nötkött torkat	Beef, dried	0.00	1.00	1.00	1.00	1.00	no	Meat	no
4183	Köttfärs ospec. stekt	Minced meat, unspecified, fried	0.00	0.02	0.03	0.04	0.09	no	Meat	no
4184	Omelett med potatis typ spansk tortilla	Omelette, with potato, Spanish tortilla type	0.00	0.08	0.08	0.08	0.18	no	Egg dishes	no
4186	Omelett m potatis grönsaker ost etc	Omelette, with potato, vegetables, cheese, etc.	0.00	0.08	0.08	0.08	0.18	no	Egg dishes	no
4187	Lax laxfiskar panerad stekt	Salmon, breaded and panfried	0.00	0.10	0.10	0.10	0.25	yes	Fish and fish products	no
4188	Inlagd strömming	Herring, pickled	0.00	0.00	0.00	0.00	0.00	no	Fish and fish products	no
4190	Sill kokt	Herring, boiled	0.00	0.00	0.00	0.00	0.00	yes	Fish and fish products	no
4191	Vit fisk rökt	White fish, smoked	0.00	0.00	0.00	0.00	0.00	yes	Fish and fish products	no
4194	Fisk vit gravad	White fish, cured	0.00	0.00	0.00	0.00	0.00	no	Fish and fish products	no
4196	Brysselkål stekt eller wokad	Brussels sprouts, panfried or stir-fried	0.00	0.15	0.15	0.15	0.35	no	Vegetables and vegetable products	no
4197	Pumpa eller squash kokt	Pumpkin or squash, boiled	0.00	0.00	0.00	0.00	0.00	yes	Vegetables and vegetable products	no
4199	Avokadosoppa	Avocado soup	0.00	0.00	0.00	0.00	0.00	no	Soups and salads	no
4200	Marängtårta	Meringue cake	0.00	0.34	0.34	0.34	1.00	x	Sugar and confectionery	no
4201	Frukostflingor Special K	Breakfast cereal, Special K	0.00	0.00	0.00	0.00	0.00	no	Breakfast cereals	no
4202	Frukostflingor vetekli Allbran Regular	Breakfast cereal, wheat bran (Allbran Regular)	1.00	1.00	1.00	1.00	1.00	no	Breakfast cereals	no
4206	Frukostflingor Loops	Breakfast cereal, Loops	1.00	1.00	1.00	1.00	1.00	no	Breakfast cereals	no
4208	Potatisgratäng med kyckling eller kalkon	Potato gratin, with chicken or turkey	0.00	0.00	0.00	0.00	1.00	no	Potato dishes	no
4209	Potatisgratäng med skinka eller annat kött	Potato gratin, with ham or other meat	0.41	0.41	0.41	0.41	1.00	no	Potato dishes	no
4211	Smördegs- eller filodegsinbakade grönsaker	Vegetables, wrapped in puff or filo pastry	0.00	1.00	1.00	1.00	1.00	no	Sandwiches, pizzas and similar	no
4212	Smördegs- eller filodegsinbakad ost	Cheese, wrapped in puff or filo pastry	0.00	1.00	1.00	1.00	1.00	no	Sandwiches, pizzas and similar	no
4213	Smördegs- eller filodegsinbakad frukt	Fruit, wrapped in puff or filo pastry	0.00	0.83	0.83	0.83	0.83	no	Fine bakery wares	no
4214	Smördegs- eller filodegsinbakad choklad	Chocolate, wrapped in puff or filo pastry	0.47	0.97	0.97	0.97	0.97	x	Fine bakery wares	no
4215	Bröd friterat typ Langos (utan fyllning)	Bread, deep-fried (Langos type, without filling)	0.00	0.00	0.00	0.00	0.00	no	Sandwiches, pizzas and similar	no
4222	Blinier ryska plättar	Blini, Russian pancakes	0.00	0.04	0.04	0.04	0.10	no	Fine bakery wares	no
4224	Vårrullar färska (med rispapper)	Spring rolls, fresh (with rice paper)	0.00	0.00	0.00	0.00	0.00	no	Savoury snacks	no
4232	Renstek stekt	Reindeer roast, panfried	0.00	0.00	0.00	0.00	0.00	yes	Meat	no
4235	Renfilé stekt	Reindeer fillet, panfried	0.00	0.00	0.00	0.00	0.00	yes	Meat	no
4239	Rådjursfilé stekt	Venison fillet, panfried	0.00	0.00	0.00	0.00	0.00	yes	Meat	no
4240	Tomat stekt eller wokad	Tomato, panfried or stir-fried	0.00	0.15	0.15	0.15	0.35	no	Vegetables and vegetable products	no
4241	Tomat kokt	Tomato, boiled	0.00	0.00	0.00	0.00	0.00	no	Vegetables and vegetable products	no
4247	Fruktsås	Fruit sauce	0.00	0.00	0.00	0.00	1.00	no	Savoury sauces and condiments	no
4248	Grönsakssås	Vegetable sauce	0.00	0.00	0.00	0.00	1.00	no	Vegetables and vegetable products	no
4252	Risbollar friterade	Rice balls, deep-fried	0.00	0.00	0.00	0.00	0.00	no	Savoury snacks	no
4253	Silltårta laxtårta	Herring cake / Salmon cake	0.22	0.43	0.46	0.47	0.57	no	Sandwiches, pizzas and similar	no
4256	Long drink - starksprit (ca 4-6 cl) blandat med läsk el juice	Long drink, spirits (approx. 4–6 cl) mixed with soda or juice	1.00	1.00	1.00	1.00	1.00	x	Alcoholic beverages	yes
4257	Cocktail - drink med starksprit (utan läsk el juice)	Cocktail, spirits-based (without soda or juice)	1.00	1.00	1.00	1.00	1.00	x	Alcoholic beverages	yes

4258 Long drink - starksprit (ca 4-6 cl) blandat med mjölk	Long drink, spirits (approx. 4–6 cl) mixed with milk	0.67	0.67	0.67	0.67	0.67	x	Alcoholic beverages	yes
4260 Vinbål	Wine punch	0.21	0.21	0.21	0.21	0.21	x	Alcoholic beverages	yes
4265 Psylliumfrön	Psyllium seeds	0.00	0.00	0.00	0.00	0.00	yes	Nuts and legumes	no
4268 Shot med frukt	Fruit shot	1.00	1.00	1.00	1.00	1.00	x	Juices	no
4448 Nudlar risnudlar kokta	Noodles, rice noodles, cooked	0.00	0.00	0.00	0.00	0.00	no	Pasta and similar	no
4511 Färskpotatis kokt m salt	New potatoes, boiled with salt	0.00	0.00	0.00	0.00	0.00	yes	Starchy roots and tubers	no
4610 Senapssill u sås	Mustard herring, without sauce	1.00	1.00	1.00	1.00	1.00	no	Fish and fish products	no
4663 Matfettsblandning fett 43% berikad typ Bregott mindre	Fat spread blend, 43% fat, enriched (Bregott Mindre type)	1.00	1.00	1.00	1.00	1.00	no	Fat	no
4736 Pastasås m. tonfisk curry	Pasta sauce, with tuna and curry	0.00	0.03	0.06	0.07	0.17	yes	Fish dishes	no
4939 Broccoli kokt	Broccoli, boiled	0.00	0.00	0.00	0.00	0.00	yes	Vegetables and vegetable products	no
5150 Mandeldryck berikad	Almond drink, fortified	1.00	1.00	1.00	1.00	1.00	yes	Meat and dairy imitations	yes
5152 Alkoläsk kolsyrad dryck vol. % 4-5	Alcoholic soda, carbonated drink, 4–5% vol.	1.00	1.00	1.00	1.00	1.00	x	Alcoholic beverages	yes
5184 Tranbärsdryck drickf	Cranberry drink, ready to drink	1.00	1.00	1.00	1.00	1.00	x	Sugar sweetened beverages	yes
5188 Barngröt ätf. flerkorn m. frukt berikad	Baby porridge, multigrain with fruit, fortified	1.00	1.00	1.00	1.00	1.00	no	Children's cereal products	no
5189 Barngröt ätf. m. fullkorn frukt bär berikad	Baby porridge, whole grain with fruit and berries, fortified	1.00	1.00	1.00	1.00	1.00	no	Children's cereal products	no
5190 Barngröt ätf. m. frukt berikad	Baby porridge, with fruit, fortified	1.00	1.00	1.00	1.00	1.00	no	Children's cereal products	no
5191 Barngröt ätf. ris mjölkfri berikad	Baby porridge, rice-based, milk-free, fortified	1.00	1.00	1.00	1.00	1.00	no	Children's cereal products	no
5193 Barngröt ätf. m. yoghurt frukt berikad	Baby porridge, with yoghurt and fruit, fortified	1.00	1.00	1.00	1.00	1.00	no	Children's cereal products	no
5194 Barngröt ätf. havre m. frukt berikad	Baby porridge, oat-based with fruit, fortified	1.00	1.00	1.00	1.00	1.00	no	Children's cereal products	no
5195 Barngröt ätf. havre naturell berikad	Baby porridge, oat-based, plain, fortified	1.00	1.00	1.00	1.00	1.00	yes	Children's cereal products	no
5196 Barngröt ätf. havre m. frukt bär berikad	Baby porridge, oat-based with fruit and berries, fortified	1.00	1.00	1.00	1.00	1.00	no	Children's cereal products	no
5218 Pepparsås	Pepper sauce	0.00	1.00	1.00	1.00	1.00	no	Savoury sauces and condiments	no
5499 Kycklingpytt	Chicken hash	0.00	1.00	1.00	1.00	1.00	yes	Meat dish	no
5500 Grönsaksgratäng	Vegetable gratin	0.00	0.00	0.00	0.00	0.00	no	Vegetable dishes	no
5502 Fiskgratäng	Fish gratin	0.02	0.08	0.08	0.08	0.15	no	Fish dishes	no
5509 Kyckling Marengo	Chicken, Marengo style	0.00	0.00	0.00	0.00	0.00	no	Meat dish	no
5510 Rotfruktsgratäng	Root vegetable gratin	0.00	1.00	1.00	1.00	1.00	no	Vegetable dishes	no
5521 Pastagratäng	Pasta gratin	0.02	0.08	0.08	0.08	0.15	no	Pasta dishes	no
5548 Sylt frukt och bär	Jam, fruit and berries	0.00	1.00	1.00	1.00	1.00	x	Fruit products	no
5552 Marmelad	Marmalade	0.00	1.00	1.00	1.00	1.00	x	Fruit products	no
5553 Glasspinne liten	Ice cream bar, small	1.00	1.00	1.00	1.00	1.00	no	Dairy based desserts	no
5554 Glasspinne stor	Ice cream bar, large	1.00	1.00	1.00	1.00	1.00	no	Dairy based desserts	no
5555 Glasstrut stor	Ice cream cone, large	1.00	1.00	1.00	1.00	1.00	no	Dairy based desserts	no
5556 Glasstrut liten	Ice cream cone, small	1.00	1.00	1.00	1.00	1.00	no	Dairy based desserts	no
5557 Glass yoghurtglass	Ice cream, yoghurt-based	1.00	1.00	1.00	1.00	1.00	no	Dairy based desserts	no
5560 Glass mjölkfri	Ice cream, milk-free	1.00	1.00	1.00	1.00	1.00	no	Dairy based desserts	no
5561 Glasstrut kulglass	Ice cream cone, scoop-style	1.00	1.00	1.00	1.00	1.00	no	Dairy based desserts	no
5562 Hårt bröd grovt	Crispbread, coarse	0.00	0.00	0.00	0.00	0.00	yes	Bread	no
5563 Riskaka salt och smaksatt	Rice cake, salted and flavored	0.00	1.00	1.00	1.00	1.00	no	Bread	no
5564 Kex smörgåskex litet	Cracker, sandwich type, small	0.00	1.00	1.00	1.00	1.00	no	Bread	no
5565 Kex smörgåskex stort	Cracker, sandwich type, large	0.00	1.00	1.00	1.00	1.00	no	Bread	no
5566 Bröd halvljust	Bread, semi-white	0.00	1.00	1.00	1.00	1.00	no	Bread	no
5567 Polarbröd	Polar bread	1.00	1.00	1.00	1.00	1.00	no	Bread	no
5568 Bröd mörkt	Bread, dark	0.00	1.00	1.00	1.00	1.00	no	Bread	no
5569 Glass Ben och Jerrys	Ice cream, Ben & Jerry's	1.00	1.00	1.00	1.00	1.00	no	Dairy based desserts	no
5570 Isglass ospec	Ice pop, unspecified	1.00	1.00	1.00	1.00	1.00	x	Sugar and confectionery	no
5572 Bagel med pålägg	Bagel, with topping	0.00	0.50	0.50	0.50	1.00	no	Sandwiches, pizzas and similar	no
5573 Fralla med pålägg	Bread roll, with topping	0.00	0.50	0.50	0.50	1.00	no	Sandwiches, pizzas and similar	no
5574 Toast med pålägg	Toast, with topping	0.00	0.50	0.50	0.50	1.00	no	Sandwiches, pizzas and similar	no
5575 Grillad macka med pålägg	Grilled sandwich, with topping	0.00	0.50	0.50	0.50	1.00	no	Sandwiches, pizzas and similar	no
5576 Focaccia med pålägg	Focaccia, with topping	0.00	0.50	0.50	0.50	1.00	no	Sandwiches, pizzas and similar	no
5577 Wrap med pålägg	Wrap, with topping	0.00	0.50	0.50	0.50	1.00	no	Sandwiches, pizzas and similar	no
5578 Sandwich med pålägg	Sandwich, with topping	0.00	0.50	0.50	0.50	1.00	no	Sandwiches, pizzas and similar	no
5579 Baguette sub med pålägg	Baguette/sub, with topping	0.00	0.50	0.50	0.50	1.00	no	Sandwiches, pizzas and similar	no
5580 Enkla kex kakor små	Simple biscuits/cookies, small	0.00	1.00	1.00	1.00	1.00	no	Fine bakery wares	no
5581 Enkla kex kakor stora	Simple biscuits/cookies, large	0.00	1.00	1.00	1.00	1.00	no	Fine bakery wares	no
5582 Dubbla kex kaka med fyllning	Double biscuit/cookie, with filling	1.00	1.00	1.00	1.00	1.00	no	Fine bakery wares	no
5583 Kex kaka med chokladöverdrag	Biscuit/cookie, chocolate-coated	1.00	1.00	1.00	1.00	1.00	no	Fine bakery wares	no
5584 Müsli	Muesli	0.00	1.00	1.00	1.00	1.00	no	Breakfast cereals	no
5585 Granola start	Granola, Start brand/type	1.00	1.00	1.00	1.00	1.00	no	Breakfast cereals	no

5586 Flingor ringar	Cereal, rings	1.00	1.00	1.00	1.00	1.00	no	Breakfast cereals	no
5589 Flingor kuddar	Cereal, pillows	1.00	1.00	1.00	1.00	1.00	yes	Breakfast cereals	no
5592 Flingor fiber	Cereal, fiber-rich	0.00	1.00	1.00	1.00	1.00	no	Breakfast cereals	no
5593 Flingor majs	Cereal, corn-based	0.00	1.00	1.00	1.00	1.00	no	Breakfast cereals	no
5594 Flingor ris	Cereal, rice-based	0.00	1.00	1.00	1.00	1.00	no	Breakfast cereals	no
5597 Hårt bröd tunt	Crispbread, thin	0.00	0.00	0.00	0.00	0.00	yes	Bread	no
5598 Hårt bröd vitt	Crispbread, white	0.00	0.00	0.00	0.00	0.00	no	Bread	no
5600 Choklad lösgodis	Chocolate, pick-and-mix	1.00	1.00	1.00	1.00	1.00	x	Sugar and confectionery	no
5601 Lösgodis påsgodis	Pick-and-mix candy, bagged	1.00	1.00	1.00	1.00	1.00	x	Sugar and confectionery	no
5602 Tuggummi ospec	Chewing gum, unspecified	1.00	1.00	1.00	1.00	1.00	x	Sugar and confectionery	no
5604 Tablettask	Candy box	1.00	1.00	1.00	1.00	1.00	x	Sugar and confectionery	no
5605 Choklad med fyllning ospec	Chocolate, filled, unspecified	1.00	1.00	1.00	1.00	1.00	x	Sugar and confectionery	no
5606 Naturgodis	Natural sweets/snacks	1.00	1.00	1.00	1.00	1.00	x	Sugar and confectionery	no
5607 Kanelgiffel	Cinnamon roll	1.00	1.00	1.00	1.00	1.00	x	Fine bakery wares	no
5608 Munk ospec	Doughnut, unspecified	1.00	1.00	1.00	1.00	1.00	x	Fine bakery wares	no
5610 Konditorbit	Pastry bite	1.00	1.00	1.00	1.00	1.00	x	Fine bakery wares	no
5611 Gräddtårta ospec	Cream cake, unspecified	0.00	0.00	0.00	0.00	1.00	x	Fine bakery wares	no
5612 Daimtårta	Daim cake	1.00	1.00	1.00	1.00	1.00	x	Fine bakery wares	no
5613 Lättmjölk	Milk, low-fat	0.00	0.00	0.00	0.00	0.00	yes	Milk, yoghurt and similar	yes
5614 Mellanmjölk	Milk, semi-skimmed	0.00	0.00	0.00	0.00	0.00	no	Milk, yoghurt and similar	yes
5615 Mjölk fett >3%	Milk, full-fat (>3%)	0.00	0.00	0.00	0.00	0.00	no	Milk, yoghurt and similar	yes
5684 Havredryck sojadryck	Oat drink / Soy drink	0.00	1.00	1.00	1.00	1.00	yes	Meat and dairy imitations	yes
5690 Havregurt Soygurt	Oat yoghurt / Soy yoghurt	1.00	1.00	1.00	1.00	1.00	no	Meat and dairy imitations	no
5691 Portionsyoghurt	yoghurt, single-serve	1.00	1.00	1.00	1.00	1.00	no	Milk, yoghurt and similar	no
5692 Cottage cheese naturell	Cottage cheese, plain	0.00	0.00	0.00	0.00	0.00	yes	Milk, yoghurt and similar	no
5693 Cottage cheese smaksatt	Cottage cheese, flavored	1.00	1.00	1.00	1.00	1.00	no	Milk, yoghurt and similar	no
5694 Cottage cheese mellanmål	Cottage cheese, snack portion	1.00	1.00	1.00	1.00	1.00	no	Milk, yoghurt and similar	no
5695 Kvarg naturell	Quark, plain	0.00	0.00	0.00	0.00	1.00	no	Milk, yoghurt and similar	no
5696 Kvarg smaksatt	Quark, flavored	1.00	1.00	1.00	1.00	1.00	no	Milk, yoghurt and similar	no
5697 Drickkvarg	Drinking quark	1.00	1.00	1.00	1.00	1.00	x	Milk, yoghurt and similar	yes
5698 Portionsyoghurt med müsli	yoghurt, single-serve, with muesli	1.00	1.00	1.00	1.00	1.00	no	Milk, yoghurt and similar	no
5699 Matfettsblandning	Fat spread blend	0.00	0.00	0.00	0.00	0.00	no	Fat	no
5700 Margarin fett 60-80%	Margarine, 60–80% fat	1.00	1.00	1.00	1.00	1.00	no	Fat	no
5701 Lättmargarin fett 40-60%	Light margarine, 40–60% fat	1.00	1.00	1.00	1.00	1.00	no	Fat	no
5702 Mjölkchoklad	Milk chocolate	1.00	1.00	1.00	1.00	1.00	x	Sugar and confectionery	no
5703 Vegetarisk lasagne	Lasagna, vegetarian	0.02	0.08	0.08	0.08	0.15	no	Pasta dishes	no
5704 Vegetarisk moussaka	Moussaka, vegetarian	0.00	0.00	0.00	0.00	1.00	yes	Vegetable dishes	no
5705 Vegetarisk schnitzel	Schnitzel, vegetarian	1.00	1.00	1.00	1.00	1.00	no	Vegetable dishes	no
5706 Vegetarisk pyttipanna	Hash, vegetarian	0.32	1.00	1.00	1.00	1.00	yes	Vegetable dishes	no
5709 Vegetarisk filé bitar	Fillet, vegetarian, pieces	1.00	1.00	1.00	1.00	1.00	no	Meat and dairy imitations	no
5710 Vegetariska nuggets	Nuggets, vegetarian	1.00	1.00	1.00	1.00	1.00	no	Vegetable dishes	no
5711 Vegetariska bollar	Veggie-balls	1.00	1.00	1.00	1.00	1.00	no	Vegetable dishes	no
5712 Vegetarisk färssås	Meat sauce, vegetarian	0.59	0.62	0.65	0.66	1.00	no	Vegetable dishes	no
5713 Vegetarisk gryta, quorn eller soja	Stew, vegetarian (quorn or soy protein)	0.41	0.41	0.41	0.41	1.00	no	Vegetable dishes	no
5714 Vegetarisk biff	Vegetarian patty	0.00	1.00	1.00	1.00	1.00	no	Meat and dairy imitations	no
5715 Vegetarisk färs	Veggie mince	1.00	1.00	1.00	1.00	1.00	no	Meat and dairy imitations	no
5717 Sallad med rödbetor och chèvre	Salad, with beetroot and chèvre	0.00	0.00	0.00	0.00	0.00	no	Soups and salads	no
5727 Minimjölk fett < 0,1% berikad	Milk, skimmed (<0.1% fat), fortified	0.00	0.00	0.00	0.00	0.00	yes	Milk, yoghurt and similar	yes
5729 Mandlar	Almonds	0.00	0.00	0.00	0.00	0.00	yes	Nuts and legumes	no
5730 Frön	Seeds	0.00	0.00	0.00	0.00	0.00	yes	Nuts and legumes	no
5731 Pommes frites	French fries	0.00	1.00	1.00	1.00	1.00	no	Savoury snacks	no
5732 Potatis stekt och råstekt	Potatoes, panfried and pan-roasted	0.00	0.11	0.11	0.11	0.25	no	Starchy roots and tubers	no
5733 Bulgur kokt vanlig o fullkorn	Bulgur, cooked (regular and whole grain)	0.00	0.00	0.00	0.00	0.00	no	Cereals	no
5734 Matgryn kokt	Cooking grains, cooked	0.00	0.00	0.00	0.00	0.00	no	Cereals	no
5735 Couscous kokt	Couscous, cooked	0.00	0.00	0.00	0.00	0.00	yes	Cereals	no
5736 Kli	Bran	0.00	0.00	0.00	0.00	0.00	yes	Cereals	no
5737 Havregryn	Oats	0.00	0.00	0.00	0.00	0.00	yes	Cereals	no
5738 Nudlar kokta	Noodles, cooked	0.00	0.00	0.00	0.00	0.00	no	Pasta and similar	no
5739 Melon	Melon	0.00	0.00	0.00	0.00	0.00	yes	Fruit	no
5741 Gröna bönor ospec	Green beans, unspecified	0.00	0.00	0.00	0.00	0.00	no	Vegetables and vegetable products	no

5742 Groddar	Sprouts	0.00	0.00	0.00	0.00	0.00	yes	Vegetables and vegetable products	no
5743 Spenat Mangold	Spinach / Swiss chard	0.00	0.00	0.00	0.00	0.00	yes	Vegetables and vegetable products	no
5744 Örtkryddor	Herbs	0.00	0.00	0.00	0.00	0.00	yes	Vegetables and vegetable products	no
5746 Tonfisk konserv	Tuna, canned	0.00	0.00	0.00	0.00	0.00	yes	Fish and fish products	no
5751 Skaldjur ospec	Seafood, unspecified	0.00	0.00	0.00	0.00	0.00	yes	Fish and fish products	no
5758 Aioli	Aioli	0.00	1.00	1.00	1.00	1.00	no	Savoury sauces and condiments	no
5759 Guacamole	Guacamole	0.00	0.00	0.00	0.00	1.00	no	Savoury sauces and condiments	no
5763 Crème fraiche	Crème fraîche	0.00	0.00	0.00	0.00	0.00	no	Savoury sauces and condiments	no
5775 Matlagningsyoghurt	Cooking yoghurt	0.00	0.00	0.00	0.00	1.00	no	Milk, yoghurt and similar	no
5777 Matlagningsbas soja havre	Cooking base, soy/oat	1.00	1.00	1.00	1.00	1.00	no	Meat and dairy imitations	no
5778 Ost hårdost fett 27%	Cheese, hard, 27% fat	0.00	0.00	0.00	0.00	0.00	no	Cheese	no
5779 Mjukost	Soft cheese	1.00	1.00	1.00	1.00	1.00	no	Cheese	no
5783 Ugnspannkaka o fläskpannkaka	Oven pancake / Pork pancake	0.00	0.35	0.35	0.35	0.42	no	Egg dishes	no
5784 Korvgryta m korv chorizo grönsaker rotfrukter	Sausage stew, with chorizo, vegetables, root vegetables	0.79	0.80	0.81	0.81	0.85	no	Meat dish	no
5789 Lax kallrökt el gravad	Salmon, cold-smoked or cured	0.00	0.00	0.00	0.00	0.00	yes	Fish and fish products	no
5794 Inälvs- organmat ej lever	Offal, excluding liver	0.00	0.00	0.00	0.00	0.00	no	Meat	no
5796 Kotlett karré tillagad	Cutlet/shoulder, cooked	0.00	0.00	0.00	0.00	0.00	no	Meat	no
5797 Filé tillagad	Fillet, cooked	0.00	0.00	0.00	0.00	0.00	yes	Meat	no
5798 Biff tillagad	Steak, cooked	0.00	0.00	0.00	0.00	0.00	yes	Meat	no
5799 Kött grillat	Meat, grilled	0.00	0.00	0.00	0.00	1.00	yes	Meat	no
5801 Rom fiskrom	Roe, fish	0.00	0.00	0.00	0.00	1.00	no	Fish and fish products	no
5802 Kaviar på tub	Caviar, tubed	0.00	0.00	0.00	0.00	0.00	no	Savoury sauces and condiments	no
5804 Pasta carbonara	Pasta, carbonara	0.16	0.16	0.16	0.16	1.00	no	Pasta dishes	no
5805 Köttfärslimpa ugnsbakad	Meatloaf, oven-baked	0.00	0.00	0.00	0.00	0.00	no	Meat dish	no
5806 Köttbullar stekta	Meatballs, panfried	0.00	0.50	0.50	0.50	1.00	no	Meat dish	no
5807 Pannbiff stekt	Pan-fried beef patty	0.00	0.50	0.50	0.50	1.00	no	Meat dish	no
5808 Järpar stekta	Meat rolls, panfried	0.00	0.50	0.50	0.50	1.00	no	Meat dish	no
5809 Köttfärssås	Meat sauce	0.02	0.06	0.06	0.06	0.12	no	Meat dish	no
5810 Kebabkött stekt	Kebab meat, panfried	1.00	1.00	1.00	1.00	1.00	no	Meat dish	no
5814 Smörgås subway	Sandwich, Subway style	1.00	1.00	1.00	1.00	1.00	no	Sandwiches, pizzas and similar	no
5831 Caramel frappuccino	Frappuccino, caramel	1.00	1.00	1.00	1.00	1.00	x	Coffee, tea and similar	yes
5833 Brownie	Brownie	0.18	1.00	1.00	1.00	1.00	no	Fine bakery wares	no
5838 Hamburgare big mac, whopper, frisco	Hamburger, Big Mac / Whopper / Frisco	1.00	1.00	1.00	1.00	1.00	no	Sandwiches, pizzas and similar	no
5839 Hamburgare stor, Big tasty, super cheese n bacon	Hamburger, large (Big Tasty / Super Cheese & Bacon)	1.00	1.00	1.00	1.00	1.00	no	Sandwiches, pizzas and similar	no
5840 Hamburgare liten	Hamburger, small	0.00	1.00	1.00	1.00	1.00	no	Sandwiches, pizzas and similar	no
5841 Hamburgare utan bröd	Hamburger, without bun	0.00	1.00	1.00	1.00	1.00	no	Sandwiches, pizzas and similar	no
5847 Gris m marinad tillagad typ Pulled pork	Pork, marinated and cooked (Pulled pork type)	0.00	1.00	1.00	1.00	1.00	yes	Meat dish	no
5850 Dumplings m fläsk räkor	Dumplings, with pork and shrimp	1.00	1.00	1.00	1.00	1.00	no	Savoury snacks	no
5852 Glasstårta rund	Ice cream cake, round	1.00	1.00	1.00	1.00	1.00	x	Dairy based desserts	no
5853 Pulled beef	Pulled beef	0.00	1.00	1.00	1.00	1.00	yes	Meat dish	no
5859 Tropisk juice drickf	Tropical juice, ready to drink	0.00	1.00	1.00	1.00	1.00	x	Juices	yes
5860 Sojabönor färska förvällda u. skal	Soybeans, fresh, blanched, shelled	0.00	0.00	0.00	0.00	0.00	no	Nuts and legumes	no
5862 Kålrot kokt	Rutabaga, boiled	0.00	0.00	0.00	0.00	0.00	yes	Vegetables and vegetable products	no
5873 Mjölkdryck extra protein 0,5% fett	Milk drink, high protein, 0.5% fat	1.00	1.00	1.00	1.00	1.00	yes	Milk, yoghurt and similar	yes
5874 Mjölkshake extra protein 1,5 % fett	Milkshake, high protein, 1.5% fat	1.00	1.00	1.00	1.00	1.00	no	Milk, yoghurt and similar	yes
5875 Kyckling m marinad tillagad typ Pulled chicken	Chicken, marinated and cooked (Pulled chicken type)	0.00	1.00	1.00	1.00	1.00	yes	Meat dish	no
5880 Krustader fyllda skagenröra svampstuvning	Pastry shells, filled with Skagen mix or mushroom stew	0.00	0.72	0.72	0.72	0.72	no	Savoury snacks	no
5903 Quesadilla m. kyckling	Quesadilla, with chicken	0.47	0.48	0.49	0.49	1.00	no	Sandwiches, pizzas and similar	no
5904 Quesadilla m. bönor	Quesadilla, with beans	0.46	0.47	0.48	0.48	1.00	yes	Sandwiches, pizzas and similar	no
5906 Dip till pommes frites bearnaise	Dip, Béarnaise, for fries	1.00	1.00	1.00	1.00	1.00	no	Savoury sauces and condiments	no
5908 Dip till kyckling nuggets	Dip, for chicken nuggets	1.00	1.00	1.00	1.00	1.00	no	Savoury sauces and condiments	no
5911 Pizzatopping sås	Pizza topping, sauce	0.00	1.00	1.00	1.00	1.00	no	Savoury sauces and condiments	no
5912 Pizza hemlagad	Pizza, homemade	0.00	0.00	0.00	0.00	0.00	no	Sandwiches, pizzas and similar	no
5913 Pizza kyl/frysvara	Pizza, chilled/frozen	0.00	1.00	1.00	1.00	1.00	no	Sandwiches, pizzas and similar	no
5927 Tacosallad	Taco salad	0.00	0.00	0.00	0.00	1.00	no	Soups and salads	no
5928 Burrito	Burrito	0.33	0.40	0.40	0.41	1.00	no	Sandwiches, pizzas and similar	no
5929 Nachotallrik	Nacho plate	0.00	0.00	0.00	0.00	1.00	no	Savoury snacks	no
5930 Nachotallrik stor	Nacho plate, large	0.00	0.00	0.00	0.00	1.00	no	Savoury snacks	no
5931 Enchiladas	Enchiladas	0.36	0.37	0.37	0.37	1.00	no	Sandwiches, pizzas and similar	no
5932 Quesadillas	Quesadillas	0.47	0.48	0.48	0.49	1.00	no	Sandwiches, pizzas and similar	no

5956	Havregurt naturell fett 2,2% berikad	Oat yoghurt, plain, 2.2% fat, fortified	1.00	1.00	1.00	1.00	1.00	no	Meat and dairy imitations	no
5957	Fraiche m. havre veg. fett 15% berikad	Crème fraîche, oat-based, vegan, 15% fat, fortified	1.00	1.00	1.00	1.00	1.00	no	Meat and dairy imitations	no
5963	Havredryck m. apelsin mango	Oat drink, with orange and mango	1.00	1.00	1.00	1.00	1.00	x	Meat and dairy imitations	yes
5966	Korv falukorv tillagad kött 58%	Sausage, Falukorv, cooked, 58% meat	1.00	1.00	1.00	1.00	1.00	no	Meat	no
5968	Blodpudding blodkorv tillagad	Blood pudding / blood sausage, cooked	1.00	1.00	1.00	1.00	1.00	no	Meat	no
5969	Gris sidfläsk el. stekfläsk rimmat stekt	Pork, side or roast, cured and panfried	1.00	1.00	1.00	1.00	1.00	no	Meat	no
5974	Bröd vitt vetetortilla	Bread, white wheat tortilla	1.00	1.00	1.00	1.00	1.00	no	Bread	no
5993	Flingor med röda bär	Cereal, with red berries	1.00	1.00	1.00	1.00	1.00	no	Breakfast cereals	no
5994	Vitlökssås	Garlic sauce	0.00	1.00	1.00	1.00	1.00	no	Savoury sauces and condiments	no
5996	Bönpasta	Pasta, bean-based	0.00	0.00	0.00	0.00	0.00	no	Nuts and legumes	no
6005	Gris fläskfilé tillagad	Pork fillet, cooked	0.00	0.00	0.00	0.00	0.00	yes	Meat	no
6010	Korv veg. soja- och veteprotein tillagad typ middagskorv	Sausage, vegetarian (soy and wheat protein), cooked (dinner sausage type)	1.00	1.00	1.00	1.00	1.00	no	Meat and dairy imitations	no
6013	Kycklinggryta i currysås	Chicken stew, in curry sauce	0.02	0.06	0.06	0.06	0.12	no	Meat dish	no
6015	Lammgryta med curry	Lamb stew, with curry	0.01	0.06	0.10	0.11	1.00	no	Meat dish	no
6040	Yoghurt smaksatt helfet	yoghurt, flavored, full-fat	1.00	1.00	1.00	1.00	1.00	no	Milk, yoghurt and similar	no
6041	Yoghurt smaksatt lätt	yoghurt, flavored, low-fat	1.00	1.00	1.00	1.00	1.00	no	Milk, yoghurt and similar	no
6042	Kokosvatten smaksatt el naturell	Coconut water, flavored or plain	0.00	0.00	0.00	0.00	1.00	x	Juices	yes
6052	Pastasås m. lax crème fraiche grädde dill	Pasta sauce, with salmon, crème fraîche, cream, dill	0.01	0.03	0.04	0.04	0.08	no	Fish dishes	no
6053	Cashewnötter rostade m. salt	Cashew nuts, roasted with salt	0.00	0.00	0.00	0.00	0.00	x	Nuts and legumes	no
6069	Drickyoghurt smaksatt fett ca 1%	Drinking yoghurt, flavored, approx. 1% fat	1.00	1.00	1.00	1.00	1.00	no	Milk, yoghurt and similar	yes
6081	Hårt bröd fullkorn råg fiber ca 20% typ sport	Crispbread, whole grain rye, approx. 20% fiber (Sport type)	0.00	0.00	0.00	0.00	0.00	yes	Bread	no
6083	Bröd vitt grovt m. nyckelhål typ formfranska	Bread, white, coarse, Keyhole label (loaf type)	1.00	1.00	1.00	1.00	1.00	yes	Bread	no
6084	Bröd vitt typ levain	Bread, white (Levain type)	0.00	1.00	1.00	1.00	1.00	no	Bread	no
6089	Kvarg drickf. olika smaker	Quark drink, various flavors	1.00	1.00	1.00	1.00	1.00	no	Milk, yoghurt and similar	yes
6126	Nori	Nori (seaweed)	0.00	0.00	0.00	0.00	0.00	no	Vegetables and vegetable products	no
6133	Ostkräm	Cheese spread	1.00	1.00	1.00	1.00	1.00	no	Savoury sauces and condiments	no
6145	Matvete kokt m. salt	Wheat grain, cooked with salt	0.00	0.00	0.00	0.00	0.00	yes	Cereals	no
6146	Mathavre kokt m. salt	Barley grain, cooked with salt	0.00	0.00	0.00	0.00	0.00	yes	Cereals	no
6157	Chiafrö	Chia seeds	0.00	0.00	0.00	0.00	0.00	yes	Nuts and legumes	no
6164	Senap sötstark	Mustard, sweet and strong	0.00	0.00	0.00	0.00	0.00	no	Savoury sauces and condiments	no
6165	Kokosolja	Coconut oil	0.00	0.00	0.00	0.00	0.00	no	Fat	no
6167	Majonnäs m. solrosolja fett 80%	Mayonnaise, with sunflower oil, 80% fat	0.00	1.00	1.00	1.00	1.00	no	Savoury sauces and condiments	no
6168	Kräfta kräftstjärtar konserv. u. lag	Crayfish tails, canned, drained	0.00	1.00	1.00	1.00	1.00	yes	Fish and fish products	no
6170	Påläggskaviar m. färskost	Spreadable caviar, with cream cheese	1.00	1.00	1.00	1.00	1.00	no	Savoury sauces and condiments	no
6172	Tångkaviar påläggskaviar	Seaweed caviar, spreadable	1.00	1.00	1.00	1.00	1.00	no	Meat and dairy imitations	no
6225	Lax odlad Norge fjordlax rå förpackad	Salmon, farmed (Norwegian fjord), raw, packaged	0.00	0.00	0.00	0.00	0.00	yes	Fish and fish products	no
6245	Barnmat pasta couscous m. kyckling konserv.	Baby food, pasta/couscous with chicken, canned	0.00	0.00	0.00	0.00	0.00	yes	Children's food	no
6246	Barnmat spagetti m. köttfärssås konserv.	Baby food, spaghetti with meat sauce, canned	0.00	0.00	0.00	0.00	0.00	yes	Children's food	no
6247	Barnmat potatis m. nötköttsgryta konserv.	Baby food, potatoes with beef stew, canned	0.00	0.00	0.00	0.00	0.00	no	Children's food	no
6248	Barnmat pastagratäng m. fläskkött konserv.	Baby food, pasta gratin with pork, canned	0.00	0.00	0.00	0.00	0.00	no	Children's food	no
6249	Barnmat fiskgryta konserv.	Baby food, fish stew, canned	1.00	1.00	1.00	1.00	1.00	yes	Children's food	no
6251	Barnmat lasagne pastagratäng veg. m. grönsaker konserv.	Baby food, vegetarian lasagna/pasta gratin with vegetables, canned	0.00	0.00	0.00	0.00	0.00	no	Children's food	no
6252	Barnmat frukt bär m. yoghurt konserv.	Baby food, fruit and berries with yoghurt, canned	1.00	1.00	1.00	1.00	1.00	yes	Children's food	no
6257	Välling för barn drickf. Fullkorn osötad berikad	Baby porridge drink, whole grain, unsweetened, fortified	1.00	1.00	1.00	1.00	1.00	no	Children's cereal products	yes
6259	Välling för barn drickf. mild havre osötad berikad	Baby porridge drink, mild oat, unsweetened, fortified	1.00	1.00	1.00	1.00	1.00	no	Children's cereal products	yes
6260	Välling för barn drickf. majs osötad berikad	Baby porridge drink, corn, unsweetened, fortified	1.00	1.00	1.00	1.00	1.00	no	Children's cereal products	yes
6261	Välling för barn drickf. Havre mjölkfri osötad berikad	Baby porridge drink, oat, milk-free, unsweetened, fortified	1.00	1.00	1.00	1.00	1.00	no	Children's cereal products	yes
6262	Barngröt ätf. havre m. banan äpple mjölkfri osötad berikad	Baby porridge, oat with banana and apple, milk-free, unsweetened, fortified	1.00	1.00	1.00	1.00	1.00	no	Children's cereal products	no
6263	Barngröt ätf. naturell fullkorn osötad berikad	Baby porridge, plain whole grain, unsweetened, fortified	1.00	1.00	1.00	1.00	1.00	no	Children's cereal products	no
6282	Mykoprotein färs bitar filé stekt	Mycoprotein, mince/pieces/fillet, panfried	1.00	1.00	1.00	1.00	1.00	yes	Meat and dairy imitations	no
6288	Sojaprotein bitar stekta typ Tzay®	Soy protein, pieces, panfried (Tzay® type)	1.00	1.00	1.00	1.00	1.00	no	Meat and dairy imitations	no
6293	Ärtprotein färs stekt	Pea protein, mince, panfried	1.00	1.00	1.00	1.00	1.00	no	Meat and dairy imitations	no
6313	Mykoprotein schnitzel ugnsstekt	Schnitzel, mycoprotein, oven-baked	1.00	1.00	1.00	1.00	1.00	no	Meat and dairy imitations	no
6314	Äggstanning	Egg mixture	0.00	0.00	0.00	0.00	0.00	no	Egg dishes	no
6336	Dressing hamburgerdressing lätt fett 28 %	Dressing, hamburger style, light, 28% fat	1.00	1.00	1.00	1.00	1.00	no	Savoury sauces and condiments	no
6337	Majonnäs äggfri	Mayonnaise, egg-free	1.00	1.00	1.00	1.00	1.00	no	Savoury sauces and condiments	no
6338	Sås mango curry m. majonnäs kylvara	Sauce, mango curry with mayonnaise, chilled	1.00	1.00	1.00	1.00	1.00	no	Savoury sauces and condiments	no

6341	Ostsås m. kalkon	Cheese sauce, with turkey	0.16	0.19	0.22	0.23	0.44	no	Meat dish	no
6342	Lasagne m. zucchini aubergine nötfärs	Lasagna, with zucchini, eggplant, and ground beef	0.02	0.08	0.08	0.08	0.15	no	Meat dish	no
6353	Mango currysås hemlagad	Mango curry sauce, homemade	0.00	0.00	0.00	0.00	0.00	no	Savoury sauces and condiments	no
6356	Cowboysoppa m. potatis köttfärs	Cowboy soup, with potatoes and minced meat	0.02	0.03	0.03	0.03	1.00	no	Soups and salads	no
6364	Färsruta m. fetaost ugnsstekt	Mince square, with feta cheese, oven-baked	0.00	0.00	0.00	0.00	0.00	no	Meat dish	no
6366	Fisk cornflakesfisk panerad ugnsstekt	Fish, breaded with cornflakes, oven-baked	0.00	0.17	0.22	0.24	1.00	yes	Fish dishes	no
6368	Rödbetshummus	Beetroot hummus	0.00	0.00	0.00	0.00	1.00	no	Savoury sauces and condiments	no
6372	Chili- och mangosås	Chili and mango sauce	0.00	0.00	0.00	0.00	0.00	no	Savoury sauces and condiments	no
6374	Bondomelett m. bönor	Farmer’s omelette, with beans	0.15	0.18	0.20	0.21	0.30	no	Egg dishes	no
6377	Gräddsås m. persilja	Cream sauce, with parsley	0.00	0.00	0.00	0.00	1.00	no	Savoury sauces and condiments	no
6385	Gratäng m. kassler ananas ost	Gratin, with smoked pork, pineapple, and cheese	0.62	0.62	0.62	0.62	0.62	no	Meat dish	no
6389	Gryta fisk m. crème fraiche curry apelsin	Fish stew, with crème fraîche, curry, and orange	0.19	0.19	0.19	0.19	0.19	no	Fish dishes	no
6393	Köttfärslåda	Minced meat casserole	0.02	0.08	0.08	0.08	0.15	no	Meat dish	no
6404	Gryta chili sin carne m. zucchini paprika veg.	Chili sin carne stew, with zucchini and bell pepper, vegetarian	0.03	0.09	0.09	0.09	0.17	yes	Vegetable dishes	no
6405	Gryta chili sin carne m. mykoprotein veg.	Chili sin carne stew, with mycoprotein, vegetarian	0.19	0.22	0.24	0.25	0.32	no	Vegetable dishes	no
6407	Mykoproteinfärssås veg.	Minced mycoprotein sauce, vegetarian	0.47	0.51	0.54	0.56	0.68	no	Vegetable dishes	no
6410	Halloumi stroganoff veg.	Halloumi stroganoff, vegetarian	0.00	0.16	0.16	0.17	0.20	no	Vegetable dishes	no
6411	Gryta halloumigryta m. aubergine veg.	Halloumi stew, with eggplant, vegetarian	0.02	0.06	0.06	0.06	0.12	no	Vegetable dishes	no
6417	Bön stroganoff veg.	Bean stroganoff, vegetarian	0.00	0.29	0.31	0.32	0.38	no	Legume dishes	no
6418	Gryta m. gröna linser rotfrukter kokosmjölk veg.	Stew, with green lentils, root vegetables, and coconut milk, vegetarian	0.00	0.45	0.46	0.46	0.49	no	Legume dishes	no
6420	Grönsaker ugnsstekta	Vegetables, oven-roasted	0.00	0.15	0.15	0.15	0.35	no	Vegetables and vegetable products	no
6424	Broccolisås	Broccoli sauce	0.00	0.00	0.00	0.00	0.00	no	Vegetable dishes	no
6426	Sojafärssås veg.	Soy mince sauce, vegetarian	0.59	0.63	0.65	0.66	0.75	no	Legume dishes	no
6427	Sojafärssås m. linser veg.	Soy mince sauce, with lentils, vegetarian	0.49	0.52	0.54	0.55	0.63	no	Legume dishes	no
6429	Tikka masala m. potatis grönsaker veg.	Tikka masala, with potatoes and vegetables, vegetarian	0.00	0.05	0.10	0.12	0.27	no	Vegetable dishes	no
6433	Gryta currygryta m. grönsaker veg.	Curry stew, with vegetables, vegetarian	0.03	0.09	0.09	0.09	0.17	no	Vegetable dishes	no
6434	Pasta carbonara m. pasta sojabönor veg.	Pasta carbonara, with pasta and soybeans, vegetarian	0.00	0.00	0.00	0.00	0.00	no	Pasta dishes	no
6440	Morotssås	Carrot sauce	0.00	0.53	0.55	0.56	0.64	no	Vegetable dishes	no
6441	Ajvar- och spenatsås	Ajvar and spinach sauce	0.00	0.30	0.30	0.30	0.30	no	Savoury sauces and condiments	no
6442	Paprika grillad	Bell pepper, grilled	0.00	0.00	0.00	0.00	0.00	yes	Vegetables and vegetable products	no
6443	Tomatsås m. grädde ospec.	Tomato sauce, with cream, unspecified	0.02	0.07	0.07	0.07	0.17	no	Vegetable dishes	no
6446	Lasagne m. cottage cheese veg.	Lasagna, with cottage cheese, vegetarian	0.02	0.08	0.08	0.08	0.15	no	Pasta dishes	no
6447	Paj m. fetaost sötpotatis veg.	Pie, with feta cheese and sweet potato, vegetarian	0.00	0.00	0.00	0.00	0.00	no	Sandwiches, pizzas and similar	no
6452	Vegestronesoppa veg.	Vegestrone soup, vegetarian	0.04	0.12	0.12	0.12	0.24	no	Soups and salads	no
6453	Gratäng m. mykoprotein ananas veg.	Gratin, with mycoprotein and pineapple, vegetarian	0.56	0.56	0.56	0.56	0.56	no	Vegetable dishes	no
6454	Pumpasoppa veg.	Pumpkin soup, vegetarian	0.08	0.13	0.17	0.19	0.34	yes	Soups and salads	no
6455	Gryta chiligryta m. bönor linser sojakorv veg.	Chili stew, with beans, lentils, and soy sausage, vegetarian	0.00	0.09	0.09	0.09	0.09	no	Vegetable dishes	no
6457	Pastasås m. kyckling paprika	Pasta sauce, with chicken and bell pepper	0.00	0.00	0.00	0.00	0.00	no	Meat dish	no
6459	Köttfärssås Vivaldi nöt m. squash majs paprika rödlök ost	Meat sauce, Vivaldi style, with beef, squash, corn, bell pepper, red onion, and cheese	0.04	0.06	0.07	0.08	0.14	no	Meat dish	no
6461	Chiapudding	Chia pudding	0.00	0.57	0.57	0.57	1.00	x	Dairy based desserts	no
6462	Korv stroganoff hemlagad	Sausage stroganoff, homemade	0.62	0.63	0.64	0.64	0.97	no	Meat dish	no
6463	Lasagne hemlagad	Lasagna, homemade	0.02	0.08	0.08	0.08	0.15	no	Pasta dishes	no
6470	Lasagne pastagratäng m. grönsaker	Lasagna/pasta gratin, with vegetables	0.02	0.08	0.08	0.08	0.15	no	Pasta dishes	no
6471	Potatisgratäng m. kött	Potato gratin, with meat	0.00	0.00	0.00	0.00	0.00	no	Potato dishes	no
6472	Pastagratäng lasagne m. kyckling	Pasta gratin/lasagna, with chicken	0.02	0.08	0.08	0.08	0.15	no	Pasta dishes	no
6473	Lasagne pastagratäng m. baljväxter	Lasagna/pasta gratin, with legumes	0.02	0.08	0.08	0.08	0.15	no	Pasta dishes	no
6480	Kornflingor ångprep. fullkorn	Barley flakes, steam-prepared, whole grain	0.00	0.00	0.00	0.00	0.00	yes	Cereals	no
6481	Rågflingor ångprep. fullkorn	Rye flakes, steam-prepared, whole grain	0.00	0.00	0.00	0.00	0.00	yes	Cereals	no
6495	Kvarg smaksatt fett 1,5-7%	Quark, flavored, 1.5–7% fat	1.00	1.00	1.00	1.00	1.00	no	Milk, yoghurt and similar	no
6496	Bröstmjölk humanmjölk flaskmatning	Breast milk, human, bottle-fed	0.00	0.00	0.00	0.00	0.00	x	Milk, yoghurt and similar	yes
6498	Soja- och veteprotein nugget tillagad	Nugget, soy and wheat protein, cooked	1.00	1.00	1.00	1.00	1.00	no	Meat and dairy imitations	no
6499	Soja- och veteprotein färs tillagad	Minced meat, soy and wheat protein, cooked	1.00	1.00	1.00	1.00	1.00	no	Meat and dairy imitations	no
6500	Soja- och veteprotein bullar tillagade	Balls, soy and wheat protein, cooked	1.00	1.00	1.00	1.00	1.00	no	Meat and dairy imitations	no
6501	Mykoprotein nugget tillagad	Nugget, mycoprotein, cooked	1.00	1.00	1.00	1.00	1.00	no	Meat and dairy imitations	no
6502	Soja- och veteprotein schnitzel tillagad	Schnitzel, soy and wheat protein, cooked	1.00	1.00	1.00	1.00	1.00	no	Meat and dairy imitations	no

6505	Grönsaksbullar m. kikärtor morot tillagade u. matfett veg.	Vegetable balls, with chickpeas and carrot, cooked without fat, vegetarian	0.00	1.00	1.00	1.00	1.00	no	Vegetable dishes	no
6506	Grönsaksbiff jordnötsbiff tillagad veg.	Vegetable patty, peanut patty, cooked, vegetarian	0.00	0.00	0.00	0.00	0.00	no	Vegetable dishes	no
6507	Grönsaksbiff grön ärtbiff tillagad u. matfett veg.	Vegetable patty, green pea patty, cooked without fat, vegetarian	0.00	1.00	1.00	1.00	1.00	no	Vegetable dishes	no
6512	Chèvrecrème	Chèvre cream	0.00	0.00	0.00	0.00	0.00	no	Savoury sauces and condiments	no
6516	Vitkålssallad m. morot	White cabbage salad, with carrot	0.00	0.00	0.00	0.00	0.00	yes	Vegetables and vegetable products	no
6520	Barnmat grönsaksgryta m. kyckling vitlök konserv.	Baby food, vegetable stew with chicken and garlic, canned	0.00	0.00	0.00	0.00	0.00	no	Children's food	no
6522	Barnmat potatis m. kyckling konserv.	Baby food, potatoes with chicken, canned	0.00	0.00	0.00	0.00	0.00	no	Children's food	no
6523	Barnmat ris m. nötkött konserv.	Baby food, rice with beef, canned	0.00	0.00	0.00	0.00	0.00	no	Children's food	no
6525	Barnmat potatis m. fläskkött konserv.	Baby food, potatoes with pork, canned	0.00	0.00	0.00	0.00	0.00	yes	Children's food	no
6528	Barnmat grönsaker m. quinoa/linser veg. konserv.	Baby food, vegetables with quinoa/lentils, vegetarian, canned	0.00	0.00	0.00	0.00	0.00	yes	Children's food	no
6529	Barnmat blåbär äpple konserv.	Baby food, blueberry and apple, canned	1.00	1.00	1.00	1.00	1.00	no	Children's food	no
6530	Barnmat katrinplommon päron äpple konserv.	Baby food, prunes, pear, and apple, canned	0.00	0.00	0.00	0.00	0.00	x	Children's food	no
6531	Barnmat banan exotiska frukter konserv.	Baby food, banana and exotic fruits, canned	1.00	1.00	1.00	1.00	1.00	x	Children's food	no
6532	Barnmat morot konserv.	Baby food, carrot, canned	0.00	0.00	0.00	0.00	0.00	yes	Children's food	no
6535	Grönsaksbiff morot linser	Vegetable patty, carrot and lentils	0.00	1.00	1.00	1.00	1.00	no	Vegetable dishes	no
6536	Grönsaksbiff m. ost	Vegetable patty, with cheese	0.00	1.00	1.00	1.00	1.00	no	Vegetable dishes	no
6537	Barnmat klämmis m. banan jordgubb gröt konserv.	Baby food pouch, banana and strawberry porridge, canned	0.00	1.00	1.00	1.00	1.00	x	Children's cereal products	no
6538	Barnmat klämmis m äpple blåbär gröt konserv.	Baby food pouch, apple and blueberry porridge, canned	0.00	1.00	1.00	1.00	1.00	x	Children's cereal products	no
6539	Barnmat klämmis m. frukt gröt konserv.	Baby food pouch, fruit porridge, canned	0.00	1.00	1.00	1.00	1.00	x	Children's cereal products	no
6544	Barnmat frukt konserv.	Baby food, fruit, canned	0.00	1.00	1.00	1.00	1.00	x	Children's food	no
6545	Pastagratäng m. skinka	Pasta gratin, with ham	0.02	0.02	0.02	0.02	0.02	no	Pasta dishes	no
6547	Falafel kikärtskroketter tillagde	Falafel, chickpea croquettes, cooked	0.00	0.00	0.00	0.00	1.00	no	Legume dishes	no
6549	Fruktstång fruktgodis ospec.	Fruit bar, fruit candy, unspecified	1.00	1.00	1.00	1.00	1.00	x	Sugar and confectionery	no
6552	Modersmjölksersättning	Infant formula	1.00	1.00	1.00	1.00	1.00	x	Formulae	yes
6553	Tillskottsnäring för småbarn	Supplementary nutrition, for toddlers	1.00	1.00	1.00	1.00	1.00	x	Formulae	yes
6555	Bröd mörkt fullkorn >30%	Bread, dark, whole grain >30%	0.00	1.00	1.00	1.00	1.00	yes	Bread	no
6565	Välling för barn drickf. havre majs osötad berikad	Baby porridge drink, oat and corn, unsweetened, fortified	1.00	1.00	1.00	1.00	1.00	no	Children's cereal products	yes
6567	Välling för barn drickf. fullkorn m. frukt osötad berikad	Baby porridge drink, whole grain with fruit, unsweetened, fortified	1.00	1.00	1.00	1.00	1.00	no	Children's cereal products	yes
6568	Barnkex m. fruktsmak	Baby biscuits, fruit-flavored	1.00	1.00	1.00	1.00	1.00	no	Children's cereal products	no
6583	Köttfärsgratäng	Minced meat gratin	0.02	0.08	0.08	0.08	0.15	no	Meat dish	no
6584	Paj m. vegetabilisk köttsubstitut veg.	Pie, with plant-based meat substitute, vegetarian	0.22	1.00	1.00	1.00	1.00	no	Sandwiches, pizzas and similar	no
6598	Grönsaksbiff ospec. grönsaker m. baljväxter	Vegetable patty, unspecified vegetables with legumes	0.00	1.00	1.00	1.00	1.00	no	Legume dishes	no
6599	Varm choklad m. havredryck sojadryck mandeldryck	Hot chocolate, with oat/soy/almond drink	1.00	1.00	1.00	1.00	1.00	no	Coffee, tea and similar	yes
6600	Viltfilé stekt	Game fillet, panfried	0.00	0.00	0.00	0.00	0.00	yes	Meat	no
6601	Gryta fiskgryta m. kokosmjölk	Fish stew, with coconut milk	0.00	0.69	0.69	0.69	0.69	no	Fish dishes	no
6602	Gryta grönsaksgryta m. baljväxter tomatbas	Vegetable stew, with legumes, tomato-based	0.03	0.09	0.09	0.09	0.17	yes	Legume dishes	no
6603	Gryta grönsaksgryta m. baljväxter veg.	Vegetable stew, with legumes, vegetarian	0.03	0.09	0.09	0.09	0.17	yes	Legume dishes	no
6604	Gryta grönsaksgryta m. baljväxter mejeriprodukter	Vegetable stew, with legumes and dairy	0.02	0.06	0.06	0.06	0.12	no	Legume dishes	no
6605	Gryta köttgryta m. mejeriprodukter	Meat stew, with dairy	0.02	0.06	0.06	0.06	0.12	no	Meat dish	no
6606	Gryta indisk köttgryta	Indian meat stew	0.02	0.09	0.09	0.09	0.17	no	Meat dish	no
6608	Ananas ospec. konserv.	Pineapple, unspecified, canned	0.00	0.00	0.00	0.00	0.00	x	Fruit products	no
6609	Pannbiff vilt stekt	Patty, game meat, panfried	0.00	0.00	0.00	0.00	0.00	no	Meat dish	no
6610	Köttfärssås vilt	Meat sauce, game meat	0.02	0.06	0.06	0.06	0.12	no	Meat dish	no
6611	Vilt färs m. salt stekt	Minced game meat, with salt, panfried	0.00	0.04	0.04	0.04	0.12	yes	Meat	no
6612	Gryta viltgryta m. grädde	Game stew, with cream	0.02	0.06	0.06	0.06	0.12	no	Meat dish	no
6613	Gryta indisk gryta m. soja mykoprotein	Indian stew, with soy and mycoprotein	0.47	0.55	0.61	0.63	0.85	no	Vegetable dishes	no
6614	Gryta mykoprotein m. mejeriprodukter	Mycoprotein stew, with dairy	0.77	0.79	0.81	0.82	0.90	no	Vegetable dishes	no
6615	Kycklingpanna ospec.	Chicken pan dish, unspecified	0.02	0.06	0.06	0.06	0.12	no	Meat dish	no
6616	Gryta kycklinggryta m. mejeriprodukter	Chicken stew, with dairy	0.02	0.06	0.06	0.06	0.12	no	Meat dish	no
6617	Köttsoppa m. buljongbas	Meat soup, broth-based	0.04	0.12	0.12	0.12	0.24	no	Soups and salads	no
6619	Grönsakssoppa m. kokosmjölk	Vegetable soup, with coconut milk	0.00	0.66	0.66	0.66	0.66	no	Soups and salads	no
6620	Grönsakssoppa m. mejeriprodukter	Vegetable soup, with dairy	0.04	0.09	0.09	0.09	0.17	yes	Soups and salads	no
6621	Pannbiff veg.	Patty, vegetarian	1.00	1.00	1.00	1.00	1.00	no	Meat dish	no
6622	Fiskgratäng m. sås u. potatismos	Fish gratin, with sauce, without mashed potatoes	0.02	0.08	0.08	0.08	0.15	no	Fish dishes	no
6623	Fisk m. ströbrödstäcke	Fish, with breadcrumb crust	0.00	0.10	0.10	0.10	0.25	yes	Fish dishes	no
6624	Pizza Vesuvio restaurang	Pizza, Vesuvio (restaurant)	0.10	0.50	0.50	0.50	1.00	no	Sandwiches, pizzas and similar	no
6625	Pizza Capricciosa restaurang	Pizza, Capricciosa (restaurant)	0.10	0.50	0.50	0.50	1.00	no	Sandwiches, pizzas and similar	no
6626	Pizza veg. restaurang	Pizza, vegetarian (restaurant)	0.00	0.50	0.50	0.50	1.00	no	Sandwiches, pizzas and similar	no
6627	Pizza Quattro stagioni restaurang	Pizza, Quattro Stagioni (restaurant)	0.09	0.50	0.50	0.50	1.00	no	Sandwiches, pizzas and similar	no

6628	Pizza m. kött restaurang	Pizza, with meat (restaurant)	0.09	0.50	0.50	0.50	1.00	no	Sandwiches, pizzas and similar	no
6629	Pizza Bussola restaurang	Pizza, Bussola (restaurant)	0.10	0.50	0.50	0.50	1.00	no	Sandwiches, pizzas and similar	no
6630	Pizza m. köttfärs restaurang	Pizza, with minced meat (restaurant)	0.00	0.50	0.50	0.50	1.00	no	Sandwiches, pizzas and similar	no
6631	Pizza m. salami restaurang	Pizza, with salami (restaurant)	0.15	0.50	0.50	0.50	1.00	no	Sandwiches, pizzas and similar	no
6633	Laxfiskar u. lax kokt m. salt	Salmon-type fish, excluding salmon, boiled with salt	0.00	0.00	0.00	0.00	0.00	yes	Fish and fish products	no
6640	Kanelbulle ospec.	Cinnamon bun, unspecified	0.00	1.00	1.00	1.00	1.00	x	Fine bakery wares	no
6642	Hårt bröd glutenfritt ospec.	Crispbread, gluten-free, unspecified	1.00	1.00	1.00	1.00	1.00	no	Bread	no
6643	Lösgodis påsgodis styckvis ospec.	Pick-and-mix candy, individually wrapped, unspecified	1.00	1.00	1.00	1.00	1.00	x	Sugar and confectionery	no
6646	D-droppar i vatten	Vitamin D drops, in water	1.00	1.00	1.00	1.00	1.00	x	Supplements	no
6649	Linsbågar bönbågar	Lentil puffs / Bean puffs	1.00	1.00	1.00	1.00	1.00	x	Savoury snacks	no
6650	Linschips bönchips	Lentil chips / Bean chips	1.00	1.00	1.00	1.00	1.00	x	Savoury snacks	no
6651	Rapsolja berikad	Rapeseed oil, fortified	0.00	0.00	0.00	0.00	0.00	yes	Fat	no
6653	Frukostflingor müsli original m. russin frön Risenta	Breakfast cereal, original muesli with raisins and seeds (Risenta)	1.00	1.00	1.00	1.00	1.00	no	Breakfast cereals	no
6654	Kex bokstavskex fullkorn ospec.	Biscuits, alphabet-shaped, whole grain, unspecified	1.00	1.00	1.00	1.00	1.00	no	Fine bakery wares	no
6674	Ostronsås	Oyster sauce	1.00	1.00	1.00	1.00	1.00	no	Savoury sauces and condiments	no
6675	Fisksås	Fish sauce	1.00	1.00	1.00	1.00	1.00	no	Savoury sauces and condiments	no
6678	Kiwi gul	Kiwi, yellow	0.00	0.00	0.00	0.00	0.00	yes	Fruit	no
6703	Jordnötter m. chokladöverdrag	Peanuts, chocolate-coated	1.00	1.00	1.00	1.00	1.00	x	Nuts and legumes	no
6704	Chokladkex m. vaniljfyllning	Chocolate biscuit, with vanilla filling	1.00	1.00	1.00	1.00	1.00	no	Fine bakery wares	no
6708	Barnmat klämmis smoothie u. mjölk konserv. ospec.	Baby food pouch, smoothie without milk, canned, unspecified	0.00	1.00	1.00	1.00	1.00	x	Children's food	no
6742	Korv falukorv ugnsstekt ospec.	Sausage, Falukorv, oven-baked, unspecified	1.00	1.00	1.00	1.00	1.00	no	Meat	no
6750	Pannkaka u. mjölk ägg	Pancake, without milk or egg	0.20	0.25	0.28	0.29	0.42	no	Fine bakery wares	no
6751	Pastasallad ospec.	Pasta salad, unspecified	0.00	0.00	0.00	0.00	1.00	no	Soups and salads	no
6752	Korvgryta veg.	Sausage stew, vegetarian	0.75	0.77	0.78	0.79	0.84	no	Meat dish	no
6753	Soja- och veteprotein baconimitation ospec.	Bacon imitation, soy and wheat protein, unspecified	1.00	1.00	1.00	1.00	1.00	no	Meat and dairy imitations	no
6755	Alger ätf. ospec.	Edible algae, unspecified	0.00	1.00	1.00	1.00	1.00	no	Vegetables and vegetable products	no
6756	Korv kryddkorv ospec.	Sausage, spicy, unspecified	1.00	1.00	1.00	1.00	1.00	no	Meat	no
6757	Bröd rågsikt fibrer ca 4% typ rågkaka	Bread, rye flour, approx. 4% fiber (rye cake type)	1.00	1.00	1.00	1.00	1.00	no	Bread	no
6759	Bröd fröbröd fullkorn vete råg	Bread, seed bread, whole grain wheat and rye	0.00	1.00	1.00	1.00	1.00	no	Bread	no
6760	Pizza m. tomatsås ost restaurang	Pizza, with tomato sauce and cheese (restaurant)	0.00	0.50	0.50	0.50	1.00	no	Bread	no
6762	Pommes frites smaksatta tillagad frysvara	French fries, flavored, cooked, frozen	1.00	1.00	1.00	1.00	1.00	no	Savoury snacks	no
6764	Frukostflingor granola m. frukt nötter	Breakfast cereal, granola with fruit and nuts	0.00	1.00	1.00	1.00	1.00	no	Breakfast cereals	no
6765	Frukostflingor granola m. kakao hallon	Breakfast cereal, granola with cocoa and raspberries	0.00	1.00	1.00	1.00	1.00	no	Breakfast cereals	no
6766	Majskaka smaksatt ost	Corn cake, cheese-flavored	1.00	1.00	1.00	1.00	1.00	no	Fine bakery wares	no
6767	Linskaka smaksatt	Lentil cake, flavored	1.00	1.00	1.00	1.00	1.00	no	Fine bakery wares	no
6769	Pepparkaksdeg kylvara	Gingerbread dough, chilled	1.00	1.00	1.00	1.00	1.00	x	Fine bakery wares	no
6770	Bröd vitt croissant gräddad kylvara frysvara el. butiksbakad	Bread, white croissant, baked (chilled/frozen/store-baked)	1.00	1.00	1.00	1.00	1.00	no	Fine bakery wares	no
6775	Hamburgare m. bröd	Hamburger, with bun	0.00	1.00	1.00	1.00	1.00	no	Sandwiches, pizzas and similar	no
6776	Korv ospec. kött ≥ 65%	Sausage, unspecified, ≥65% meat	1.00	1.00	1.00	1.00	1.00	no	Meat	no
6777	Kryddor torkade	Spices, dried	0.00	0.00	0.00	0.00	0.00	x	Nuts and legumes	no
6782	Ris sushiris kokt	Rice, sushi rice, cooked	0.00	0.00	0.00	0.00	0.00	no	Cereals	no
6784	Frukostflingor chokladsmak ospec.	Breakfast cereal, chocolate-flavored, unspecified	1.00	1.00	1.00	1.00	1.00	no	Breakfast cereals	no
6785	Injera etiopisk pannkaka	Injera, Ethiopian pancake	0.00	0.00	0.00	0.00	0.00	x	Cereals	no
6786	Nudlar äggnudlar kokta u. salt	Noodles, egg noodles, cooked without salt	0.00	0.00	0.00	0.00	0.00	no	Pasta and similar	no
6795	Havredryck fett 1,5% eko. berikad	Oat drink, 1.5% fat, organic, fortified	0.00	0.00	0.00	0.00	0.00	yes	Meat and dairy imitations	yes
6801	Havregurt naturell fett 11 % berikad	Oat yoghurt, plain, 11% fat, fortified	1.00	1.00	1.00	1.00	1.00	no	Meat and dairy imitations	no
6812	Havregurt smaksatt berikad	Oat yoghurt, flavored, fortified	1.00	1.00	1.00	1.00	1.00	no	Meat and dairy imitations	no
6813	Havredryck fett 3,0% berikad	Oat drink, 3.0% fat, fortified	0.00	1.00	1.00	1.00	1.00	no	Meat and dairy imitations	yes
6814	Glass havreglass smaksatt	Ice cream, oat-based, flavored	1.00	1.00	1.00	1.00	1.00	no	Sugar and confectionery	no
6815	Havrebaserat bredbart pålägg smaksatt	Oat-based spreadable topping, flavored	1.00	1.00	1.00	1.00	1.00	no	Meat and dairy imitations	no
6816	Havredryck m. choklad ospec.	Oat drink, chocolate-flavored, unspecified	1.00	1.00	1.00	1.00	1.00	x	Meat and dairy imitations	no
6833	Sött vetebröd saffransbröd	Sweet wheat bread, saffron bread	0.00	1.00	1.00	1.00	1.00	no	Fine bakery wares	no
