# Supplementary material for: Intake of ultra-processed foods in Sweden and its association with diet quality and weight status: cross-sectional analyses from three national dietary surveys
Source: Nutr J. 2026 Oct 13;25:110. doi: 10.1186/s12937-026-01408-5 (PMC13663064; doi:10.1186/s12937-026-01408-5)
Supplement: Supplementary file 3 [file 12937_2026_1408_MOESM3_ESM.pdf]

Supplement Table S1. Categorisation of food groups according to FoodEx2

**FoodEx2 main group**	**FoodEx2 subgroup**	**Abbreviated name**	**FoodEx2 code**	**Items (n)**	**Entirely UPF n (%)**	**Partly UPF n (%)**
**Total**				**2 218**	**990 (44.6)**	**370 (16.7)**
Grains and grain-based products	Cereal and cereal primary derivatives	Cereals	00020	38	1 (2.6)	0 (0)
	Bread and similar products	Bread	01160	80	54 (67.5)	1 (1.2)
	Pasta, doughs and similar products	Pasta and similar	01740	17	4 (23.5)	2 (11.8)
	Fine bakery wares	Fine bakery wares	02360	125	87 (69.6)	15 (12.0)
	Breakfast cereals	Breakfast cereals	03080	57	40 (70.2)	0 (0)
Vegetables and vegetable products		Vegetables	03580	174	17 (9.8)	33 (19.0)
Starchy roots or tubers and products thereof		Starchy roots and tubers	09950	15	0 (0)	4 (26.7)
Legumes, nuts, oilseeds and spices		Nuts and legumes	10200	57	4 (7.0)	0 (0)
Fruit and Fruit Products	Processed fruit products	Fruit products	17900	81	55 (67.9)	2 (2.5)
	Fruit used as fruit	Fruits	14220	54	0 (0)	0 (0)
Meat and meat products		Meat and meat products	18760	186	72 (38.7)	3 (1.6)
Fish, seafood, amphibians, reptiles and invertebrates		Fish and fish products	22110	79	15 (19.0)	7 (8.9)
Milk and dairy products	Cheese	Cheese	27310	36	11 (30.6)	0 (0)
	Dairy dessert and similar	Dairy desserts	29360	35	30 (85.7)	4 (11.4)
	Milk and dairy products (other)	Milk, yoghurt and similar	26490	86	43 (50)	3 (3.5)
Eggs and egg products		Egg and egg products	29480	8	0 (0)	2 (25)
Sugar and similar, confectionery and water-based sweet desserts		Sugar and confectionery	29810	76	65 (85.5)	4 (5.3)
Animal and vegetable fats and oils and primary derivatives		Fat	30910	39	21 (53.8)	0 (0)
Fruit- and vegetable juices and nectars	Juices (here restricted to 100% fruit juice)	Juices	31570	23	13 (56.5)	1 (4.3)
Water and water-based beverages	Water based beverages (excl. sugar-sweetened beverages)	Water based beverages	32610	11	8 (72.7)	0 (0)

	Sugar-sweetened beverages (not originally a FoodEx2 food group. Includes here soft drinks, cordials and sugar-sweetened fruit drinks)	Sugar-sweetened beverages		21	21 (100)	0 (0)
Alcoholic beverages		Alcoholic beverages	33300	32	23 (71.9)	3 (9.4)
Coffee, cocoa, tea and infusions		Coffee, tea and similar	34020	24	9 (37.5)	0 (0)
Food products for young population	Infant and follow-on formulae	Children’s formulae	37480	2	2 (100)	0 (0)
	Ready to eat meal for infants and young children	Children’s food	37710	18	6 (33.3)	0 (0)
	Processed cereal-based food for infants and young children	Children’s cereal products	37790	21	21 (100)	0 (0)
Products for non-standard diets, food imitations and food supplements	Food supplements and similar preparations	Supplements	38070	12	10 (83.3)	2 (16.7)
	Meat and dairy imitations	Meat and dairy imitations	38250	65	62 (95.4)	0 (0)
Composite dishes	Meat-based dishes	Meat dishes	38480	147	29 (19.7)	71 (48.3)
	Fish and seafood-based dishes	Fish dishes	38610	48	11 (22.9)	22 (45.8)
	Egg based dishes	Egg dishes	38730	13	0 (0)	11 (84.6)
	Potato based dishes	Potato dishes	38800	24	10 (41.7)	7 (29.2)
	Legume based dishes	Legume dishes	38970	25	5 (20)	16 (64.0)
	Vegetable based dishes	Vegetable dishes	39070	76	18 (23.7)	48 (63.2)
	Sandwiches, pizza and other stuffed bread-like cereal products	Sandwiches, pizzas and similar	39180	77	40 (51.9)	32 (41.6)
	Pasta and rice-based dishes	Pasta dishes	39580	51	13 (25.5)	28 (54.9)
	Soups and salads	Soups and salads	39840	99	38 (38.4)	31 (31.3)
	Fried or extruded cereal, seed or root-based products	Savory snacks	40330	39	24 (61.5)	1 (2.6)
Seasoning, sauces and condiments		Savory sauces and condiments	40500	147	102 (69.4)	16 (10.9)

Supplement S2 – Classification of Generally Encourages Food choices (GEF)

The classification generally followed the criteria for Keyhole-labelling as described elsewhere [1]. Exceptions were made for the following types of foods that were evaluated according to the criteria for close resembling food groups.

Foods directed at children –	Mixed dishes (typically canned) are categorised according to the criteria for corresponding composite dishes.
Savoury biscuits and crackers –	Categorised according to criteria for bread
Ice-cream –	Categorised according to criteria for dairy products
Mayonnaise –	Categorised according to criteria for sauces
Seeds and quinoa –	Categorised according to criteria for nuts

[1] Swedish Food Agency. LIVSFS 2021:1. The Swedish Food Agency's Code of Statutes: Regulations Amending the Swedish Food Agency's Regulations (SLVFS 2005:9) on the Use of the Keyhole Symbol

Supplement Table S3. Average requirements (AR) for selected vitamins and minerals from Nordic Nutrition Recommendations 2023.

**Age interval (years)**		**1-3**	**4-6**	**7-10**	**11-14**		**15-17**		**18-24**		**25-50**		**51-70**		**>70**	
**Sex**					**F**	**M**	**F**	**M**	**F**	**M**	**F**	**M**	**F**	**M**	**F**	**M**
**AR**																
Vitamin D	µg	7.5	7.5	7.5	7.5	7.5	7.5	7.5	7.5	7.5	7.5	7.5	7.5	7.5	7.5	7.5
Vitamin C	mg	20	30	45	60	65	75	85	75	90	75	90	75	90	75	90
Folate	µg	90	110	160	220	200	240	250	250	250	250	250	250	250	250	250
Calcium	mg	400	700	675	980	980	980	980	870	870	750	750	750	750	750	750
Iron	mg	6	5	7	10	9	9	9	9	7	9	7	6	7	6	7
**Provisional AR***																
Vitamin E	α-TE	6	7	7	8	9	9	10	8	9	8	9	8	9	8	9
Vitamin B12	µg	1.2	1.4	2	2.8	2.6	3.1	3.2	3.2	3.2	3.2	3.2	3.2	3.2	3.2	3.2
Potassium	mg	700	900	1450	1900	2050	2250	2700	2800	2800	2800	2800	2800	2800	2800	2800
Selenium	µg	15	20	35	50	50	55	70	60	70	60	70	60	70	60	70

*Provisional average requirement (AR) calculated as 0.8 times the provisional recommended intake, assuming a CV of 12.5%.

Supplement Table S4. Reference values from Nordic Nutrition Recommendations (NNR2023) used for the calculation of Nutrient Rich Foods index (NRF).

	Ages, years:	1-3	4-6	7-10	11-14		15-17		18-24		25-50		51-70		>70	
	Sex:				F	M	F	M	F	M	F	M	F	M	F	M
**Qualifying nutrients**	**Unit**															
Protein*	E%	15	15	15	15	15	15	15	15	15	15	15	15	15	15	15
Dietary fiber	g/MJ	2	2	2	2.5	2.5	2.5	2.5	3	3	3	3	3	3	3	3
Vitamin A	RE	300	350	450	650	700	650	750	700	800	700	800	700	800	650	750
Vitamin C	Mg	25	35	55	75	80	90	105	95	110	95	110	95	110	95	110
Vitamin D	µg	10	10	10	10	10	10	10	10	10	10	10	10	10	20	20
Vitamin E	α-TE	7	8	9	10	11	11	12	10	11	10	11	9	11	9	11
Folate	µg	120	140	200	280	260	310	320	330	330	330	330	330	330	330	330
Iron	Mg	7	7	9	13	11	15	11	15	9	15	9	8	9	7	9
Calcium	Mg	450	800	800	1150	1150	1150	1150	1000	1000	950	950	950	950	950	950
Potassium	Mg	850	1150	1800	2400	2550	2850	3400	3500	3500	3500	3500	3500	3500	3500	3500
Magnesium	Mg	170	230	230	250	300	250	300	300	350	300	350	300	350	300	350
**Disqualifying nutrients**																
Saturated fat	max. E%	10	10	10	10	10	10	10	10	10	10	10	10	10	10	10
Free and added sugars	max. E%	10	10	10	10	10	10	10	10	10	10	10	10	10	10	10
NaCl†	max. g	2.75	3.50	4.25	5	5	5.75	5.75	5.75	5.75	5.75	5.75	5.75	5.75	5.75	5.75
Ref. energy intake‡	MJ/d	4.6	6.3	7.8	9.2	10.5	10.1	12.7	9.4	11.8	9.0	11.3	8.3	10.3	8.2	10.1
	Kcal/d	1099	1506	1864	2199	2510	2414	3035	2247	2820	2151	2701	1984	2462	1960	2414

Reference values are used to derive nutrient density benchmarks and are not interpreted as intake adequacy thresholds. For the calculation of NRF, the values presented here are scaled to 100 kcal using reference energy intake.

*The mean of the recommended range 10-20 E%.

† Reference values for NaCl for children and adolescents up to 14 years are extrapolated from recommended maximum amount for adults based on energy intake.

‡ Reference values for energy intakes assuming an average physical activity level (PAL) of 1.6 for adults and children 4-10 years, PAL 1-3 for children 1-3 years and PAL 1.7 for children 11-17 years. For conversions between MJ to kcal, a factor of 1 MJ = 239 kcal was used, resulting in a conversion multiplier of 4.184

Supplement Figure S5. Exclusion flowchart for regression analyses with BMI / BMI z-score and overweight/obesity as outcomes

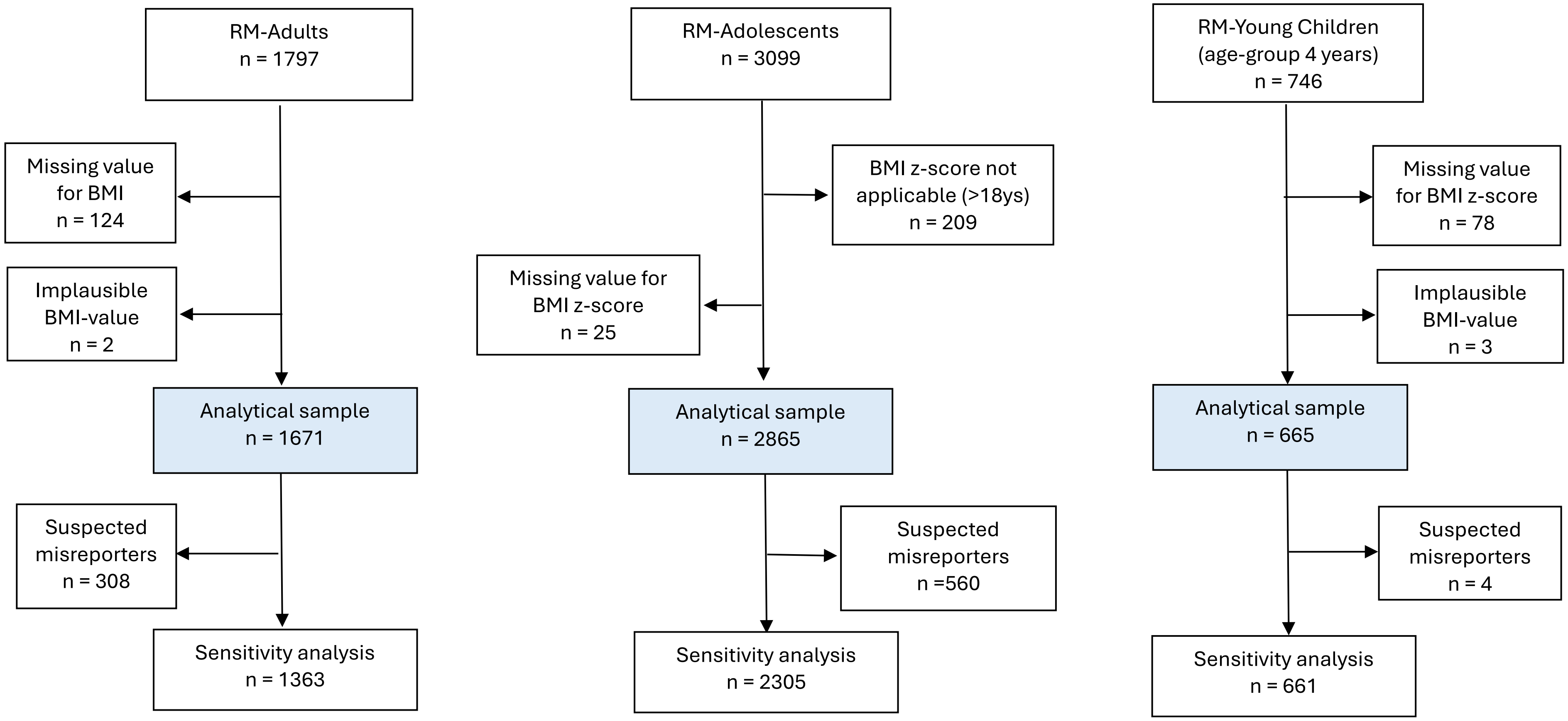

Supplement S6. Calculation of predictive interval for identifying suspected misreporters of energy intake

Step 1: Predictive equation for total energy expenditure

ln(TEE) =		
- 0.2172		
+ ln(Bodyweight)	x	4.167×10^{-1}
+ Height	x	6.565×10^{-3}
- Age	x	2.054×10^{-2}
+ Age^2	x	3.308×10^{-4}
- Age^3	x	1.852×10^{-6}
+ ln(Elevation)	x	9.125×10^{-2}
- Sex	x	4.092×10^{-2}
+ Ethnicity	x	3.589×10^{-3}
- Height x ln(Elevation)	x	6.759×10^{-4}
+ Age x ln(Elevation)	x	2.018×10^{-3}
- Age^2 x ln(Elevation)	x	2.262×10^{-5}
- Sex x ln(Elevation)	x	6.947×10^{-3}

Explanations:

TEE	Total Energy Expenditure/day in megajoules
Bodyweight	In kilograms
Height	In centimetres
Elevation	Elevation over sea-level in meters. Set to 30 for all participants based on a weighted average of the three most populated metropolitan areas in Sweden (Stockholm, Gothenburg and Malmö)
Sex	Coded -1 for males and +1 for females
Ethnicity	Not assessed in the Swedish national dietary survey. Coefficient for "NA" used for all participants.

Step 2. Calculation of predictive intervals

Lower 95% PI = (*p*TEE x 0.7466) – 1.5405

Upper 95% PI = (*p*TEE x 1.1396) + 2.7668

Where *p*TEE is the predicted mean TEE (MJ d^{-1}) obtained from exponentiating the result from the equation in step 1.

Conversion factor used: 1 MJ = 239.006 kcal.

Supplement Table S7. Inter-rater reliability for ultra-processed food classification by food group

Foodgroup	n	κ	95% CI		% Agreement
			lower	upper	
Alcoholic beverages	32	0.80	0.50	1.00	93.8
Bread	80	0.59	0.43	0.76	80.0
Breakfast cereals	57	0.81	0.64	0.99	93.0
Cereals	38	0.48	-0.12	1.00	94.7
Cheese	36	0.72	0.50	0.95	86.1
Children's cereal products	21	NA	NA	NA	100.0
Children's food	18	1.00	1.00	1.00	100.0
Children's formulae	2	NA	NA	NA	100.0
Coffee, tea and similar	24	0.70	0.46	0.94	83.3
Dairy desserts	35	0.78	0.51	1.00	91.4
Egg and egg products	8	NA	NA	NA	100.0
Egg dishes	13	0.57	0.03	1.00	84.6
Fat	39	0.85	0.68	1.00	92.3
Fine bakery wares	125	0.58	0.39	0.76	80.8
Fish and fish products	79	0.63	0.42	0.84	87.3
Fish dishes	48	0.29	-0.06	0.64	70.8
Fruit products	81	0.74	0.58	0.90	88.9
Fruits	54	NA	NA	NA	100.0
Juices	23	0.31	-0.15	0.77	56.5
Legume dishes	25	0.86	0.73	1.00	84.0
Meat and dairy imitations	65	0.37	-0.11	0.85	95.4
Meat and meat products	186	0.87	0.80	0.94	93.5
Meat dishes	147	0.76	0.65	0.87	86.4
Milk, yoghurt and similar	86	0.87	0.78	0.97	91.9
Nuts and legumes	57	0.44	0.07	0.82	89.5
Pasta and similar	17	1.00	1.00	1.00	100.0
Pasta dishes	51	0.68	0.45	0.90	80.4
Potato dishes	24	0.78	0.59	0.96	79.2
Sandwiches, pizzas and similar	77	0.38	0.17	0.58	61.0
Savory sauces and condiments	147	0.68	0.53	0.82	85.0
Savory snacks	39	0.69	0.38	0.99	89.7
Soups and salads	99	0.71	0.62	0.80	67.7
Starchy roots and tubers	15	NA	NA	NA	100.0
Sugar and confectionery	76	0.69	0.44	0.95	93.4
Sugar-sweetened beverages	21	NA	NA	NA	100.0
Supplements	12	0.13	-0.17	0.43	83.3
Vegetable dishes	76	0.71	0.54	0.88	80.3
Vegetables	174	0.80	0.65	0.96	96.6
Water based beverages	11	1.00	1.00	1.00	100.0
TOTAL	2218	0.75	0.72	0.78	86.9

Weighted Cohen's kappa for inter-rater reliability between two raters when classifying food items into one of three groups: UPF, partly UPF or non-UPF. CI, confidence interval; NA, not applicable. NA indicates that weighted kappa could not be estimated for items with no variability in ratings.

.

Supplement Table S8. Top five food groups based on calories from ultra-processed foods by age group and sex.

	Overall		Males		Females	
	Top five food groups	UPF (E%†)	Top five food groups	UPF (E%†)	Top five food groups	UPF (E%†)
**RM-Adults**	Bread	9.5	Bread	9.9	Bread	9.1
	Meat and meat products	4.5	Meat and meat products	5.2	Fine bakery wares	4.3
	Sandwiches, pizzas and similar	4.0	Sandwiches, pizzas and similar	4.7	Meat and meat products	3.7
	Fine bakery wares	3.8	Fine bakery wares	3.3	Sugar and confectionery	3.7
	Sugar and confectionery	3.0	Savoury sauces and condiments	2.6	Sandwiches, pizzas and similar	3.3
Age groups						
**60-80 years**	Bread	9.2	Bread	9.8	Bread	8.5
	Fine bakery wares	4.9	Meat and meat products	5.6	Fine bakery wares	5.2
	Meat and meat products	4.9	Fine bakery wares	4.7	Meat and meat products	4.0
	Breakfast cereals	2.6	Breakfast cereals	2.6	Breakfast cereals	2.6
	Savoury sauces and condiments	2.2	Sandwiches, pizzas and similar	2.0	Savoury sauces and condiments	2.3
**40-59 years**	Bread	9.6	Bread	10.0	Bread	9.1
	Meat and meat products	4.8	Meat and meat products	5.6	Fine bakery wares	4.3
	Fine bakery wares	3.7	Sandwiches, pizzas and similar	4.3	Meat and meat products	4.0
	Sandwiches, pizzas and similar	3.7	Fine bakery wares	3.2	Sugar and confectionery	3.7
	Sugar and confectionery	3.0	Savoury sauces and condiments	2.6	Sandwiches, pizzas and similar	2.9
**18-39 years**	Bread	9.6	Bread	9.7	Bread	9.5
	Sandwiches, pizzas and similar	6.2	Sandwiches, pizzas and similar	8.0	Sugar and confectionery	4.8
	Sugar and confectionery	3.9	Meat and confectionery	4.3	Sandwiches, pizzas and similar	4.7
	Meat and meat products	3.8	Savoury sauces and condiments	3.1	Fine bakery wares	3.7
	Savoury sauces and condiments	3.3	Sugar-sweetened beverages	3.1	Savoury sauces and condiments	3.5
**RM-Adolescents**	Sandwiches, pizzas and similar	8.2	Sandwiches, pizzas and similar	9.5	Bread	8.3
	Bread	7.7	Bread	7.1	Sandwiches, pizzas and similar	6.9
	Sugar and confectionery	5.8	Sugar and confectionery	4.9	Sugar and confectionery	6.8
	Sugar-sweetened beverages	3.8	Sugar-sweetened beverages	4.2	Fine bakery wares	3.5
	Savoury snacks	3.5	Savoury snacks	3.7	Sugar-sweetened beverages	3.4
Age groups						
**11th grade**	Sandwiches, pizzas and similar	9.5	Sandwiches, pizzas and similar	10.7	Sandwiches, pizzas and similar	8.5
	Bread	7.4	Bread	6.5	Bread	8.3
	Sugar and confectionery	4.7	Sugar-sweetened beverages	4.8	Sugar and confectionery	6.2
	Sugar-sweetened beverages	4.4	Savoury snacks	4.0	Savoury sauces and condiments	4.3
	Savoury snacks	3.8	Sugar and confectionery	3.2	Sugar-sweetened beverages	4.0
**8th grade**	Sandwiches, pizzas and similar	8.2	Sandwiches, pizzas and similar	9.4	Sugar and confectionery	8.1
	Bread	7.6	Bread	7.3	Bread	8.0
	Sugar and confectionery	7.3	Sugar and confectionery	6.6	Sandwiches, pizzas and similar	6.8
	Sugar-sweetened beverages	3.5	Sugar-sweetened beverages	4.0	Fine bakery wares	3.7

	Savoury snacks	3.4	Savoury snacks	3.7	Savoury snacks	3.1
**5th grade**	Bread	8.2	Sandwiches, pizzas and similar	8.5	Bread	8.7
	Sandwiches, pizzas and similar	6.9	Bread	7.7	Sugar and confectionery	6.1
	Sugar and confectionery	5.4	Sugar and confectionery	4.6	Sandwiches, pizzas and similar	5.4
	Meat and meat products	3.6	Meat and meat products	3.9	Savoury snacks	3.4
	Sugar-sweetened beverages	3.4	Sugar-sweetened beverages	3.7	Meat and meat products	3.3
**RM-Young Children**	Bread	9.1	Bread	9.5	Bread	8.7
	Children's cereal products	6.2	Children's cereal products	6.2	Children's cereal products	6.4
	Meat and meat products	5.4	Meat and meat products	5.4	Meat and meat products	5.5
	Fine bakery wares	2.7	Meat dishes	2.7	Fine bakery wares	2.7
	Meat dishes	2.5	Fine bakery wares	2.7	Meat dishes	2.3
Age groups						
**Four years**	Bread	9.8	Bread	10.4	Bread	9.2
	Meat and meat products	5.7	Meat and meat products	5.7	Meat and meat products	5.8
	Sugar and confectionery	4.0	Sugar and confectionery	4.0	Sugar and confectionery	4.1
	Fine bakery wares	3.9	Fine bakery wares	3.9	Fine bakery wares	3.8
	Meat dishes	2.6	Sandwiches, pizzas and similar	3.0	Meat dishes	2.3
**18 months**	Children's cereal products	10.5	Children's cereal products	10.7	Children's cereal products	10.3
	Bread	8.5	Bread	8.6	Bread	8.3
	Meat and meat products	5.1	Meat and meat products	5.1	Meat and meat products	5.2
	Meat dishes	2.5	Meat dishes	2.7	Sandwiches, pizzas and similar	2.4
	Sandwiches, pizzas and similar	2.1	Savoury sauces and condiments	2.0	Meat dishes	2.3

† Population-level E% of total caloric intake. Abbreviations: UPF, ultra-processed foods; E%, energy percentage; RM, Riksmaten (Swedish national dietary survey)

Supplement Table S9. Estimated change in intake of selected vitamins and minerals expressed as percentage of the average requirement (AR) per 10 E% higher ultra-processed food intake.

	Model 1			Model 2		
	β	95% CI	*p*-value	β	95% CI	*p*-value
**RM-Adults**						
Vitamin D	-5.9	(-8.2, -3.5)	<0.001	-8.0	(-9.9, -6.1)	<0.001
Vitamin E	1.5	(-0.7, 3.8)	0.204	-3.6	(-5.0, -2.1)	<0.001
Folate	-3.7	(-5.1, -2.3)	<0.001	-5.5	(-6.5, -4.6)	<0.001
Vitamin B12	-9.1	(-12,9, -5.2)	<0.001	-13.9	(-17.0, -10.9)	<0.001
Vitamin C	-5.4	(-8.6, -2.3)	0.001	-6.9	(-9.6, -4.2)	<0.001
Iron	0.3	(-1.9, 2.6)	0.764	-4.7	(-6.4, -3.1)	<0.001
Calcium	-2.4	(-4.8, 0.0)	0.063	-5.0	(-6.5, -3.6)	<0.001
Potassium	-2.9	(-4.4, -1.5)	<0.001	-6.6	(7.4, -5.8)	<0.001
Selenium	-4	(-5.5, -2.5)	<0.001	-5.9	(-6.8, -4.9)	<0.001
**RM-Adolescents**						
Vitamin D	-5.4	(-6.7, -4.1)	<0.001	-7.3	(-8.3, -6.3)	<0.001
Vitamin E	2.0	(0.4, 3.6)	0.019	-1.2	(-2.3, 0.0)	0.042
Folate	-5.3	(-6.9, -3.8)	<0.001	-9.3	(-10.3, -8.4)	<0.001
Vitamin B12	-8.7	(-11.2, -6.3)	<0.001	-15.4	(-17.7, -13.1)	<0.001
Vitamin C	-5.3	(-7.2, -3.5)	<0.001	-7.9	(-9.8, -5.9)	<0.001
Iron	0.9	(0.0, 1.7)	0.055	-2.2	(-2.7, -1.7)	<0.001
Calcium	-3.7	(-5.3, -2.0)	<0.001	-7.7	(-8.8, -6.6)	<0.001
Potassium	-3.1	(-4.4, -1.8)	<0.001	-6.9	(-7.7, -6.1)	<0.001
Selenium	-5.1	(-6.2, -4.0)	<0.001	-6.8	(-7.6, -6.0)	<0.001
**RM-Young Children**						
Vitamin D	-3.3	(-6.3, -0.3)	0.040	-4.1	(-6.7, -1.4)	0.004
Vitamin E	3.1	(1.1, 5.2)	0.004	2.2	(0.7, 3.8)	0.007
Folate	-10.6	(-13.1, -8.0)	<0.001	-11.0	(-12.7, -9.2)	<0.001
Vitamin B12	-16.5	(-20.8, -12.2)	<0.001	-16.9	(-20.8, -13.0)	<0.001
Vitamin C	-5.7	(-12.6, 1.2)	0.118	-3.9	(-10.2, 2.5)	0.237
Iron	6.6	(4.9, 8.4)	<0.001	5.7	(4.3, 7.2)	<0.001
Calcium	-0.9	(-3.1, 1.4)	0.455	-1.6	(-3.7, 0.5)	0.144
Potassium	-5.8	(-8.9, -2.7)	<0.001	-6.9	(-9.1, -4.7)	<0.001
Selenium	-6.7	(-9.0, -4.3)	<0.001	-6.2	(-8.0, -4.5)	<0.001

Results from multivariable quantile regression at the median. Model 1 adjusted for: Adults – age, sex, education, leisure-time physical activity and smoking; Adolescents – age group, sex, parental education, leisure-time physical activity; Young children – age group, sex, parental education, average daily screen time. Model 2 adjusted for all covariates in model 1 + energy intake. p-values adjusted for multiple testing using Benjamini-Hochberg method. The table complements Figure 3 in the main paper. Abbreviations: AR, average requirement; CI, confidence intervals; RM, Riksmaten (Swedish national dietary survey)

Supplement Table S10. Indicators of dietary quality by quintiles of dietary energy contribution from ultra-processed foods (UPF) classified according to the lower-bound scenario, with adjusted associations from quantile regression.

	Quintiles of E% UPF (range) Medians					Per 10 E% higher UPF		*p*-value‡
	**I**	**II**	**III**	**IV**	**V**	β†	95% CI	
**RM-Adults**	**(0–13.2)**	**(13.2–19.0)**	**(19.0–24.3)**	**(24.4–31.6)**	**(31.6–98.5)**			
**n =**	**360**	**360**	**359**	**359**	**359**			
**UPF, *E%***	**9.5**	**16.1**	**21.3**	**27.3**	**37.2**			
Energy intake, *kcal/day*	1759	1918	1916	1937	2040	49	(18.50, 79.13)	0.002
Energy density, *kcal/g*	1.39	1.50	1.56	1.61	1.72	0.09	(0.07, 0.10)	<0.001
Protein, *E%*	17.9	16.9	16.1	15.5	15.2	-0.9	(-1.10, -0.62)	<0.001
Dietary fiber, *g/1000 kcal*	11.2	10.5	9.6	9.5	9.0	-0.5	(-0.62, -0.32)	<0.001
Wholegrain, *g/1000 kcal*	24.4	22.2	19.1	18.5	15.5	-2	(-2.86, -1.16)	<0.001
Saturated fatty acids, *E%*	12.6	13.0	13.3	13.5	13.4	0.2	(-0.03, 0.35)	0.109
Added sugars, *E%*	3.6	5.5	6.1	7.4	9.6	1.9	(1.67, 2.06)	<0.001
Salt (NaCl), *g/1000 kcal*	4.0	3.9	3.8	3.8	3.7	-0.1	(-0.16, -0.07)	<0.001
GEF, *E%*	29.8	25.0	22.4	21.1	16.6	-2.6	(-3.11, -2.00)	<0.001
NRF11.3-S	6.9	6.6	6.2	6.0	5.5	-0.4	(-0.45, -0.33)	<0.001
**RM-Adolescents**	**(0–12.1)**	**(12.1–20.2)**	**(20.2–28.6)**	**(28.6–39.1)**	**(39.1–93.4)**			
**n =**	**620**	**620**	**620**	**620**	**619**			
**UPF, *E%***	**6.7**	**16.1**	**24.4**	**33.5**	**46.4**			
Energy intake, *kcal/day*	1808	1938	1996	2085	2149	86	(62.9, 109.5)	<0.001
Energy density, *kcal/g*	1.56	1.65	1.73	1.78	1.96	0.09	(0.08, 0.11)	<0.001
Protein, *E%*	18.0	17.2	16.4	15.4	14.0	-1.0	(-1.11, -0.94)	<0.001
Dietary fiber, *g/1000 kcal*	9.5	8.8	8.2	8.0	6.8	-0.6	(-0.71, -0.57)	<0.001
Wholegrain, *g/1000 kcal*	12.5	11.5	11.5	9.5	6.8	-1.3	(-1.56, -0.96)	<0.001
Saturated fatty acids, *E%*	13.0	13.6	14.0	13.7	14.3	0.3	(0.16, 0.38)	<0.001
Added sugars, *E%*	4.6	7.2	9.1	11.6	14.7	2.5	(2.31, 2.63)	<0.001
Salt (NaCl), *g/1000 kcal*	4.3	4.1	3.9	3.8	3.5	-0.2	(-0.23, -0.16)	<0.001
GEF, *E%*	16.9	14.0	11.7	10.3	7.6	-2.0	(-2.29, -1.76)	<0.001
NRF11.3-S	5.8	5.4	5.0	4.7	3.7	-0.5	(-0.53, -0.45)	<0.001
**RM-Young children**	**(0–16.2)**	**(16.2–24.0)**	**(24.0–32.2)**	**(32.3–41.8)**	**(41.9–82.6)**			
**n =**	**364**	**364**	**364**	**364**	**364**			
**UPF, *E%***	**10.4**	**19.8**	**28.2**	**36.8**	**49.4**			
Energy intake, *kcal/day*	1213	1241	1282	1278	1193	1.0	(-9.8, 12.2)	0.834
Energy density, *kcal/g*	1.3	1.4	1.4	1.5	1.5	0.04	(0.03, 0.05)	<0.001
Protein, *E%*	15.3	14.9	14.8	14.2	13.5	-0.4	(-0.53, -0.32)	<0.001
Dietary fiber, *g/1000 kcal*	12.2	11.3	10.6	10.0	9.4	-0.7	(-0.79, -0.56)	<0.001
Wholegrain, *g/1000 kcal*	22.4	19.8	19.5	19.0	19.5	-0.8	(-1.57, 0.03)	0.071
Saturated fatty acids, *E%*	13.5	13.7	13.2	13.4	12.9	-0.2	(-0.31, -0.02)	0.030
Added sugars, *E%*	2.8	4.2	5.1	5.9	5.5	0.7	(0.53, 0.79)	<0.001
Salt (NaCl), *g/1000 kcal*	3.6	3.6	3.5	3.6	3.3	-0.1	(-0.1, -0.02)	0.006
GEF, *E%*	26.8	22.4	19.5	16.7	13.1	-2.8	(-3.23, -2.45)	<0.001
NRF11.3-S	7.4	7.1	7.1	6.8	7.0	-0.1	(-0.18, -0.07)	<0.001

†Estimates from multivariable quantile regression at the median (τ = 0.5); ‡ *p*-values for the association with UPF intake, modelled continuously (per 10 E% increase), with correction for multiple comparisons across nutrients within each age group using the Benjamini–Hochberg method. Covariates: Adults—age, sex, education, leisure-time physical activity, smoking; Adolescents—age group, sex, parental education, leisure-time physical activity; Young Children—age group, sex, parental education, average daily screen time. Medians are shown for the full samples; regression estimates are based on complete cases. Abbreviations: CI, confidence interval; GEF, generally encouraged food choices; NRF11.3-S, Nutrient Rich Foods index 11.3–Swedish version; RM, Riksmaten (Swedish national dietary survey)

Supplement Table S11 Density of selected vitamins and minerals by quintiles of dietary energy contribution from ultra-processed foods (UPF) classified according to the lower-bound scenario, with adjusted associations from quantile regression.

	Quintiles of E% UPF (range) Median					Per 10 E% higher UPF		*p*-value‡
	**I**	**II**	**III**	**IV**	**V**	β†	95% CI	
**RM-Adults**	**(0–13.2)**	**(13.2–19.0)**	**(19.0–24.3)**	**(24.4–31.6)**	**(31.6–98.5)**			
**n =**	**360**	**360**	**359**	**359**	**359**			
**UPF, *E%***	**9.5**	**16.1**	**21.3**	**27.3**	**37.2**			
Vitamin D, *µg/1000 kcal*	3.7	3.3	2.9	2.8	2.4	-0.2	(-0.35, -0.15)	<0.001
Vitamin E, *α-TE/1000 kcal*	5.8	5.6	5.4	5.3	4.9	-0.2	(-0.28, -0.13)	<0.001
Folate, *µg/1000 kcal*	147	138	127	120	111	-9	(-11.00, -7.16)	<0.001
Vitamin B12, *µg/1000 kcal*	2.9	2.7	2.4	2.4	2.1	-0.2	(-0.25, -0.11)	<0.001
Vitamin C, *mg/1000 kcal*	53.2	45.8	44.3	41.4	35.6	-2.9	(-4.12, -1.71)	<0.001
Iron, *mg/1000 kcal*	5.6	5.3	5.0	5.0	4.7	-0.2	(-0.30, -0.14)	<0.001
Calcium, *mg/1000 kcal*	470	440	441	428	409	-18	(-26.4, -9.3)	<0.001
Potassium, *mg/1000 kcal*	1782	1631	1576	1524	1412	-90	(-108.5, -72.0)	<0.001
Selenium, *µg/1000 kcal*	26.7	23.5	21.8	20.5	18.2	-2.3	(-2.65, -1.88)	<0.001
**RM-Adolescents**	**(0–12.1)**	**(12.1–20.2)**	**(20.2–28.6)**	**(28.6–39.1)**	**(39.1–93.4)**			
**n =**	**620**	**620**	**620**	**620**	**619**			
**UPF, *E%***	**6.7**	**16.1**	**24.4**	**33.5**	**46.4**			
Vitamin D, *µg/1000 kcal*	2.9	2.9	2.6	2.4	1.8	-0.3	(-0.30, -0.23)	<0.001
Vitamin E, *α-TE/1000 kcal*	5.6	5.6	5.2	5.0	4.7	-0.3	(-0.32, -0.21)	<0.001
Folate, *µg/1000 kcal*	147	134	122	115	99	-11	(-12.11, -10.2)	<0.001
Vitamin B12, *µg/1000 kcal*	2.5	2.5	2.3	2.1	1.8	-0.2	(-0.22, -0.16)	<0.001
Vitamin C, *mg/1000 kcal*	38.8	33.5	28.9	27.8	24.1	-3.4	(-3.94, -2.84)	<0.001
Iron, *mg/1000 kcal*	4.1	3.9	3.8	3.8	3.7	-0.1	(-0.12, -0.07)	<0.001
Calcium, *mg/1000 kcal*	543	517	493	469	410	-29	(-34.3, -23.2)	<0.001
Potassium, *mg/1000 kcal*	1469	1394	1309	1275	1146	-72	(-80.7, -63.5)	<0.001
Selenium, *µg/1000 kcal*	21.4	19.9	19.0	17.1	14.3	-1.6	(-1.83, -1.44)	<0.001
**RM-Young children**	**(0–16.2)**	**(16.2–24.0)**	**(24.0–32.2)**	**(32.3–41.8)**	**(41.9–82.6)**			
**n =**	**364**	**364**	**364**	**364**	**364**			
**UPF, *E%***	**10.4**	**19.8**	**28.2**	**36.8**	**49.4**			
Vitamin D, *µg/1000 kcal*	9.3	7.1	6.7	6.7	7.7	-0.2	(-0.37, -0.04)	0.018
Vitamin E, *α-TE/1000 kcal*	6.3	6.2	6.5	6.5	6.4	0	(-0.05, 0.12)	0.517
Folate, *µg/1000 kcal*	147	137	131	127	118	-7	(-7.9, -5.38)	<0.001
Vitamin B12, *µg/1000 kcal*	2.6	2.4	2.3	2.3	2.0	-0.1	(-0.16, -0.08)	<0.001
Vitamin C, *mg/1000 kcal*	53.2	50.8	49.1	51.7	58.9	1.5	(0.44, 2.63)	0.009
Iron, *mg/1000 kcal*	4.0	4.1	4.5	4.4	6.0	0.4	(0.29, 0.45)	<0.001
Calcium, *mg/1000 kcal*	540	545	544	542	545	2	(-7.08, 10.78)	0.723
Potassium, *mg/1000 kcal*	1493	1434	1431	1389	1351	-30	(-42.73, -16.89)	<0.001
Selenium, *µg/1000 kcal*	17.7	16.7	15.9	15.5	14.6	-0.6	(-0.85, -0.38)	<0.001

†Estimates from multivariable quantile regression at the median ($\tau = 0.5$); ‡ *p*-values for the association with UPF intake, modelled continuously (per 10 E% increase), with correction for multiple comparisons across nutrients within each age group using the Benjamini–Hochberg method. Covariates: Adults—age, sex, education, leisure-time physical activity, smoking; Adolescents—age group, sex, parental education, leisure-time physical activity; Young Children—age group, sex, parental education, average daily screen time. Medians are shown for the full samples; regression estimates are based on complete cases. Abbreviations: CI, confidence interval; RM, Riksmaten (Swedish national dietary survey)

Supplement Table S12. Linear regression models of dietary contribution of ultra-processed foods (per 10 E%) in relation to BMI and BMI *z*-score.

	n	Scenario‡								
		Middle-bound			Lower-bound			Upper-bound		
		Estimate†	(95% CI)	*p*	Estimate†	(95% CI)	*p*	Estimate†	(95% CI)	*p*
RM-Adults	1671									
Model 1[a]		0.61	(0.04, 1.18) %	0.036	0.62	(-0.07, 1.31) %	0.076	0.54	(-0.01, 1.10) %	0.056
Model 2[d]		0.72	(0.15, 1.30) %	0.013	0.71	(-0.02, 1.40) %	0.045	0.67	(0.10, 1.23) %	0.020
Model 3[e]		0.72	(0.09, 1.36) %	0.026	0.66	(-0.08, 1.40) %	0.081	0.67	(0.03, 1.30) %	0.039
RM- Adolescents	2865									
Model 1[b]		-0.01	(-0.04, 0.01)	0.269	-0.03	(-0.05, -0.01)	0.015	0.02	(0.00, 0.04)	0.106
Model 2[d]		0.00	(-0.04, 0.06)	0.746	-0.02	(-0.04, 0.00)	0.110	0.03	(0.01, 0.05)	0.012
Model 3[e]		-0.02	(-0.04, 0.01)	0.194	-0.05	(-0.07, -0.02)	0.002	0.02	(0.00, 0.05)	0.070
RM-Young Children, 4ys	665									
Model 1[c]		0.01	(-0.04, 0.06)	0.697	-0.02	(-0.08, 0.04)	0.480	0.01	(-0.04, 0.06)	0.762
Model 2[d]		0.01	(-0.04, 0.06)	0.744	-0.02	(-0.08, 0.03)	0.430	0.01	(-0.05, 0.06)	0.821
Model 3[e]		0.02	(-0.04, 0.08)	0.523	-0.02	(-0.08, 0.04)	0.542	0.02	(-0.04, 0.07)	0.587

†For Adults, BMI was log-transformed for regression analyses; results are back-transformed and expressed as percent change in BMI per 10 E% increase in ultra-processed food intake. For Adolescents (<18 years old) and Young Children (four years old age group), BMI *z*-score was modelled directly; results are expressed as absolute change in *z*-score per 10 E% increase in ultra-processed food intake. ‡ Middle-bound representing the most likely degree of processing, lower-bound the lowest realistic degree of processing and upper-bound the highest realistic degree of processing.

[a]Model 1: age, sex, education, leisure-time physical activity and smoking

[b]Model 1: age-group, sex, education and leisure-time physical activity

[c]Model 1: age, sex, education and average daily screentime

[d]Model 1 + energy intake

[e]Model 2 + diet quality as captured by the NRF11.3-S score

Abbreviations: CI, confidence interval; RM, Riksmaten (Swedish national dietary survey)

Supplement Table S13. Prevalence ratios for overweight or obesity across tertiles of dietary contribution from ultra-processed foods.

	Scenario†								
	Middle-bound			Lower-bound			Upper-bound		
	PR	(95% CI)	*p*	PR	(95% CI)	*p*	PR	(95% CI)	*p*
**RM-Adults**[a]									
T1 (reference)									
T2	0.95	(0.84, 1.07)	0.408	0.99	(0.87, 1.11)	0.810	1.08	(0.95, 1.21)	0.233
T3	1.12	(1.00, 1.27)	0.060	1.12	(0.99, 1.26)	0.081	1.17	(1.03, 1.32)	0.016
**RM-Adolescents**[b]									
T1 (reference)									
T2	1.00	(0.84, 1.18)	0.967	0.97	(0.82, 1.15)	0.703	1.03	(0.87, 1.24)	0.707
T3	0.89	(0.74, 1.06)	0.183	0.84	(0.71, 1.01)	0.059	1.06	(0.89, 1.26)	0.531
**RM-Young Children, 4 years**									
T1 (reference)									
T2	0.88	(0.45, 1.72)	0.716	0.97	(0.51, 1.83)	0.918	1.23	(0.65, 2.34)	0.522
T3	1.37	(0.74, 2.51)	0.312	1.25	(0.69, 2.27)	0.465	1.36	(0.73, 2.55)	0.330

Prevalence ratios, assessed by Poisson regression with robust variance. †Middle-bound scenario classifying foods according to the most likely degree of processing. Lower-bound scenario classifying foods according to the lowest realistic degree of processing. Upper-bound scenario classifying foods according to the highest realistic degree of processing.

[a]Adjusted for age, sex, education, leisure-time physical activity and smoking

[b]Adjusted for age group, sex, parental education and leisure-time physical activity

[c]Adjusted for age, sex, parental education and average daily screentime

Abbreviations: CI, confidence interval; PR, prevalence ratio; RM, Riksmaten (Swedish national dietary survey); T, tertile.
